# The LysE Superfamily of Transport Proteins Involved in Cell Physiology and Pathogenesis

**DOI:** 10.1371/journal.pone.0137184

**Published:** 2015-10-16

**Authors:** Brian V. Tsu, Milton H. Saier

**Affiliations:** Department of Molecular Biology, Division of Biological Sciences, University of California at San Diego, La Jolla, California, United States of America; Vanderbilt University, UNITED STATES

## Abstract

The LysE superfamily consists of transmembrane transport proteins that catalyze export of amino acids, lipids and heavy metal ions. Statistical means were used to show that it includes newly identified families including transporters specific for (1) tellurium, (2) iron/lead, (3) manganese, (4) calcium, (5) nickel/cobalt, (6) amino acids, and (7) peptidoglycolipids as well as (8) one family of transmembrane electron carriers. Internal repeats and conserved motifs were identified, and multiple alignments, phylogenetic trees and average hydropathy, amphipathicity and similarity plots provided evidence that all members of the superfamily derived from a single common 3-TMS precursor peptide via intragenic duplication. Their common origin implies that they share common structural, mechanistic and functional attributes. The transporters of this superfamily play important roles in ionic homeostasis, cell envelope assembly, and protection from excessive cytoplasmic heavy metal/metabolite concentrations. They thus influence the physiology and pathogenesis of numerous microbes, being potential targets of drug action.

## Introduction

Members of the LysE superfamily have long been known to catalyze solute export [1]. Three families had been shown to comprise this novel superfamily: (i) L-lysine and L-arginine exporters (LysE); (ii) homoserine/threonine resistance proteins (RhtB); and (iii) cadmium ion resistance proteins (CadD) [1]. While LysE and RhtB proteins catalyze export of amino acids, the more distant CadD proteins are involved in efflux of the heavy metal ion, cadmium (Cd^2+^) [1,2,3]. Most members of these families share similar sizes, around 200 amino acyl residues, similar hydrophobicity plots indicative of 6 transmembrane α-helical segments (TMSs), high degrees of sequence similarity within but not between families and prokaryotic origins [1].

In this paper, we report investigations allowing expansion of the LysE superfamily to include members from all three domains of life. Using computational methods, we demonstrate that the previously established members of this superfamily are homologous to members of eight additional families: (i) tellurium ion resistance proteins (TerC); (ii) iron/lead transporters (ILT); (iii) Mn^2+^ exporters (MntP); (iv) Ca^2+^/H^+^ antiporters-2 (CaCA2); (v) Ni^2+^/Co^2+^ transporters (NicO); (vi) neutral amino acid transporters (NAAT); (vii) peptidoglycolipid addressing proteins (GAP); and (viii) disulfide bond oxidoreductase D proteins (DsbD). We confirm this expansion and provide superfamily descriptions with thorough analyses of identified internal repeats and conserved motifs, multiple alignments of identified homologues, phylogenetic trees and average hydropathy, amphipathicity and similarity plots. The superfamily phylogenetic tree shows the relationships of these eleven families to each other.

## Materials and Methods

### Potential New Families

Previously established members of the LysE superfamily were initially examined in the Transporter Classification Database (TCDB; www.tcdb.org) [4]. PSI-BLAST searches with iterations against TCDB (TC-BLAST) were conducted to locate distant homologues with overlapping TMSs. The Web-based Hydropathy, Amphipathicity & Topology (WHAT) program was used to generate hydropathy plots for preliminary topological predictions of individual proteins [5]. Established families within the LysE superfamily are listed in Table 1 with previously assigned transporter classification numbers (TC#) from TCDB.

**Table 1:** Characteristics of all families in the LysE superfamily included in this study.

Family Name	Family Abbreviation	Transporter Classification No. (TC) #	Relative Family size^a^	Average Protein Size^b^	# TMSs^c^	# Subfamilies in TCDB^d^	Established Substrates (S)	Polarity of transport	Taxonomic Distribution	References
L-Lysine Exporter	LysE	2.A.75	1799	204 ± 20	6	1	D- and L-lysine, histidine and arginine	in—> out	Bacteria	[37]
Resistance to Homoserine/Threonine	RhtB	2.A.76	2711	207 ± 14	5, 6	2	O-aetylserine/cysteine/azaserine, threonine, serine, homoserine, homoserine lactones, leucine, alanine, 3-methyarginine and pimaricin-inducer PI-factor	in—> out	Bacteria	[38, 39, 40, 41, 42]
Cadmium Resistance	CadD	2.A.77	578	210 ± 68	4, 5, 6, 7	1	cadmium ions	in—> out	Bacteria	[3]
Neutral Amino Acid Transporter	NAAT	2.A.95	588	207 ± 17	6	1	glycine, L-alanine, L-serine, L-threonine and a variety of neutral L-amino acids	in—> out	Bacteria, Archaea	[26]
Ca^2+^:H^+^ Antiporter-2	CaCA2	2.A.106	1852	252 ± 106	5, 6, 7	4	calcium ions	cytoplasm—> golgi lumen	Bacteria, Archaea, Eukaryota	[23]
Mn^2+^ exporter	MntP	2.A.107	298	188 ± 14	6	3	manganese ions	in—> out	Bacteria, Archaea	[25]
Iron/Lead Transporter	ILT	2.A.108	1063	350 ± 128	6, 7, 8	3	iron and lead ions	out—> in	Bacteria, Archaea	[22]
Tellurium Ion Resistance	TerC	2.A.109	2592	328 ± 41	7, 8, 9	3	tellurium ions	in—> out	Bacteria, Archaea, Eukaryota	[32]
Nickel/cobalt Transporter	NicO	2.A.113	539	345 ± 111	5, 6, 7	2	nickel and cobalt ions	in—> out	Bacteria, Archaea, Eukaryota	[35]
Peptidoglycolipid Addressing Protein	GAP	2.A.116	113	233 ± 41	6	3	peptidoglycolipids	in—> out	Bacteria, Archaea	[36]
Disulfide Bond Oxidoreductase D	DsbD	5.A.1	1981	533 ± 189	6, 8, 9	6	electrons	cytoplasm—> periplasm	Bacteria, Archaea, Eukaryota	[43]

^a^A single search with the first protein in TCDB (x.x.x.1.1) was used as the query sequence to BLAST the NCBI protein database with a 95% cutoff. The BLAST searches were run on July 22, 2013.

^b^Average number of amino acyl residues in the proteins retrieved by Protocol1 for column 4.

^c^Dominant numbers of predicted TMSs for the proteins retrieved by Protocol1 for column 4.

^d^Number of subfamilies currently included in TCDB.

### Obtaining Homologues

A single FASTA-formatted protein sequence was selected from TCDB and used as the input for Protocol1, a program available through the BioV Suite software [6]. With Protocol1, we utilize NCBI PSI-BLAST with a threshold of 0.80 to generate a list of non-redundant homologues. This setting ensured that only one of any set of proteins with greater than 80% identity would be retained [7]. Protocol1 was applied to proteins of each family in the study.

### Establishing Homology between Families

The FASTA-formatted homologue sequences generated with Protocol1 were used as input into another BioV Suite program, Protocol2. Protocol2 requires two such input files and generates a graphical report, displaying sequence alignments between homologous members of two different protein families [6]. Two sequences with strong TMS alignment and z-scores above the value of 13.0 standard deviations (S.D.) are considered sufficient to provide strong evidence of homology. The higher the z-score, the greater the sequence similarity [6]. The z-scores obtained with Protocol2 were then verified through the use of a TCDB web program, Global Sequence Alignment Tool (GSAT) [6]. Good scoring pairs of sequences identified with Protocol2 were then tested using 20,000 random shuffles (GSAT) for more accurate results. Once verified, the GSAT results were analyzed for TMS overlap through use of the TMS prediction program, HMMTOP [8]. The top comparison scores and number of aligned TMSs between each family are shown in Table 2. Finally, a GSAT comparison score, based on 2,000 random shuffles, was generated between sequences of query proteins and respective proteins obtained from Protocol1 to manually check for homology of A versus B and C versus D (Table 3) [9,10]. Specific proteins identified in this paper are reported with UniProt accession numbers (www.uniprot.org). Proteins lacking UniProt accession numbers are assigned NCBI (GenBank) accession numbers.

**Table 2:** Comparison scores between LysE superfamily members. Scores equal to or greater than 13.0 Standard Deviations (S.D.) are bolded. The number of aligned TMSs is included below each score. Comparisons with the negative control, the Mitochondrial Carrier (MC) family, are provided to the right of the bolded border.

	LysE	RhtB	CadD	CaCA2	MntP	ILT	TerC	NAAT	NicO	GAP	DsbD	MC
**LysE**		**20.1 (5TMSs)**	12.1 S.D. (4TMSs)	**13.5 S.D. (3TMSs)**	11.8 S.D. (3TMSs)	12.5 S.D. (2TMSs)	**14.6 S.D. (3TMSs)**	**14.0 S.D. (5TMSs)**	10.8 S.D. (6TMSs)	12.7 S.D. (3TMSs)	12.3 S.D. (5TMSs)	4.1 S.D. (0TMSs)
**RhtB**			11.9 S.D. (3TMSs)	**13.0 S.D. (4TMSs)**	**13.7 S.D. (3TMSs)**	**13.7 S.D. (3TMSs)**	**13.5 S.D. (3TMSs)**	**15.0 S.D. (5TMSs)**	**13.8 S.D. (6TMSs)**	**14.5 S.D. (5TMSs)**	**14.0 S.D. (5TMSs)**	8.8 S.D. (2TMSs)
**CadD**				**14.2 S.D. (3TMSs)**	**15.7 S.D. (4TMSs)**	**13.5 S.D. (6TMSs)**	**13.6 S.D. (4TMSs)**	**14.4 S.D. (5TMSs)**	**15.1 S.D. (6TMSs)**	12.3 S.D. (5TMSs)	11.5 S.D. (6TMSs)	8.5 S.D. (2TMSs)
**CaCA2**					**15.1 S.D. (3TMSs)**	**15.3 S.D. (3TMSs)**	**16.2 S.D. (3TMSs)**	12.0 S.D. (5TMSs)	12.5 S.D. (5TMSs)	11.6 S.D. (5TMSs)	**13.2 S.D. (5TMSs)**	10.5 S.D. (1TMS)
**MntP**						12.5 S.D. (6TMSs)	**13.5 S.D. (5TMSs)**	**15.1 S.D. (4TMSs)**	12.3 S.D. (5TMSs)	11.3 S.D. (4TMSs)	**16.0 S.D. (5TMSs)**	9.1 S.D. (2TMSs)
**ILT**							**13.1 S.D. (5TMSs)**	11.8 S.D. (6TMSs)	12.8 S.D. (6TMSs)	12.8 S.D. (6TMSs)	10.9 S.D. (4TMSs)	9.1 S.D. (1TMS)
**TerC**								**15.2 S.D. (3TMSs)**	**13.9 S.D. (5TMSs)**	12.1 S.D. (5TMSs)	12.9 S.D. (5TMSs)	4.4 S.D. (0TMSs)
**NAAT**									**13.5 S.D. (3TMSs)**	12.8 S.D. (4TMSs)	**15.3 S.D. (6TMSs)**	10.0 S.D. (1TMS)
**NicO**										12.7 S.D. (5TMSs)	**14.8 S.D. (5TMSs)**	9.3 S.D. (1TMS)
**GAP**											**13.1 S.D. (5TMSs)**	5.8 S.D. (2TMS)
**DsbD**												9.9 S.D. (1TMS)

**Table 3:** Use of the Superfamily Principle (transitivity rule) to establish homology: If A and B are homologous, B and C are homologous, and C and D are homologous, then A is homologous to D. Families being compared are presented in column 1. Uniprot IDs are provided in columns 2–5. When a Uniprot accession number is unavailable, an NCBI accession number is provided. Comparison scores, expressed in standard deviations (S.D.), are provided in columns 6–9. Columns 6–8 allow establishment of homology. Column 9 gives the value determined when A is compared to D directly. For example, in a comparison between LysE and RhtB, Protein A and Protein D are query proteins from each respective family. Protein B is a homologue of Protein A. Protein C is a homologue of Protein D. Comparisons with the negative control, the Mitochondrial Carrier (MC) family, are provided below the double-lined border.

	Proteins Compared (Accession numbers provided)				Score for each comparison (S.D.)			
Families Compared	Protein A	Protein B	Protein C	Protein D	A v B	B v C	C v D	A v D
LysEvRhtB	P94633	H3RH39	Q2SUV5	P76249	32.5	20.1	52.0	9.0
LysEvCadD	P64711	K0HW07	K9TWQ5	Q45153	37.0	12.1^a^	36.1	0.7
RhtBvCadD	P76249	G9Y0F1	G9WHF3	O05469	72.0	11.9^a^	36.0	1.1
LysE v CaCA2	P94633	E0MXD6	C1MR94	P52876	63.0	13.5	31.7	1.6
RhtB v CaCA2	P76249	G9Y0F1	K9ULS7	P52876	73.0	13.0	62.4	1.3
CadD v CaCA2	O05469	L2SR21	B7FUM2	P52876	50.7	14.2	57.2	2.0
RhtB v MntP	P76249	C4GM93	D9SW99	O27840	45.9	13.7	37.5	1.9
CadD v MntP	O05469	H3NKZ1	Q727E5	O27840	48.0	15.7	34.3	1.0
CaCA2 v MntP	P52876	E0UDP4	C0DV56	P76264	74.5	15.1	57.3	1.3
RhtB v ILT	P0AG34	A1RAR9	Q2NBF8	Q58AJ4	50.5	13.7	125.9	0.4
CadD v ILT	O05469	C2D135	G5JVH6	Q5HSD5	43.1	13.5	41.0	4.2
CaCA2 v ILT	P52876	F0Y333	Q97V64	Q4J7V8	52.7	15.3	67.2	5.3
LysE v TerC	P94633	D7GFT1	Q20ZD5	I3XAB3	40.8	14.6	72.7	-0.2
RhtB v TerC	P76249	K8W4X6	WP_010022951	B5UIP4	63.3	13.5	54.9	1.4
CadD v TerC	O05469	WP_010652183	G8LRD3	B5UIP4	46.0	13.6	38.5	3.9
CaCA2 v TerC	P52876	B7FUM2	D7V5X7	B5UIP4	57.2	16.2	62.9	1.3
MntP v TerC	P76264	E7S0L5	A2TWJ9	Q7UHX7	43.9	13.5	40.3	2.6
ILT v TerC	Q58AJ4	G6EJJ4	Q8KAT3	B5UIP4	125.3	13.1	37.6	0.7
LysE v NAAT	P11667	G8QX72	Q2C9W5	O32244	35.1	14.0	40.6	3.9
RhtB v NAAT	P0AG38	L7BNM7	H1S8A2	Q8J305	95.4	15.0	39.2	5.2
CadD v NAAT	Q45153	K6U069	E3T754	Q8J305	27.1	14.4	40.4	-0.1
MntP v NAAT	O27840	A6VQU4	WP_018748573	P67143	20.7	15.1	46.8	2.6
TerC v NAAT	I3XAB3	Q5L1S7	T2GCR6	P67143	26.2	15.2	45.5	3.0
RhtB v NicO	P0AG38	N9DHM2	G2TLK3	F8C138	68.9	13.8	34.5	1.2
CadD v NicO	Q45153	K9ZC80	K6XDF4	F8C138	24.8	15.1	22.4	0.2
TerC v NicO	I3XAB3	F4QZA6	M1YUV4	F8C138	55.7	13.9	32.8	1.4
NAAT v NicO	Q8J305	H1L1H6	WP_022692950	P76425	38.4	13.5	34.9	0.8
RhtB v GAP	P76249	F3KVR3	WP_019358971	K6W6C5	45.2	14.5	16.6	1.7
RhtB v DsbD	P0AG38	M4RA58	R1CD96	P45706	35.6	14.0	43.5	-0.2
CaCA2 v DsbD	B9MIH1	D1JG69	F9DXY9	P45706	23.2	13.2	77.7	-0.5
MntP v DsbD	E4RIT5	F7ZP38	F5SD76	P45706	28.2	16.0	70.7	0.6
NAAT v DsbD	Q8J305	Q8U2T5	K0NNX9	P45706	82.4	15.3	41.9	2.5
NicO v DsbD	B2JAZ6	K9Z039	M1ZHA3	P45706	34.2	14.8	43.2	0.2
GAP v DsbD	K6W6C5	WP_018161757	C6D6Q6	Q939U6	31.7	13.1	41.8	1.0
LysE v MC	P94633	G8QX72	XP_395934	P12235	35.7	4.1^a^	162.4	0.7
RhtB v MC	P76249	F3KVR3	I3WBB4	P12235	43.0	8.8^a^	157.0	1.0
CadD v MC	O05469	D2AZ49	XP_003796317	P12235	30.8	8.5^a^	200.7	1.6
CaCA2 v MC	G0PPC8	L7L942	Q4PMB2	P12235	17.5	10.5^a^	158.1	0.7
MntP v MC	O27840	L7VM13	S7NPK9	P12235	35.2	9.1^a^	153.6	-1.0
ILT v MC	Q5HSD5	L0W8N6	V9KQ68	P12235	48.2	9.1^a^	149.5	-1.4
TerC v MC	I3XAB3	K9CUK2	Q91336	P12235	48.9	4.4^a^	172.4	0.4
NAAT v MC	Q8J305	F9RL32	Q91336	P12235	42.7	10.0^a^	176.1	0.6
NicO v MC	F8C138	G9QNI4	S9XZZ3	P12235	33.1	9.3^a^	171.4	-0.3
GAP v MC	K6W6C5	WP_019971730	V9KQ68	P12235	10.1	5.8^a^	155.4	-0.6
DsbD v MC	P45706	B3E4Q5	XP_007059219	P12235	48.4	9.9^a^	159.0	-0.8

^a^These comparison scores are insufficient to establish homology.

### Viewing Average Hydropathy, Amphipathicity and Similarity Plots

Multiple alignments for each family in the study were generated using the ClustalX, Mafft and ProbCons programs [11,12,13]. The topologies of these sequences were then examined using AveHAS, a web-based program that displays the average hydropathy, amphipathicity and similarity plots for a set of homologues [14].

### Identifying Internal Repeats

The multiple alignment file produced from ClustalX was used as the input for IntraCompare, a program for the detection of internal repeats. Generated AveHAS plots for respective multiple alignment files were referenced to locate comparable regions of interest. IntraCompare generates comparison scores expressed in S.D. for non-overlapping regions of the same homologous proteins [15].

### Motif Analyses

Motif analyses were carried out using the MEME program (The MEME Suite; http://meme.nbcr.net/meme/) [16]. Default settings were used to search for ungapped, conserved residues within a given set of homologues. Results from HMMTOP were used to predict relationships between conserved regions relative to the TMSs. Motifs identified for each family were then paired to different families to observe similar residue conservation.

### Construction of Phylogenetic Trees

Phylogenetic trees were derived using multiple programs. RAxML and FastTree methods have been explored using raxmlgui [17]. Phylip-formatted multiple alignments generated using ClustalX, Mafft and Probcons were used as inputs to generate FastTree trees for each protein family in this study. In addition, a Phylip-formatted multiple alignment of members from all eleven families was generated from Mafft and used to create a set of 100 trees using the RAxML method of analysis [18]. The Mafft alignment used for the RAxML tree analysis was generated using the Mafft-homologs function with 200 homologs retrieved per input sequence at a threshold of 1e^-20^ [12]. All FastTree trees and the best tree indicated by the RAxML method were viewed using FigTree. SuperfamilyTree (SFT) [19,20,21,22,23,24,25,26] and TreeView [27] were also utilized. Agreement between 100 trees was evaluated. FASTA-formatted sequences corresponding to the TC families were inputted and used to compile tens of thousands of NCBI BLAST bit-scores upon which SFT trees were based. SFT and Fitch programs then generated a default of 100 superfamily trees based on the results. These 100 trees were used to create a consensus tree [19,20,21,22,23,24,25,26]. The parameters for these programs are described in S1 Fig.

## Results

In addition to the three previously established LysE superfamily members (Table 1), eight families were analyzed in this study: (i) CaCA2 (TC# 2.A.106); (ii) MntP (TC# 2.A.107); (iii) ILT (TC# 2.A.108); (iv) TerC (TC# 2.A.109); (v) NAAT (TC# 2.A.95); (vi) NicO (TC# 2.A.113); (vii) GAP (TC# 2.A.116) and (viii) DsbD (TC# 5.A.1) (Table 1). Mitochondrial carriers (TC# 2.A.29) were used as a negative control when generating comparison scores expressed in standard deviations (S.D.) using the GSAT program [6]. Like most members of the LysE superfamily, MC proteins have 6 TMSs but evolved via a different pathway [28]. They arose by triplication of a 2TMS-encoding genetic element, while LysE superfamily proteins arose by intragenic duplication of a 3TMS-encoding genetic element. Of the eight novel families, seven are included in the 2.A subclass of TCDB, secondary carrier-type facilitators known to catalyze symport, uniport and antiport. The exception, DsbD, is a family of transmembrane 2-electron transfer carriers with TC #5.A.1 [4,29,30].

Statistical evidence (Table 2) argued that the TerC, ILT, MntP, CaCA2, NAAT, NicO, GAP and DsbD families are related to the LysE, RhtB and CadD families. Multiple alignments additionally revealed that six TMSs align across all families included in this study. Statistical evidence for homology, multiple alignments of homologues, AveHAS plots, identified internal repeats, MEME/MAST diagrams of conserved motifs, and a proposed evolutionary pathway (evolutionary history) for this expanded superfamily are presented (Figs 1, 2, 3 and 4; S2, S3, S4, S5, S6, S7, S8, S9, S10, S11, S12, S13, S14, S15, S16, S17, S18, S19, S20, S21, S22, S23, S24, S25, S26 and S27 Figs; Tables 1, 2, 3, 4 and 5). In addition, our results confirm topological findings reported in previous studies regarding LysE, RhtB, CadD, MntP, ILT, CaCA2, NAAT and DsbD homologues [1,29,31,32,33,34,35].

**Fig 1:**
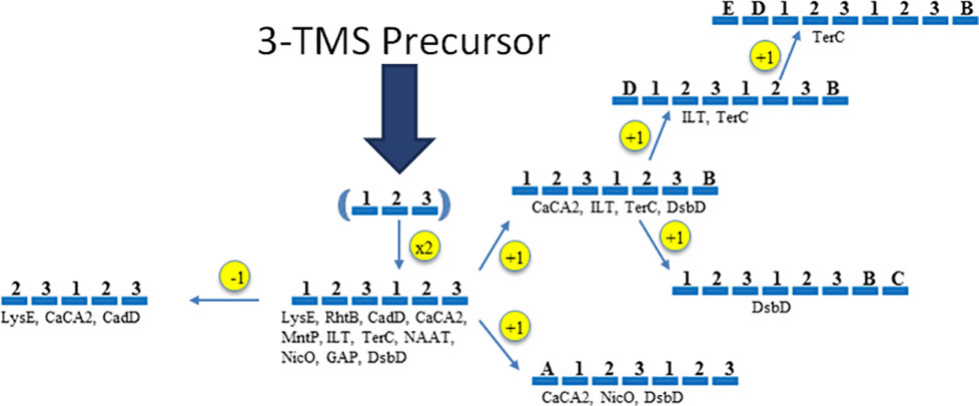
Proposed evolutionary history for the appearance of the eleven recognized families in the LysE superfamily. Protein topologies are indicated with bars representing TMSs and numbers indicating the positions of the TMSs in the proposed TMS primordial protein (in parentheses). Families are indicated by their standard abbreviations while numbers indicate "extra" TMSs outside of their basic 6-TMS unit, resulting from intragenic duplication of the primordial 3TMS precursor. A family abbreviation with a particular topology indicates that at least some members of the family are believed to have this topology.

**Fig 2:**
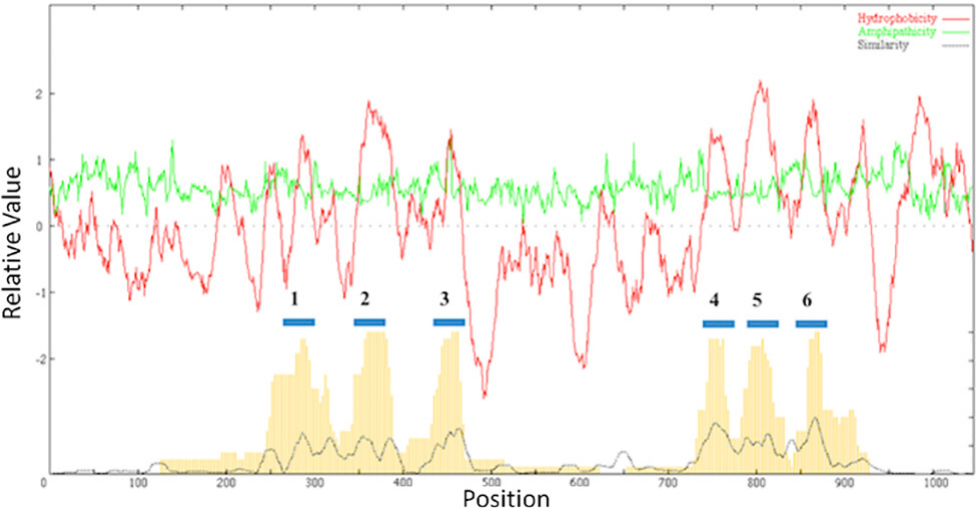
Combined AveHAS plot of proteins in the eleven recognized families in the LysE superfamily. Upper plot: The dark line shows average hydropathy while the light line shows average amphipathicity. Lower plot: The dotted line presents average similarity while the vertical lines indicate average hydropathy, determined by a second method. Numbers above the six bars indicate their TMSs in the basic transport protein unit.

**Fig 3:**
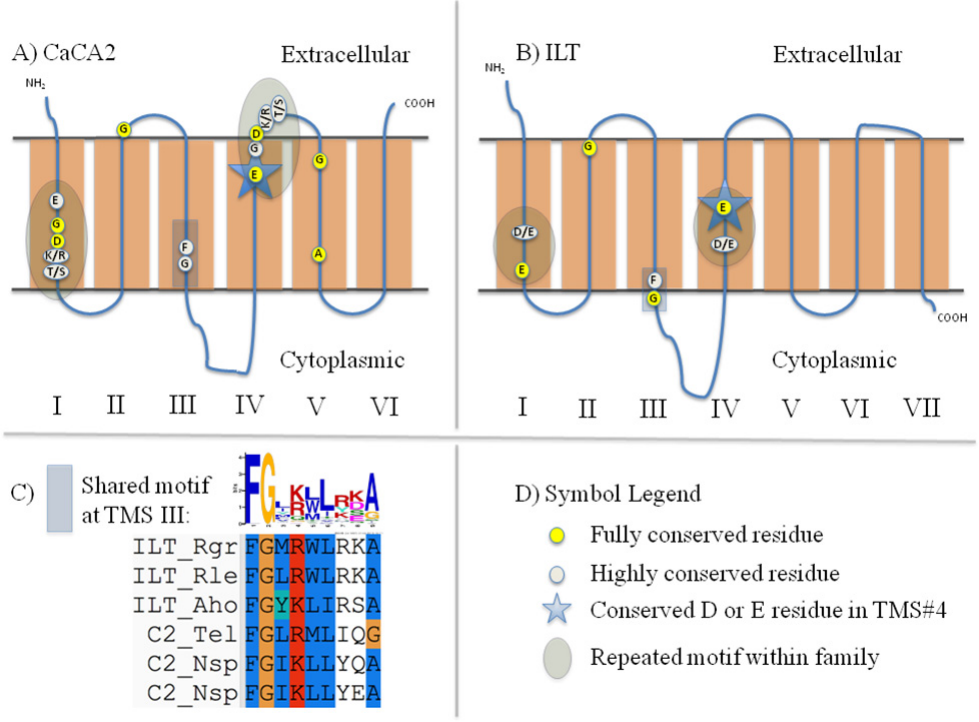
Schematic diagrams depicting motifs and highly conserved residues within and between the CaCA2 (C2) and ILT families. Highly conserved residues were identified using alignments generated from Mafft. In Part C, the MEME/MAST Suite was used to generate the graphical logo, and the alignment was presented using the ClustalX2 user interface with the associated Mafft multiple sequence alignment (MSA). A) Schematic diagram of CaCA2 proteins. B) Schematic diagram of ILT proteins. C) Graphical representation of the shared motifs depicted in Part A and Part B. D) Symbol Legend.

**Fig 4:**
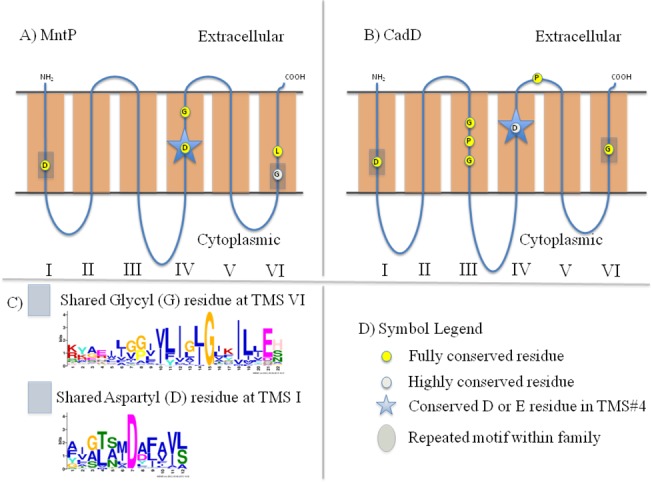
Schematic diagrams depicting motifs and highly conserved residues within and between the MntP (MP) and CadD (CD) families. A) Schematic diagram of MntP proteins. B) Schematic diagram of CadD proteins. C) Graphical representation of the shared motifs depicted in Part A and Part B. D) Symbol Legend.

**Table 4:** Protein families with Demonstrated Internal Repeat Elements. UniProt accession numbers are provided in Column 2. The TMSs aligned refers to the positions of the TMSs from the N-terminus. For 6-TMS proteins, we find the 3-TMS internal repeat elements occur as two tandem 3-TMS elements for all families examined. For 7-TMS proteins, we find the 3-TMS internal repeat elements in the first 6 TMSs, suggesting these 7-TMS proteins have a 3+3+1 topology. The GSAT alignments generated using 20,000 shuffles for these comparisons are presented in Column 6.

Family	Protein Accession #	# of TMSs in Protein	TMSs aligned	Score (S.D.)	Figure #
CaCA2	Q2JWH3	6	1–3 and 4–6	13.5	S11A
	I7M883	6	1–3 and 4–6	11.3	S11B
	K4DX00	6	1–3 and 4–6	5.7	S11C
ILT	Q8YX33	7	1–3 and 4–6	10.7	S12A
	K9Q6B8	7	1–3 and 4–6	9.4	S12B
	J2KV33	7	1–3 and 4–6	8.0	S12C
MntP	A8SU47	6	1–3 and 4–6	8.1	S13A
	R9SLI6	6	1–3 and 4–6	7.4	S13B
	C6JCY1	6	1–3 and 4–6	6.9	S13C
TerC	A4IKQ1	7	1–3 and 4–6	9.4	S14A
	G8M4S7	7	1–3 and 4–6	9.1	S14B
	R9LI44	7	1–3 and 4–6	7.8	S14C

**Table 5:** Protein families with Identified Motifs using MEME/MAST. Protein families demonstrating shared, conserved residues are shown below. HMMTOP was used to predict the TMS location for each motif. Schematic diagrams showing the motif locations and other highly conserved residues are found in Fig 3A–3G.

Families	Predicted TMS region	# Proteins displaying motif/# Total Proteins	Motif
CaCA2 & ILT	#3 of both	80/80 (40 ILT, 40 CaCA2)	FGX(K/R)XL
CadD & MntP	#4 of both	170/170 (85 CadD, 85 MntP)	Fully conserved D
CadD & MntP	#6 of both	170/170 (85 CadD, 85 MntP)	Conserved G
CadD & MntP	#1 of both	170/170 (85 CadD, 85 MntP)	Fully conserved D
TerC & LysE	#3 of both	248/248 (124 LysE, 124 TerC)	GXXXL
TerC & RhtB	#3 of both	176/176 (88 TerC, 88 RhtB)	GXXYL

### Controls

#### The Mitochondrial Carrier Family and the LysE superfamily

Members of the MC family have been shown to transport keto acids, amino acids, nucleotides, inorganic ions and co-factors across the membranes of mitochondria and other eukaryotic organelles [36,37]. Crystal structures for MC proteins have been elucidated, and these 6-TMS proteins were shown to have arisen via a 2-TMS triplication [28,38,39]. Members of the LysE superfamily, however, are predicted to have arisen via a 3-TMS duplication. Because of the differences in these two evolutionary pathways, MC proteins have been selected as a negative control to establish the highest possible comparison score that can be obtained by chance using non-homologous members of two unrelated superfamilies (Tables 2 and 3).

The best comparison score between 3-TMS segments of the MC and LysE superfamily members was 10.5 S.D. This score was obtained between proteins of the MC family and the CaCA2 family. The average score for the five best comparisons between LysE superfamily members and the MC family was 9.8 S.D. Although at least 3 TMSs of members of these two superfamilies were included in each alignment, the TMS alignments were poor (S16J and S16K Fig). TMS overlap in the alignments is present in Table 2. In contrast, the average score for all of the best comparisons for the eleven LysE superfamily families with each other (Table 3) is 13.5 S.D, and corresponding TMSs were strongly aligned. Based on these results, we suggest that three conditions are sufficient to provide strong evidence for homology: (1) a standard comparison score of at least 13.0 S.D.; (2) proper alignment of at least 3 TMSs and (3) a unified evolutionary pathway for all superfamily members (Fig 1). These criteria were satisfied for all eleven members of the LysE superfamily.

### Establishing Homology

#### The L-Lysine and L-Arginine Exporters (LysE; TC# 2.A.75); Homoserine/Threonine Resistance Proteins (RhtB; TC# 2.A.76); Cadmium Ion Resistance Proteins (CadD; TC# 2.A.77)

Previously published studies have shown that LysE, RhtB and CadD are distantly related [1]. We support this conclusion with additional statistical analyses (S2A–S2C Fig). Six TMSs are predicted for each of the homologues analyzed in this section. The top pair-wise analysis of RhtB and LysE homologues, Pst1 (H3RH39) v Bth1 (Q2SUV5)**,** demonstrated a comparison score of 20.1 S.D. The first five of six TMSs for each of these two proteins aligned (S2A Fig). A score of 32.5 S.D. resulted when comparing the full sequences of Pst1 with the LysE protein, TC# 2.A.75.1.1 (P94633). In addition, a score of 52.0 S.D. was obtained when comparing the full sequences of Bth1 with RhtB protein, TC# 2.A.76.1.5 (P76249). These comparison scores satisfy our statistical standards for homology, and thus, we apply the superfamily principle to confirm that these two families are related (Table 3).

TMSs 2–4 of Oki1 (G9WHF3), a CadD homologue, aligned with TMSs 2–4 of the RhtB homologue Hal1 (G9Y0F1) with a comparison score of 11.9 S.D (S2B Fig). A comparison score of 12.1 S.D. (S2C Fig) resulted from alignment of TMSs 2–5 of the CadD homologue Cth1 (K9TWQ5) with TMSs 2–5 of the LysE homologue Asp2 (K0HW07). The relationships between CadD proteins and LysE and RhtB proteins are not apparent based on our statistical standards for sequence similarity. Additional evidence will be discussed to expand upon these relationships and establish homology.

#### Ca^2+^/H^+^ antiporters-2 (CaCA2; TC# 2.A.106)

Members of the family of Ca^2+^/H^+^ antiporters, CaCA2, contain around 200–350 amino acyl residues, with 6 TMSs, typically with a 3+3 TMS arrangement, and are found in all three domains of life. Functionally characterized members of this family play roles in Ca^2+^ export driven by coupled H^+^ influx [32,33]. These proteins display significant sequence similarity with 6-TMS CadD, LysE, and RhtB homologues (S3A–S3C Fig).

TMSs 1–3 of the CaCA2 homologue Mpu4 (C1MR94) and the LysE homologue Cac2 (E0MXD6) were compared, yielding a score of 13.5 S.D. A score of 31.7 S.D. occurred when comparing the full sequences of Mpu4 and the CaCA2 protein, TC# 2.A.106.1.1 (P52876). In addition, a score of 63.0 S.D. resulted when comparing the full sequences of Cac2 with LysE, TC# 2.A.75.1.1 (P94633). Therefore these two families are homologous.

Particularly strong evidence was obtained from a comparison between CaCA2 and CadD proteins. TMSs 1–3 of the cadmium resistance protein Efa1 (L2SR21) aligned with TMSs 1–3 of the CaCA2 homologue Ptr2 (B7FUM2) to give a comparison score of 14.2 S.D (S3A Fig). A score of 57.2 S.D. resulted when comparing the full sequence of Ptr2 with that of the CaCA2 protein, TC# 2.A.106.1.1 (P52876). In addition, a comparison of the full-length sequences of Efa1 and CadD TC# 2.A.77.1.1 (O05469) yielded a score of 50.7 S.D. Because the CaCA2 family is homologous to CadD, LysE and RhtB family members, we conclude that CaCA2 and CadD are members of the LysE superfamily. Comparison scores between the CaCA2 family and the MntP, ILT, TerC and DsbD families were also 13.0 S.D or greater (Tables 2 and 3).

#### Mn^2+^ exporters (MntP; TC# 2.A.107)

Similar to previously established members of the LysE superfamily, members of the MntP family are characterized by a size of around 200 amino acyl residues with 6 TMSs in a 3+3 TMS arrangement. They are exclusively found in bacteria and archaea. A member of this family, YebN, is known to export manganese ions [34,40]. YebN has been suggested to share significant sequence similarity with members of the LysE family efflux pumps [34]. 6-TMS MntP proteins share sufficient sequence similarity with RhtB, CadD and CaCA2 family members to establish homology (Tables 2 and 3, S4A–S4C Fig).

A comparison between the MntP homologue Dvu1 (Q727E5) and the cadmium resistance protein Hku1 (H3NKZ1) displayed an alignment of TMSs 3–6 in both proteins with a score of 15.7 S.D (S4B Fig). A score of 34.3 S.D. was obtained when comparing the full sequences of Dvu1 with MntP protein, TC# 2.A.107.1.2 (O27840), and a score of 48.0 S.D. resulted when comparing the full sequences of Hku1 with the CadD protein, TC# 2.A.77.1.1 (O05469). Although significant scores were not observed with LysE homologues, relationships between RhtB, CadD and CaCA2 families have been established, providing sufficient evidence for the inclusion of MntP as a member of the LysE superfamily. Comparison scores between MntP and TerC, NAAT and DsbD family members were also 13.0 S.D or greater (Tables 2 and 3).

#### Iron/Lead Transporters (ILT; TC# 2.A.108)

ILT family members are heavy metal ion transporters specific for iron and/or lead ions. Topological analyses confirmed that most members of the ILT family have 7 conserved TMSs arranged in a 3+3+1 arrangement [31]. ILT protein sizes vary substantially due to the inclusion of large hydrophilic domains near the N-termini in many of these proteins. A majority of family members are found in bacteria and archaea, but are also found in eukaryotes such as fungi. ILT proteins demonstrate significant sequence similarity with proteins of CadD, RhtB and CaCA2 families (S5A–S5C Fig).

The 6-TMS cadmium resistance homologue Lbr1 (C2D135) and the 8-TMS ILT homologue Sma2 (G5JVH6) were compared. All of the six TMSs in Lbr1 aligned with TMSs 2–7 of Sma2 with a comparison score of 13.5 S.D (S5A Fig). Investigating further with HMMTOP and a WHAT hydropathy plot, we observed that the 8-TMS Sma2 contains the core 3+3+1 arrangement near its C-terminus with a lone TMS at the N-terminus. From these depictions, we note that the 6-TMS Lbr1 protein aligns within the 3+3 region of the 8-TMS Sma2 protein. A score of 41.0 S.D. was obtained when comparing the full sequences of Sma2 with ILT protein, TC# 2.A.108.2.4 (Q5HSD5). In addition, comparing the full length sequences of Lbr1 and CadD TC# 2.A.77.1.1 (O05469), yielded a score of 43.1 S.D., establishing homology between these two families. Additional studies comparing TMSs 1–3 of the 6-TMS RhtB homologue Aau1 (A1RAR9) and TMSs 2–4 of the ILT homologue Eli1 (Q2NBF8) demonstrated a 3-TMS alignment with a score of 13.7 S.D (S5B Fig). Eli1 is predicted to have 7 TMSs, but HMMTOP and WHAT did not recognize a strongly hydrophobic region between predicted TMS#1 and TMS#2 as a transmembrane segment, thus suggesting that this protein has 8 TMSs. Finally, we compared TMSs 1–3 of the ILT homologue Sso1 (Q97V64) with TMSs 1–3 of the CaCA2 homologue Aan1 (F0Y333). This comparison yielded a score of 15.3 S.D (S5C Fig). A score of 67.2 S.D. resulted when comparing the full sequences of Sso1 and ILT protein, TC# 2.A.108.3.3 (Q4J7V8). In addition, a score of 52.7 S.D. was obtained when comparing the full sequences of Aan1 and CaCA2 protein, TC# 2.A.106.1.1 (P52876). With this statistical evidence, we conclude that ILT is an additional member to the LysE superfamily. A comparison between ILT and TerC proteins also yielded high comparison scores (Tables 1 and 2).

#### Tellurium Ion Resistance Proteins (TerC; TC# 2.A.109)

Members of the TerC family are believed to function in tellurium ion resistance [41]. These proteins share a 7-TMS core with a 3+3+1 TMS arrangement and are typically found in bacteria and archaea, but are also found in eukaryotic organisms [42]. Sizes for these proteins range from 180 to 350 with as many as 9 TMSs. Coinciding with the proposed evolutionary pathway (Fig 1), no triplication could be demonstrated for these 9-TMS proteins. TerC members show significant sequence similarities with homologues from a large number of the different families (S6A–S6F Fig).

Of the TerC comparisons, the highest score was observed between TerC and CaCA2 family members (S6F Fig). TMSs 1–3 of the 7-TMS TerC protein Lga1 (D7V5X7) and TMSs 1–3 of the 6-TMS CaCA2 protein Ptr2 (B7FUM2) aligned and yielded a score of 16.2 S.D. A score of 62.9 S.D. resulted when comparing the full sequences of Lga1 and TerC protein, TC# 2.A.109.1.3 (B5UIP4). Furthermore, a score of 57.2 S.D. was obtained when comparing the full sequences of Ptr2 and CaCA2 protein, TC# 2.A.106.1.1 (P52876). In addition, TerC proteins yielded significant comparison scores with 8 of the 10 other families shown in Table 2. These relationships provide further evidence for the inclusion of the TerC families in the LysE superfamily.

#### Neutral Amino Acid Transporter Family (NAAT; TC# 2.A.95)

NAAT family proteins are exclusively found in bacteria and archaea. The majority of these proteins have sizes between 190–280 amino acids with 6 predicted TMSs in a 3+3 TMS arrangement. The best characterized member of the NAAT family, SnatA, is involved in the uptake of neutral amino acids, glycine and alanine [35]. Several homologues have been annotated as multiple drug resistance proteins. However, a recent study provided evidence that disagrees with this functional assignment [43]. Significant comparison scores with NAAT proteins were seen between LysE, RhtB, CadD, MntP, and TerC family proteins (S7A–S7E Fig).

The best example of homology is seen with the comparison of TMSs 1–5 of the RhtB homologue Pag1 (L7BNM7) and the NAAT homologue Cba1 (H1S8A2), which yielded a score of 15.0 S.D (S7B Fig). When comparing the full length sequences of Cba1 and NAAT protein, TC# 2.A.95.1.4 (Q8J305), a score of 39.2 S.D. was obtained. Comparing the full sequences of Pag1 and RhtB protein, TC# 2.A.76.1.2 (P0AG38), gave a score of 95.4 S.D., thus establishing homology between these two families. In addition to the relationships with members of the LysE, RhtB, CadD, MntP and TerC families, relationships with NicO and DsbD family members were apparent, providing sufficient evidence for the inclusion of NAAT as a member of the LysE superfamily.

#### Nickel/Cobalt Transporter Family (NicO; TC# 2.A.113)

RcnA of the NicO family has been shown to play a role in Ni^2+^ and Co^2+^ efflux from *E*. *coli* [44]. Members of this family are found across all three domains of life. Here we report significant comparison scores with RhtB, CadD, TerC and NAAT family proteins (S8A–S8D Fig).

Comparing TMSs 1–6 of the CadD homologue Acy3 (K9ZC80) with the NicO homologue Gar1 (K6XDF4) yielded a score of 15.1 S.D (S8B Fig). In this comparison, every TMS aligned correspondingly in the two sequences. A score of 22.4 S.D. resulted when the full sequence of Gar1 was compared with that of the NicO protein, TC# 2.A.113.1.9 (F8C138), and a score of 24.8 S.D. was obtained when comparing the full sequence of Acy3 with an established CadD protein, TC# 2.A.77.1.2 (Q45153). These results provided strong evidence that NicO is homologous to the previously discussed families and support further expansion of the LysE superfamily. A significant comparison score between NicO and DsbD was also noted.

#### Peptidoglycolipid Addressing Protein Family (GAP; TC# 2.A.116)

GAP family proteins are typically found in bacteria and are prominent in members of the mycobacterial genus. The majority of these proteins have sizes between 180–290 amino acids with 6 predicted TMSs in a 3+3 TMS orientation. The best characterized member of the GAP family, Q3L890 of *Mycobacterium smegmatis*, has been reported to play a role in biogenesis of the mycobacterial cell envelope via the transport of peptidoglycolipids [45]. The mechanism by which transport occurs is largely unknown. However, statistical relationships between GAP proteins and members of RhtB and DsbD families were determined (S9A and S10E Figs).

A comparison between sequences containing TMSs 1–5 of the RhtB homologue Hgr1 (F3KVR3) and the GAP homologue Ssp3 (NCBI: WP_019358971.1) yielded a comparison score of 14.5 S.D., demonstrating homology between the two families. A score of 16.6 S.D. was found when comparing the full length sequence of Ssp3 with that of the GAP protein, TC# 2.A.116.1.7 (K6W6C5), and a score of 45.2 S.D. resulted when comparing the full sequences of Hgr1 and RhtB protein, TC# 2.A.76.1.5 (P76249). This relationship with the LysE superfamily allows predictions and guided exploration into the mechanistic features of GAP proteins.

#### Disulfide Bond Oxidoreductase D Family (DsbD; TC# 5.A.1)

DsbD is a large family of transmembrane electron carriers that is represented in all domains of life. Several functional roles have been reported for these proteins: (i) thiol-disulfide exchange, (ii) cytochrome c biogenesis, (iii) methylamine utilization, (iv) mercury resistance, (v) copper resistance, and (vi) various additional reductase functions. Previous studies demonstrated that DsbD arose from an intragenic gene duplication of 3-TMS elements [29]. Homology was established between DsbD and the RhtB, CaCA2, MntP, NAAT and GAP family proteins (S10A–S10E Fig).

In exploring these relationships, 6 TMSs of the NAAT homologue Pfu1 (Q8U2T5) were found to align with 6 TMSs of the DsbD homologue Dto1 (K0NNX9), yielding a score of 15.3 S.D (S10D Fig). A score of 41.9 S.D. resulted when comparing the full length sequences of Dto1 with DsbD protein, TC# 5.A.1.2.1 (P45706), and comparing the full length sequences of Pfu1 and NAAT protein, TC# 2.A.95.1.4 (Q8J305) yielded a score of 82.4 S.D. These alignments establish membership within the LysE superfamily.

### Topological Analyses

Using ClustalX, Mafft and Probcons, we created multiple alignments for homologues within each family included in our study [11]. The alignments generated with each program showed a high degree of agreement. Because Mafft alignments were able to produce comparable residue patterns to ClustalX without excessive expansion of the residue position axis (S11 Fig), Mafft alignments were selected to represent the data. With these Mafft alignments, we generated AveHAS plots to examine the relative average hydropathy, amphipathicity and similarity plots for the homologues (S11 Fig). Additionally, AveHAS plots were generated from multiple alignments of homologues for all families with established statistical relationships (Fig 2).

Examining the plots for S11A–S11K Fig, we observe that the homologues for the LysE, RhtB, CadD, CaCA2, MntP, NAAT, NicO, GAP and DsbD families are most similar in regions corresponding to predicted TMS#1 and TMS#6. Furthermore, these figures show that the largest hydrophilic region separates TMSs #3 and 4, corresponding to regions that are highly dissimilar. These analyses support a 3+3 topological arrangement for all LysE superfamily proteins. Homologues of TerC and ILT display a 7-TMS core (S11J–S11K Fig) but share the previous characteristics with LysE, RhtB, CadD, CaCA2 and MntP. With respect to the TerC and ILT proteins, we observe a predicted 3+3+1 topological arrangement (Fig 1), but many ILT family homologues have 8 predicted TMSs, where an additional hydrophobic peak occurs at the N-termini. TerC proteins, on the other hand, can vary between 6 to 9 TMSs, and additions may occur either in the C-terminal or N-terminal regions of the sequences.

Finally, we examined a combined AveHAS plot of all eleven families with established statistical relationships. The plot (Fig 2) reveals a core of 6 TMSs among the different families with a large hydrophilic region separating the aligned core TMS#3 and TMS#4. These results further support a 3+3 TMS arrangement for members of the LysE superfamily.

### Identifying Internal Repeats

Previous work on the LysE superfamily suggested that members derived from a 3-TMS internal duplication to result in a 3+3 TMS arrangement [1]. A recent examination of ILT transporters suggested a 3+3+1 arrangement with two 3-TMS repeat elements followed by a single extra TMS [31]. In addition, CaCA2 and DsbD proteins have been suggested to contain 3-TMS repeat elements [29,32]. Using IntraCompare and GSAT, we report evidence for internal 3-TMS repeats in several members of the LysE superfamily (Table 4, S12–S15 Figs). This evidence supports the proposed hypothesis that all of these proteins arose via a common intragenic duplication event.

Strong evidence is seen in the 6-TMS CaCA2 Ssp2 protein (S12 Fig). Comparing the first and second halves of the Ssp2 protein (Q2JWH3), TMSs 1–3 and TMSs 4–6 were found to align. The comparison yielded a score of 13.5 S.D., which is sufficient to establish the existence of two homologous internal repeats. The existence of this internal repeat element confirms previous reports regarding the repeating ExGD(KR)(TS) motif in TMS#1 and TMS#4 of the CaCA2 family [32]. Since we have demonstrated that CaCA2 is a member of the LysE superfamily, the other LysE superfamily proteins are presumed to share the same evolutionary pathway.

### Motif Analyses

**Fig 5:**
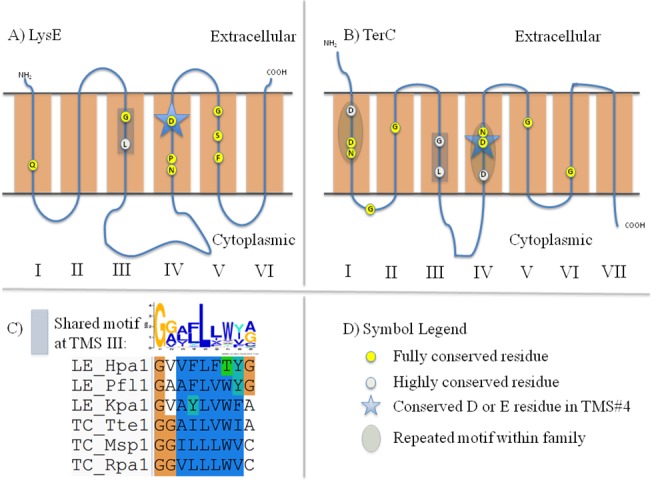
Schematic diagrams depicting motifs and highly conserved residues within and between the LysE (LE) and TerC (TC) families. A) Schematic diagram of LysE proteins. B) Schematic diagram of TerC proteins. C) Graphical representation of the shared motifs depicted in Part A and Part B. D) Symbol Legend.

**Fig 6:**
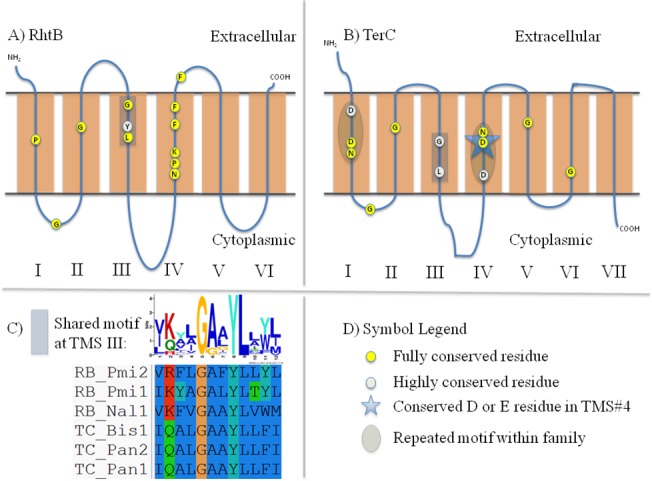
Schematic diagrams depicting motifs and highly conserved residues within and between the RhtB (RB) and TerC (TC) families. A) Schematic diagram of RhtB proteins. B) Schematic diagram of TerC proteins. C) Graphical representation of the shared motifs depicted in Part A and Part B. D) Symbol Legend.

Previous mutation studies on the LysE protein in *Corynebacterium glutamicum* demonstrated the importance of highly conserved residues in the second and fourth hydrophobic segments of the protein [46]. A highly conserved aspartic acid (D) is present in the second hydrophobic segment of LysE, and its negative charge is essential for translocation of L-lysine. In addition, mutations to the fully conserved asparaginyl (N) and prolyl (P) in the fourth hydrophobic segment reduce export function dramatically. The prolyl residue in particular holds importance for three-dimensional structures of the carrier, and any changes in the neighboring asparaginyl residue would introduce steric hindrance. A fully conserved aspartic acid (D) is also present in the fourth hydrophobic segment, and has been proposed to bind the L-lysine substrate. Change of this aspartic acid (D) to a lysyl (K) residue resulted in an inactive protein. In the present study, motifs identified using the MEME/MAST Suite (www.meme.nbcr.net/meme/) for the different families were compared with one another (Figs 3, 4, 5 and 6, Table 5) [16]. Here we report strongly conserved residues within and between families.

#### CaCA2 vs. ILT

80 proteins of CaCA2 and ILT homologues were combined and found to exhibit a shared motif in TMS#3 in these 6-TMS proteins (Fig 3A and 3B, Table 5). Not only do the two motifs align in the MEME/MAST Suite, all tested proteins share many strongly conserved residues. Positions 1–2 of this motif correspond to the second half of TMS#3 that is shared in proteins of the two families. Of the 9 positions, amino acids in positions 1, 3, 5, 6 and 9 consist largely of hydrophobic residues. In positions 1 and 2, both families contain fully conserved phenylalanine (F) and glycine (G) residues, respectively.

At TMS#1 and TMS#4, both families contain two strongly conserved negatively charged amino acyl residues (D/E). Similar to proteins in the CaCA2 and ILT families, conserved negatively charged residues have been found in MntP, CadD and TerC proteins (Figs 3, 4, 5 and 6). With the exception of the CadD proteins, the conserved, negatively charged residues in TMS#1 and TMS#4 within each protein align (S12, S13, S14 and S15 Figs). The D/E residue in these 5 families could have functional significance similar to the D residue in the fourth hydrophobic segment of LysE described previously. However, the biological significance of the conserved, negatively charged residues in TMS#1 is not yet understood. These findings imply an evolutionary relationship between these five families and a closer relationship between CaCA2 and ILT.

#### MntP vs. CadD

Sequences of 85 MntP and 85 CadD proteins, all containing 6 TMSs, were combined into a single file shown to share motifs (Fig 4A and 4B, Table 5). The best shared motif in TMS#4 of MntP and CadD proteins was found in all of 170 selected proteins. Positions 1–13 in this motif correspond to the second half of TMS#4 that is shared in proteins of these two families. A highly conserved aspartic acid (D) is contained in this shared motif. Differing within the TMS#4 motif are positions 5, 8, 12 and 14. Position 5 is a fully conserved serine (S) in MntP homologues, but is a strongly conserved glycine (G) in CadD homologues. Position 8 is a strongly conserved asparagine residue in CadD homologues, but a strongly conserved alanine in MntP homologues. Additionally, position 12 corresponds to a well-conserved tyrosine in CadD proteins, but a fully conserved glycine in MntP proteins. Finally, we note well-conserved polar amino acids in position 14 for MntP homologues, but a conserved proline residue in CadD homologues.

A shared motif corresponding to the entire TMS#6 in 85 MntP and 85 CadD proteins was identified (Fig 4A and 4B, Table 5). A completely conserved glycine was shared at position 15, and strongly conserved acidic residues occurred at position 21. Finally, well-conserved hydrophobic amino acids were present in positions 6, 9, 10, 12, 14, 16, 18, 19 and 20, providing additional support for a close evolutionary relationship between MntP and CadD proteins.

The strongly conserved residues of the two sets of homologues differ at positions 4, 7, 8, 11, 13 and 22. In position 4, negatively charged amino acids are largely conserved only in MntP homologues. Position 11 differs where a completely conserved leucine residue in MntP homologues but either a phenylalanine or a tyrosine in CadD homologues is found. A glycine is well-conserved at position 13 of CadD homologues, but it is weakly conserved in MntP homologues. Position 22 of CadD homologues shows well-conserved polar amino acids (S, N), while this position in MntP homologues contains a conserved histidyl residue. Finally, we note two unique residues at positions 7 and 8: proline and glycine. Conserved proline residues can be found in CadD only (position 8), while two almost fully conserved glycines are present in MntP homologues (positions 7 and 8). These unique differences may provide insight into the divergence of these proteins and possibly correlate with their differing specificities.

#### LysE, RhtB and TerC

More distantly related are the motifs within members of the LysE, RhtB and TerC families. Among these three families, two residues in TMS#3 are shared (Figs 5–6, Table 5). In the middle of TMS#3, all three families show a fully conserved glycine. Additionally, a fully conserved leucine, three residues (one helical turn) away from the glycine, can be found. Strongly conserved hydrophobic residues between the fully conserved glycyl and leucyl residues are present. A tyrosine (Y) is also conserved between 88 RhtB and 88 TerC proteins (GxxYL) but is not observed in LysE proteins (GxxxL).

### Phylogenetic Tree

**Fig 7:**
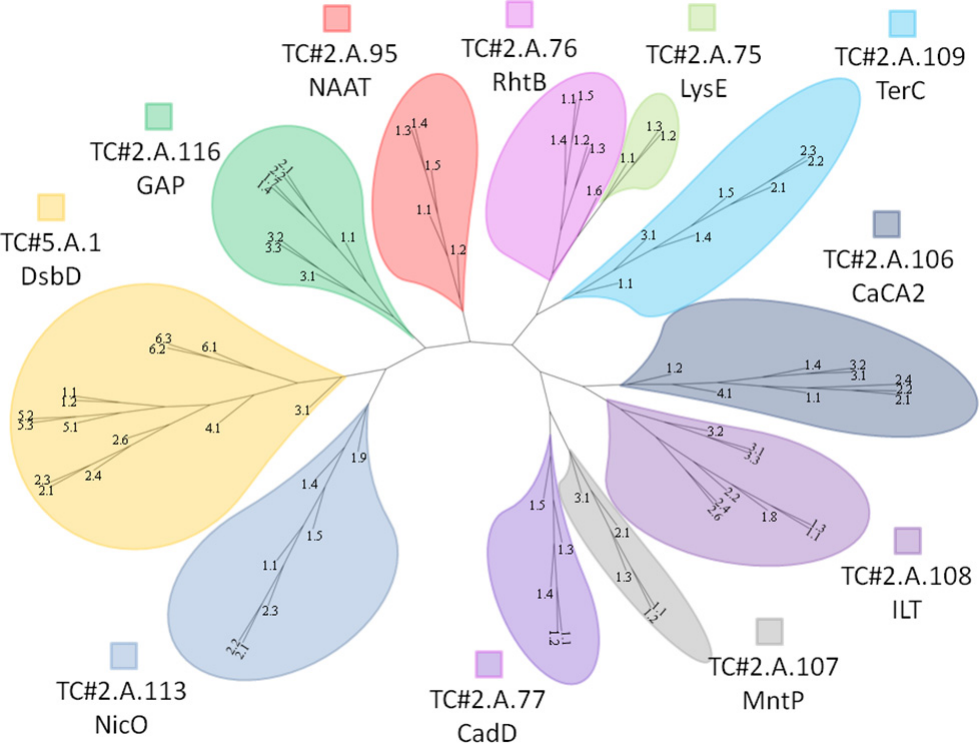
Phylogenetic Tree of the LysE Superfamily. The tree was generated using the SuperFamilyTree program and viewed using FigTree. It depicts the evolutionary relationship between the 11 different families in this study. Clustering indicates closer phylogenetic relationships. The tree is based on tens of thousands of BLAST bit scores generated with the SFT1 program where every protein was compared with every other protein included in the analysis. The SFT2 program was used to integrate all of the information to show the relationships of the eleven families to each other. Associated bootstrap values can be found in S29 Fig. When using BLAST bit score comparisons for determining phylogeny, the bootstrap values become less indicative of the reliability and accuracy of observed clustering patterns for very closely related proteins [19].

Proteins listed in TCDB for each family were used to generate a phylogenetic tree based on tens of thousands of BLAST bit-scores using the SFT1 program (Fig 7) [20]. RhtB, LysE and TerC localize to a single branch. Similarly, CaCA2 clusters with ILT, and CadD clusters with MntP. Based on these branching patterns, members in each of these groupings must be more strongly related to each other than to other families as had been suggested from motif analyses. A tree including all eleven families generated using a Mafft multiple alignment and RAxML with bootstrap values was included for comparison (S17 Fig). The SFT and Mafft trees show remarkable agreement, particularly with respect to family relationships. However, the branches sometimes differ between the two trees (compare Fig 7 with S17 Fig), but all of the proteins cluster with their respective families, with the exception 2.A.109.3.1 (TerC.3.1), 2.A.108.2.6 (ILT.2.6) and 2.A.108.3.2 (ILT.3.2). A significant difference deals with the proteins of the CaCA2 family in the two trees. Based on our previous experience [19,20,21,22,23,24,25,26], this and other differences suggest that the phylogenetic distances between the eleven families are too great to allow the generation of accurate multiple sequence alignments. Trees representing each individual family have been constructed using multiple alignments generated by ClustalX, Mafft and ProbCons (S18–S28 Figs).

## Discussion

Using rigorous statistical criteria, we have expanded the LysE superfamily nearly four-fold. In addition to the LysE, RhtB and CadD families identified previously, this superfamily now includes the following families: NAAT, CaCA2, MntP, ILT, TerC, NicO, GAP and DsbD. Members of each of these families have been characterized and shown to play roles in transport of amino acids and resistance of heavy metal ions, along with cell surface maintenance. Most families include secondary carrier type transporters catalyzing heavy metal or amino acid efflux, but one family catalyzes amino acid uptake, another catalyzes heavy metal ion uptake, and a third catalyzes transmembrane electron transfer. GAP proteins have not been mechanistically characterized, but based on their inclusion in the LysE superfamily, we tentatively propose that GAP proteins operate as secondary carriers, where the energy source for lipid export is the proton motive force.

Through sequence analyses, we were able to recognize a distinct pattern of homology. That is, LysE, RhtB, NAAT, CaCA2, MntP, ILT, TerC, NicO, GAP and DsbD proved to be homologous in 3 or more TMSs. The 3 TMSs that aligned are usually between the first 3 TMSs, the second 3 TMSs or both. This observation fits the predicted evolutionary pathway presented in Fig 1. The presence of 3-TMS internal repeats supports the conclusion that all members of the LysE superfamily arose from a 3-TMS precursor via the same pathway in which the proposed duplication gave rise to 6 TMSs in a 3+3 TMS arrangement. In some TerC and ILT proteins, the topologies differ from the 3+3 TMS arrangement with the addition of one or two TMSs at the C- or N-terminal end, resulting in a 3+3+1, 3+3+2, or 1+3+3 arrangement.

According to the phylogenetic tree, amino acid exporter families RhtB and LysE branch close to each other, as suggested from previous studies [1]. In contrast to these two amino acid exporter families, TerC, which branches near RhtB and LysE in the tree, has been observed to play roles in tellurium ion resistance. MntP and CadD cluster together, and both are involved in divalent metal cation transport. Likewise, divalent cation transporters of the CaCA2 and ILT families branch in close proximity.

This study suggests that members of the LysE Superfamily are involved in ionic homeostasis, protection from excessive cytoplasmic heavy metal/metabolite concentrations, cell envelope assembly and transmembrane electron flow. Many of the family members, however, are still poorly understood from functional and physiological standpoints. In continuing this project, genome context analyses will be conducted on members of each family. This will allow functional predictions, further promoting an understanding of the significance of these proteins. To date, no crystal structures exist for a member of this superfamily, and such studies will be crucial for understanding their mechanistic details. Thus, studies on the LysE superfamily remain in their infancy.

## Supporting Information

S1 Fig Flowchart of the materials and methods. Along with a step-wise description of the methods, the parameters for the programs used in major analyses are summarized.
(TIF)

S2 Fig GSAT comparisons between previously established LysE superfamily members. (A) LysE vs. RhtB. (B) RhtB vs. CadD. (C) LysE vs. CadD.
(PDF)

S3 Fig GSAT comparisons with CaCA2. (A) CadD vs. CaCA2. (B) LysE vs. CaCA2. (C) RhtB vs. CaCA2.
(PDF)

S4 Fig GSAT comparisons with MntP. (A) RhtB vs. MntP. (B) CadD vs. MntP. (C) CaCA2 vs. MntP.
(PDF)

S5 Fig GSAT comparisons with ILT. (A) CadD vs. ILT. (B) RhtB vs. ILT. (C) CaCA2 vs. ILT.
(PDF)

S6 Fig GSAT comparisons with TerC. (A) RhtB vs. TerC. (B) CadD vs. TerC. (C) LysE vs. TerC (D) MntP vs. TerC. (E) ILT vs. TerC. (F) CaCA2 vs. TerC.
(PDF)

S7 Fig GSAT comparisons with NAAT. (A) LysE vs. NAAT. (B) RhtB vs. NAAT. (C) CadD vs. NAAT (D) MntP vs. NAAT. (E) TerC vs. NAAT.
(PDF)

S8 Fig GSAT comparisons with NicO. (A) RhtB vs. NicO. (B) CadD vs. NicO. (C) TerC vs. NicO (D) NAAT vs. NicO.
(PDF)

S9 Fig GSAT comparisons with GAP. (A) RhtB vs. GAP.
(PDF)

S10 Fig GSAT comparisons with DsbD. (A) RhtB vs. DsbD. (B) CaCA2 vs. DsbD. (C) MntP vs. DsbD. (D) NAAT vs. DsbD. (E) GAP vs. DsbD.
(PDF)

S11 Fig AveHAS plots of each family based on multiple alignments generated using three different programs. (A) LysE. (B) RhtB. (C) CadD. (D) CaCA2. (E) MntP. (F) NAAT. (G) NicO. (H) GAP. (I) DsbD. (J) ILT. (K) TerC.
(PDF)

S12 Fig Identification of internal repeats in the CaCA2 family. GSAT comparisons between TMS#1–3 and TMS#4–6 for three CaCA2 homologues with assigned UniProt accession numbers. (A) Q2JWH3. (B) I7M883. (C) K4DX00.
(PDF)

S13 Fig Identification of internal repeats in the ILT family. GSAT comparisons between TMS#1–3 and TMS#4–6 for three ILT homologues with assigned UniProt accession numbers. (A) Q8YX33. (B) K9Q6B8. (C) J2KV33.
(PDF)

S14 Fig Identification of internal repeats in the MntP family. GSAT comparisons between TMS#1–3 and TMS#4–6 for three MntP homologues with assigned UniProt accession numbers. (A) A8SU47. (B) R9SLI6. (C) C6JCY1.
(PDF)

S15 Fig Identification of internal repeats in the TerC family. GSAT comparisons between TMS#1–3 and TMS#4–6 for three TerC homologues with assigned UniProt accession numbers. (A) A4IKQ1. (B) G8M4S7. (C) R9LI44.
(PDF)

S16 Fig GSAT comparisons with MC, the negative control. (A) LysE. (B) RhtB. (C) CadD. (D) CaCA2. (E) MntP. (F) ILT. (G) TerC. (H) NAAT. (I) NicO. (J) GAP. (K) DsbD.
(PDF)

S17 Fig RAxML Phylogenetic Tree of the LysE Superfamily based on a multiple alignment generated with Mafft. The Mafft-homologs function was set to retrieve 200 homologs at a threshold E-value of 1e^-20^ by BLAST (Using UniProt) for each query sequence to improve the accuracy of aligning a small number of distantly related sequences. The bootstrap values are shown in blue text and located near each node.
(TIF)

S18 Fig Phylogenetic Trees of the LysE Family based on multiple alignments generated with (A) ClustalX, (B) Mafft, (C) ProbCons. (PDF)

S19 Fig Phylogenetic Trees of the RhtB Family based on multiple alignments generated with (A) ClustalX, (B) Mafft, (C) ProbCons. (PDF)

S20 Fig Phylogenetic Trees of the CadD Family based on multiple alignments generated with (A) ClustalX, (B) Mafft, (C) ProbCons. (PDF)

S21 Fig Phylogenetic Trees of the CaCA2 Family based on multiple alignments generated with (A) ClustalX, (B) Mafft, (C) ProbCons. (PDF)

S22 Fig Phylogenetic Trees of the MntP Family based on multiple alignments generated with (A) ClustalX, (B) Mafft, (C) ProbCons. (PDF)

S23 Fig Phylogenetic Trees of the ILT Family based on multiple alignments generated with (A) ClustalX, (B) Mafft, (C) ProbCons. (PDF)

S24 Fig Phylogenetic Trees of the TerC Family based on multiple alignments generated with (A) ClustalX, (B) Mafft, (C) ProbCons. (PDF)

S25 Fig Phylogenetic Trees of the NAAT Family based on multiple alignments generated with (A) ClustalX, (B) Mafft, (C) ProbCons. (PDF)

S26 Fig Phylogenetic Trees of the NicO Family based on multiple alignments generated with (A) ClustalX, (B) Mafft, (C) ProbCons. (PDF)

S27 Fig Phylogenetic Trees of the GAP Family based on multiple alignments generated with (A) ClustalX, (B) Mafft, (C) ProbCons. (PDF)

S28 Fig Phylogenetic Trees of the DsbD Family based on multiple alignments generated with (A) ClustalX, (B) Mafft, (C) ProbCons. (PDF)

S29 Fig Phylogenetic Tree of the LysE Superfamily with bootstrap values. The tree was generated using the SuperFamilyTree program and viewed using FigTree. It depicts the evolutionary relationship between the 11 different families in this study. Clustering indicates closer phylogenetic relationships. The tree is based on tens of thousands of BLAST bit scores generated with the SFT1 program where every protein was compared with every other protein included in the analysis. The SFT2 program was used to integrate all of the information to show the relationships of the eleven families to each other. Bootstrap values have been added in blue text and located near each node.
(TIF)

S1 Supporting Information FASTA Files for each family. The corresponding zip file contains the FASTA files generated using Protocol1, for comparisons with Protocol2.
(ZIP)

S2 Supporting Information Multiple Sequence Alignments. The corresponding zip file contains the multiple sequence alignment (MSE) outputs generated using ClustalX, Mafft, and ProbCons. These MSEs have been used to generate S17–S28 Figs.
(ZIP)

S3 Supporting Information Newick and SFT FASTA files. The corresponding zip file contains the 100 trees generated from SFT, the consensus tree, the FASTA sequences used to generated the trees, and the newick file for the best tree generated from RAxML analyses (described in S17 Fig).
(ZIP)

S4 Supporting Information MEME Input Sequences for Figs 3–6. The corresponding zip file contains the FASTA files used to conduct MEME Suite analyses shown in Figs 3–6 and described in Table 5.
(ZIP)

S5 Supporting Information S2–S10 Figs Combined PDF. The corresponding PDF file contains the S2–S10 Figs described previously.
(PDF)

S6 Supporting Information S12–S15 Figs Combined PDF. The corresponding PDF file contains the S12–S15 Figs described previously.
(PDF)

S7 Supporting Information S18–S28 Figs Combined PDF. The corresponding PDF file contains the S18–S28 Figs described previously.
(PDF)
