# Supplementary figures and images for: The LysE Superfamily of Transport Proteins Involved in Cell Physiology and Pathogenesis
Source: PLoS One. 2015 Oct 16;10(10):e0137184. doi: 10.1371/journal.pone.0137184 (PMC4608589; doi:10.1371/journal.pone.0137184)

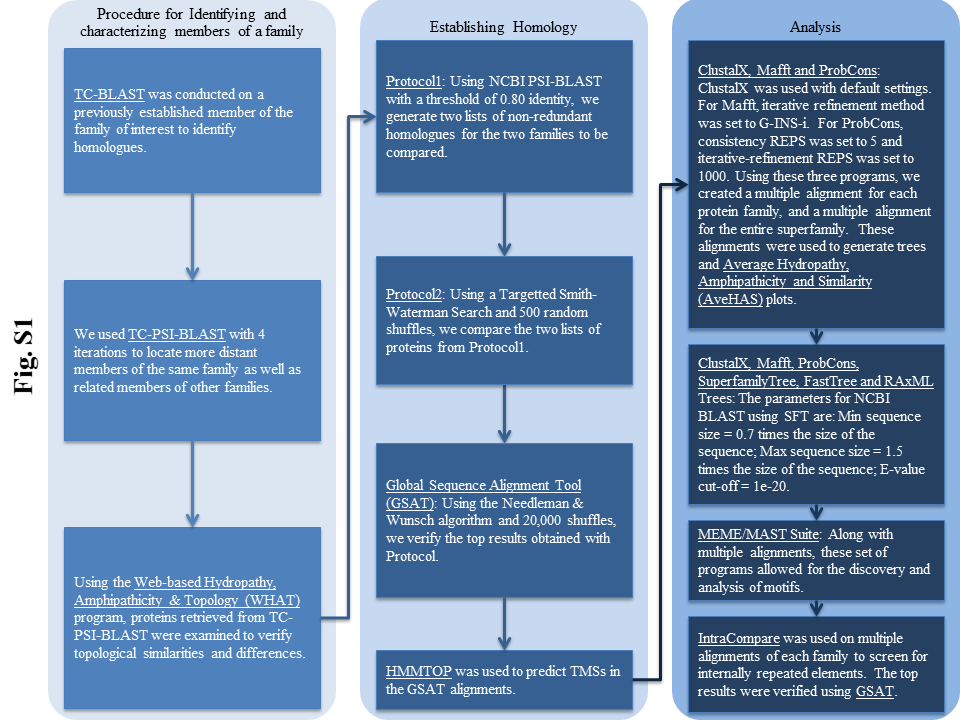

Supplement: S1 Fig — Along with a step-wise description of the methods, the parameters for the programs used in major analyses are summarized. (TIF) [file pone.0137184.s001.tif]

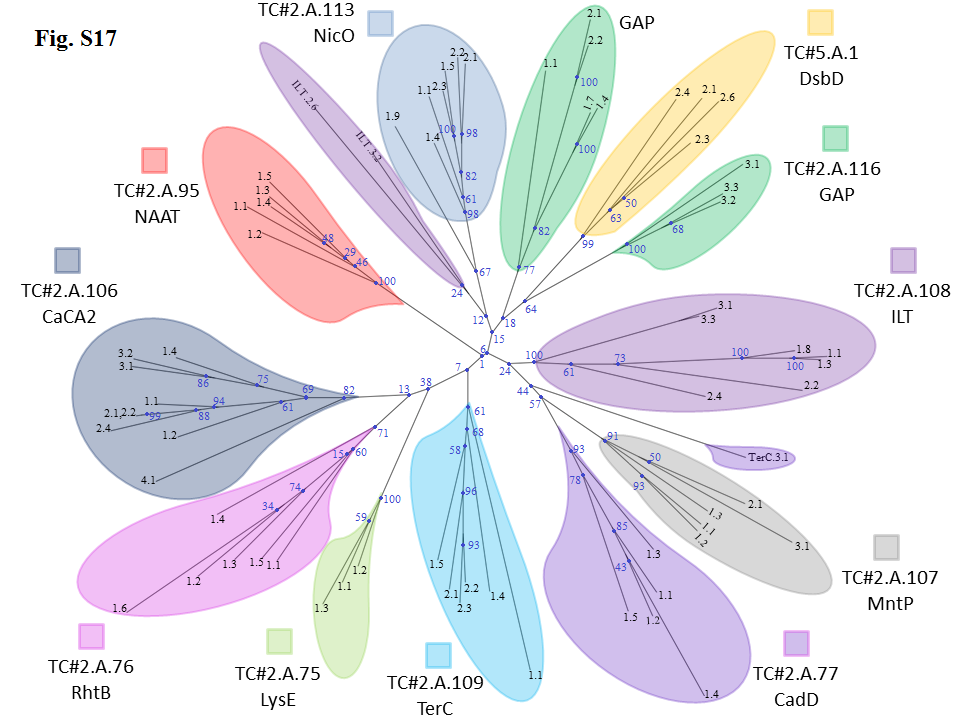

Supplement: S17 Fig — The Mafft-homologs function was set to retrieve 200 homologs at a threshold E-value of 1e-20 by BLAST (Using UniProt) for each query sequence to improve the accuracy of aligning a small number of distantly related sequences. The bootstrap values are shown in blue text and located near each node. (TIF) [file pone.0137184.s017.tif]

Fig. 18A

LysE - Clustal

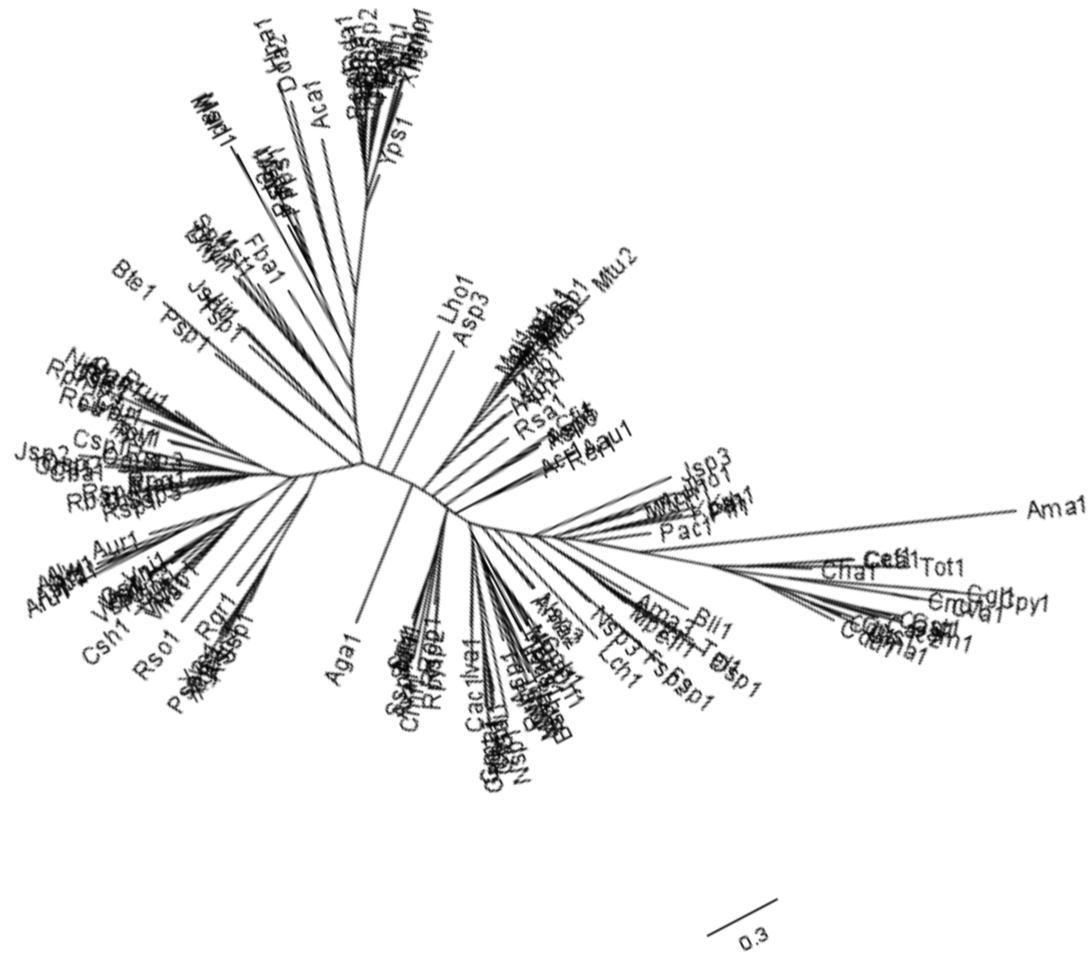

**Fig. 18B**

## LysE - Mafft

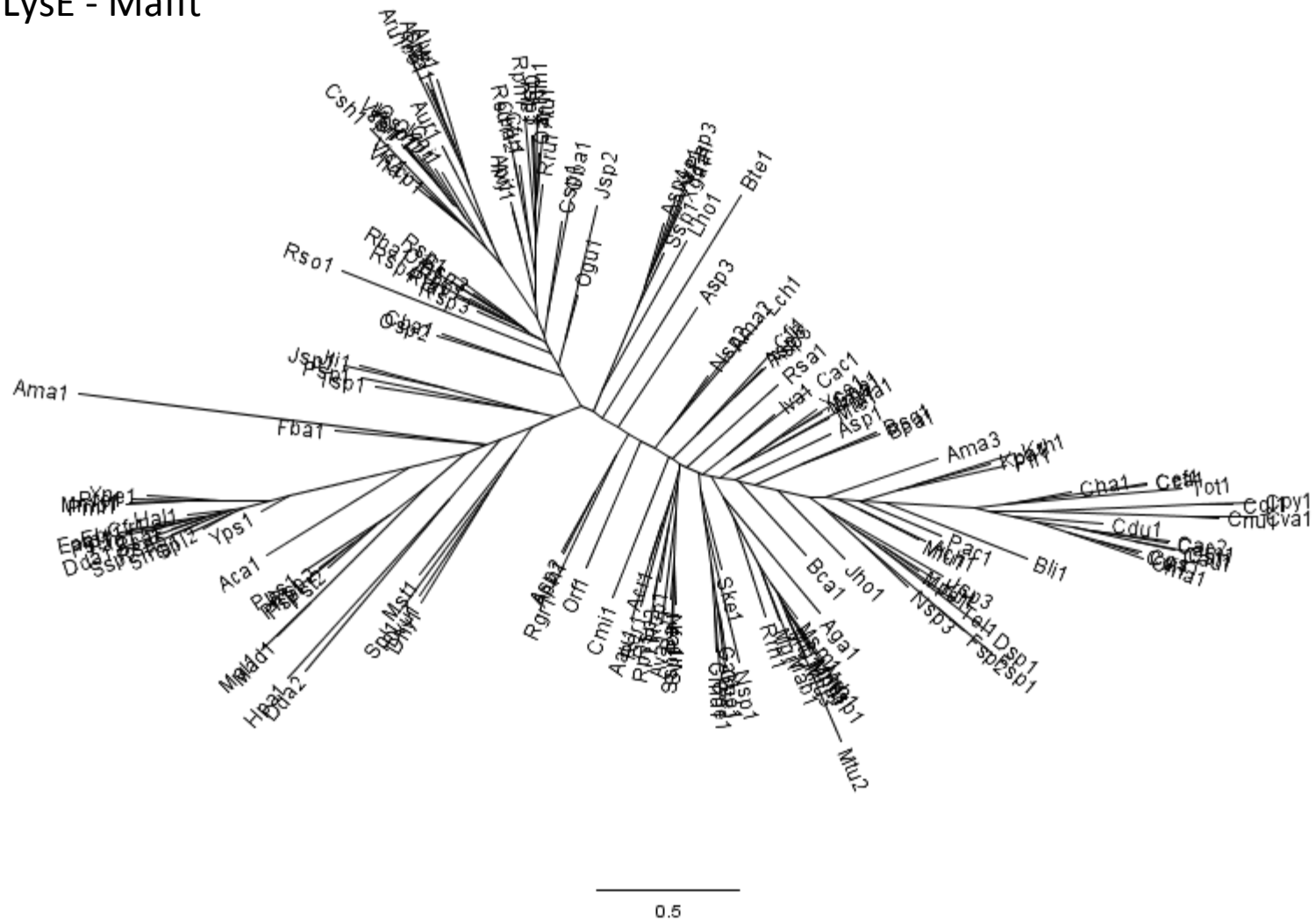

Fig. 18C

LysE - ProbCons

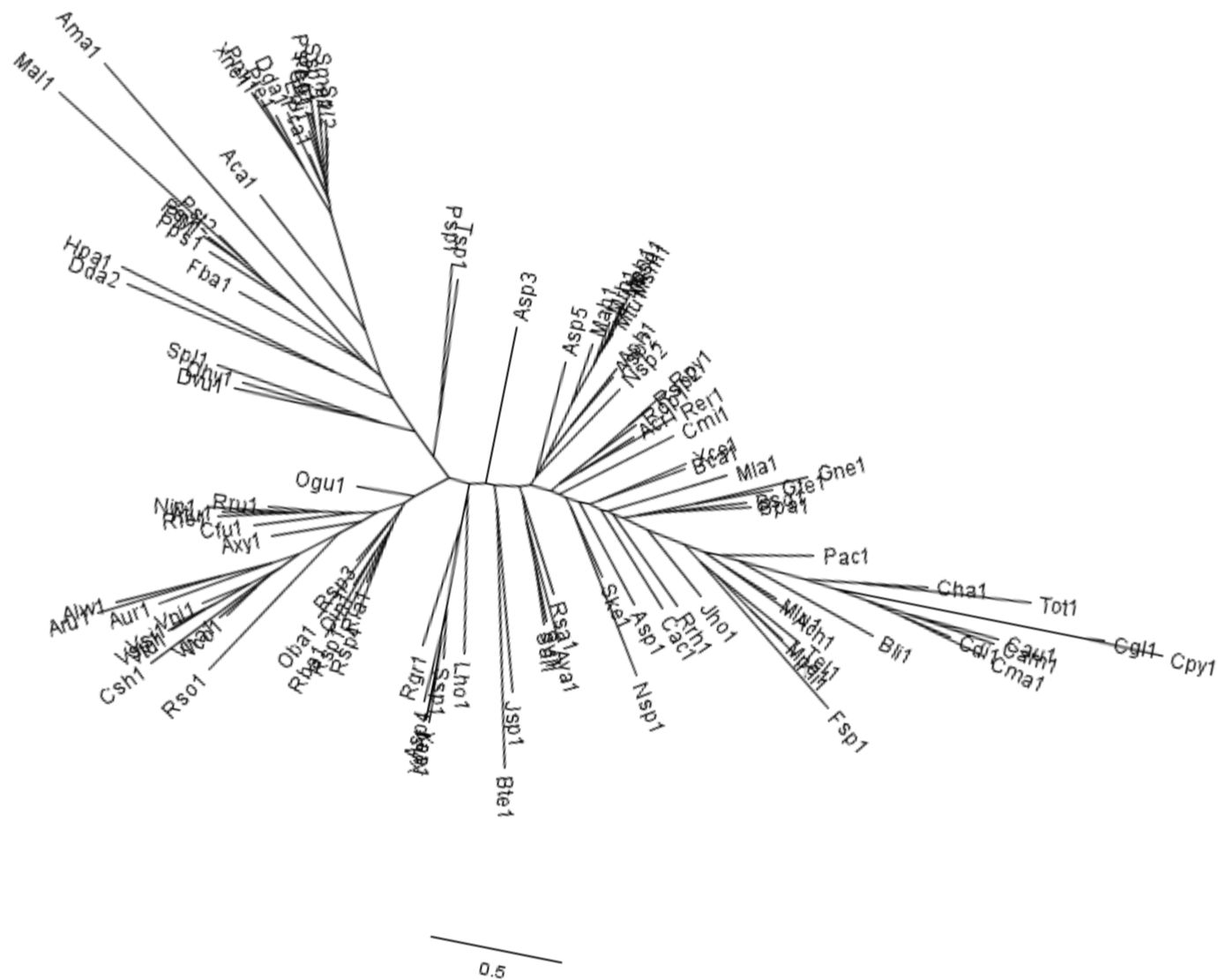

Supplement: S18 Fig — (PDF) [file pone.0137184.s018.pdf]

**Fig. 19A**

RhtB - Clustal

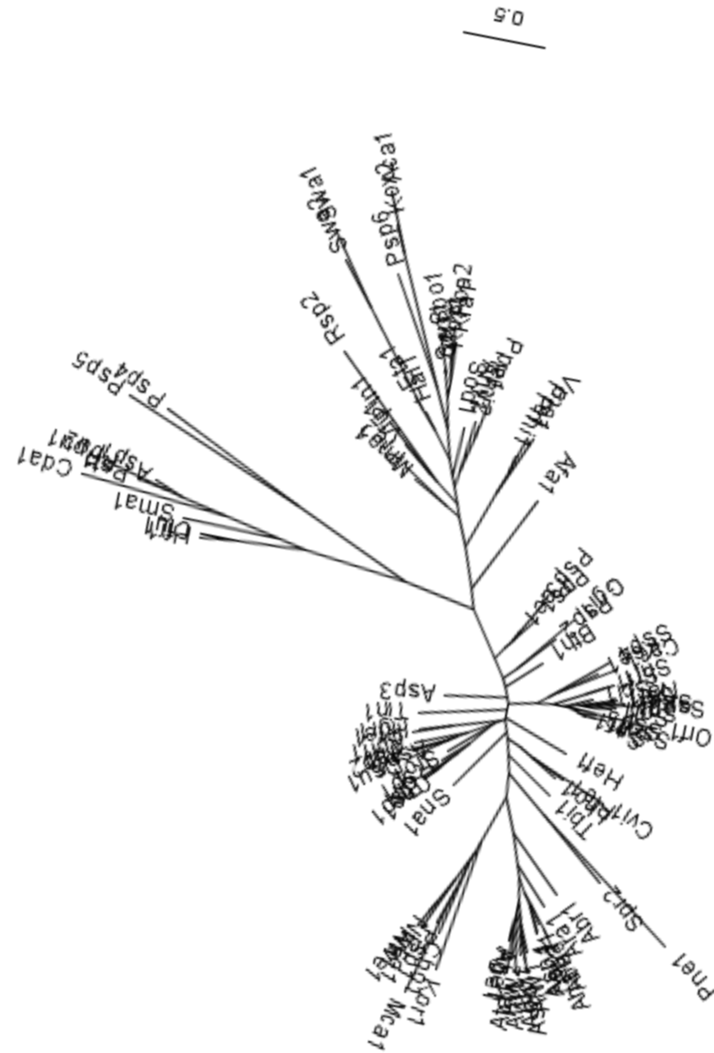

**Fig. 19B**

## RhtB - Maffit

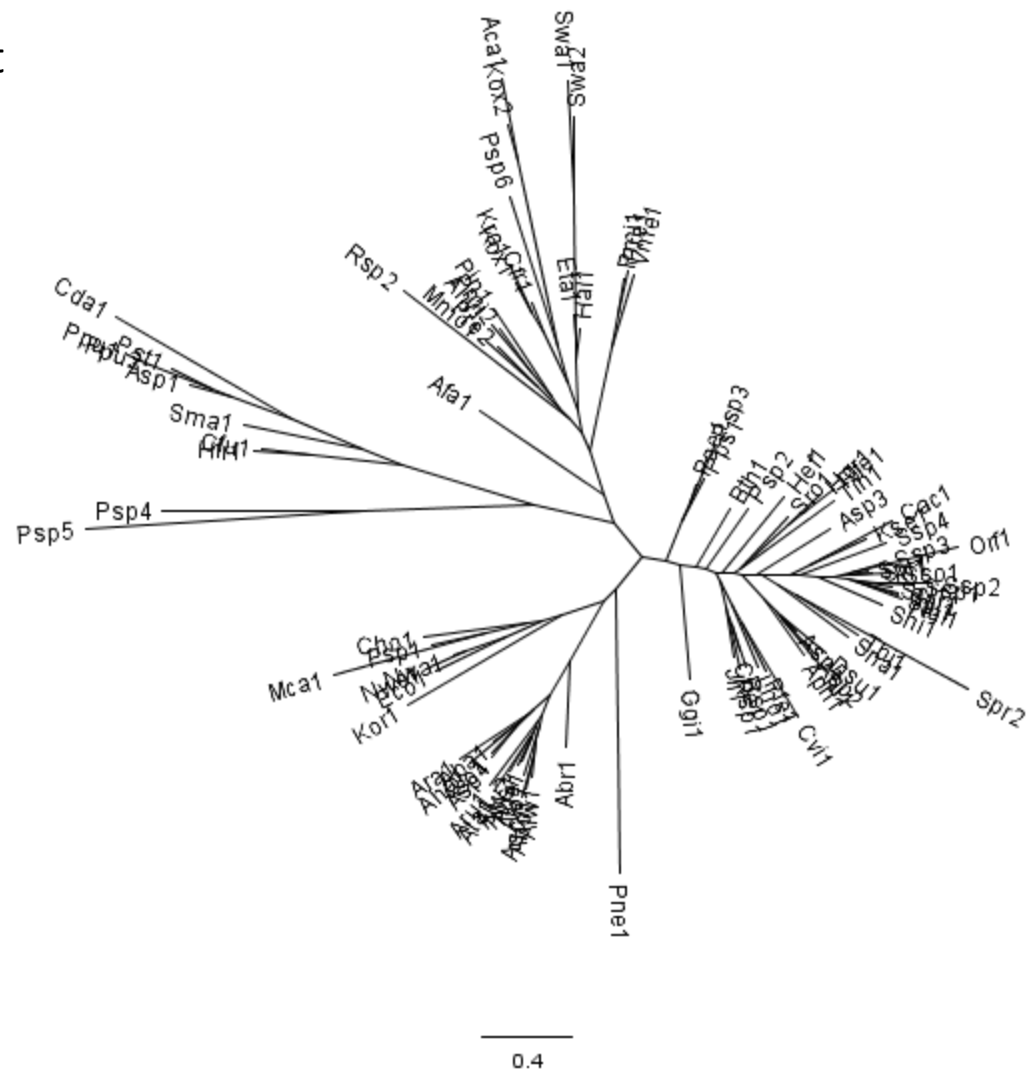

**Fig. 19C**

RhtB - ProbCons

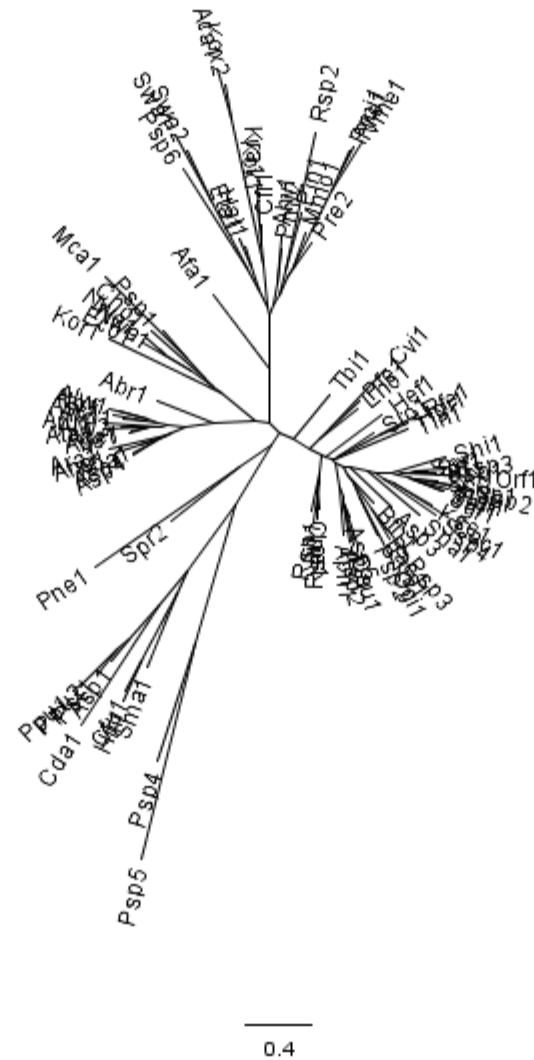

Supplement: S19 Fig — (PDF) [file pone.0137184.s019.pdf]

Fig. 20A

CadD - Clustal

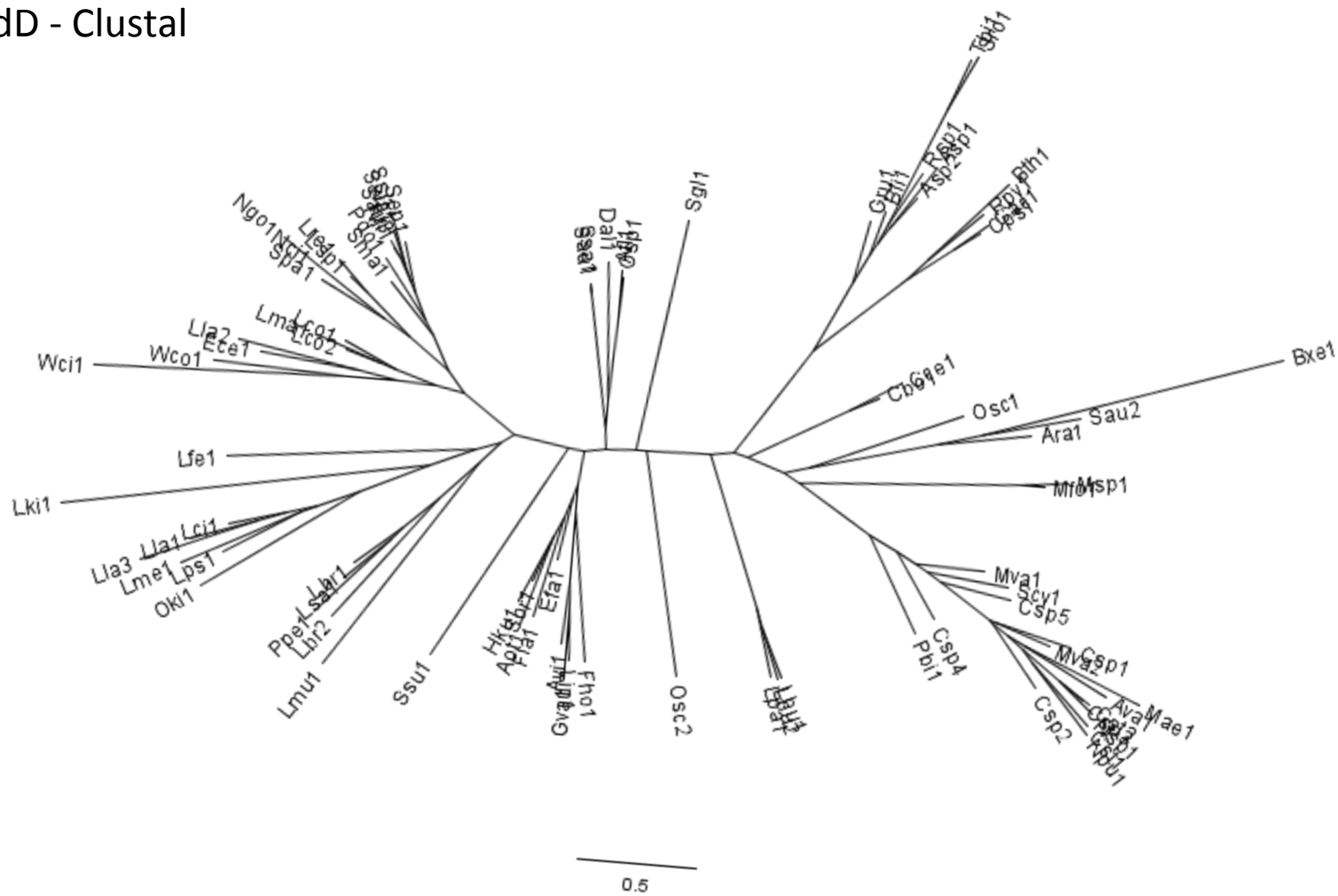

Fig. 20B

CadD - Mafft

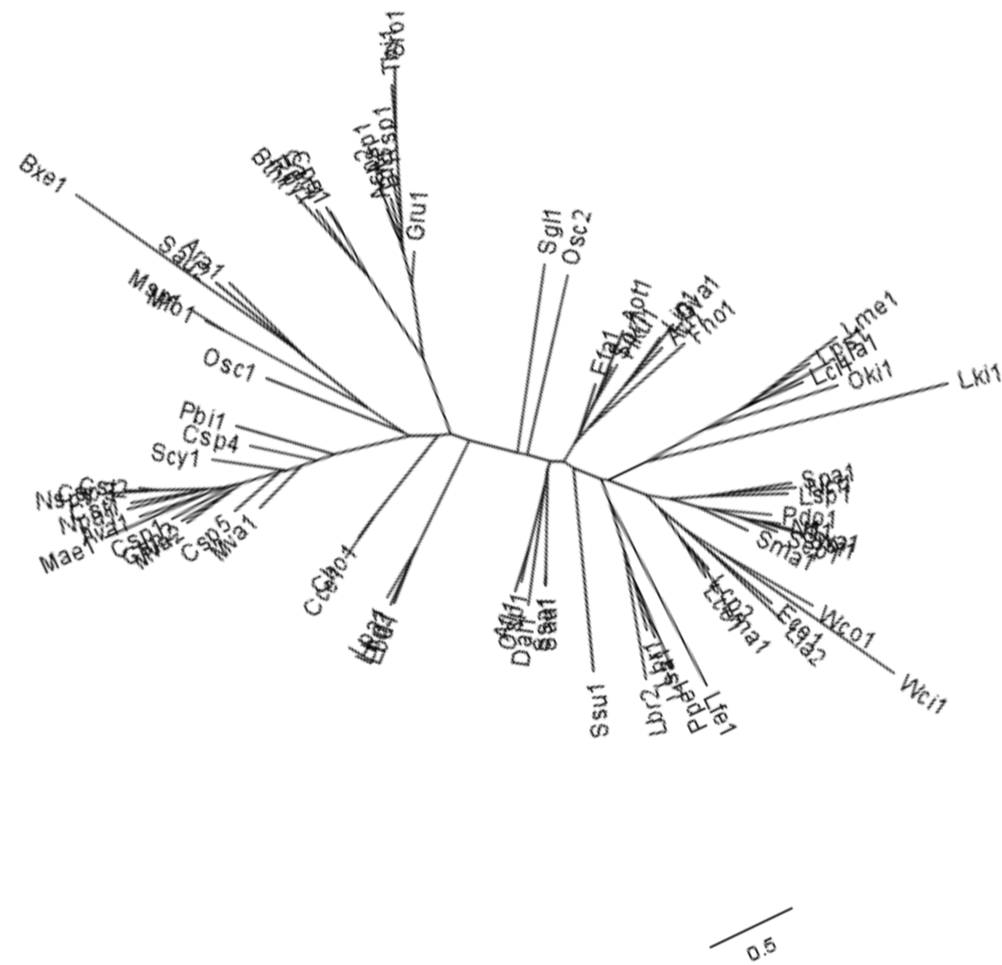

Fig. 20C

CadD - ProbCons

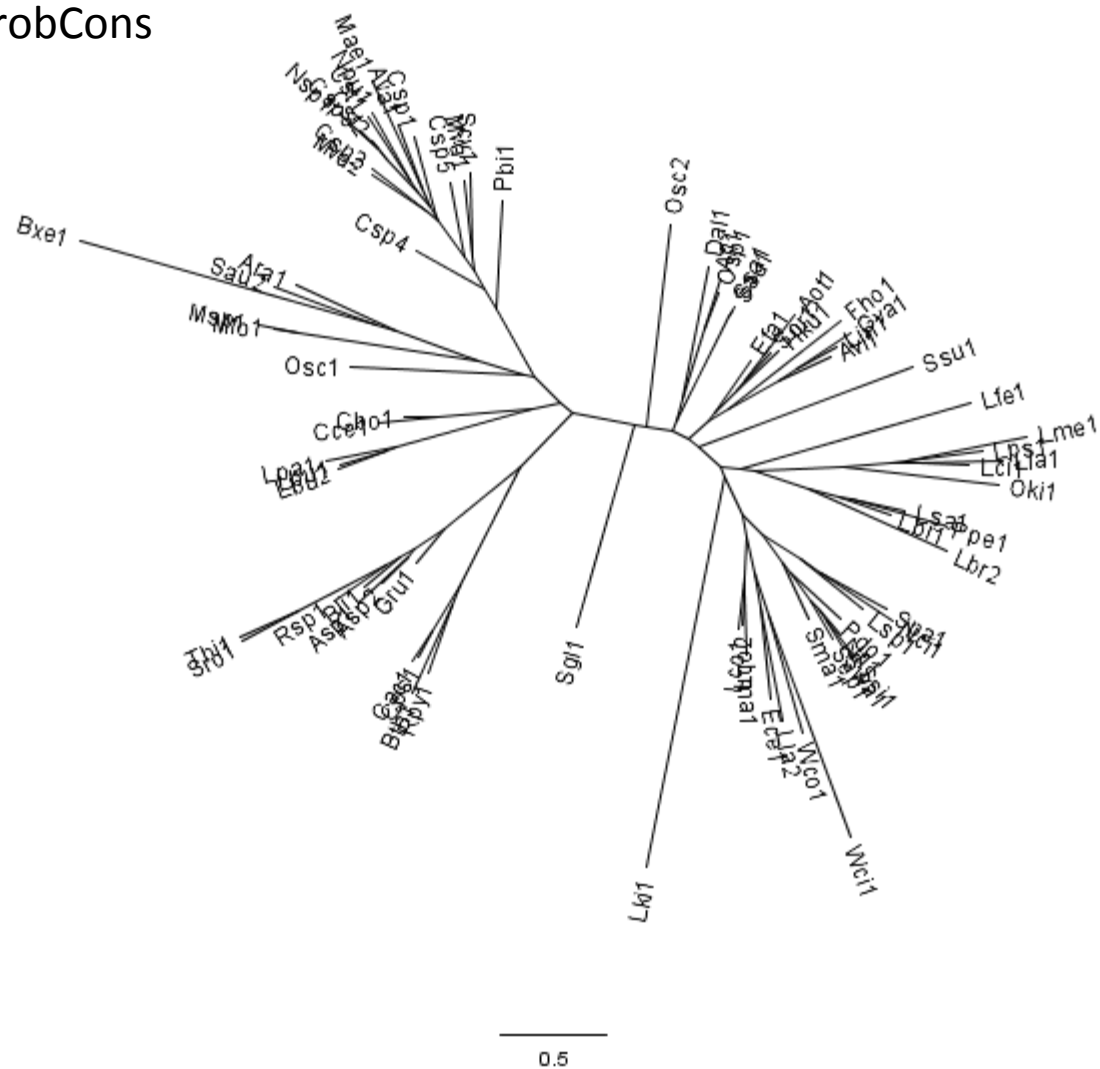

Supplement: S20 Fig — (PDF) [file pone.0137184.s020.pdf]

**Fig. 21A**

CaCA2 - Clustal

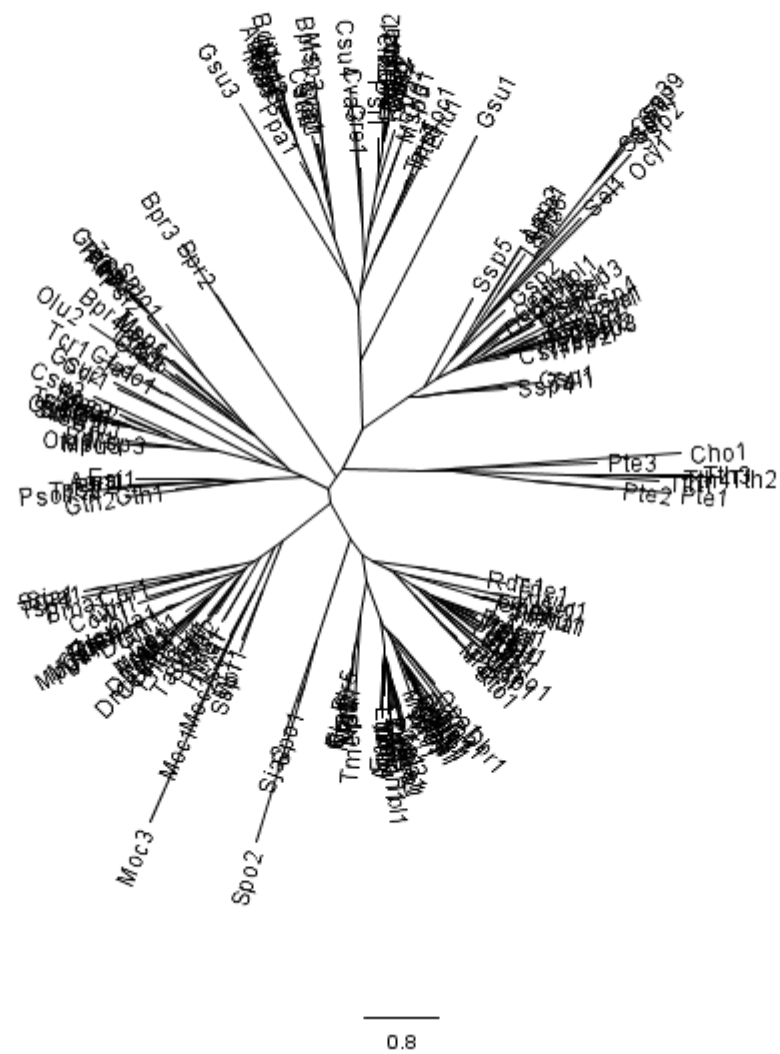

**Fig. 21B**

## CaCA2 - Maffit

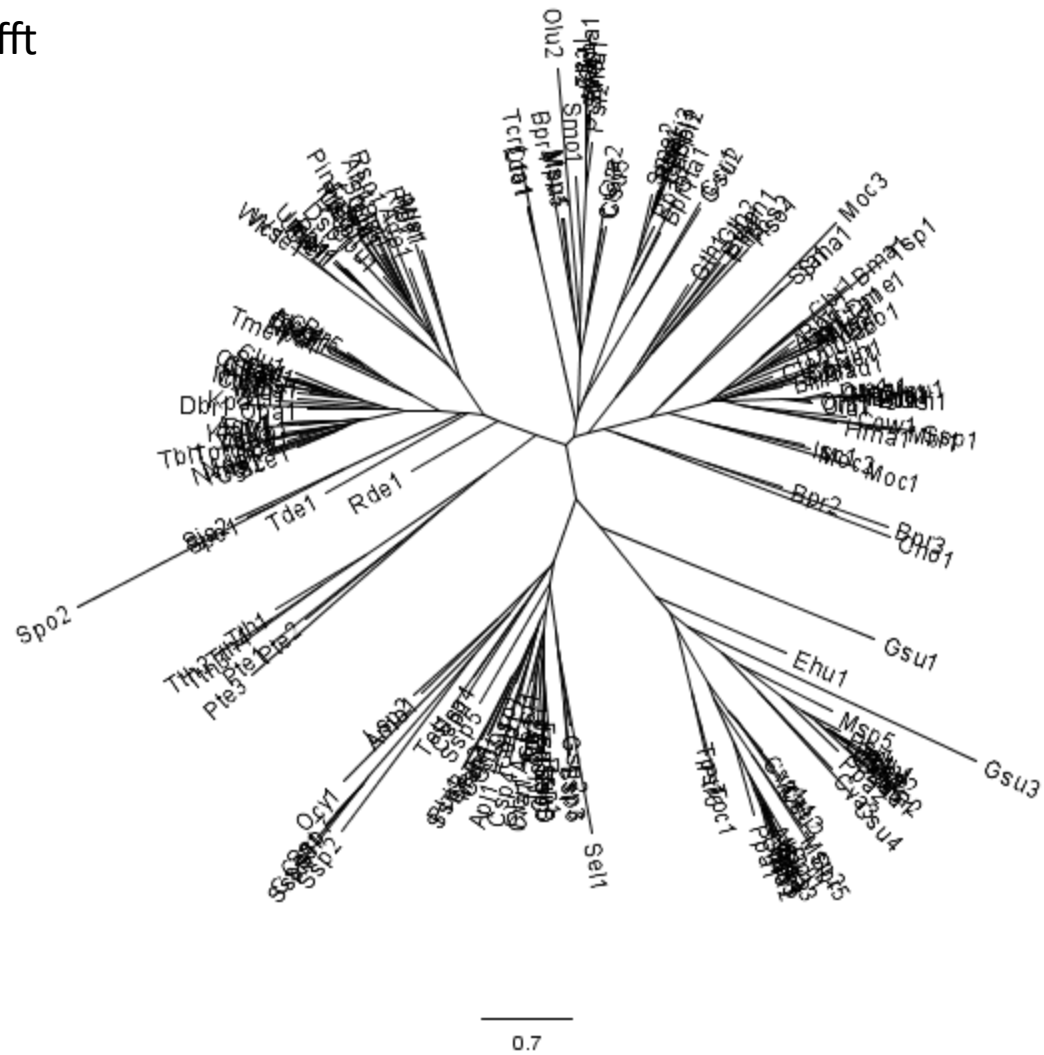

**Fig. 21C**

## CaCA2 - ProbCons

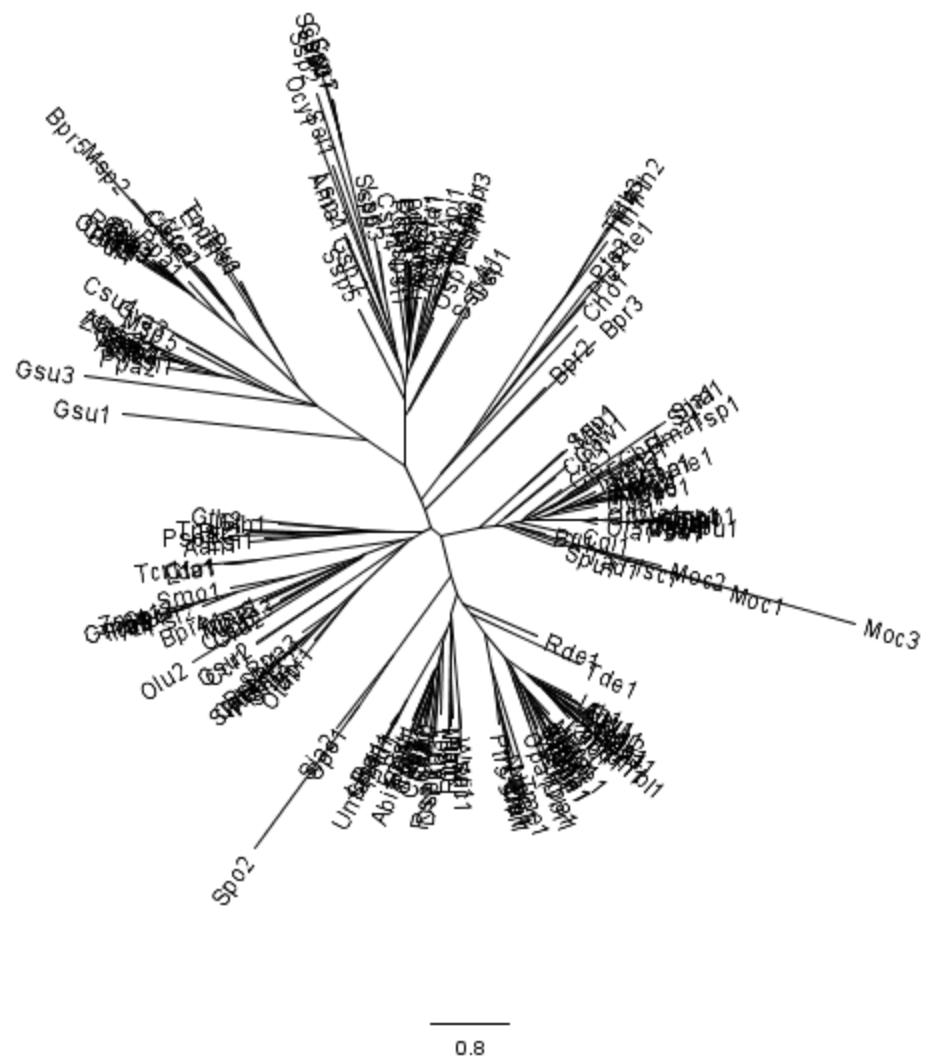

Supplement: S21 Fig — (PDF) [file pone.0137184.s021.pdf]

## MntP - Clustal

## MntP - Clustal

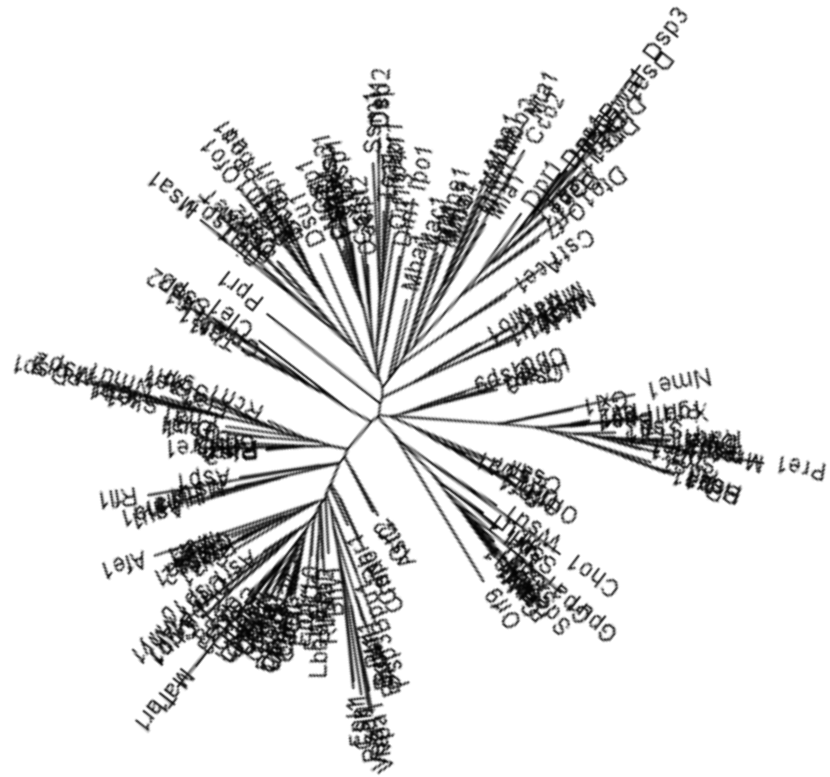

## MntP - Mafft

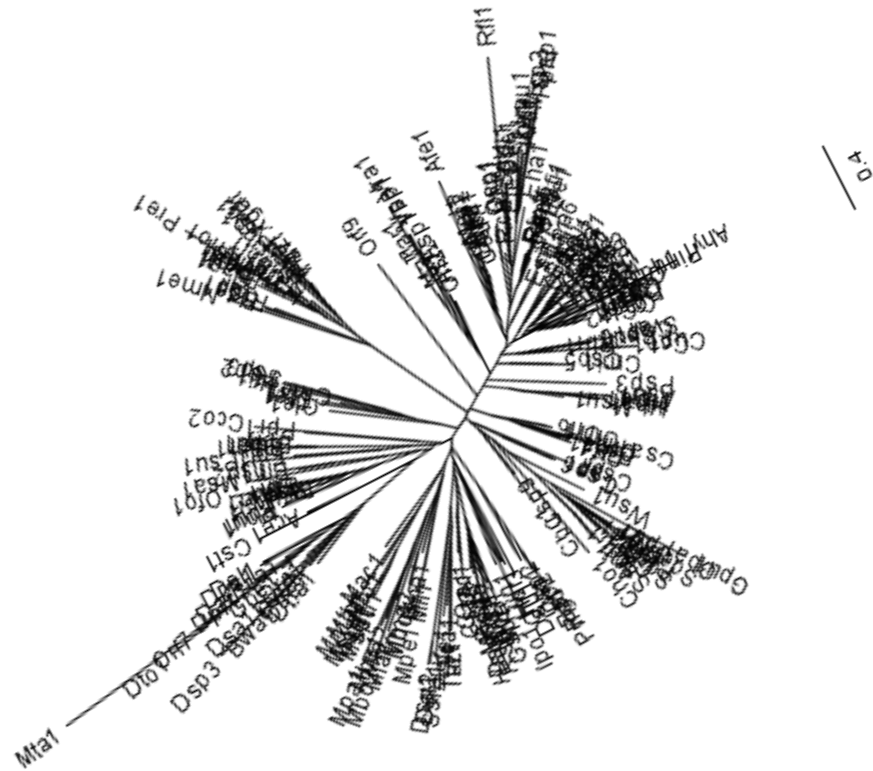

Fig. 22C

MntP - ProbCons

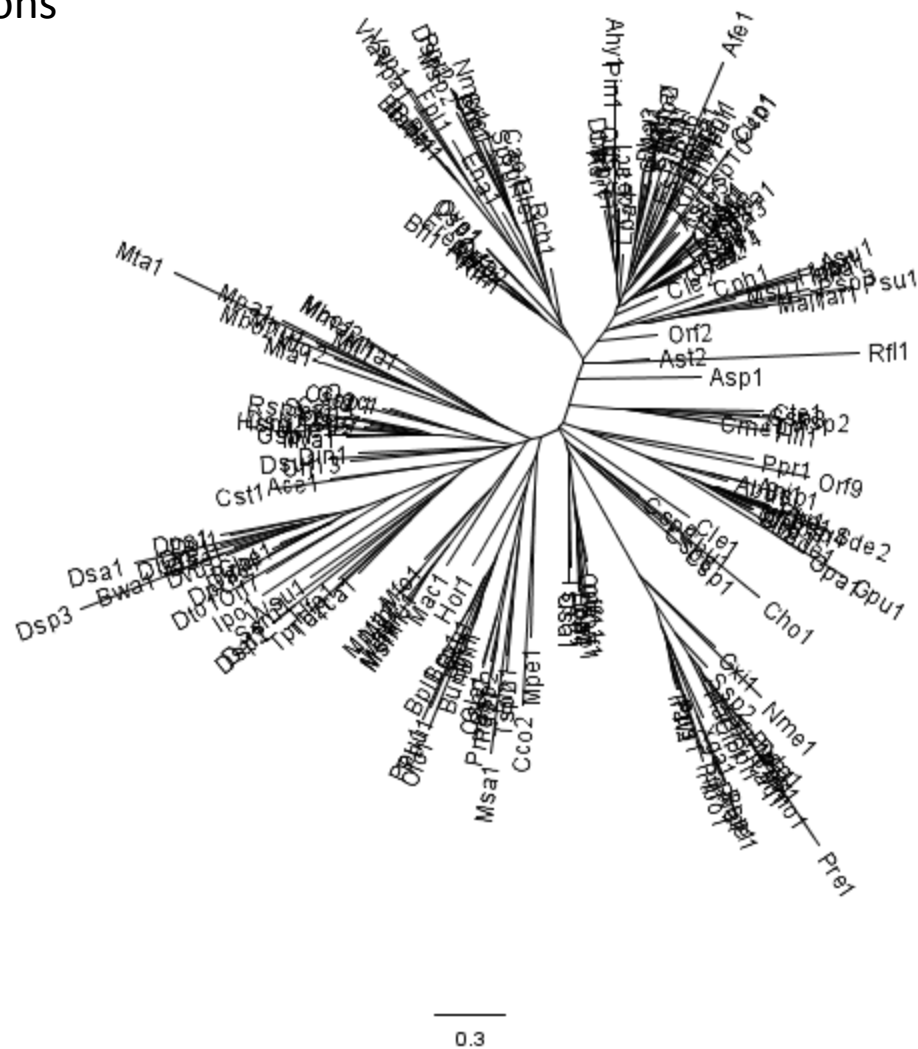

Supplement: S22 Fig — (PDF) [file pone.0137184.s022.pdf]

**Fig. 23A**

ILT - Clustal

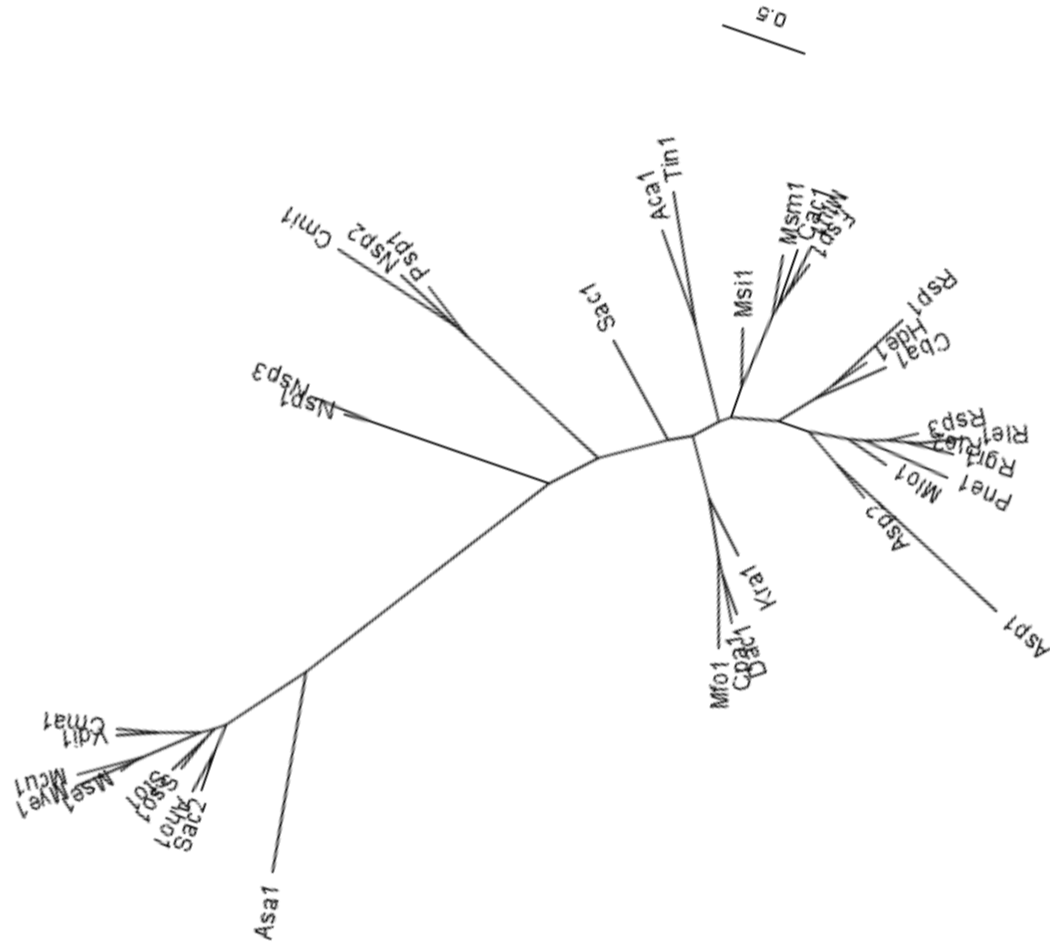

**Fig. 23B**

# ILT - Mafft

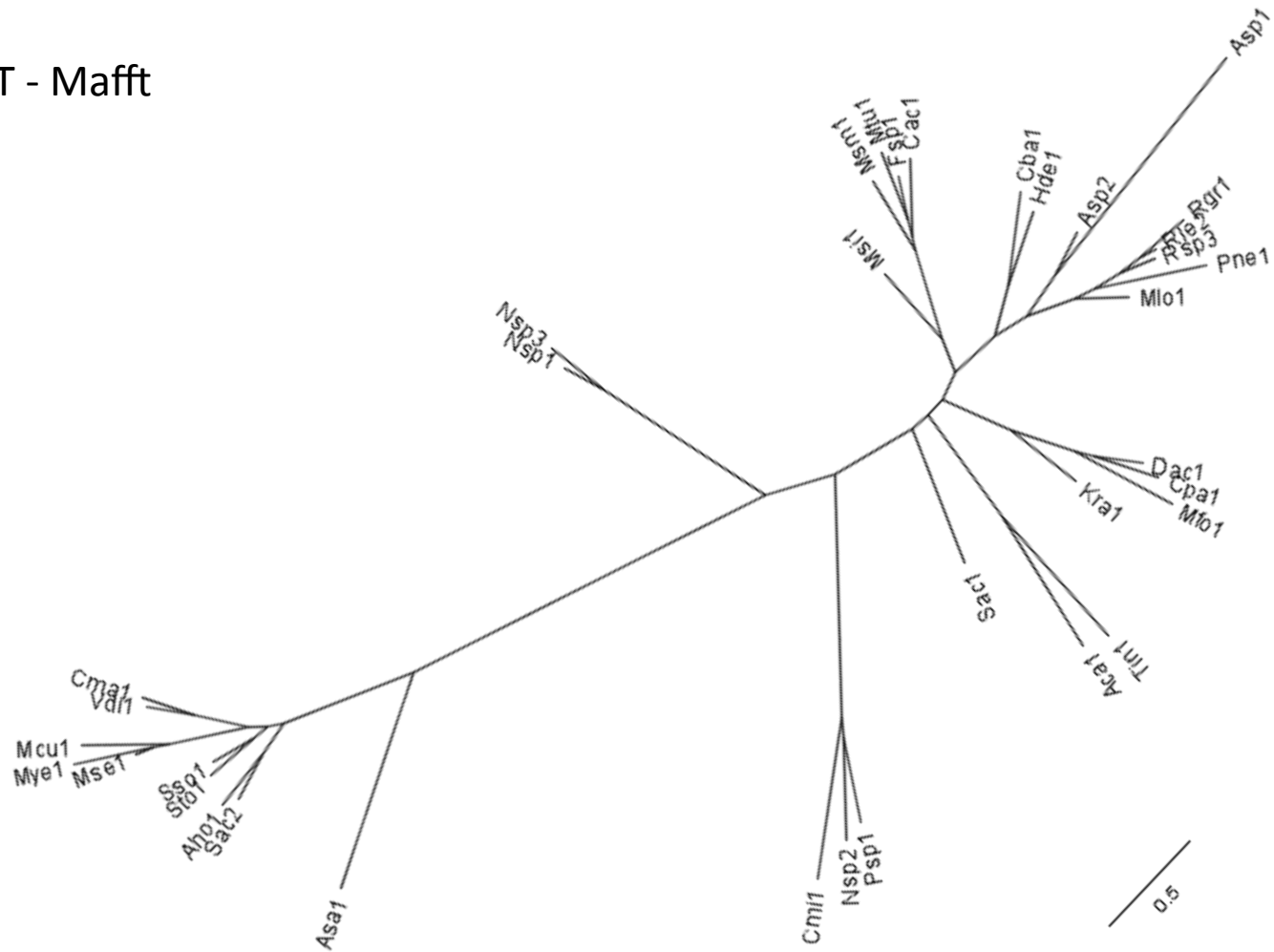

**Fig. 23C**

ILT – ProbCons

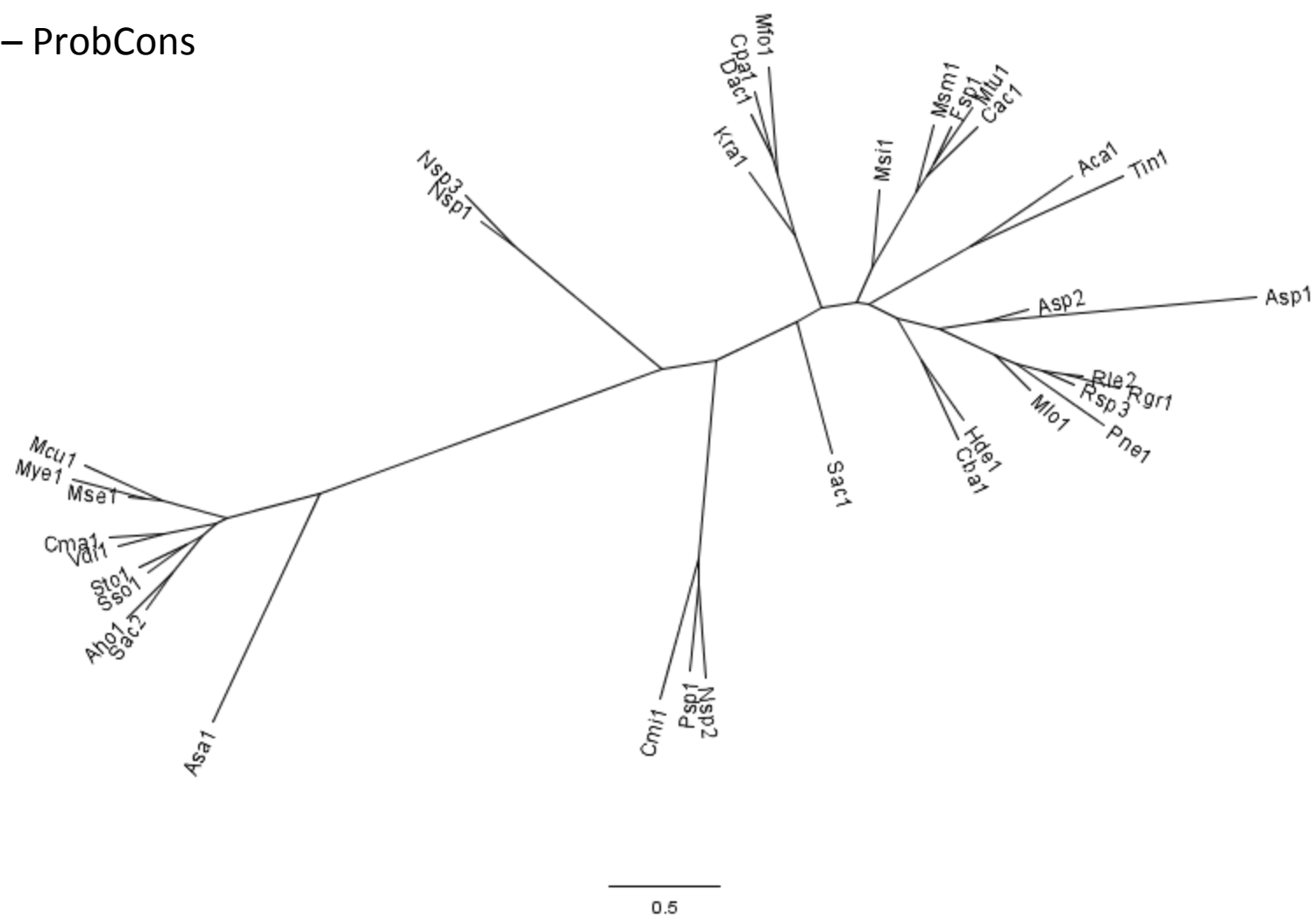

Supplement: S23 Fig — (PDF) [file pone.0137184.s023.pdf]

**Fig. 24A**

TerC – Clustal

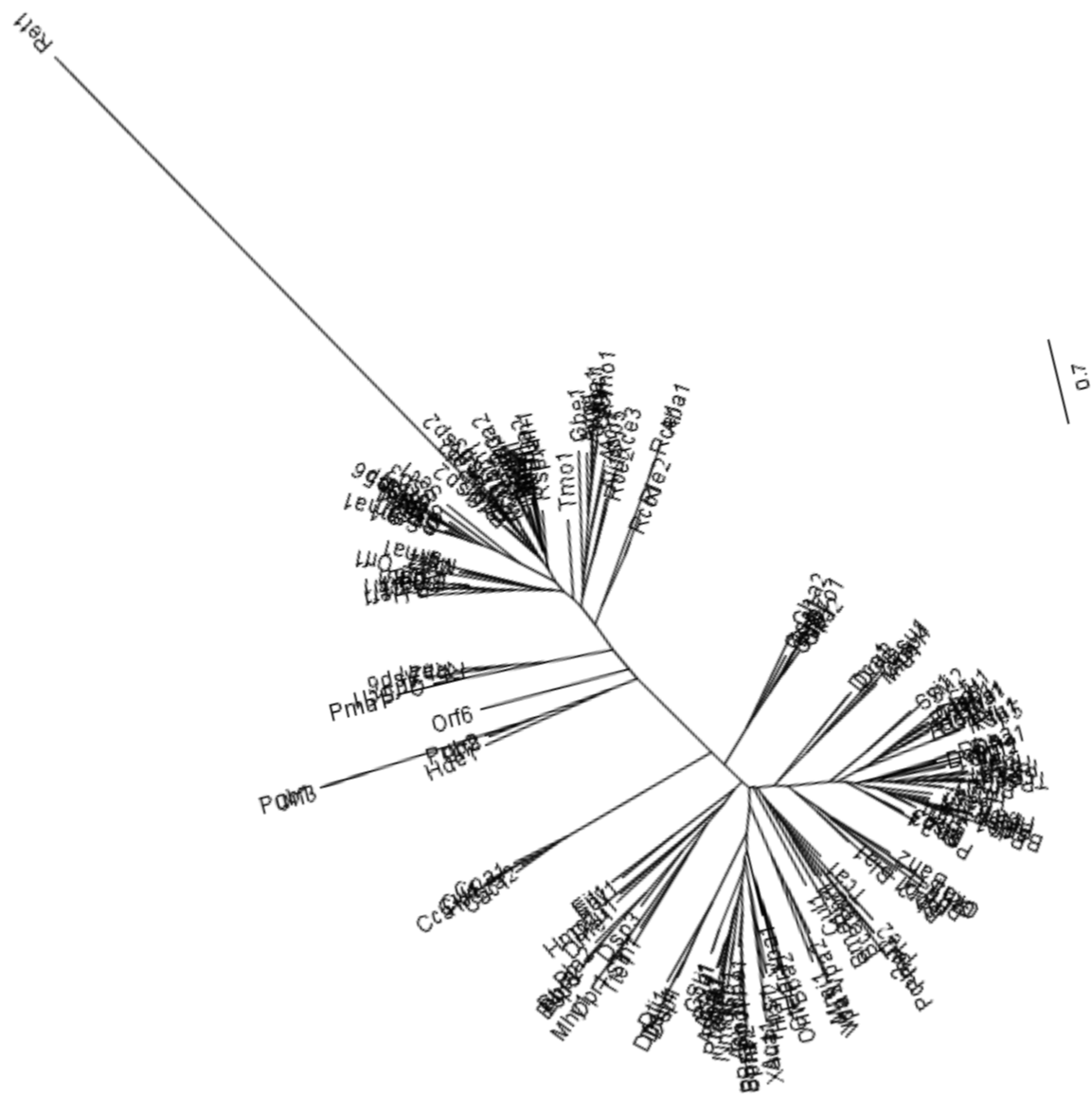

## TerC - Maffit

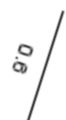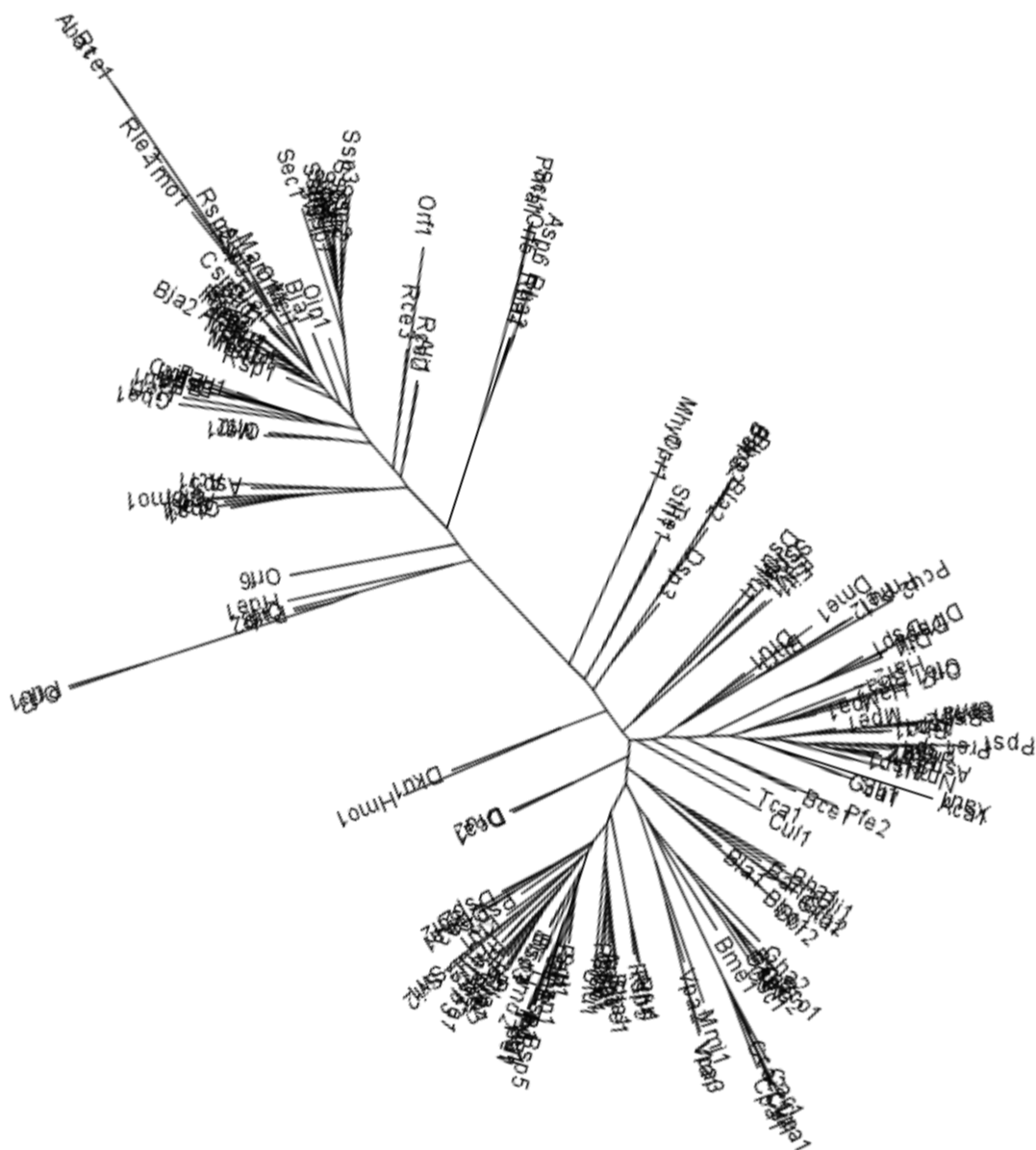

Fig. 24C

TerC - ProbCons

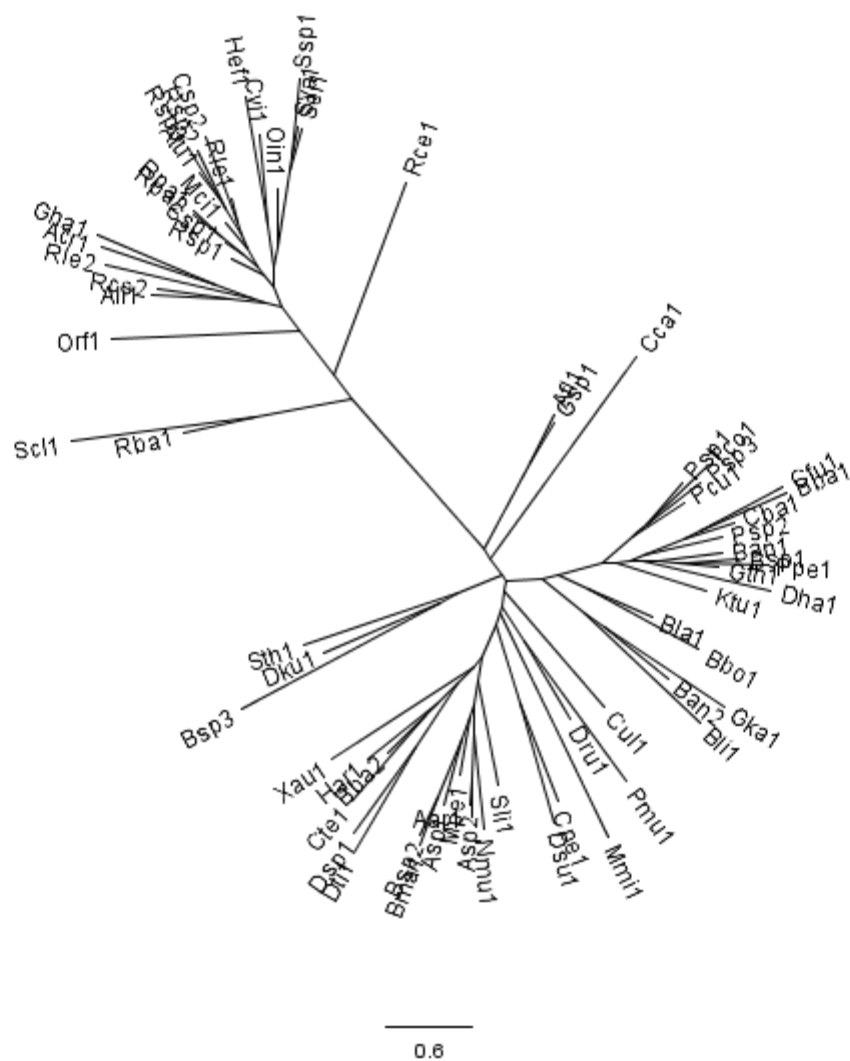

Supplement: S24 Fig — (PDF) [file pone.0137184.s024.pdf]

Fig. 25A

NAAT- Clustal

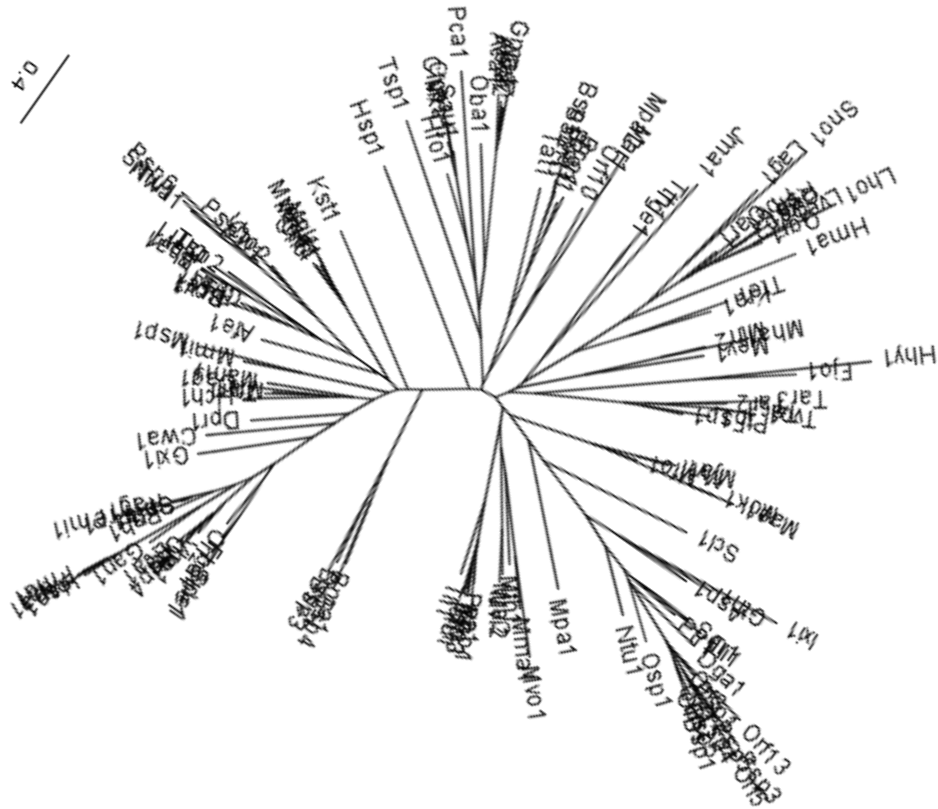

**Fig. 25B**

## NAAT - Mafft

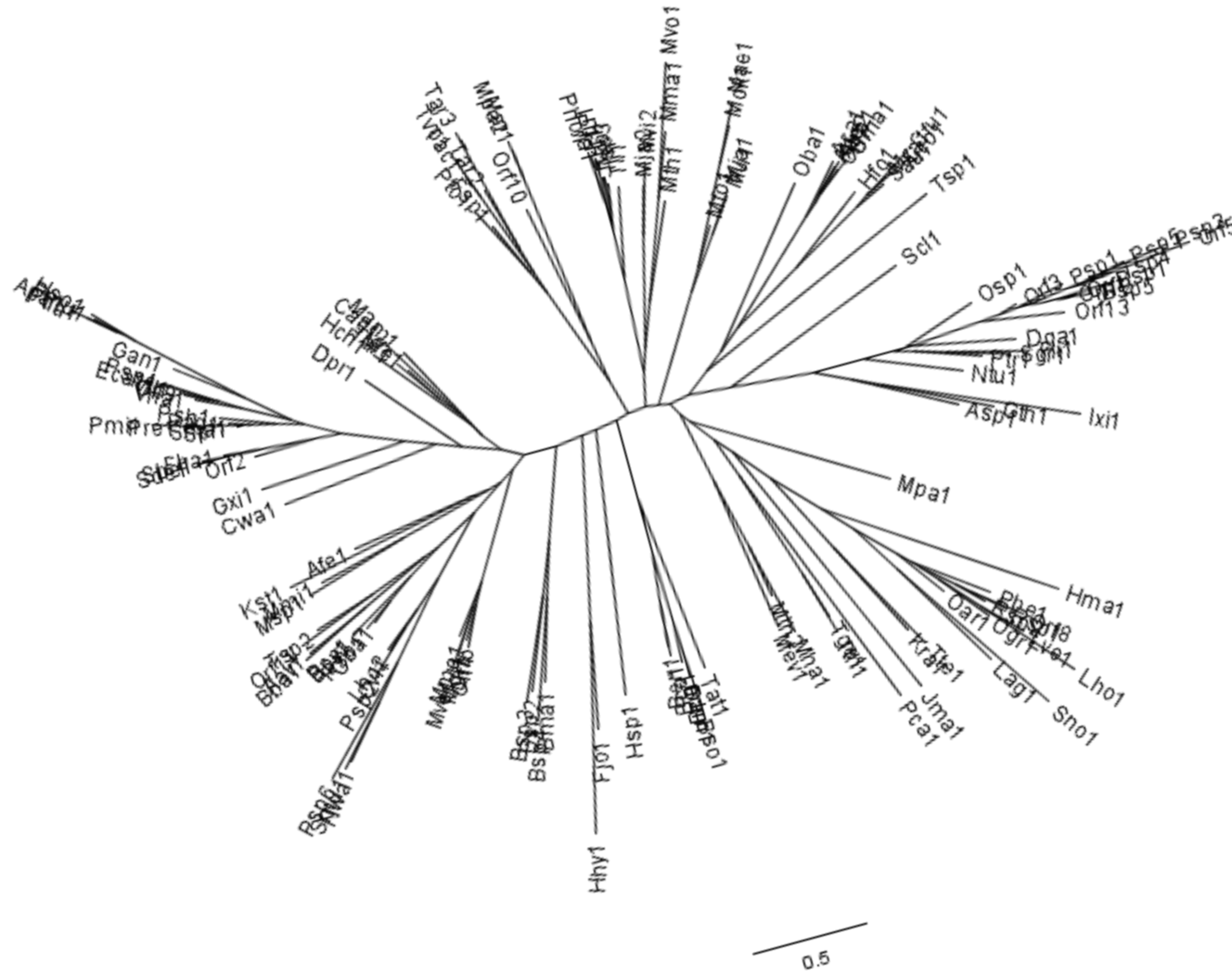

Fig. 25C

NAAT - ProbCons

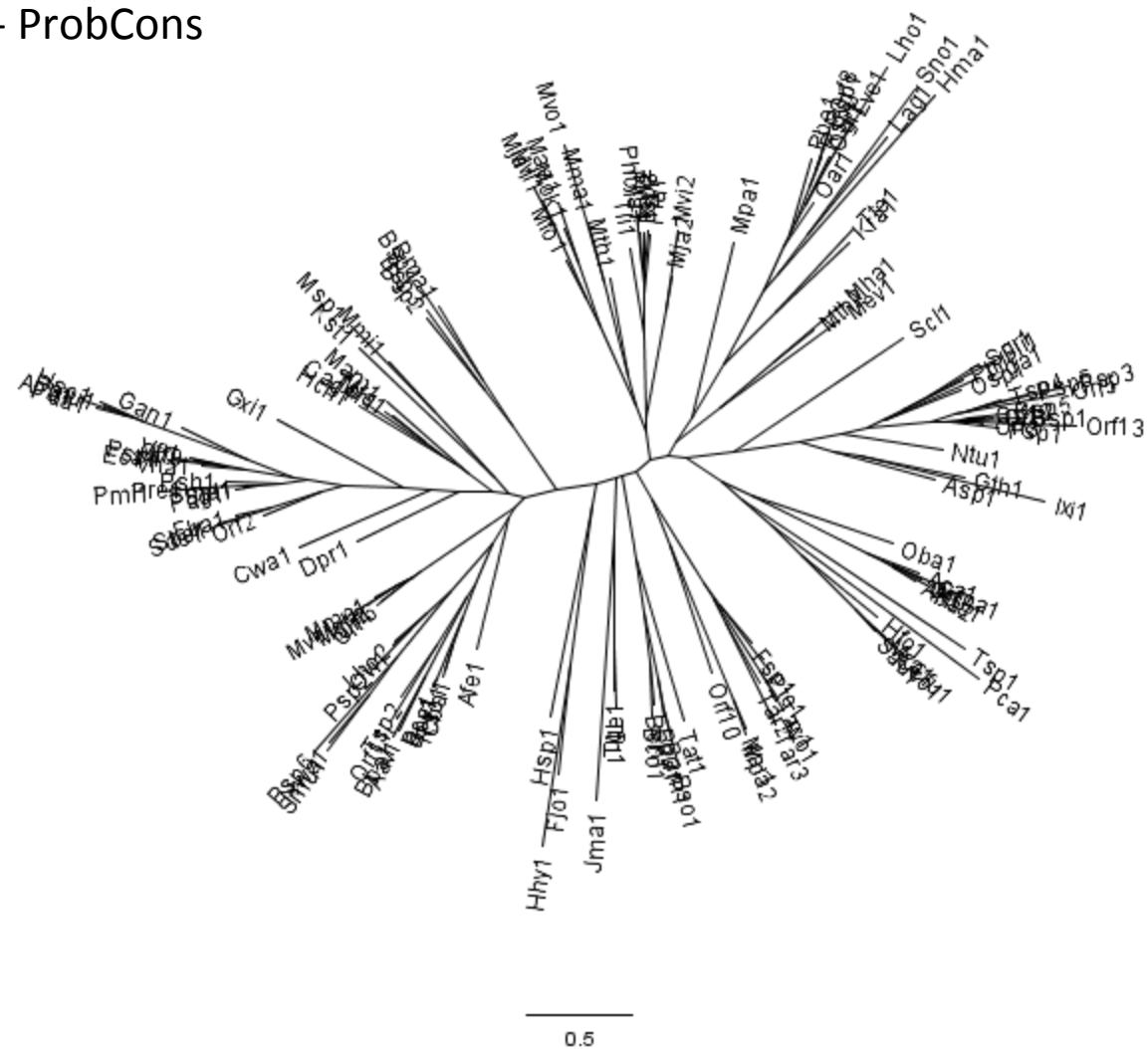

Supplement: S25 Fig — (PDF) [file pone.0137184.s025.pdf]

**Fig. 26A**

NicO – Clustal

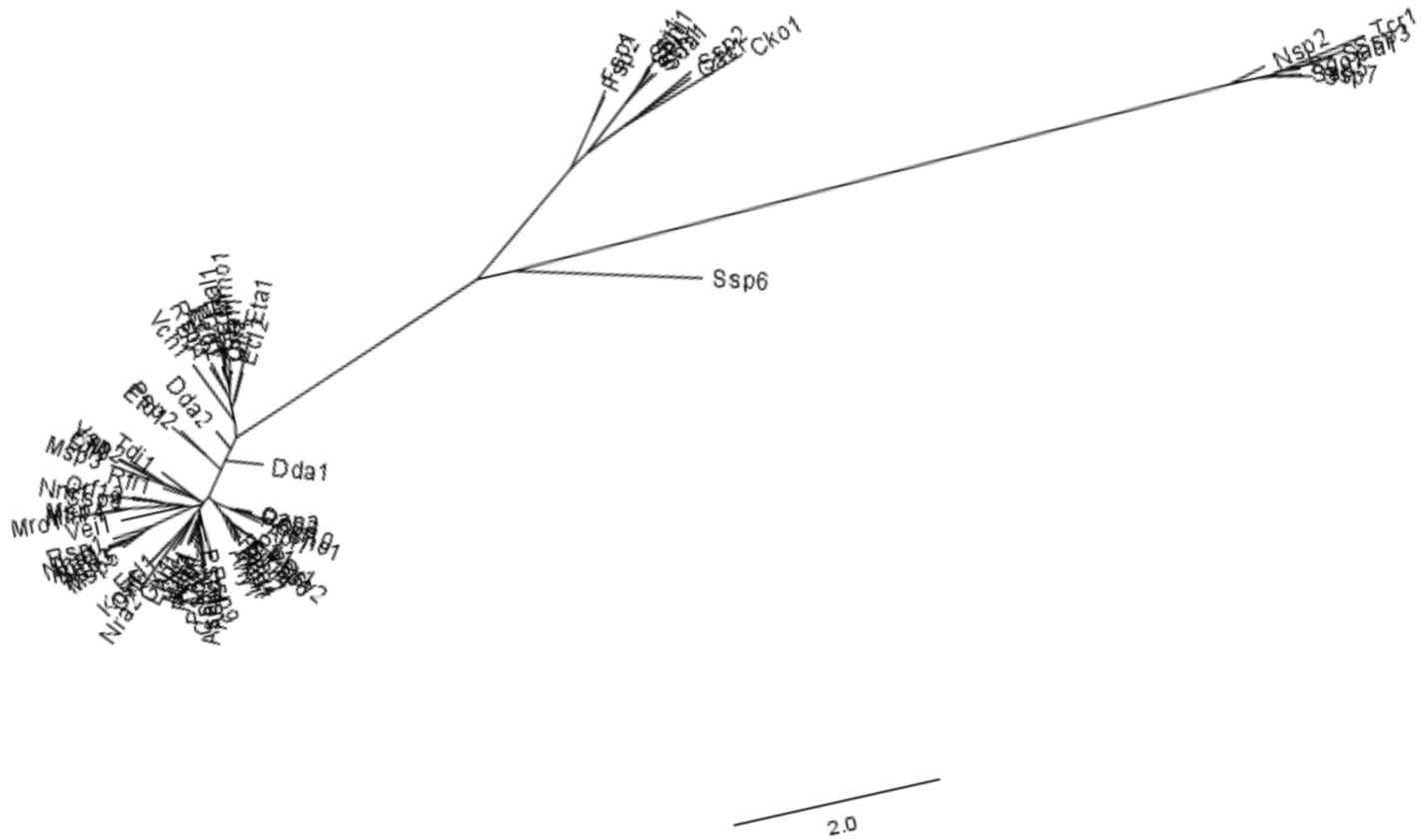

# NicO - Maffit

[illegible]

**Fig. 26C**

# NicO - ProbCons

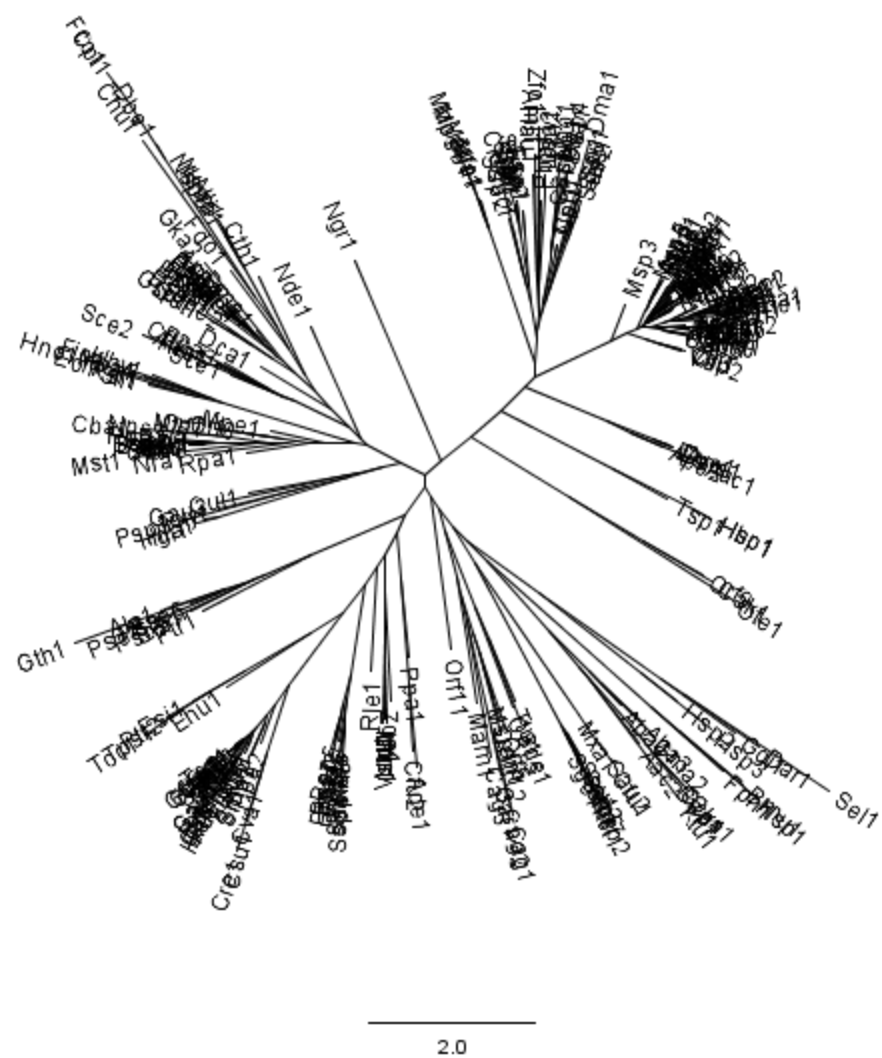

Supplement: S26 Fig — (PDF) [file pone.0137184.s026.pdf]

Fig. 27A

GAP – Clustal

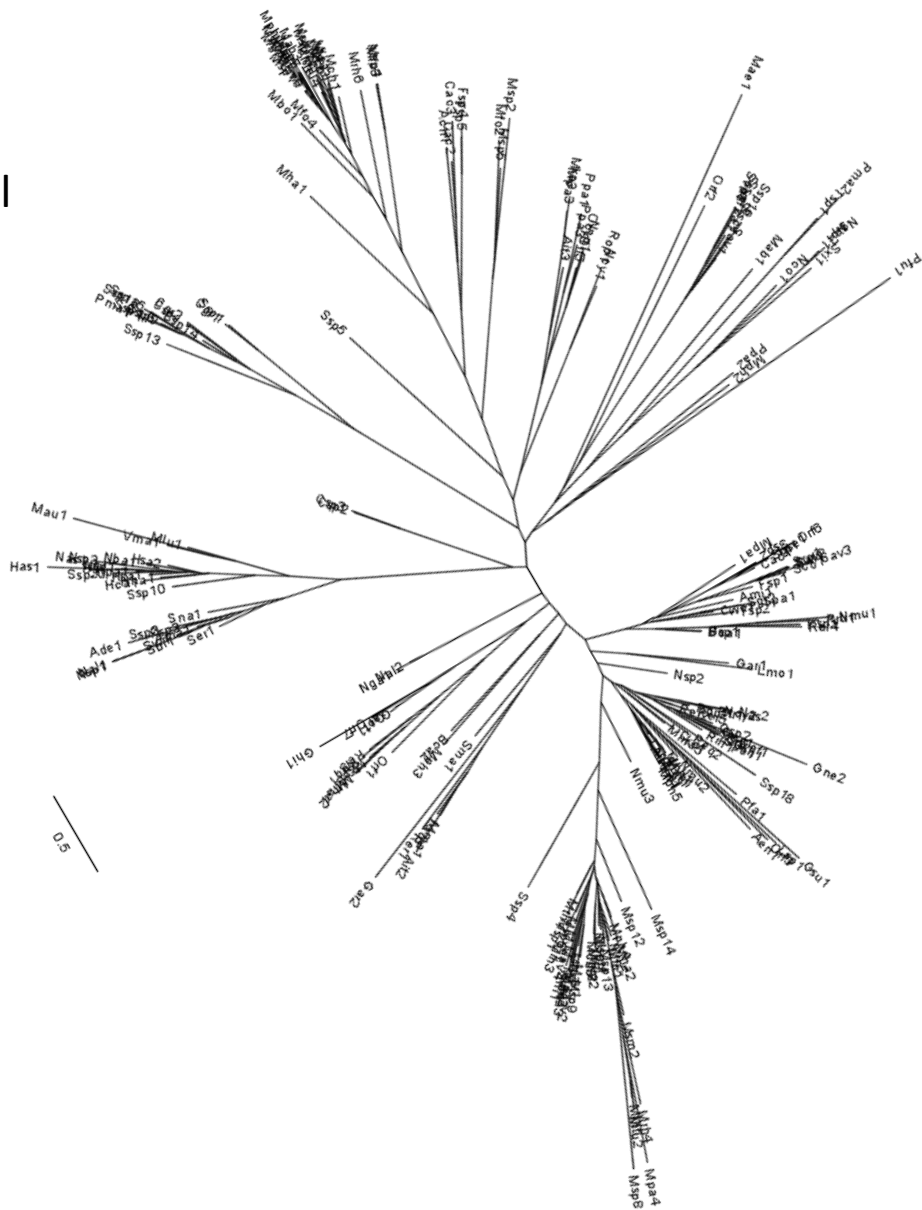

**Fig. 27B**

GAP - Mafft

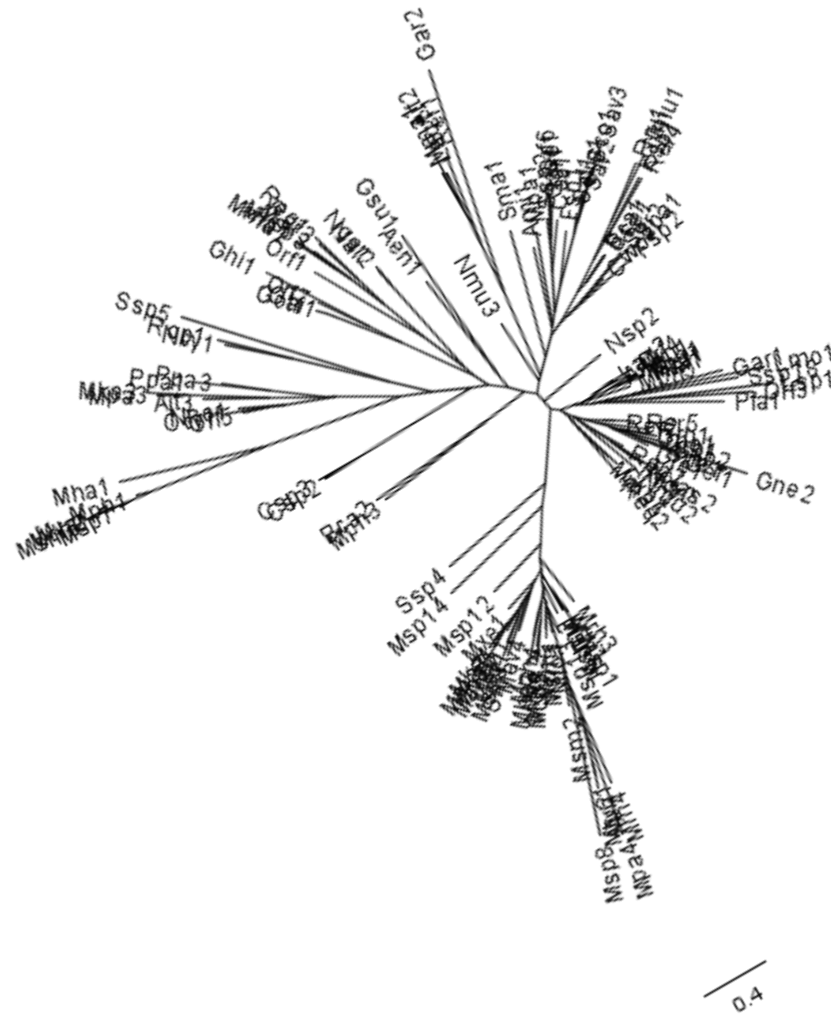

**Fig. 27C**

## GAP - ProbCons

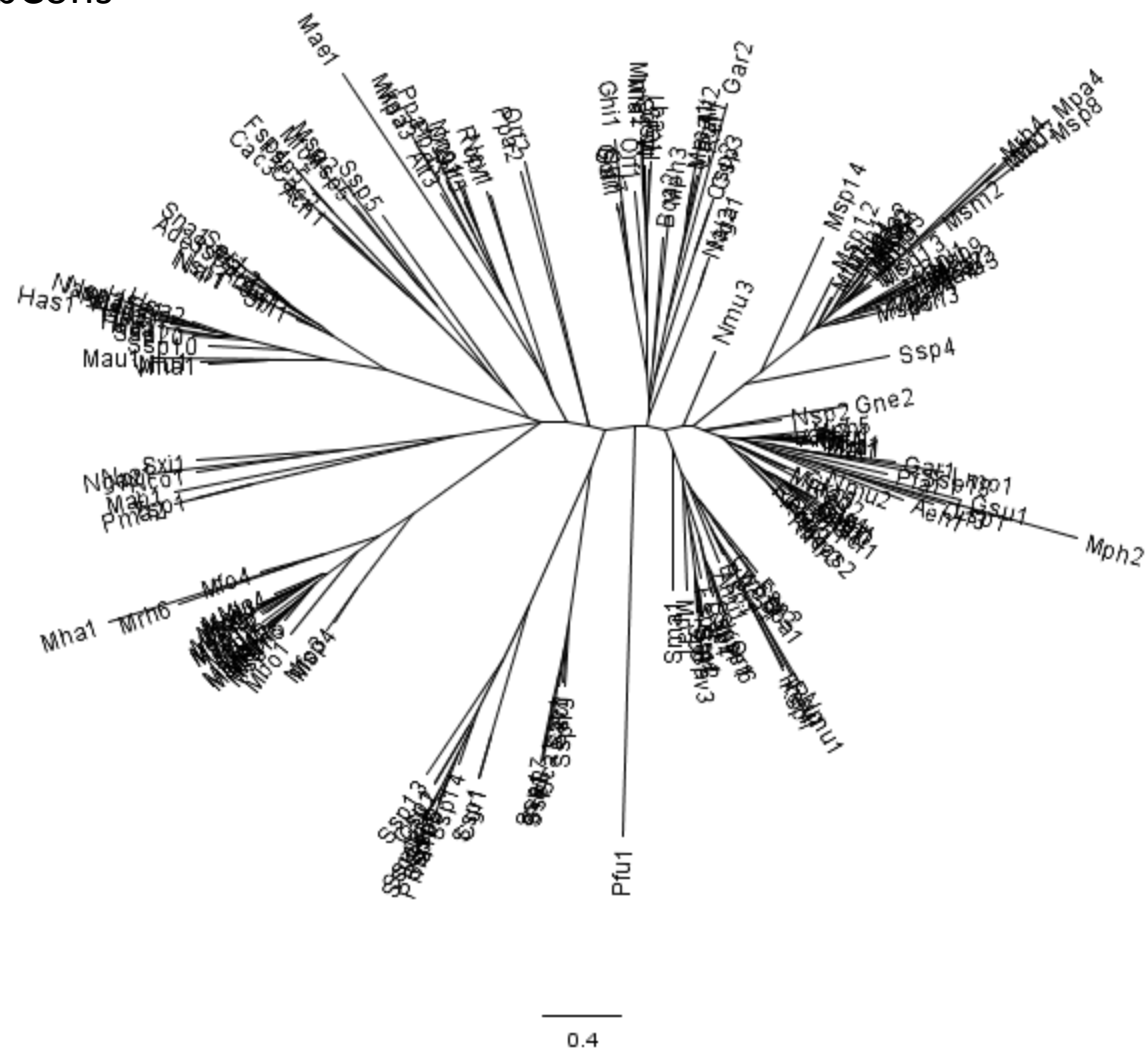

Supplement: S27 Fig — (PDF) [file pone.0137184.s027.pdf]

Fig. 28A

DsbD – Clustal

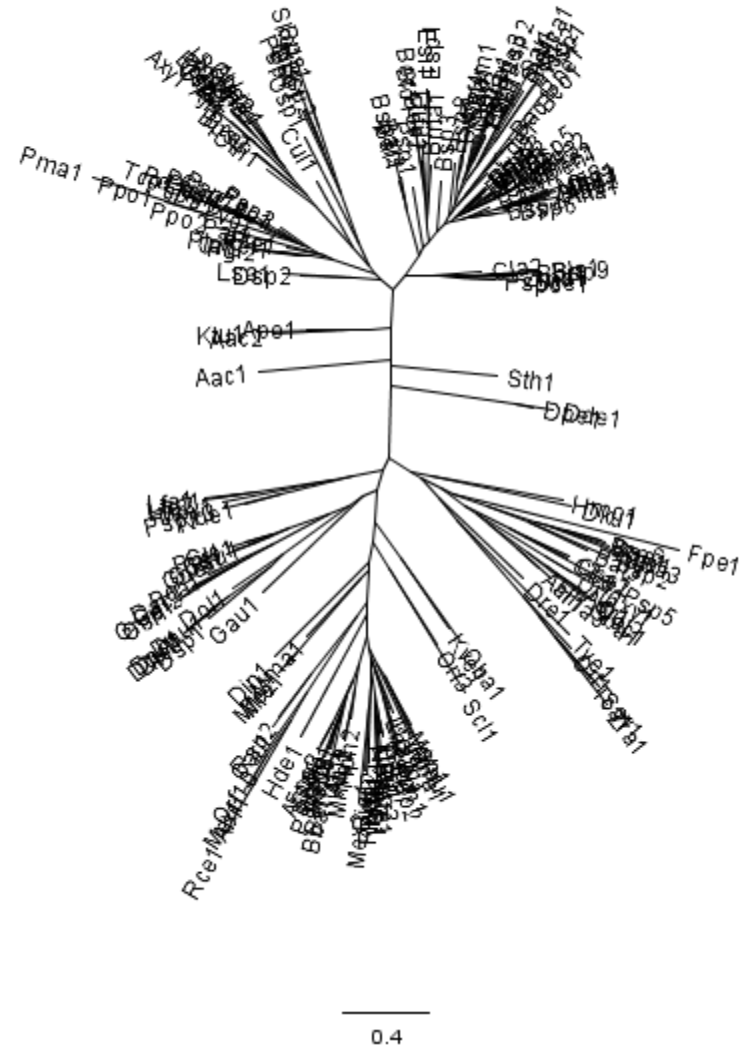

**Fig. 28B**

## DsbD - Maffit

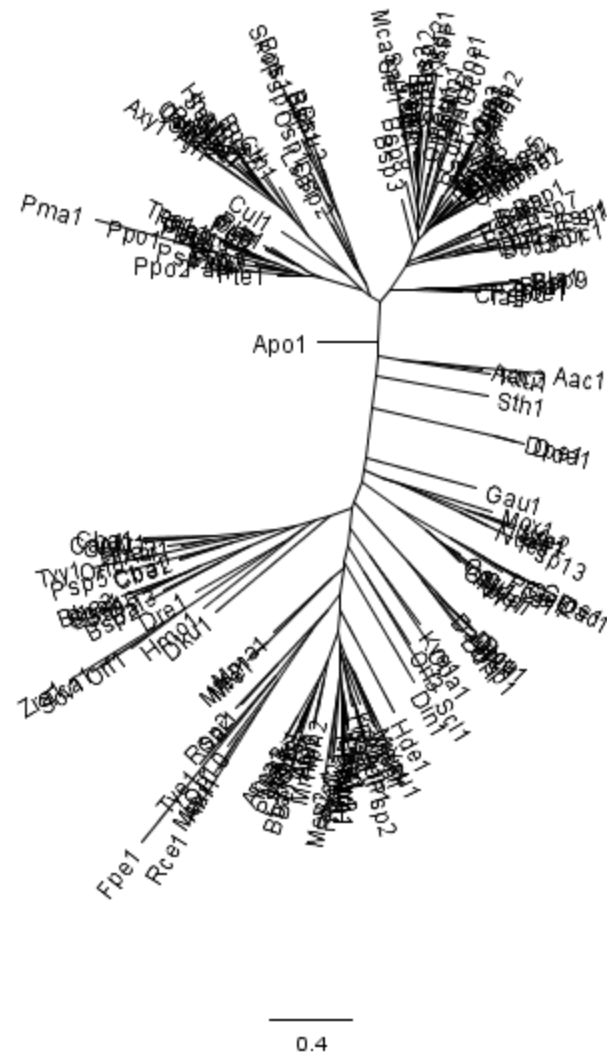

Fig. 28C

DsbD - ProbCons

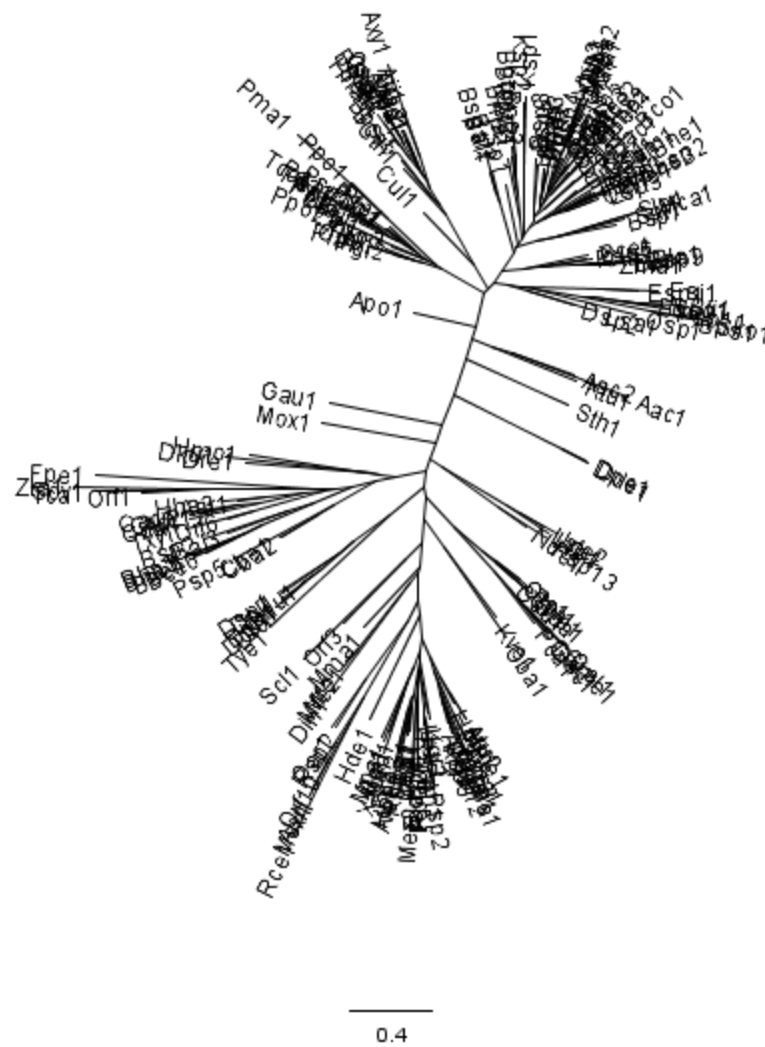

Supplement: S28 Fig — (PDF) [file pone.0137184.s028.pdf]

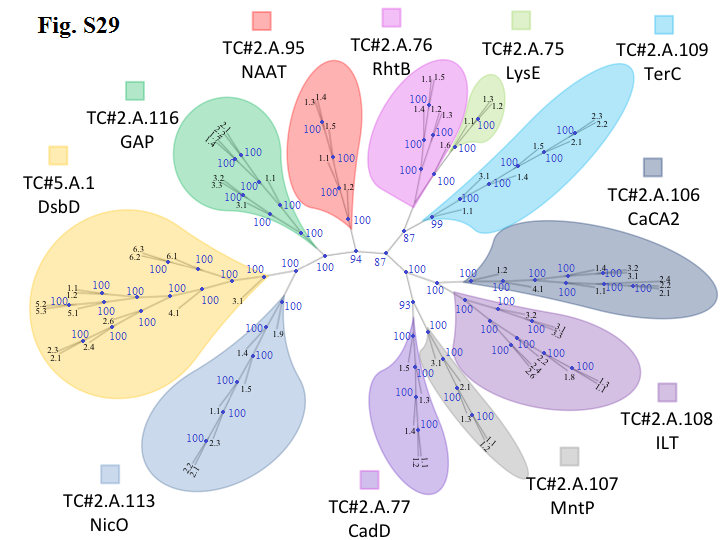

Supplement: S29 Fig — The tree was generated using the SuperFamilyTree program and viewed using FigTree. It depicts the evolutionary relationship between the 11 different families in this study. Clustering indicates closer phylogenetic relationships. The tree is based on tens of thousands of BLAST bit scores generated with the SFT1 program where every protein was compared with every other protein included in the analysis. The SFT2 program was used to integrate all of the information to show the relationships of the eleven families to each other. Bootstrap values have been added in blue text and located near each node. (TIF) [file pone.0137184.s029.tif]
