# Supplementary material for: The LysE Superfamily of Transport Proteins Involved in Cell Physiology and Pathogenesis
Source: PLoS One. 2015 Oct 16;10(10):e0137184. doi: 10.1371/journal.pone.0137184 (PMC4608589; doi:10.1371/journal.pone.0137184)
Supplement: S2 Fig — (A) LysE vs. RhtB. (B) RhtB vs. CadD. (C) LysE vs. CadD. (PDF) [file pone.0137184.s002.pdf]

Fig. S2A

```
# 1: A_Sequence: Pst1 (2.A.75.1.1 homologue)
# 2: B_Sequence: Bth1 (2.A.76.1.5 homologue)
# Matrix: EBLOSUM62
# Gap_penalty: 8.0
# Extend_penalty: 2.0
#
# Length: 171
# Identity:      57/171 (33.3%)
# Similarity:    87/171 (50.9%)
# Gaps:          20/171 (11.7%)
# Score: 159.0
#=======================================
                         1                        2
A_Sequence         1 LILPLGPQNAFVLN----QGVKRHYHLMTATLCSLSDVVLICAGIFGGSA     46
                     ::|| || : :||:    :|||  |         : | ||:     | ::
B_Sequence         1 ILLP-GPNSMYVLSLAAQRGVKAGYRAACGVF--VGDTVLMVLSAAGVAS     47
                         1                        2

                             3
A_Sequence        47 LLQQSPLLLTVITWAGVAFLLWYGWGALRTAFRRELALA-SGLDIRQS-R     94
                     ||: :||| :|: : | |:||: | | || |:|:    | :| |:|::
B_Sequence        48 LLKANPLLFSVVKYGGAAYLLYIGSGMLRGAWRKLARPADAGADVRRAVD     97
                              3

                               4                                   5
A_Sequence        95 G-RIIATLLAVTWLNPHVYLDTFVVLGSLGSQFPD---TH-ARQWFALGT    139
                     | |     | |: |||   |  | :  |   || |    | |  :  ||
B_Sequence        98 GERPFRKALVVSLLNPKAIL--FFI--SFFIQFVDPSYAHPALSFVVLGA    143
                               4                                    5

A_Sequence       140 VS--ASVLWFFGLALLAAWLA    158
                     ::  || ::   |    | ||
B_Sequence       144 IAQFASFVYLSTLIFTGARLA    164
#---------------------------------------
============ FINISHED =============
Average Quality (AQ)      18.75 +/- 6.96
Standard score (Z):       20.0
Precise score (Z):  20.1
```

Fig. S2B

```
# 1: A_Sequence: Hal1 (2.A.76.1.5 homologue)
# 2: B_Sequence: Oki1 (2.A.77.1.1 homologue)
# Matrix: EBLOSUM62
# Gap_penalty: 8.0
# Extend_penalty: 2.0
#
# Length: 124
# Identity:      33/124 (26.6%)
# Similarity:    63/124 (50.8%)
# Gaps:          12/124 ( 9.7%)
# Score: 107.0
#=======================================
                          2                       3
A_Sequence         1 IGDAVLIFCAYIGIASLIRSSPFLFSLVKMLGALYLLYLGLKILYSTLAK     50
                     ||: :||  : : :| |::  |  : :: :|| |: : :|||  :|
B_Sequence         1 IGNGILIVMSLL-LAYLLKFIPESW-ILGLLG-LFPITVGLKTFFS----     43
                          2                       3

                                            4
A_Sequence        51 KGQEQSAAKEEPEHTFRKALTLSLTNPKA--ILFYVSFFVQFIDMDYAHT     98
                     |  | : ||    |  |  : ::||   |  :  |: ||     :|:::
B_Sequence        44 KEDETAKAKASDAHLIRDVVLMTLTTCSADNLAIYIPFFA---SVDFSYL     90
                                            4

                          5
A_Sequence        99 GVSFAILAVILEMISFCYMTLLIF    122
                      |   :  :||  :||  : :  |
B_Sequence        91 PVILIVFLLILSAVSFTALKITKF    114
                          5
#---------------------------------------
============ FINISHED =============
Average Quality (AQ)      23.76 +/- 7.02
Standard score (Z):       12.0
Precise score (Z):  11.9
```

Fig. S2C

```
# 1: A_Sequence: Asp2 (2.A.75.1.3 homologue)
# 2: B_Sequence: Cth1 (2.A.77.1.2 homologue)
# Matrix: EBLOSUM62
# Gap_penalty: 8.0
# Extend_penalty: 2.0
#
# Length: 144
# Identity:      38/144 (26.4%)
# Similarity:    65/144 (45.1%)
# Gaps:          14/144 ( 9.7%)
# Score: 92.0
#=======================================
                                 2                             3
A_Sequence         1 LRQGLRREHVMPVVLVCALSDAVLLQVGVWGMGGVLLARPEWAQFMRWAG     50
                     :    || |:: :      :  ||  :  :  ||::: | ||   :   |
B_Sequence         1 INANFRRRHIV-IGQYLGFTTIVLASLPGF-FGGLIVPR-EWIGLL---G     44
                                 2                             3

                                                            4
A_Sequence        51 ALFLLMYAAQTAARALRPGQLLVATSGPGTSLRTTLATVVALTWLNPHVY     100
                      | :::   |   | :   |:   ||   :| | :  : : |: |||| |
B_Sequence        45 LLPIIIGFKQLVNRKIETVQVQTVTSFENSSYRNSTFSFL-LSLLNPHTY     93
                                                            4

                                                  5
A_Sequence       100 LDTVVLLGTMATPYPAWGRALFAAGGSLAS-----ALWFLLIGL    139
                         | |         :   || ||  |||     |::||::|:
B_Sequence        94 KVAAVTLANGGDNISIY-IPLF-AGSQLASLSIILAVFFLMVGV    135
                                                  5

#---------------------------------------
============ FINISHED =============
Average Quality (AQ)      16.11 +/- 6.25
Standard score (Z):       12.0
Precise score (Z):  12.1
```
