# Supplementary material for: The LysE Superfamily of Transport Proteins Involved in Cell Physiology and Pathogenesis
Source: PLoS One. 2015 Oct 16;10(10):e0137184. doi: 10.1371/journal.pone.0137184 (PMC4608589; doi:10.1371/journal.pone.0137184)
Supplement: S3 Fig — (A) CadD vs. CaCA2. (B) LysE vs. CaCA2. (C) RhtB vs. CaCA2. (PDF) [file pone.0137184.s003.pdf]

Fig. S3A

```
# 1: A_Sequence: Efa1 (2.A.77.1.1 homologue)
# 2: B_Sequence: Ptr2 (2.A.106.1.1 homologue)
# Matrix: EBLOSUM62
# Gap_penalty: 8.0
# Extend_penalty: 2.0

# Length: 115
# Identity:      32/115 (27.8%)
# Similarity:    57/115 (49.6%)
# Gaps:          11/115 ( 9.6%)
# Score: 108.0
#=======================================
                            1                                     2
A_Sequence         1 LQNILSALAVYISTSI-DYLFILLIIFSQNHTKKGLRQIFFGQYLGTGIL     49
                          |::|: |:| | |  | :  : |  |::     :|||  |   ::
B_Sequence         1 WNAFTSSVAMIIATEIGDKTFFIAAVLSMKHSRSA---VFFGAILALIVM     47
                            1                                     2

                                         3
A_Sequence        50 VAISLFAAYVL-NFIPQDWIIGLLGLIPIYLGIRVAF------VGEEEEE     92
                       :|     :| ||||:::   | ||: :| | :: :       |:  ||
B_Sequence        48 TVLSTAMGMMLPNFIPKEYTHLLGGLLFLYFGCKLIYDSRQMEAGKTSEE     97
                                         3

A_Sequence        93 EGEVVEKLGSRGTNR    107
                       || |:|  :|  :
B_Sequence        98 LEEVEEELLQQGKKK    112
#---------------------------------------
============ FINISHED =============
Average Quality (AQ)     15.13 +/- 6.54
Standard score (Z):      14.0
Precise score (Z):  14.2
```

Fig. S3B

```
# 1: A_Sequence: Cac2 (2.A.75.1.1 homologue)
# 2: B_Sequence: Mpu4 (2.A.106.1.1 homologue)
# Matrix: EBLOSUM62
# Gap_penalty: 8.0
# Extend_penalty: 2.0

# Length: 139
# Identity:      38/139 (27.3%)
# Similarity:    62/139 (44.6%)
# Gaps:          16/139 (11.5%)
# Score: 91.0
#=======================================
                            1                               2
A_Sequence         1 LSLIVAVGPQNAMLLKYGIRRDHIGLIIVVCALSDVILITSGTAGVGYLV     50
                     | |:  :| :   |      | |  | : : ::| :  :| |:|  ||||
B_Sequence         1 LILLTEIGDKTFFLAMMLAAR-HGKLQVFLASISALFFMTLGSALAGYLV     49
                            1                              2

                                          3
A_Sequence        51 ----ERFPNALEALKYIGAAYLAFFTFTCFRDAFKTKGEAIDVESTSPNS     96
                         |   :::: : :: |     |      || |   |  | :
B_Sequence        50 STSAEMLHSSVKIMDWVAAVLFVLFGAQMLWDARKLHKE--DAKD-----     92
                                          3
A_Sequence        97 TEEVATFDGDGDSTGGVGTEHGSVATATATQRQEIKRSP    135
                      ||||   | |:  |   : ||  | |  | |:: ::||
B_Sequence        93 -EEVAALLG-GE--GARSSSHGERADAEETLREKDEKSP    127

#---------------------------------------
============ FINISHED =============
Average Quality (AQ)      12.36 +/- 5.83
Standard score (Z):       13.0
Precise score (Z):  13.5
```

Fig. S3C

```
# 1: A_Sequence: Hal1 (2.A.76.1.5 homologue)
# 2: B_Sequence: Cmi1 (2.A.106.1.1 homologue)
# Matrix: EBLOSUM62
# Gap_penalty: 8.0
# Extend_penalty: 2.0

# Length: 166
# Identity:      42/166 (25.3%)
# Similarity:    81/166 (48.8%)
# Gaps:          19/166 (11.4%)
# Score: 103.0
#=======================================
                         2                                 3
A_Sequence         1 LAVFIGDAVLIFCAYIGIASLIRSSPFLFSL-VKMLGALYLLYLGLKILY      49
                     |:|:||  :::    :|   |  |  ||  : :: :||:   : |:|:||
B_Sequence         1 LSVWIGQLLMLLPKLVG-QYLPPSLGFLTHISIEYVGAVLFFFFGIKLLY      49
                         2                                 3

                                                                4
A_Sequence        50 S--TLAKKGQ-----EQSAAKEEPEHTFRKALTL-SLTNPKAILFYVSFF      91
                     |   :::|       |   | |: |  |::  |   :     :| :|: :
B_Sequence        50 SARNMSRKTDIEVMAEAEEAIEDGERKFKQRNTAWKIFIESGVLTFVAEW      99
                                                                4

                                                  5
A_Sequence        92 ---VQFIDMDYAHTGVSFAILAVILEMISFCYMTLLIFSGAALAHFLSEK     138
                         ||  :  | |  |  ::|  :   : | : : :  | |:|  :||
B_Sequence       100 GDRTQFATVTLAATKDSLGVMAGGIVGHAICAL-IAVIGGRAIASHISE-     147
                                                  5

                                   6
A_Sequence       139 KRLAKLGNSMVGLLFL    154
                     : :  :|    ||||:
B_Sequence       148 RTITIIG----GLLFI    159
                                   6
#---------------------------------------
============ FINISHED =============
Average Quality (AQ)      18.42 +/- 6.49
Standard score (Z):       13.0
Precise score (Z):  13.0
```
