# Supplementary material for: The LysE Superfamily of Transport Proteins Involved in Cell Physiology and Pathogenesis
Source: PLoS One. 2015 Oct 16;10(10):e0137184. doi: 10.1371/journal.pone.0137184 (PMC4608589; doi:10.1371/journal.pone.0137184)
Supplement: S4 Fig — (A) RhtB vs. MntP. (B) CadD vs. MntP. (C) CaCA2 vs. MntP. (PDF) [file pone.0137184.s004.pdf]

Fig. S4A

```
# 1: A_Sequence: Kor1 (2.A.76.1.5 homologue)
# 2: B_Sequence: Cce1 (2.A.107.1.2 homologue)
# Matrix: EBLOSUM62
# Gap_penalty: 8.0
# Extend_penalty: 2.0

# Length: 105
# Identity:      26/105 (24.8%)
# Similarity:    58/105 (55.2%)
# Gaps:           9/105 ( 8.6%)
# Score: 80.0
#========================================
                          3                                         4
A_Sequence         1 LYLAYLGINMLRGAWAARRRTAAQAPAQTLSNIHTHDNV-FRHALLLSLS     49
                     : || :||||:: :    | :: :    |:::::| ::: |::  :|:::
B_Sequence         1 ILLAIIGINMIKES----RNSSCEVAVDTVADVNTDNSLSFKNMFVLAVA     46
                          3                                         4

                                                              5
A_Sequence        50 NPKAALFFLSFFIPFVNPRYPHPALSFFILAAVMQTLSMCYLATLALAGD     99
                         ||  :     |:|     ||:||  :  |  ||||  :   :: |:
B_Sequence        47 TSIDAL-AVGITFAFLNVNI-IPAVSF--IGIVTFTLSMIGVRIGSVFGE     92
                                                              5

A_Sequence       100 KLLAK    104
                     |  ::
B_Sequence        93 KFKSR     97
#---------------------------------------
============ FINISHED =============
Average Quality (AQ)      11.53 +/- 4.99
Standard score (Z):       14.0
Precise score (Z):  13.7
```

Fig. S4B

```
# 1: A_Sequence: Hku1 (2.A.77.1.5 homologue)
# 2: B_Sequence: Dvu1 (2.A.107.1.2 homologue)
# Matrix: EBLOSUM62
# Gap_penalty: 8.0
# Extend_penalty: 2.0

# Length: 137
# Identity:      41/137 (29.9%)
# Similarity:    69/137 (50.4%)
# Gaps:          16/137 (11.7%)
# Score: 110.0
#=======================================
                                  3                                  4
A_Sequence         1 -DKWIVGLLGLIPLFIGIKFALSGEDEDETEEIREKIEQDKSKNLLWTVV     49
                      | |:    ||: |:||::  :  |  :||||  ::   | :: |  |::
B_Sequence         1 WDHWLA--FGLL-LYIGVR--MMREAFEETEENDDRC--DPTRGL--TLI     41
                                  3                                  4

                                                           5
A_Sequence        50 LLTIASGGDNLGVYIPYFSSLNWSKIIIVLIIFAIGIAILCELSRSLSKI     99
                     :| :|:  | | | :    ||:   | ||     ||:  |   :  |
B_Sequence        42 MLAVATSIDALAVGL----SLSVLGIDIVTPAIVIGVVCLLFTATGLHLG     87
                                                           5


                                          6
A_Sequence       100 PMVS--EIIEKYEKIIVPVVFIALGIYIMYENGTIQT    134
                      |:|  | : :   :   || | :|: |:||:|   |
B_Sequence        88 RMLSRAESLGRRAALAGGVVLIGIGLRILYEHGVFDT    124
                                          6
#---------------------------------------
============ FINISHED =============
Average Quality (AQ)     14.66 +/- 6.04
Standard score (Z):      16.0
Precise score (Z):  15.7
```

Fig. S4C

```
# 1: A_Sequence: Csp2 (2.A.106.1.1 homologue)
# 2: B_Sequence: Eco2 (2.A.107.1.1 homologue)
# Matrix: EBLOSUM62
# Gap_penalty: 8.0
# Extend_penalty: 2.0

# Length: 152
# Identity:      47/152 (30.9%)
# Similarity:    70/152 (46.1%)
# Gaps:          15/152 ( 9.9%)
# Score: 134.0
#=======================================
                          2                                 3
A_Sequence         1 AALASMTLLSVLMGQAISFLPKHYI----HWAEIALFLGFGLKLIYDASQ     46
                     |   |:  |: |:| ||  : :|||    ||    | |  ||::|| | |
B_Sequence         1 AVFGSVETLTPLIGWAIGSVAQHYIADWDHWIAFTLLLLLGLRMIYGALQ     50
                          2                                 3
                                                             4
A_Sequence        47 MPSQSQGTVIKEAAEAVDQIPQSGNR-----LTKLLARYPQIGIWLQAFS     91
                      | |  |   :::|||  :  ||| |     :   :|    |   :
B_Sequence        51 -PEQPAG---EQSAEAQPESGQSGRRPPSPLMLVAIAFATSIDSMIVGVG     96
                                                             4
                                                   5
A_Sequence        92 MTFLAEWGDRTQISTIALASS-YNVIGVTTGAILGHGICSVIAVIGGKLV    140
                     : || |         | ||::    ||:  |: ||  |     ::|| ::
B_Sequence        97 LAFL-EVNILLTALAIGLATTIMAAIGLRLGSFLGSAIGKRAEILGGLVL    145
                                  5                                 6

A_Sequence       141 AG    142
                      |
B_Sequence       146 IG    147
#---------------------------------------
============ FINISHED =============
Average Quality (AQ)     22.13 +/- 7.43
Standard score (Z): 15.0
Precise score (Z):  15.1
```
