# Supplementary material for: The LysE Superfamily of Transport Proteins Involved in Cell Physiology and Pathogenesis
Source: PLoS One. 2015 Oct 16;10(10):e0137184. doi: 10.1371/journal.pone.0137184 (PMC4608589; doi:10.1371/journal.pone.0137184)
Supplement: S5 Fig — (A) CadD vs. ILT. (B) RhtB vs. ILT. (C) CaCA2 vs. ILT. (PDF) [file pone.0137184.s005.pdf]

Fig. S5A

```
# 1: A_Sequence: Lbr1 (2.A.77.1.1 homologue)
# 2: B_Sequence: Sma2 (2.A.108.2.4 homologue)
# Matrix: EBLOSUM62
# Gap_penalty: 8.0
# Extend_penalty: 2.0

# Length: 207
# Identity:      55/207 (26.6%)
# Similarity:   101/207 (48.8%)
# Gaps:          31/207 (15.0%)
# Score: 138.0
#=======================================
                         1                         2
A_Sequence         1 IDYLIIILMVIFGA--TPKRHRFLVYLGDFLGTAILVLTSYLMAVILGFV-      47
                     :: |:|:: : |     |: | | ::  :|| |: || | : |::| |:
B_Sequence         1 VEALLIVLALIGTLKASKQKRGLKWV--YLGAALGVLASVVTAIMLQFLF      48
                         2                         3

                                          3
A_Sequence        48 PA-------EWLLGFLGLIPILM--GVKLLIFGEKEDDDLIENEIQKKTN      88
                     ||       | | | :|:  : |  || : :   | :    :| ::|: |
B_Sequence        49 PALTSGNNREMLEGAVGIFAVFMMIGVGVWLH-SKANISAWQNYMEKQLN      97
                                          4

                         4                                          5
A_Sequence        89 VILK---------VAIITIATCGADNIGIYVPLFTQISPTN--IPILLVT     127
                     :::          :: : :   ||: |  || :   ||  |  : ||
B_Sequence        98 LVMSTGSFVSMFALSFLAVFREGAETILFYVGILPNISLQNLLLGILAAV     147
                         5                                          6

                                                      6
A_Sequence       128 FFIMMTLFCYLGYLLSKIPTIGNILE--KWSRYITAVVYIGLGIYILWES     175
                       :||  | ::     ||| |  : :   |: || |   :|: |: |  :
B_Sequence       148 LILMMLAFVFI-KSSEKIP-IHRVFQLLTWTIYILAFKMLGVSIHALQLT     195
                                                      7

A_Sequence       176 GTL-THL    181
                       | ||:
B_Sequence       196 NALPTHV    202
#---------------------------------------
============ FINISHED =============
Average Quality (AQ)      27.14 +/- 8.23
Standard score (Z):       13.0
Precise score (Z):  13.5
```

Fig. S5B

```
# 1: A_Sequence: Aau1 (2.A.76.1.5 homologue)
# 2: B_Sequence: Eli1 (2.A.108.2.1 homologue)
# Matrix: EBLOSUM62
# Gap_penalty: 8.0
# Extend_penalty: 2.0

# Length: 104
# Identity:      25/104 (24.0%)
# Similarity:    48/104 (46.2%)
# Gaps:           5/104 ( 4.8%)
# Score: 84.0
#=======================================
                                  4                                    5
A_Sequence         1 KKNESALSMFQRGIWVNLLNPKAIVFFLA-FMPQFIRPDQPLLQQYAVLT      49
                     |  :     |  | |   |   |: :  | ::       :  :: :  |
B_Sequence         1 KAGQDRALRFVHGGWTGALVAGALTWLAATYLLDISGAGRESIEAFGSLI      50
                                  2                                    3
                                                        6
A_Sequence        50 ATVIIIDIMVMWFFFAFAARSFQRFTHDQKGQKVLNRVFGCLFVLVGILL      99
                     | :::: : | |      | ::||:  |: | | |:|  | |: | ||:
B_Sequence        51 AALVLLSVGV-WMHGKSQADNWQRYIRDKLG-KALSR--GSLWFLFGIVF      96
                                                        4

A_Sequence       100 AVIH    103
                      |::
B_Sequence        97 LVVY    100
#---------------------------------------
============ FINISHED =============
Average Quality (AQ)     11.31 +/- 5.29
Standard score (Z):      14.0
Precise score (Z):  13.7
```

Fig. S5C

```
# 1: A_Sequence: Aan1 (2.A.106.1.1 homologue)
# 2: B_Sequence: Sso1 (2.A.108.3.3 homologue)
# Matrix: EBLOSUM62
# Gap_penalty: 8.0
# Extend_penalty: 2.0

# Length: 82
# Identity:      28/82 (34.1%)
# Similarity:    47/82 (57.3%)
# Gaps:           2/82 ( 2.4%)
# Score: 104.0
#=======================================
                     1                     2                         3
A_Sequence         1 IAAILAMKHARLVIFLGAVSALAVMTVLSAAMGYALPALMPRTYTHYASA     50
                     ||||    :   : |: ||  :|:: : :  :|  |  |:|  |   |||
B_Sequence         1 IAAIYHNIYKNNLPFIYAVLGVAIVLIPTFTLG-KLIYLVPLNYVLLASA     49
                     1                     2                         3

A_Sequence        51 LLFFYFGCRMLKDASSMSGSGVSEELGEVEEE     82
                     :: |||| |::: |   |  |: :: || :||
B_Sequence        50 VILFYFGYRLIRSA-RRSFKGIKKKGGEEKEE     80
#---------------------------------------
============ FINISHED =============
Average Quality (AQ)      13.74 +/- 5.89
Standard score (Z):       15.0
Precise score (Z):  15.3
```
