# Supplementary material for: The LysE Superfamily of Transport Proteins Involved in Cell Physiology and Pathogenesis
Source: PLoS One. 2015 Oct 16;10(10):e0137184. doi: 10.1371/journal.pone.0137184 (PMC4608589; doi:10.1371/journal.pone.0137184)
Supplement: S6 Fig — (A) RhtB vs. TerC. (B) CadD vs. TerC. (C) LysE vs. TerC (D) MntP vs. TerC. (E) ILT vs. TerC. (F) CaCA2 vs. TerC. (PDF) [file pone.0137184.s006.pdf]

Fig. S6A

```
# 1: A_Sequence: Pre2 (2.A.76.1.5 homologue)
# 2: B_Sequence: Lfr1 (2.A.109.1.3 homologue)
# Matrix: EBLOSUM62
# Gap_penalty: 8.0
# Extend_penalty: 2.0

# Length: 120
# Identity:      39/120 (32.5%)
# Similarity:    55/120 (45.8%)
# Gaps:          16/120 (13.3%)
# Score: 91.0
#=======================================
                        1                                          2
A_Sequence         1 NFWTYLAGLVLIIIVPGPNSLYVLKTSTS-SGTRFGYRAAL--GVFTGDG     47
                     | |  :  ||:|  :   ::  ||   |    |:     :|  |:: |
B_Sequence         1 NDWLIIFSLVVIECLLSVDNAVVLAAQTQVLPTKKWQEESLFYGMW-GAY     49
                        1                                          2

                                                   3
A_Sequence        48 ILIFLSFIGV-ASVIKASPTLFMIVRYLGAAYLLYLGCKILYSTFM--HK     94
                     |  ||  |||   :||     | |:: |||||| ||     |:     ||
B_Sequence        50 IFRFL-IIGVGVYLIK-----FWIIKVLGAAYLFYLAFSFFYNMHQNRHK     93
                                                   3

A_Sequence        95 KSNQDGTDTISIKTENHFTR    114
                     ||:   |  :    :||  |
B_Sequence        94 KSH---THQVKPNKKNHTRR    110
#---------------------------------------
============ FINISHED =============
Average Quality (AQ)     13.27 +/- 5.74
Standard score (Z):      14.0
Precise score (Z):  13.5
```

Fig. S6B

```
# 1: A_Sequence: Osp1 (2.A.77.1.1 homologue)
# 2: B_Sequence: Bsp1 (2.A.109.1.3 homologue)
# Matrix: EBLOSUM62
# Gap_penalty: 8.0
# Extend_penalty: 2.0

# Length: 118
# Identity:      36/118 (30.5%)
# Similarity:    60/118 (50.8%)
# Gaps:          11/118 ( 9.3%)
# Score: 104.0
#========================================
                         1                                 2
A_Sequence         1 SIDYIVIL--VVLFAQNERRKRAVRDIFLGQYIGFTILIAISLLAAFGLT     48
                     |||   :|  :::  : | ||:|::    | |  |     |||:  |
B_Sequence         1 SIDNAAMLASMIMKLKKEDRKKALKYGIFGAYF-FR---GISLI--FASI     44
                         1                                 2

                                  3                                  4
A_Sequence        49 LIPQHWIGLL-GLVPIFIGLKVLFEKE--DDDDQEEIIDTNRFTSFILSV     95
                     ||   |: || ||  ::||:   |:|:    : :: ||  | |   |:|:
B_Sequence        45 LIKIWWLKLLGGLYLVYIGISHFFKKKLIKKNSKKNIILRNSFWKIIISI     94
                                  3                                  4


A_Sequence        96 AVIMLAAGGDNLGVYIPY    113
                      :: |    ||:   | :
B_Sequence        95 EIMDLTFSIDNIFATIAF    112
#----------------------------------------
============ FINISHED =============
Average Quality (AQ)     16.41 +/- 6.46
Standard score (Z):      14.0
Precise score (Z):  13.6
```

Fig. S6C

```
# 1: A_Sequence: Pfr1 (2.A.75.1.1 homologue)
# 2: B_Sequence: Rpa3 (2.A.109.1.5 homologue)
# Matrix: EBLOSUM62
# Gap_penalty: 8.0
# Extend_penalty: 2.0

# Length: 103
# Identity:      31/103 (30.1%)
# Similarity:    51/103 (49.5%)
# Gaps:          11/103 (10.7%)
# Score: 112.0
#=======================================
                     3                                            4
A_Sequence         1 YLCWFAWRSFRSALRPQSD--DALTGQGPDAGALRPIVGTTL-ALTWLNP     47
                     ::||  ||  ||  :  :|  |||   |  :|| |  :|  :  :|  :
B_Sequence         1 WVCWKMWRELRSQSQHDADALDALNDDGTASGAPRKTLGQAVWQITLADI     50
                     3                                            4
                                                 5
A_Sequence        48 HVYLDTMVMLGGLANQHPGLTRWAFAGGAMLGSALWFAALGLGARALSRP     97
                      : || :: : | | :|| :         : | ||  | :|| |  :::
B_Sequence        51 SMSLDNVLAVAGAAREHPII--------LVFGLALSIALMGLAASFIAKL     92
                                                 5

A_Sequence        98 LSK    100
                     | |
B_Sequence        93 LQK     95
#---------------------------------------
============ FINISHED =============
Average Quality (AQ)      16.06 +/- 6.58
Standard score (Z):       15.0
Precise score (Z):  14.6
```

Fig. S6D

```
# 1: A_Sequence: Lmi1 (2.A.107.1.1 homologue)
# 2: B_Sequence: Ddo1 (2.A.109.5.1 homologue)
# Matrix: EBLOSUM62
# Gap_penalty: 8.0
# Extend_penalty: 2.0

# Length: 188
# Identity:      48/188 (25.5%)
# Similarity:    94/188 (50.0%)
# Gaps:          29/188 (15.4%)
# Score: 132.0
#=======================================

                         2                                 3
A_Sequence         1 LSQALGIGILFGV---VEATTPLIGWLLGSAASRFVASIDHWVAFVLLAG     47
                     |:  : | :|||:   |  : |   | :   ||     |  | | :|:||
B_Sequence         1 LAMGMRIALLFGISWLVALSAPF--WHIN--ASWITGGIS-WQAVILIAG     45
                         2                                 3

A_Sequence        48 LGIHMVWKSFQPLEPDCDDQTDAPYDTGVQLGADGSALRTGRLLPAGLLS     97
                      || ::|||   :    |       :||::   :  : ::   |   ::
B_Sequence        46 -GIFLIWKSVHEIHEKVD-------ETGLE--EEEISKKSSTTLGNAIVQ     85

                         4                                 5
A_Sequence        98 MLLTSVATSIDAM--AVGVTLAFVDVPIGQVALVIGLCTTMMVTLGVML-    144
                     : : ::  | |::  |||:|    | |    ||:| :   :::::|:|:
B_Sequence        86 IAVINLVFSFDSILTAVGMTNGLSDNPTD--ALII-MVIAVVISVGIMML    132
                         4                                 5
                                          6
A_Sequence       145 -GRLLGTLVGRR--AEMLGGIVLIVIGTVILYE--HLA    177
                         :|  : :    ::||   ||:|| ::: |  ||:
B_Sequence       133 FANPVGNFIAKHPSLQILGLSFLILIGFMLIAEGAHLS    170
                                          6
#---------------------------------------
============ FINISHED =============
Average Quality (AQ)      26.22 +/- 7.83
Standard score (Z):       14.0
Precise score (Z):  13.5
```

Fig. S6E

```
# 1: A_Sequence: Npe1 (2.A.108.2.1 homologue)
# 2: B_Sequence: Cte1 (2.A.109.1.3 homologue)
# Matrix: EBLOSUM62
# Gap_penalty: 8.0
# Extend_penalty: 2.0

# Length: 202
# Identity:      57/202 (28.2%)
# Similarity:    91/202 (45.0%)
# Gaps:          19/202 ( 9.4%)
# Score: 125.0
#=======================================
                                                                    2
A_Sequence         1 SSFVAAFTILVREGLEAI---LIVIAMITFLAKADRRDVLPYVHGGWIAA      47
                     || :  | ::| ||| ::    ::  |:  | :  |   | |   | : |
B_Sequence         1 SSLLVIFNLIVIEGLLSVDNAAVLATMVLDLPQKQRPAALTY---GILGA      47
                                  1                                 2

                                                   3
A_Sequence        48 -LFAGAGTWAAATWLITISGASRELTEGFGGVFAALVLLWVGIWMH-GKS      95
                      || |   : || :|:     |      |||::  | |:|   | : |
B_Sequence        48 YLFRGLFLFFAA-FLV-----SAWWLRPFGGLY-LLYLVW-NWWNNRGSK      89
                                                   3

                                                             4
A_Sequence        96 NADAWQRYIRD-KLGRALNRRSAWFLFALAFIVVYREVFETILFYAAIWS     144
                     : ||     || :| | ::||   |   : |: :    |     :||:
B_Sequence        90 DGDAMCTEKRDNRLYRFVSRRIGPFWATVLFVEMMDIAFSIDNVFAAVAF     139
                                                             4

                                  5                                 6
A_Sequence       145 QGNGGAVVAGAFAAIAVLAVIAFVMLRHSRTLPIGKFFAYSSALIAVLAV     194
                       |   |  | |  | |:  :|:  :|     |  :  ||   ::||| :
B_Sequence       140 TDNLILVCTGVFIGILVMRFVAYGFIRLMEEYPFLESCAY--IVLAVLGL     187
                                  5                                 6


A_Sequence       195 VL    196
                      |
B_Sequence       188 RL    189
#---------------------------------------
============ FINISHED =============
Average Quality (AQ)      22.32 +/- 7.82
Standard score (Z):       13.0
Precise score (Z):  13.1
```

Fig. S6F

```
# 1: A_Sequence: Ptr2 (2.A.106.1.1 homologue)
# 2: B_Sequence: Lga1 (2.A.109.1.3 homologue)
# Matrix: EBLOSUM62
# Gap_penalty: 8.0
# Extend_penalty: 2.0

# Length: 194
# Identity:      53/194 (27.3%)
# Similarity:    85/194 (43.8%)
# Gaps:          31/194 (16.0%)
# Score: 111.0
#=======================================
                                  1                                 2
A_Sequence         1 GGFWNAFTSSVAMIIATEIGDKTFFIAA---VLSMK-HSRSAVFFGAILA      46
                     |  |    : : |     : |    :||   ||  |   | :: :| :
B_Sequence         1 GQDWMMILTLILMECLLSV-DNAVVLAAQTQVLPTKDEQRKSLVYG-LWG      48
                                  1                                 2

                                                   3
A_Sequence        47 LIVMTVLSTAMGMMLPNFIPKEYTHLLGGLLFLYFGCKLIYDSRQMEAGK      96
                       :   :   :|  | ||       |||||  ||   |  || |
B_Sequence        49 AYLFRFIVIGIGTYLINFWE---IKLLGGLYLLYLVYKYFYDVRHP----      91
                                                   3

                                                                       4
A_Sequence        97 TSEELEEVEEELLQQGKKKADLEEGSRSNRPPSKKQMGWNQVV-IQSLTL     145
                           :| ::  :  ||:|  :: |:: :      : |  |: |:|: :
B_Sequence        92 -----AQVAKK--EAAKKEAHKKKNSKTRK--HHLSLFWRTVISIESMDI     132
                                                                       4

                                                   5
A_Sequence       146 TFVAEWGDRSQIATIALAASKNPIGVTIGGCVGHSLC-TGLAVV     188
                      |  :    | :|  ||| | ||: | :|| :|  ||  |:| |
B_Sequence       133 VFSID----SVLA--ALAMSNNPVVVLVGGMIG-ILCMRGVAEV     169
                                                   5

#---------------------------------------
============ FINISHED ============
Average Quality (AQ)      13.76 +/- 5.99
Standard score (Z):       16.0
Precise score (Z):   16.2
```
