# Supplementary material for: The LysE Superfamily of Transport Proteins Involved in Cell Physiology and Pathogenesis
Source: PLoS One. 2015 Oct 16;10(10):e0137184. doi: 10.1371/journal.pone.0137184 (PMC4608589; doi:10.1371/journal.pone.0137184)
Supplement: S7 Fig — (A) LysE vs. NAAT. (B) RhtB vs. NAAT. (C) CadD vs. NAAT (D) MntP vs. NAAT. (E) TerC vs. NAAT. (PDF) [file pone.0137184.s007.pdf]

Fig. S7A

```
# 1: A_Sequence: Spl1 (2.A.75.1.1 homologue)
# 2: B_Sequence: Ogr1 (2.A.95.1.3 homologue)
# Matrix: EBLOSUM62
# Gap_penalty: 8.0
# Extend_penalty: 2.0

# Length: 157
# Identity:      50/157 (31.8%)
# Similarity:    74/157 (47.1%)
# Gaps:          14/157 ( 8.9%)
# Score: 124.0
#=======================================
                         1                         2
A_Sequence         1 LIVAIGAQNTFV-LTQGIRKQHRFVVVALICSL-CDAFLISAGVAG--LGS     46
                     :|  ||    || ||||:  : |  :|:  :|   | |::  | |  |
B_Sequence         1 IIDPIGLTPLFVALTQGMPDRQRRAIAVRATLVAVAVLLAFAVFGEALLG     50
                         1                         2
                                  3
A_Sequence        47 LIEQSPTLLRLAGGGGALFLFIYGLKCLFSALQAEQELGETESNPTSRRQ     96
                      :  |    |:|||   : ||:  |  ||   || :|  :|  :||
B_Sequence        51 FVGISMAAFRIAGG---VLLFLTALDMLFQRRQARRE--DTADDPTEDPS     95
                                  3
                          4                                         5
A_Sequence        97 VILTILAI-TLCNPNVYLDTVVLLGGISATFVGQGRYLFGAGAISMSFIW    145
                     |    |||  |  |   : |::|| | | :  |    | |     :: ::
B_Sequence        96 VF--PLAIPLLAGPGA-IATIILLTGQSESVAGFAAVL-GVMVAVLTIVF    141
                          4                                         5

A_Sequence       146 FFILSYG    152
                      | |: |
B_Sequence       142 LFFLAAG    148
#---------------------------------------
============ FINISHED =============
Average Quality (AQ)     20.31 +/- 7.39
Standard score (Z):      14.0
Precise score (Z):  14.0
```

Fig. S7B

```
# 1: A_Sequence = Pag1 (2.A.76.1.2 homologue)
# 2: B_Sequence = Cba1 (2.A.95.1.4 homologue)
# Matrix: EBLOSUM62
# Gap_penalty: 8.0
# Extend_penalty: 2.0

# Length: 167
# Identity:      44/167 (26.3%)
# Similarity:    79/167 (47.3%)
# Gaps:          15/167 ( 9.0%)
# Score: 115.0
#=======================================
                          1                                 2
A_Sequence         1 ALVHLVALMSP-GPDFFFVS-QTAASRSRKEAMMGVLGITLGIVVWAGV-     47
                     : : |:||::| |   ||:|  |  :   |   : :  |::  ||  |:
B_Sequence         1 SFISLLALINPIGAIPFFISLTTQQTEEEKRHTIKIAAISVATVV--GIS     48
                          1                                 2
                                           3
A_Sequence        48 ALMGLHLILEKMAWLHQVIMVGGGLYLLWMGWQLMCSARQRHKPQQDEP     97
                     ||:|   |:|        : ||||| :: |   :: :   | |   ::|
B_Sequence        49 ALLG-QQIIEFFNISVASLQVGGGLIMIMMAMNMLNAQTSRTKATPEEED     97
                                           3
                                    4
A_Sequence        98 VVELPKRGMSFLKGLLTNLSNPKAIIYFGSVFSLFVGDDVGSAERWGLFL    147
                       |  |  :: :   |  |: |      ||: :: |    |  : |   |
B_Sequence        98 EAE-AKASIAVVPLALPLLTGP------GSISTVIV--YAGKTQHWYQLL    138
                                    4
                          5
A_Sequence       148 LIIGETFAWFALVAAIF    164
                     :::|   |  |:|  :|
B_Sequence       139 ILVGIGVALGAVVYIVF    155
                          5
#---------------------------------------
============ FINISHED =============
Average Quality (AQ)      17.31 +/- 6.50
Standard score (Z):       15.0
Precise score (Z):  15.0
```

Fig. S7C

```
# 1: A_Sequence: Msp1 (2.A.77.1.4 homologue)
# 2: B_Sequence: Orf7 (2.A.95.1.4 homologue)
# Matrix: EBLOSUM62
# Gap_penalty: 8.0
# Extend_penalty: 2.0

# Length: 152
# Identity:      42/152 (27.6%)
# Similarity:    68/152 (44.7%)
# Gaps:           9/152 ( 5.9%)
# Score: 97.0
#=======================================
                     1                             2
A_Sequence         1 FLLAAFFANPEFRAKDVVLGQYLGFIVLLT--ISSLAYFVQF--IIPSNW     46
                     ||      ||: | |         |:|| |  |:    |  |   :|:
B_Sequence         1 FLAVTTGQNPQKRRKTARKASLTAFVVLTTFAIAGTFIFKMFGITLPAFE     50
                     1                             2

                         3                                          4
A_Sequence        47 ISLLGVIPIMIGIRSFLHLKK-PQTDYSGENRDFSKYKEGQMMLPVTLVT     95
                     |:  ||| ::||:   |  |: |  : |||  : :  ||   ::|: :
B_Sequence        51 IA-GGVILLLIGL-DMLEAKRSPTQESSGETAEAAS-KEDVGIVPLGIPM     97
                         3                                          4

                                                   5
A_Sequence        96 LANGGDNLGVYMPLFASMGPFDL-FLTAIIFLIMVGVWCFLGYKLVNNRV    144
                     ||  |    | : :  :  |: :  : | | :  |  :  ||      |:
B_Sequence        98 LAGPGAITSVMVLVGQAQNPWQVGTIIAAIAITAVSCYVVLGAATRVARI    147
                                                   5

A_Sequence       145 LG    146
                     ||
B_Sequence       148 LG    149
#---------------------------------------
============ FINISHED =============
Average Quality (AQ)      13.03 +/- 5.85
Standard score (Z):       14.0
Precise score (Z):  14.4
```

Fig. S7D

```
# 1: A_Sequence: Asu1(2.A.107.2.1 homologue)
# 2: B_Sequence: Csh1(2.A.95.1.5 homologue)
# Matrix: EBLOSUM62
# Gap_penalty: 8.0
# Extend_penalty: 2.0

# Length: 124
# Identity:      33/124 (26.6%)
# Similarity:    64/124 (51.6%)
# Gaps:           6/124 ( 4.8%)
# Score: 108.0
#=======================================
                                  3                                4
A_Sequence         1 YISEFDHWIA-FALLCVIGINMIKMSVTNENSDDDPSD--FSL-RHLTML     46
                     ::||    ||   :| :| : |:     : |  | |:|  | :   : ::
B_Sequence         1 HLSETSLGIAGGVILFLIALRMV-FPAPHGNGADHPADEPFVVPLAIPLI     49
                                  3                                4
                                                  5
A_Sequence        47 GVATSIDALAMGVSFAFLKVNIWTAAAIIGITTTILSL-FGVKAGHWLGD     95
                        :::  : : ||    ::   | ||  : :    |:| |  |  ||||:
B_Sequence        50 AGPSALATVLLLVSREPARLWEWVAALALTMVVCALTLAFAEKISHWLGE     99
                                                  5
                                  6
A_Sequence        96 RIHKQAELLGGIILIAMGVKVLIE    119
                     |:    | | |::| |: |::|::
B_Sequence       100 RVTTAFERLMGLVLTAIAVQMLLD    123
                                  6
#---------------------------------------
============ FINISHED ============
Average Quality (AQ)     16.70 +/- 6.06
Standard score (Z):      15.0
Precise score (Z):  15.1
```

Fig. S7E

```
# 1: A_Sequence: Gka1 (2.A.109.1.5 homologue)
# 2: B_Sequence: Dgi1 (2.A.95.1.5 homologue)
# Matrix: EBLOSUM62
# Gap_penalty: 8.0
# Extend_penalty: 2.0

# Length: 106
# Identity:      29/106 (27.4%)
# Similarity:    52/106 (49.1%)
# Gaps:           6/106 ( 5.7%)
# Score: 93.0
#=======================================
                         1                                 2
A_Sequence         1 LIIGIDVILGGDNAVVIALAS-RNLPEQKRNVAIIVGTALAIAVRIVLTV     49
                     | : : :|: |   | : ::  |  | :::   |      |:|: |:
B_Sequence         1 LAVPLFLIMDGLGNVPVCMSMLRRFPPRRQQRIIFRELCFALAISILFCF     50
                         1                                 2
                                          3
A_Sequence        50 AVVWLLTI----P-FLQLAGGVVLFWIALKLIGQKDEKPTMIKAEPSLWK     94
                        |||      |  |:|||||||| |:::::   : | |    :||
B_Sequence        51 FGDWLLKFLGLGPSTLRLAGGVVLFVISMRMVFPDESKETADPEDPSALA    100
                                          3
                         4
A_Sequence        95 AIQTIV    100
                     | :  :
B_Sequence       101 AEEPFI    106
                         4
#---------------------------------------
============ FINISHED =============
Average Quality (AQ)      12.39 +/- 5.29
Standard score (Z):       15.0
Precise score (Z):  15.2
```
