# Supplementary material for: The LysE Superfamily of Transport Proteins Involved in Cell Physiology and Pathogenesis
Source: PLoS One. 2015 Oct 16;10(10):e0137184. doi: 10.1371/journal.pone.0137184 (PMC4608589; doi:10.1371/journal.pone.0137184)
Supplement: S8 Fig — (A) RhtB vs. NicO. (B) CadD vs. NicO. (C) TerC vs. NicO (D) NAAT vs. NicO. (PDF) [file pone.0137184.s008.pdf]

Fig. S8A

```
# 1: A_Sequence: Aur1 (2.A.76.1.2 homologue)
# 2: B_Sequence: Bco1 (2.A.113.1.9 homologue)
# Matrix: EBLOSUM62
# Gap_penalty: 8.0
# Extend_penalty: 2.0

# Length: 209
# Identity:      52/209 (24.9%)
# Similarity:    93/209 (44.5%)
# Gaps:          18/209 ( 8.6%)
# Score: 128.0
#=======================================
                         1                                          2
A_Sequence         1 FIALITLMFIQFCALITPGPDFFLVSQTAISRSRREAVFVVLGITVGVMF     50
                     ||::: | |:         ||  :   |  |||:: :   : |:  |:  
B_Sequence         1 FISVLALGFVLGIKHAIE-PDHIIAVSTIASRSKKLSQSSLAGVFWGIGH     49
                         1                                          2

                                                   3
A_Sequence        51 WAILALMGLNIIFEK----MAWLKQILLVIGGIYLCWLGFQMLRSAFSKQ     96
                      | |::|: ::  |      |   :  :: || | :||   | ||| :
B_Sequence        50 TATLFIVGICLLIIKGEIPEKWAMSLEFLV-GIMLVYLGITTL-SAFKRV     97
                                                   3

                                                           4
A_Sequence        97 KVQNTNTPIDLPKTETKF-FLKGLLTNLSNPKAVIYFGS-VFSLFLANPA    144
                     ::   |     |  :  : ::| :     :  |    ||    |   :
B_Sequence        98 RI---NHHYHEPGHKRNYSYIKSVCIGFVHGLA----GSGAMVLLTMSTV    140
                                                           4

                                          5
A_Sequence       145 LDHVHSLLFIII-AVETLIWFLFVVFVFSLPSFKSAYQ-NVAKWIDGVSG    192
                        | | ::|:|  : |:   ||   :  :|   || :  | | :  ::|
B_Sequence       141 KSVVESAIYILIFGIGTIFGMLFFTTILGIPFIISAKKVEVNKTLTQITG    190
                                          5

                           6
A_Sequence       193 GIFTAFGIY     201
                      | | ||||
B_Sequence       191 AISTVFGIY     199
                           6
#---------------------------------------
============ FINISHED ============
Average Quality (AQ)      23.09 +/- 7.61
Standard score (Z):       14.0
Precise score (Z):  13.8
```

Fig. S8B

```
# 1: A_Sequence = Acy3 (2.A.77.1.4 homologue)
# 2: B_Sequence = Gar1 (2.A.113.1.9 homologue)
# Matrix: EBLOSUM62
# Gap_penalty: 8.0
# Extend_penalty: 2.0

# Length: 195
# Identity:      55/195 (28.2%)
# Similarity:    94/195 (48.2%)
# Gaps:          23/195 (11.8%)
# Score: 129.0
#=======================================
                           1                                        2
A_Sequence         1 NIITPILTGV-FAFI-ATNIDDIVILLVFFSQVNEN--FRPWQIVMGQYL      46
                     : :  :|||: |  | | ::| || :  |  | |:|     :    |
B_Sequence         1 DFLAAVLTGIMFGIIHAFDVDHIVAMATFSEQKNKNKQILTYAFKWGTGH      50
                           1                                        2

                                                           3
A_Sequence        47 GFTILVIFSLPGFFGGLILPPAWIG----LLGLIPIGIGISSLVNKEKEQ      92
                     |  ||::  :   | |  ||  ::     ::|:: | :|:  ||   ::
B_Sequence        51 G-GILLLLGMLLIFIGFQLPNWFVHYSEIMVGVLLIYLGVKLLVLLHRKG      99
                                                           3

                                                   4
A_Sequence        93 LADVPEEIISPATSINNYSLTPQIYTVAAITVANGSDNISIYIPLFSSIS     142
                        ||| :   | |:| :  || ::    | : :|    :  : |  ::
B_Sequence       100 TFSVPESLDLAARSLNKHDHTP-LF----IGMLHGVAGSAPLLALLPNML     144
                                                   4

                                  5                             6
A_Sequence       143 FNSFLLIIGLFFF--LLGVWC--YV--AYQL-THQK--KVADFFT    178
                        ||| | ||    | |::|  |:  :||:   ||  |:|  ||
B_Sequence       145 ETQFLLHISLFSIGCLFGMFCFGYIFGSYQVYIKQKKEKLAKAFT    189
                                  5                             6

#---------------------------------------
============ FINISHED =============
Average Quality (AQ)     18.67 +/- 7.32
Standard score (Z):      15.0
Precise score (Z):  15.1
```

Fig. S8C

```
# 1: A_Sequence: Bdi1 (2.A.109.1.5 homologue)
# 2: B_Sequence: Cul1 (2.A.113.1.9 homologue)
# Matrix: EBLOSUM62
# Gap_penalty: 8.0
# Extend_penalty: 2.0

# Length: 196
# Identity:      55/196 (28.1%)
# Similarity:    83/196 (42.3%)
# Gaps:          19/196 ( 9.7%)
# Score: 117.0
#=======================================
                                  1                        2
A_Sequence         1 ASALGKVLMIDLVLAGDNAVAVGLAAAALPQEQRRKAILIGLA-----AA      45
                     |  :| :| :   |  |: |||  :  || :    :   ||       |
B_Sequence         1 AMGIGILLGLRHALDADHVVAV--STMALEERNLLRGGWIGFCWGVGHAL      48
                                  1                        2

                                          3
A_Sequence        46 VVMRIGLALIT--VQLLAIVGLLLAG--GFLLLWVCWKMWRELREQATHD      91
                     |:   | |||   ::|  :||  | |  | :|: :    || :|    |
B_Sequence        49 VLFLFGGALILSGIRLPEVVGRWLEGGVGVMLILIALGSWRRMRRSKLHI      98
                                          3

                                                                4
A_Sequence        92 QAEAEAEIERAMAIEHGGGPSPEEL----GLK-RKTFGAALIQIMIADLT     136
                         : : ||     |    ||  |    | |   :|    :  :
B_Sequence        99 HVH-QHDGERYHTHFHVHDDSPRHLEKHHGWKGSHSFLIGTVHGLAGTGA     147
                                                                4

                                          5
A_Sequence       137 MSLDNVLAVAGASHEHPWIMVFGL--ILSIALMGLAATFIAKLLNR    180
                     : :  : ||:       ::  |||  |||: |  |: | | |||||
B_Sequence       148 VMVLTIAAVSDPLQRIAYLASFGLGTILSMTLFSLSLTLITKLLNR    193
                                          5

#---------------------------------------
============ FINISHED =============
Average Quality (AQ)      18.97 +/- 7.06
Standard score (Z):       14.0
Precise score (Z):  13.9
```

Fig. S8D

```
# 1: A_Sequence: Mfo1 (2.A.95.1.4 homologue)
# 2: B_Sequence: Orf5 (2.A.113.2.1 homologue)
# Matrix: EBLOSUM62
# Gap_penalty: 8.0
# Extend_penalty: 2.0

# Length: 128
# Identity:      33/128 (25.8%)
# Similarity:    66/128 (51.6%)
# Gaps:          11/128 ( 8.6%)
# Score: 109.0
#=======================================
                                                                    4
A_Sequence         1 FKIAWDMLHAEMSKTKHSPREEIDMRMGSVAVVPLAIPLLAGPGAITTTI      50
                     :: | :  |||  : : : ||   :  | : :  |   |:  |||||  :
B_Sequence         1 YQDAHERQHAEDIRRRFAGRE---VTTGQIILFGLTGGLIPCPGAITVLL      47
                                                                    4

                                                   5
A_Sequence        51 ILME-KAQSLANKTIVISSI--ILTMIVSGLILSASDIVVKKLKVSGINA      97
                     : :: |  :| :  ::  ||   |||: || :::|  :   : : ||  :
B_Sequence        48 LCLQLKRVALGSVLVLCFSIGLALTMVASG-VIAALSVKYAERRFSGFGS      96
                                                   5

                                           6
A_Sequence        98 IVR----IMGLILAAISVQIIFSGAYGL    121
                     :||      ||::  : : :  || : |
B_Sequence        97 LVRKAPYASGLVILCVGLYVALSGWHSL    124
                                           6

#---------------------------------------
============ FINISHED =============
Average Quality (AQ)     18.86 +/- 6.66
Standard score (Z):      14.0
Precise score (Z):  13.5
```
