# Supplementary material for: The LysE Superfamily of Transport Proteins Involved in Cell Physiology and Pathogenesis
Source: PLoS One. 2015 Oct 16;10(10):e0137184. doi: 10.1371/journal.pone.0137184 (PMC4608589; doi:10.1371/journal.pone.0137184)
Supplement: S9 Fig — (A) RhtB vs. GAP. (PDF) [file pone.0137184.s009.pdf]

Fig. S9A

```
# 1: A_Sequence: Hgr1 (2.A.76.1.5 homologue)
# 2: B_Sequence: Ssp3 (2.A.116.1.7 homologue)
# Matrix: EBLOSUM62
# Gap_penalty: 8.0
# Extend_penalty: 2.0

# Length: 188
# Identity:      60/188 (31.9%)
# Similarity:    91/188 (48.4%)
# Gaps:          27/188 (14.4%)
# Score: 138.0
#=======================================
                         1                                 2
A_Sequence         1 IGIVLLPGPNSLFVLSVATA-RGVRVGYHAACGVF----LGDSILL-LFT     44
                     : | :: ||  :  : :||| | |||      ||     || :|:| : |
B_Sequence         1 LAITMMAGPQIMSAVILATAQRAVRVSLGFVTGVLIATSLGVAIMLGIAT     50
                         1                                 2

                                          3
A_Sequence        45 ALGAA---SLLRGYPALFMVVKYVGAAYLFWVGMNLAWSAWRKWRAAGIA     91
                     ||| |          ::  |::||  | |      ||  | | ||
B_Sequence        51 ALGGAVDFGSSGDKSSVGRVIQYVLVALLI-----LA--ALRNWR----K     89
                                          3

                                                           4
A_Sequence        92 TQLVEPTA-LAAAQSAHLLAPFQRALVISLLNPKAILFLLSFFVQFIDPA    140
                      : |||   | |  ||     |:  |:: || |  :: :|:  |  :|
B_Sequence        90 RETVEPPKWLHALMSADTRKAFETGLLVVLLMPSDLMVMLTVGVH-LDQG    138
                                                           4

                                          5
A_Sequence       141 YDT--PAIPFLILSVIVMAFSAVYLSVLIVAGARLADA    176
                     : :   |:||: |: :| | : : | ||:  | | | |
B_Sequence       139 HSSFVDALPFIALTTLVAA-TPLLLRVLL--GRRAASA    173
                                          5
#---------------------------------------
============ FINISHED =============
Average Quality (AQ)      26.84 +/- 7.65
Standard score (Z):       15.0
Precise score (Z):   14.5
```
