# Supplementary material for: The LysE Superfamily of Transport Proteins Involved in Cell Physiology and Pathogenesis
Source: PLoS One. 2015 Oct 16;10(10):e0137184. doi: 10.1371/journal.pone.0137184 (PMC4608589; doi:10.1371/journal.pone.0137184)
Supplement: S10 Fig — (A) RhtB vs. DsbD. (B) CaCA2 vs. DsbD. (C) MntP vs. DsbD. (D) NAAT vs. DsbD. (E) GAP vs. DsbD. (PDF) [file pone.0137184.s010.pdf]

Fig. S10A

```
# 1: A_Sequence: Btr2 (2.A.76.1.2 homologue)
# 2: B_Sequence: Cba1 (5.A.1.2.1 homologue)
# Matrix: EBLOSUM62
# Gap_penalty: 8.0
# Extend_penalty: 2.0

# Length: 196
# Identity:      52/196 (26.5%)
# Similarity:    90/196 (45.9%)
# Gaps:          24/196 (12.2%)
# Score: 134.0
#=======================================
                                                2
A_Sequence         1 YVCRKAMADSRRNAMLGALGIALG--VGFWAVIVLFGLT--FLNHTIPNF     46
                     |:    : :  ::  | ||   ||  :||  : ::||:   |:      :
B_Sequence         1 YITGTTLEEELQDKKLFALSRTLGFVLGFTIIFMIFGILAGFVGQAFIRY     50
                                                2

                                   3
A_Sequence        47 QFYLMLLGGSYLAYCGIKMVQVRKSVEIDENLKSQANEKSPL----W-KE     91
                     :  |  :||  :   |: || : |     | |  | | :||     |
B_Sequence        51 RNVLTKIGGIIIVLFGLNMVGLLKL----EFLNKQRNVRSPKEVKNWFSS     96
                                   3

                         4                                         5
A_Sequence        92 ILGGLAINLS-NPKVVVFFSSVL--AGYVANISAFKDILAVLAILMGSTL    138
                     || |:|      | :     ::|   |  | :|  | |: :||  :|
B_Sequence        97 ILMGMAFAAGWTPCIGPVLGTILIYVGTTATVS--KGIILLLAYSIG-LA    143
                         4                                         5

                                                          6
A_Sequence       139 IWFWTVAILFSQNKIRRFYAKNNR---YLDNAAGVVFILFGLKLIY    181
                     | |   |:|   |:  :|  |: :   |:   :||| |: |: :::
B_Sequence       144 IPFLLTALLI--NQFSKFLMKSEKVLPYIVKISGVVIIVVGVLIVF    187
                                                          6

#---------------------------------------

============ FINISHED =============
Average Quality (AQ)      23.74 +/- 7.86
Standard score (Z):       14.0
Precise score (Z):  14.0
```

Fig. S10B

```
# 1: A_Sequence = Sne3 (2.A.106.1.2 homologue)
# 2: B_Sequence = Orf5 (5.A.1.2.1 homologue)
# Matrix: EBLOSUM62
# Gap_penalty: 8.0
# Extend_penalty: 2.0

# Length: 161
# Identity:      52/161 (32.3%)
# Similarity:    82/161 (50.9%)
# Gaps:          22/161 (13.7%)
# Score: 130.0
#=======================================



                          1                                         2
A_Sequence         1 LLGIILAFLIVDGIAILAGEWITDIAPRELIKMLSGAIFI-IFGLVTLIF     49
                     |:|  : |: :   : | | :      ::|:: : ||||| ||||: | |
B_Sequence         1 LVGFSVIFIFLGYSSSLVGTFFYQY--QDLLRQI-GAIFIVIFGLMILGF     47
                          2                                         3

                                                   3
A_Sequence        50 RNKREEIK-TKYHFEN-P--FYSGFI--LIFVSEWGDKTQIATG---LFA     90
                        :  :|  |  |:| |  ::  |:  | | : |   |   ||   : |
B_Sequence        48 FTPKFLMKEKKLQFKNRPAGYFGTFLIGLAFAAGWTPCTGPITGAVFMMA     97
                                                   4

                                           4
A_Sequence        91 TQYNG------LMVLTGVIIALSLLSVIAIYSGKFISDKVTRETLTKLTG    134
                      |  |      |: : |  |   |||:  |   |:|  |  | |:||: |
B_Sequence        98 AQNPGSGMWYMLVYVLGFAIPFFLLSIF-ITRVKWI-QKYNR-TITKVGG    144
                                  5

                          5
A_Sequence       135 FLFISMGVLFF    145
                     :| |::|:| |
B_Sequence       145 YLMIALGILLF    155
                          6
#---------------------------------------
============ FINISHED =============
Average Quality (AQ)      26.20 +/- 7.85
Standard score (Z):       13.0
Precise score (Z):  13.2
```

Fig. S10C

```
# 1: A_Sequence: Cac1 (2.A.107.2.1 homologue)
# 2: B_Sequence: Dsp2 (5.A.1.2.1 homologue)
# Matrix: EBLOSUM62
# Gap_penalty: 8.0
# Extend_penalty: 2.0

# Length: 154
# Identity:      47/154 (30.5%)
# Similarity:    79/154 (51.3%)
# Gaps:          14/154 ( 9.1%)
# Score: 146.0
#=======================================
                         2                                 3
A_Sequence         1 FAISFGFFQFLCTFIGAYSGFLFNTYITYVPQIIGGMIIAFVGAFM---I     47
                     | : |    |   |  :: |  |: |   : :::||::|| :| ||   |
B_Sequence         1 FILGFSIIFFALGFSASWVGSFFSEYRDLI-RMLGGVLIAVMGLFMLGLI     49
                         2                                 3

                                                   4
A_Sequence        48 KEGFDNKEEKLLLNFKMYFVLGISVSIDAAVVGFT-MFNKISSNYVILGD     96
                     | ||  ||::| :  | :  || ||   |   |:|     |  : : |
B_Sequence        50 KPGFMMKEKRLEVGRKRWGYLGSSVIGMAFAAGWTPCVGPILVSVLALAA     99
                                                   4

                                  5                                 6
A_Sequence        97 S------VFIGIVTLILSIIAFIISRYLKRIQLVCKYADYI---GGIILV    137
                     |       :|   ||  :|  ||:: :| | : : ||:: :   || ::|
B_Sequence       100 SNPSAGLAYITAYTLGFAIPFFIMAFFLGRTRWILKYSNSLMKAGGALMV    149
                                  5                                 6


A_Sequence       138 IFGL    141
                     :||:
B_Sequence       150 VFGV    153

#---------------------------------------
============ FINISHED ============
Average Quality (AQ)      28.64 +/- 7.96
Standard score (Z):       15.0
Precise score (Z):  14.7
```

Fig. S10D

```
# 1: A_Sequence: Pfu1 (2.A.95.1.4 homologue)
# 2: B_Sequence: Dto1 (5.A.1.2.1 homologue)
# Matrix: EBLOSUM62
# Gap_penalty: 8.0
# Extend_penalty: 2.0

# Length: 213
# Identity:      57/213 (26.8%)
# Similarity:   104/213 (48.8%)
# Gaps:          25/213 (11.7%)
# Score: 140.0
#=======================================
                        1                                          2
A_Sequence        1 GLFAITNP--IGAVPVFIAVTRNLSPEKRKEIARKTSTTVMVTLLVFALV     48
                    || :  :|  :  :| : :    || :: ||  |:|   | ::  || : 
B_Sequence        1 GLLSFFSPCILPLIPAYFSFITGLSLDELKENKRQTRQKVFLS-TVFYVA     49
                        1                                          2
                                                          3
A_Sequence       49 G-EWIFKFFGASTDAFS------------IAGGIILFRMSLEMLSGKLSS     85
                    |  :||  ||||                 | |||||    | :| | :: 
B_Sequence       50 GFSFIFILFGASASFLGGLASQYAWVVRYIGGGIILV-FGLHLL-GIINI     97
                                                          3
                                                  4
A_Sequence       86 VKIS-EEEEHISEEAVTLEEVAIIPLAIPLLSGP--GAITTTMLYMAKSS    132
                       : |:: |: |: : |    :| :|      |  | :  ::| :| : 
B_Sequence       98 KGFNFEKKIHVKEKPLHLMGTFVIGMAFGAGWSPCIGPLLGSILIVAGNQ    147
                                                  4
                                 5
A_Sequence      133 TMIEKSIVLLVVVAIGITV-WIILSA-ANRIHQ--KLGTIGIKVMTRMMG    178
                      : | : || | : |: | ::|||   | | :  |  |  |:|: :: |
B_Sequence      148 ETVLKGVFLLAVYSAGLAVPFLILSVFINSILEIMKRATKFIRVLNKISG    197
                                 5
                        6
A_Sequence      179 LILASMAVQMVIN    191
                    ::| :: : :| :
B_Sequence      198 ILLIAIGLLLVFD    210
                        6
#---------------------------------------
============ FINISHED =============
Average Quality (AQ)      22.82 +/- 7.67
Standard score (Z):       15.0
Precise score (Z):  15.3
```

Fig. S10E

```
# 1: A_Sequence: Sni1 (2.A.116.1.4 homologue)
# 2: B_Sequence: Psp5 (5.A.1.2.1 homologue)
# Matrix: EBLOSUM62
# Gap_penalty: 8.0
# Extend_penalty: 2.0

# Length: 194
# Identity:      46/194 (23.7%)
# Similarity:    97/194 (50.0%)
# Gaps:          15/194 ( 7.7%)
# Score: 117.0
#=======================================
                                   2                                 3
A_Sequence           1 KPRPTSLAFLAGWVVGLVGLTVVFIEASSLAGGEQHTRPAWMSWVRIILG     50
                       |  |  |  ::  |: ::| :::|:                |  |: | |
B_Sequence           1 KNSPNKLTVISQTVLFILGFSILFVLLGISVSTVSRLLSEHMRLVQQIGG     50
                                   2                                 3

                                                               4
A_Sequence          51 AALIVFGVYRF-VTRHR--HTEQPRWMRPFAKLTPG-RAG--LTGVAVAV     94
                       | ::|||::   : | :  ::|: |:: |    :|| :||  : |:| ||
B_Sequence          51 ALIVVFGLHMTGLLRIKLLYSEK-RYL-PSG--SPGKKAGALVLGMAFAV     96
                                                               4

                                                                  5
A_Sequence          95 VRPEVLALVATAGLEIGAGGLSTAGAWTCGVLFIAVAASTVAIPVLAYAI    144
                            :  : :: | | || ::| |    ||| ::: |  :|:| |  |:
B_Sequence          97 GWTPCIGPILSSIL-IYAGSMATLGK---GVLLLSMYALGLAVPFLLSAV    142
                                                                  5

                                                     6
A_Sequence         145 AGERLDPTMARIKDWMDRNLGAMEAVVLVVIGLMVIEKGISSLS    188
                         : |   : ::   : : :     ||::::|::|    :   |
B_Sequence         143 LIDNLTAYLRKVTKHLPK-ISVASGVVMMLMGVLVFTNQLEVFS    185
                                                     6

#---------------------------------------
============ FINISHED =============
Average Quality (AQ)      21.78 +/- 7.27
Standard score (Z):       13.0
Precise score (Z):  13.1
```
