# Supplementary material for: The LysE Superfamily of Transport Proteins Involved in Cell Physiology and Pathogenesis
Source: PLoS One. 2015 Oct 16;10(10):e0137184. doi: 10.1371/journal.pone.0137184 (PMC4608589; doi:10.1371/journal.pone.0137184)

Fig. S11A

LysE

Clustal

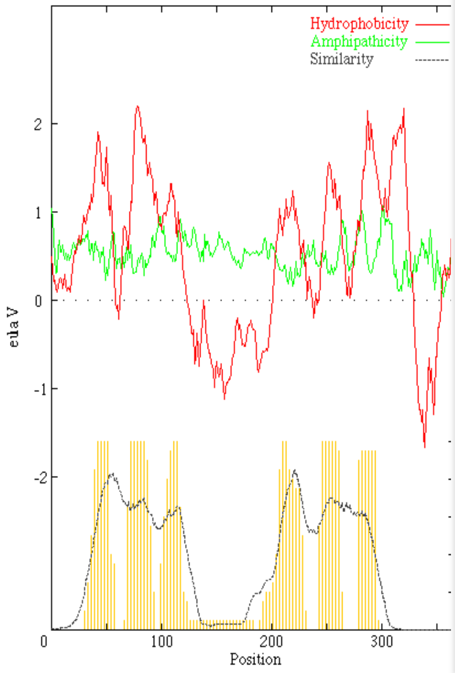

Mafft

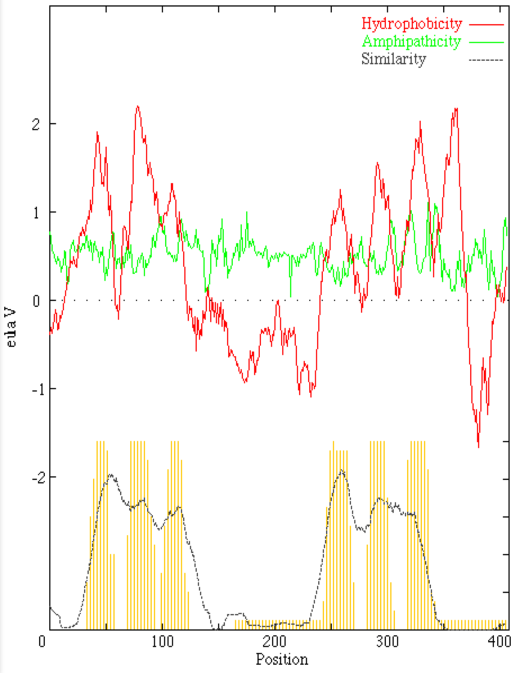

ProbCons

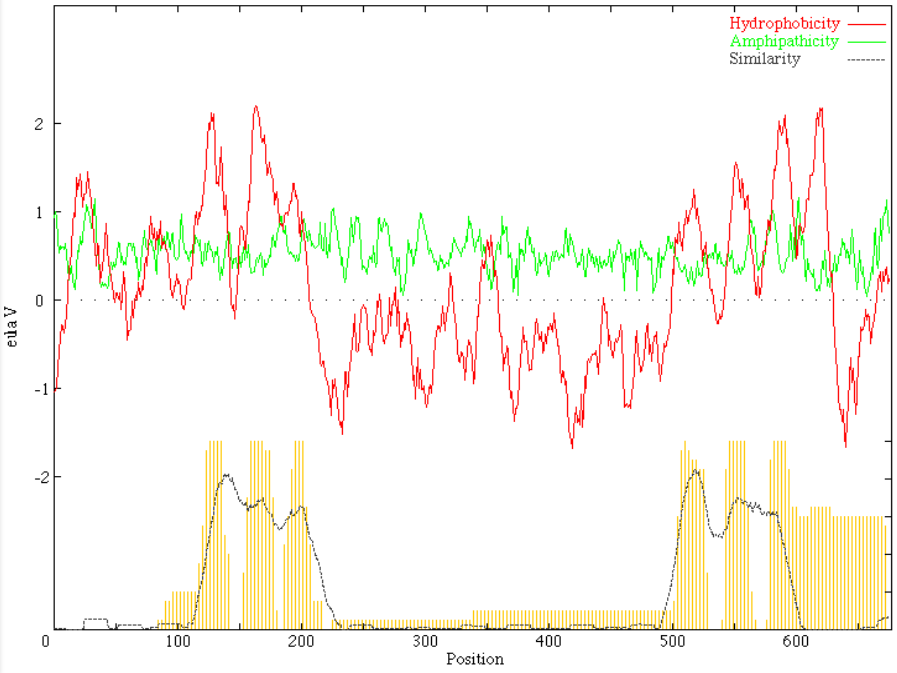

Fig. S11B

RhtB

Clustal

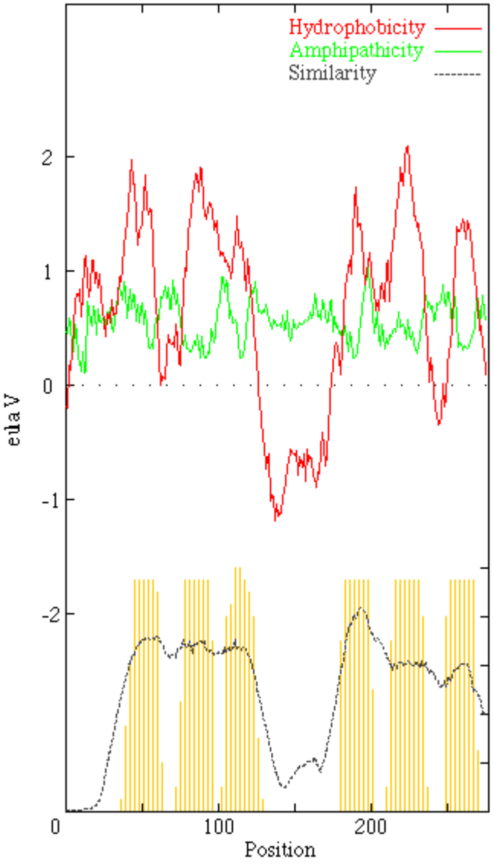

Mafft

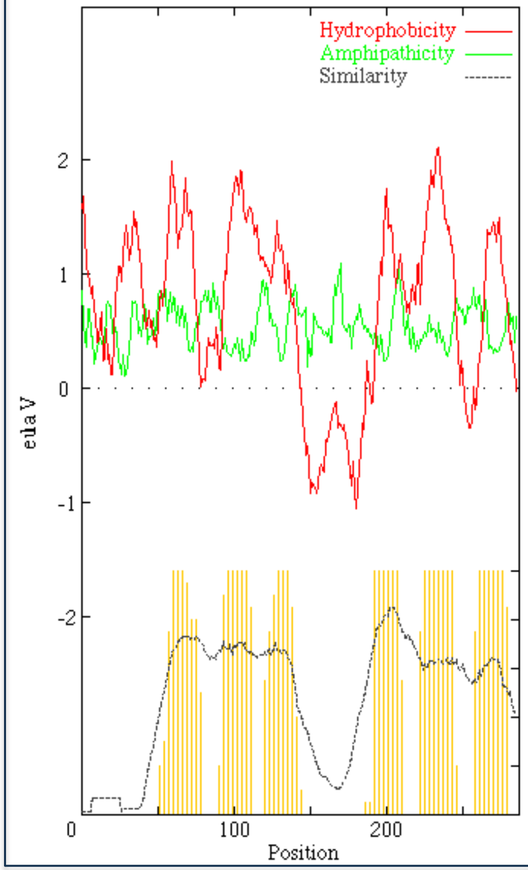

ProbCons

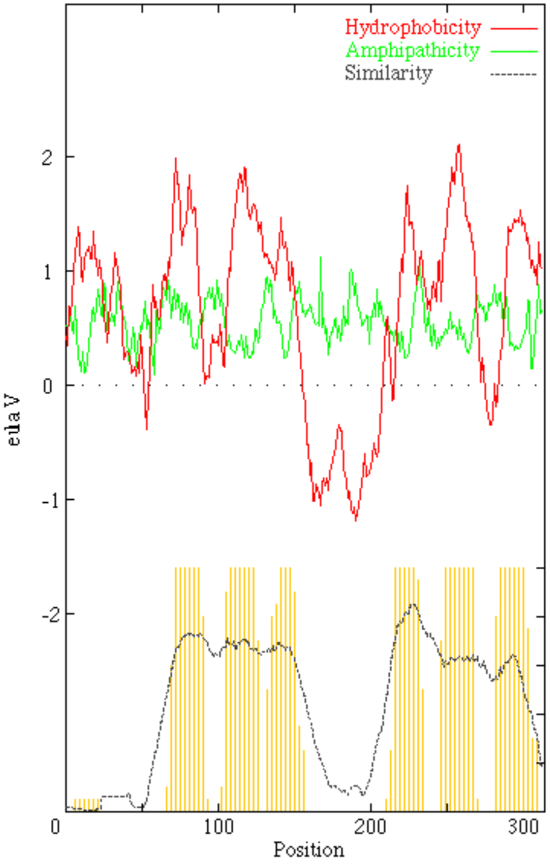

Fig. S11C

CadD

Clustal

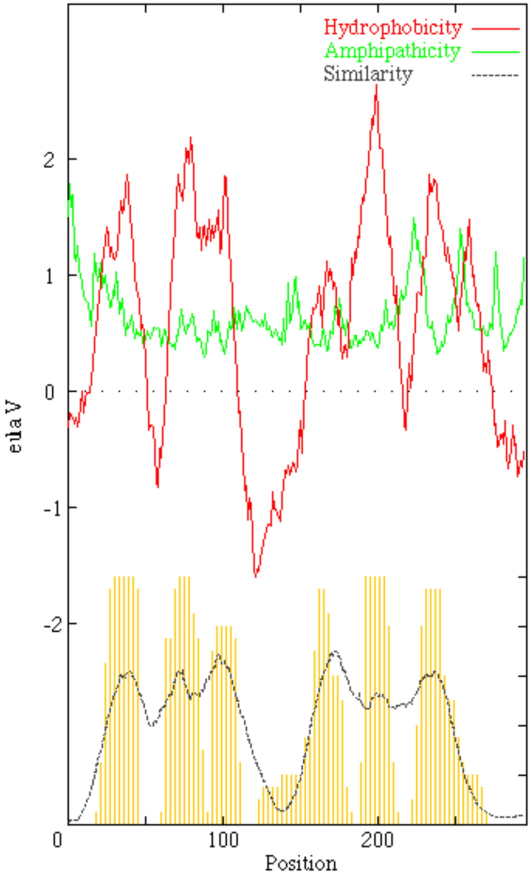

Mafft

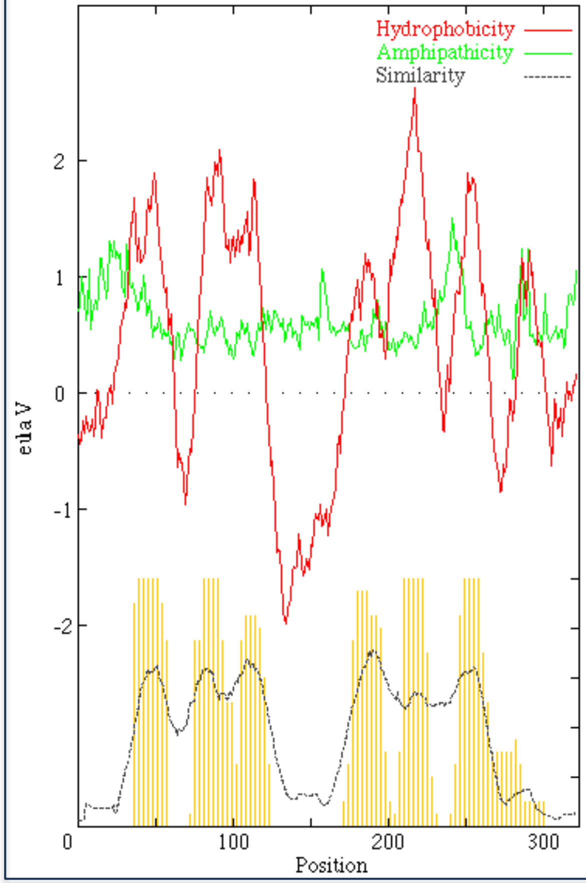

ProbCons

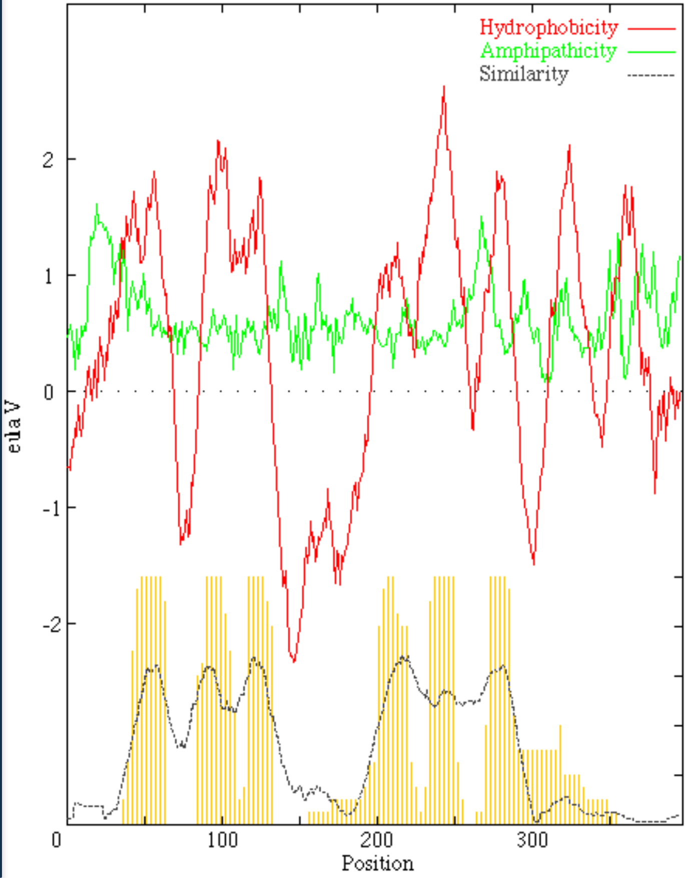

Fig. S11D

CaCA2

Clustal

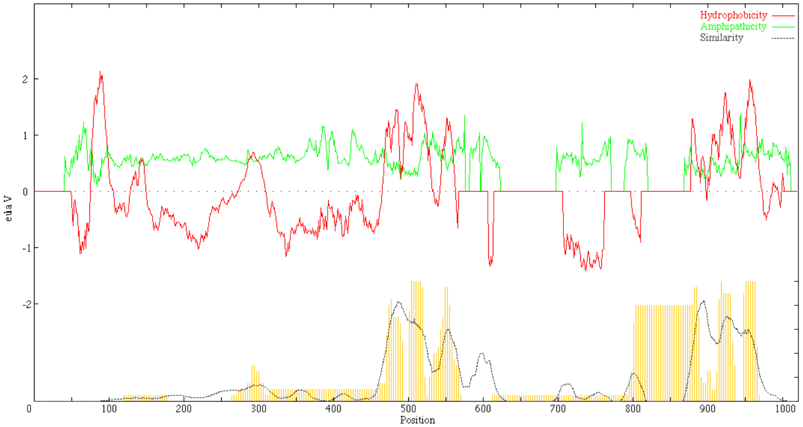

Mafft

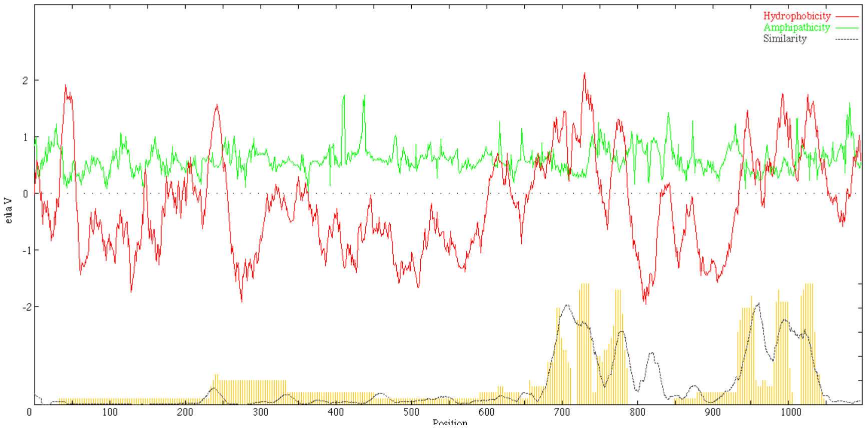

ProbCons

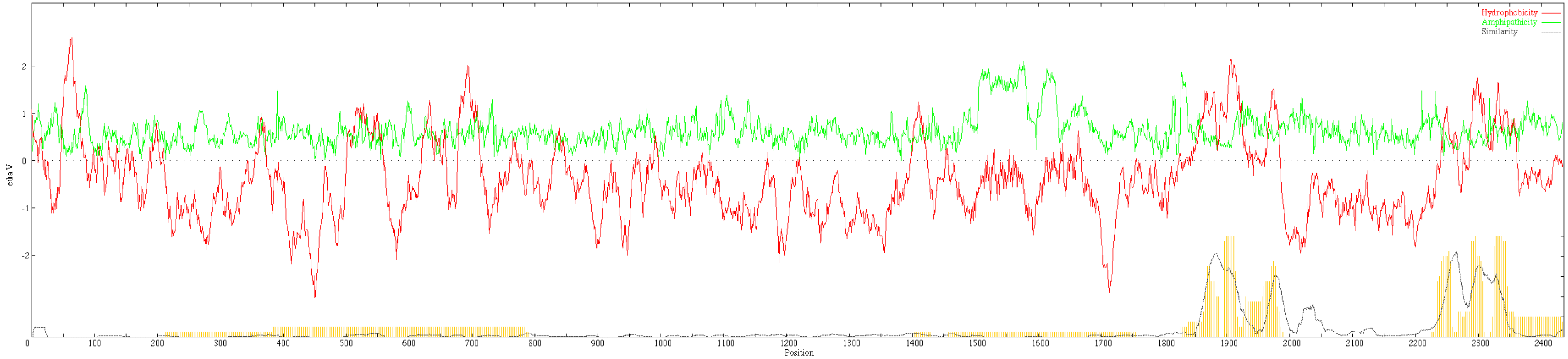

Fig. S11E

MntP

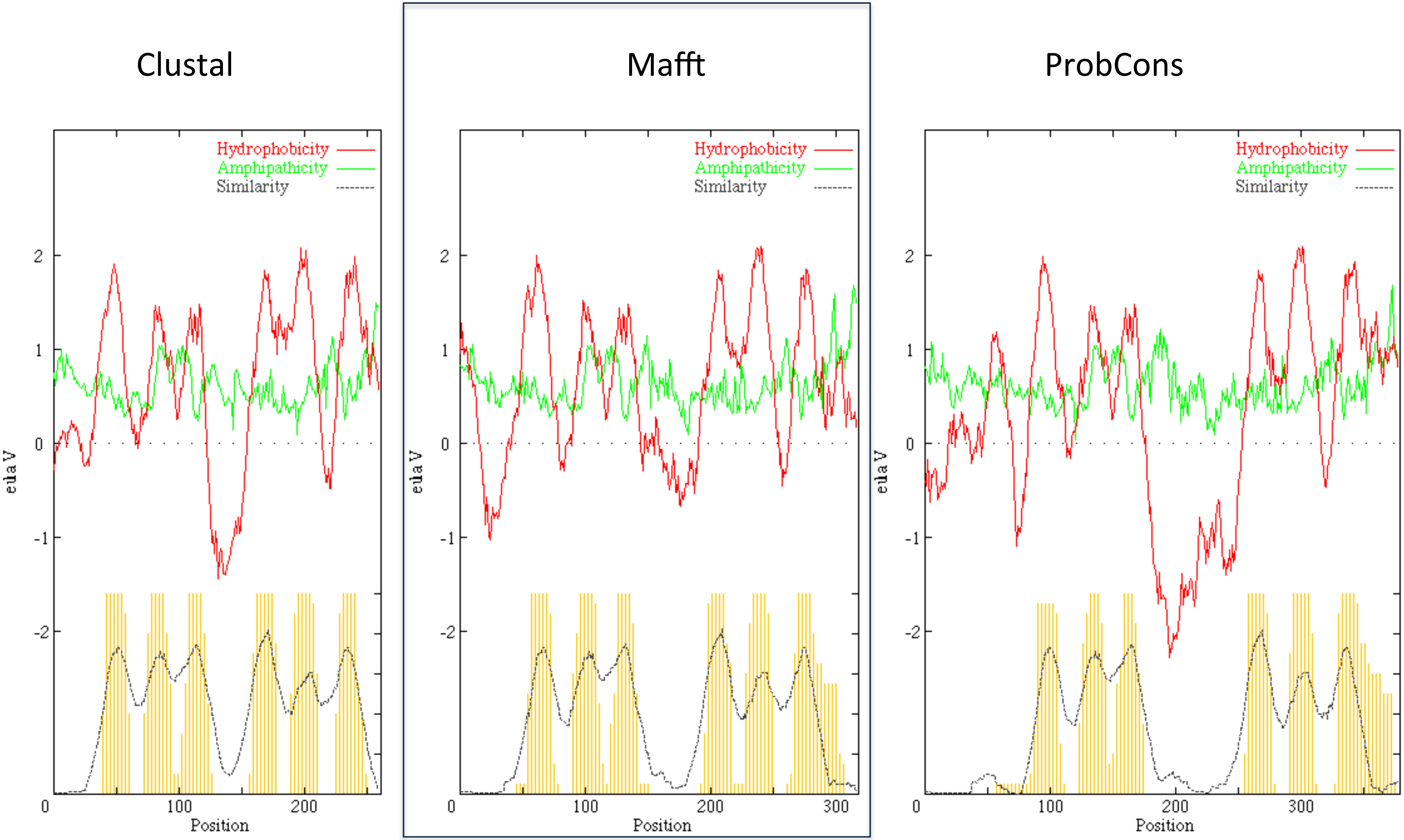

Fig. S11F
NAAT

Clustal

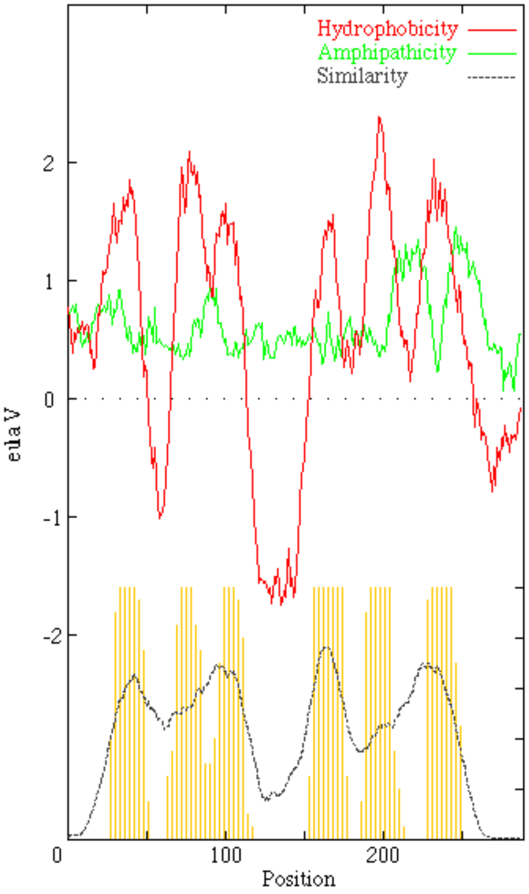

Mafft

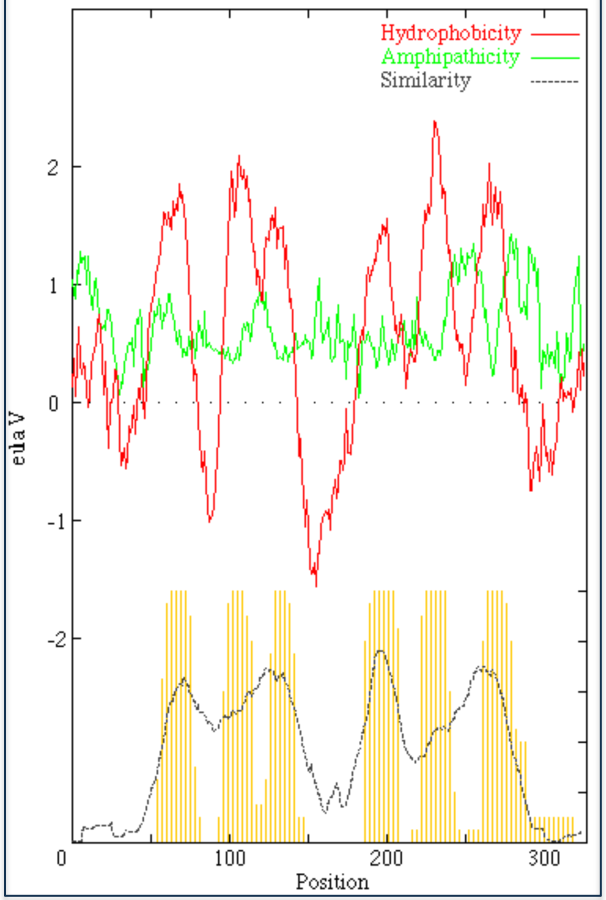

ProbCons

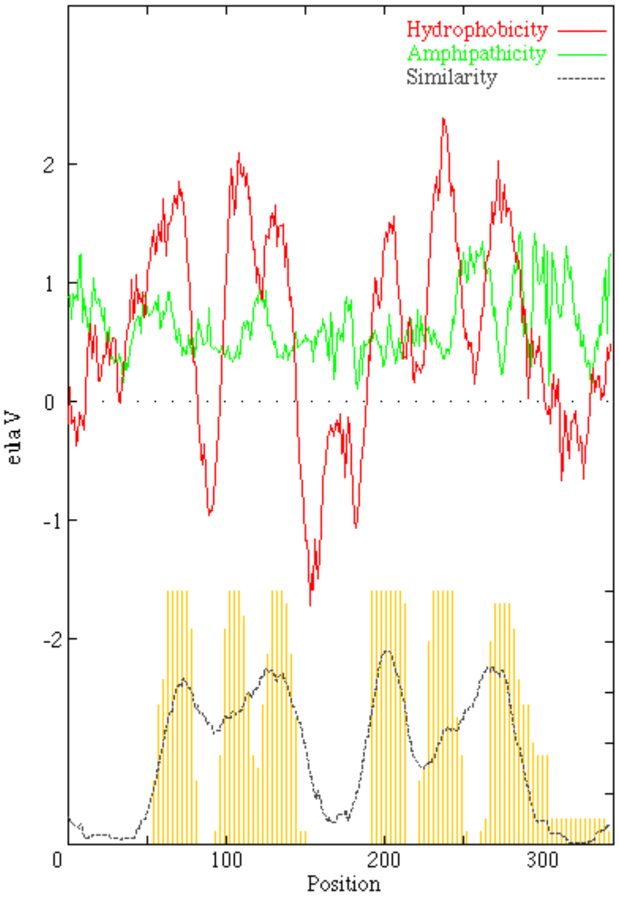

Fig. S11G

NicO

Clustal

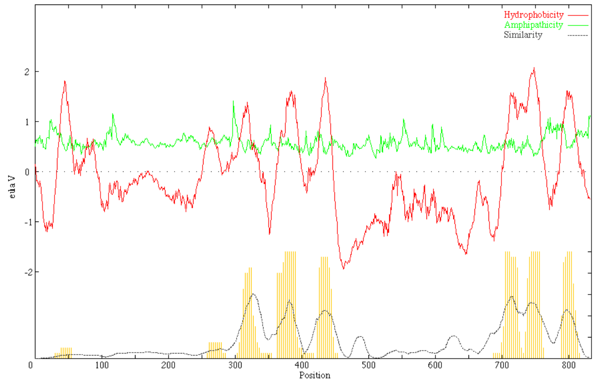

Mafft

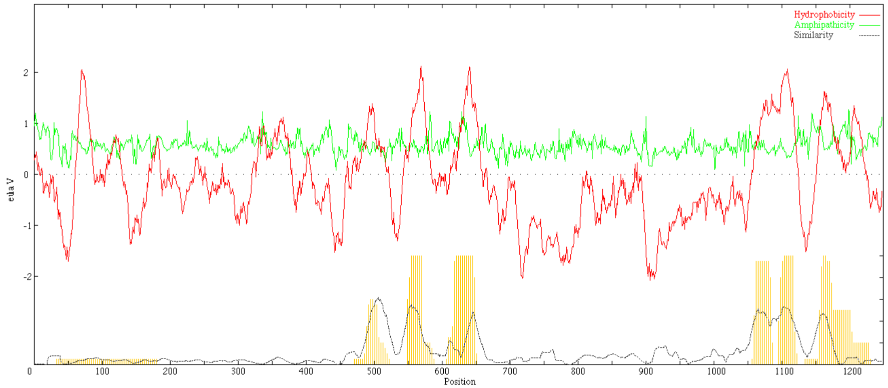

ProbCons

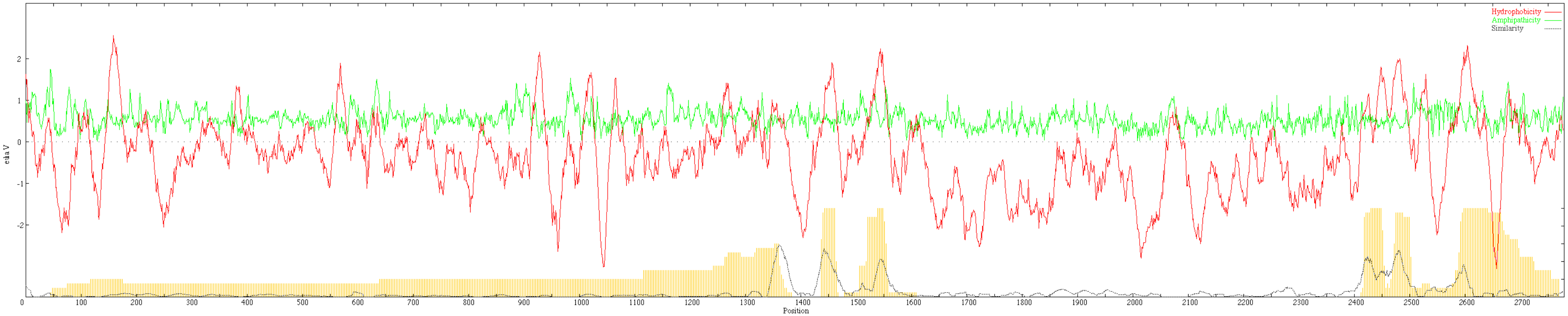

Fig. S11H

GAP

Clustal

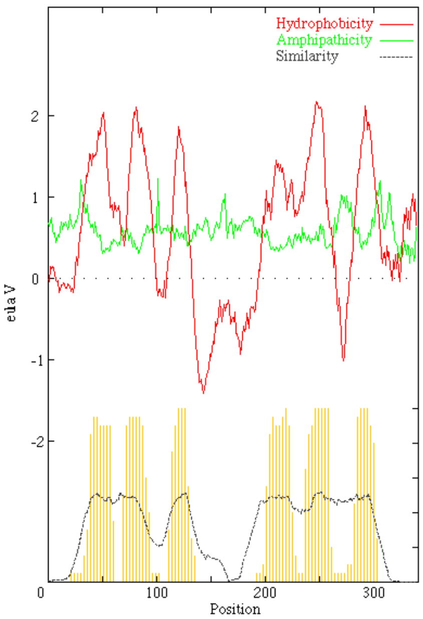

Mafft

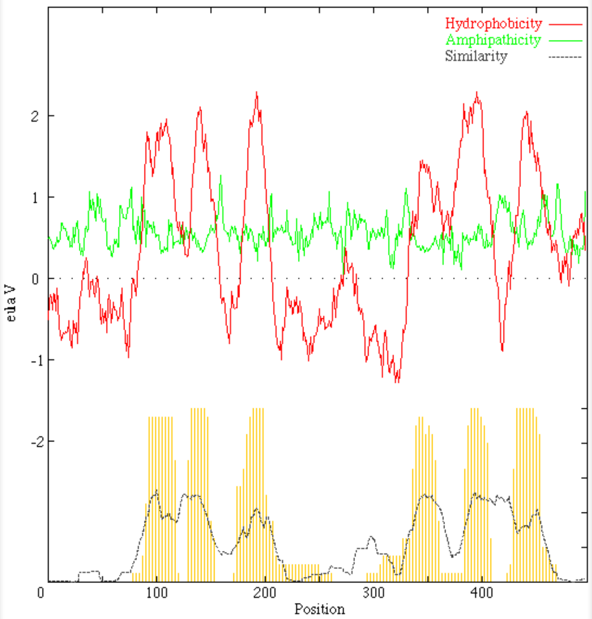

ProbCons

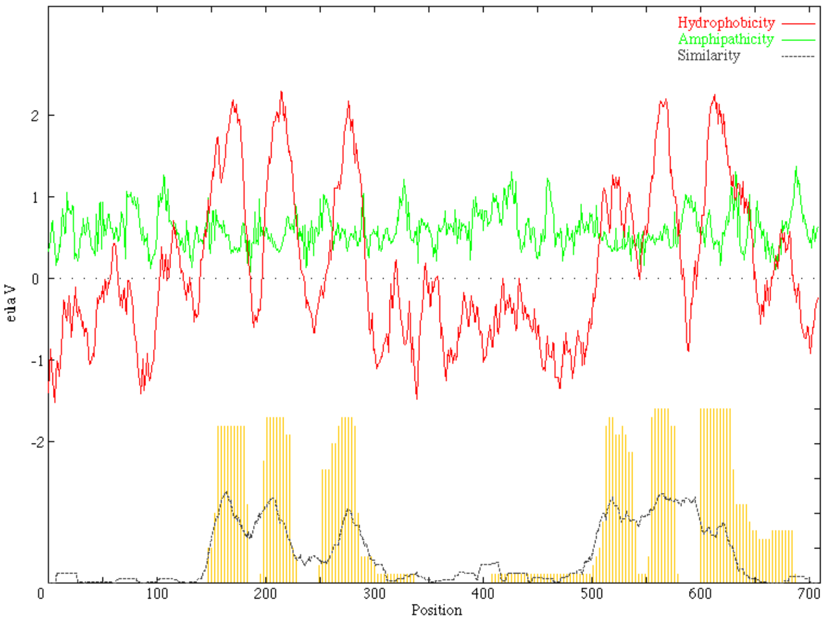

Fig. S11I

DsbD

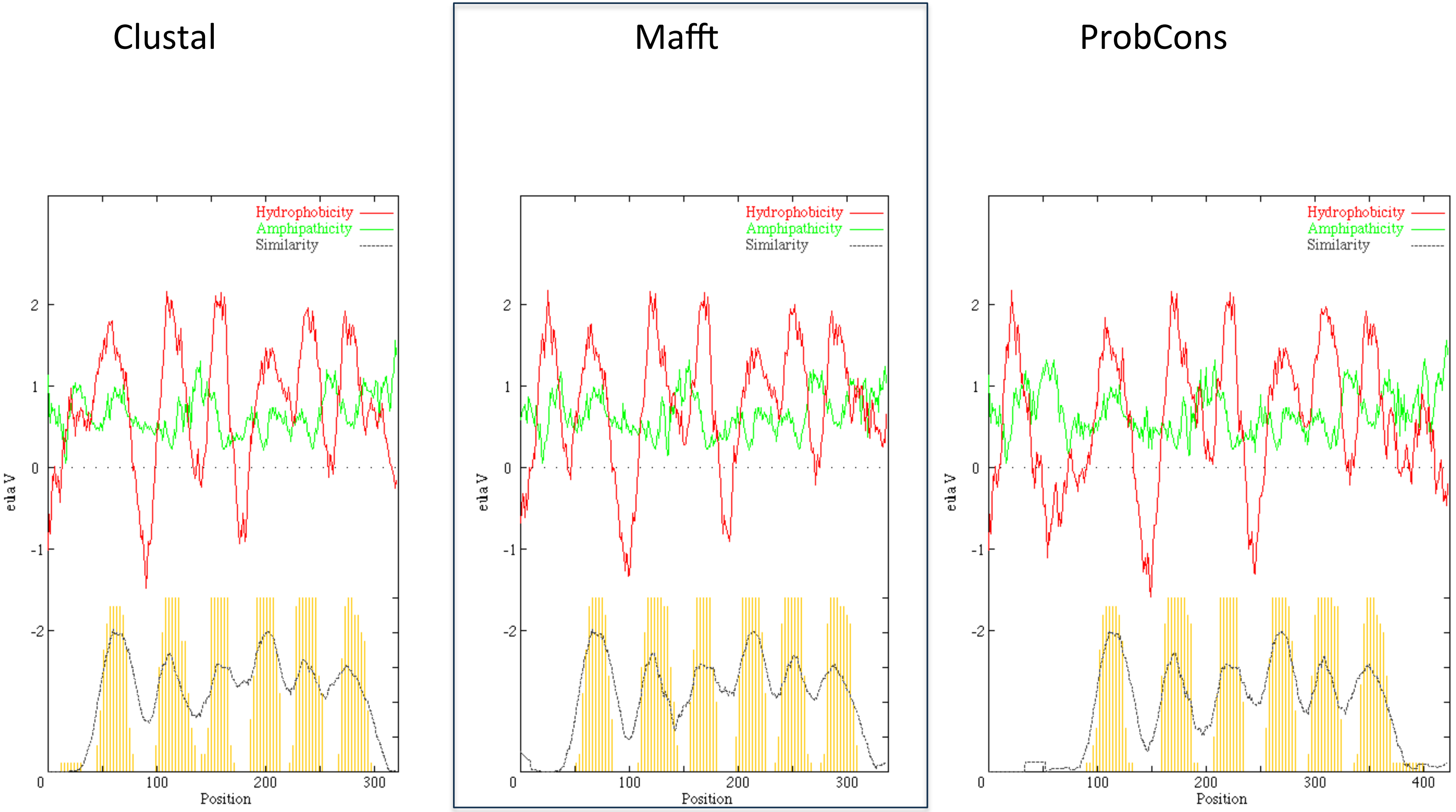

Fig. S11J

ILT

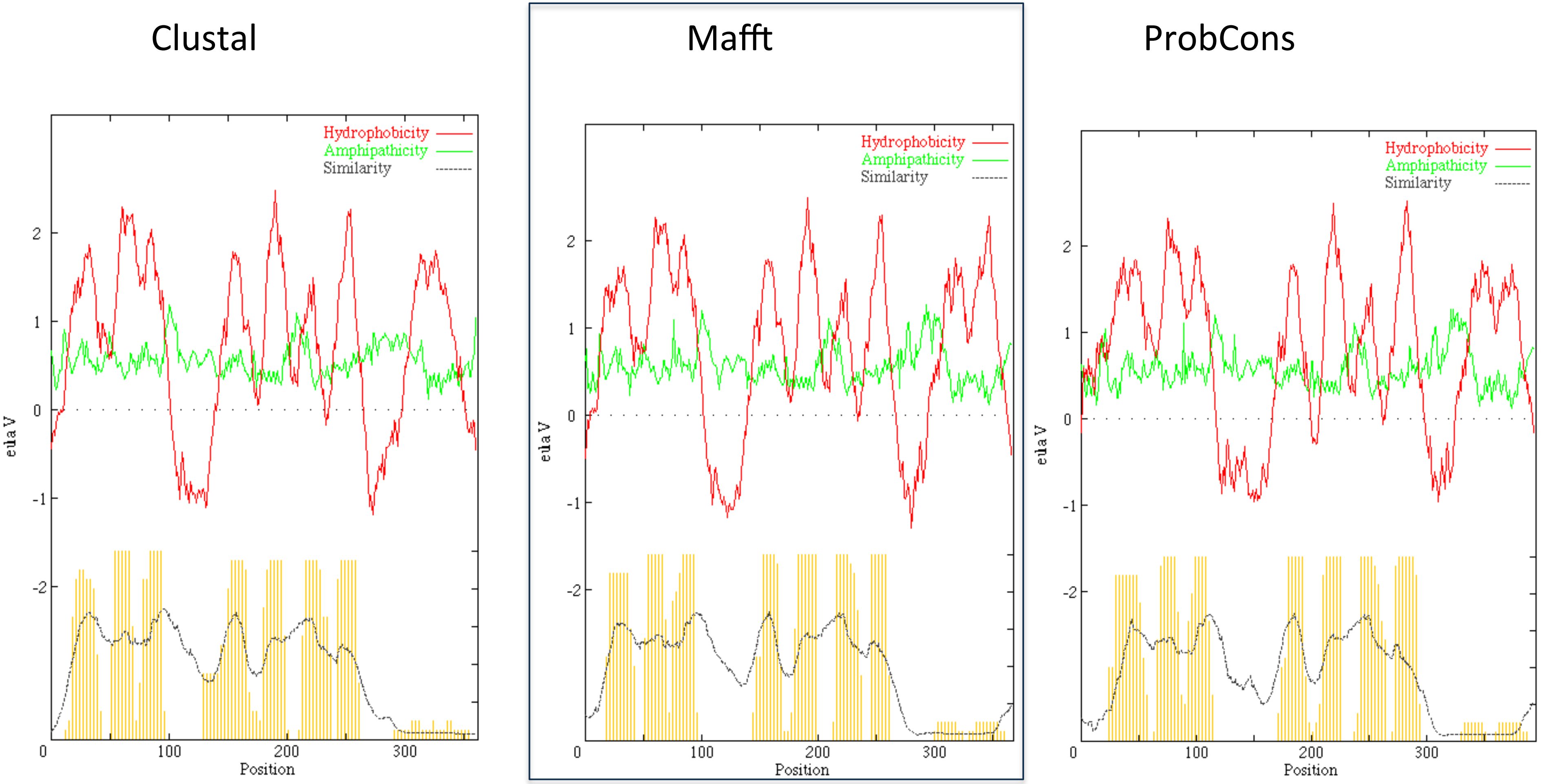
Clustal
Mafft
ProbCons
Hydrophobicity
Amphipathicity
Similarity
Position
eula V

Fig. S11K
TerC

Clustal

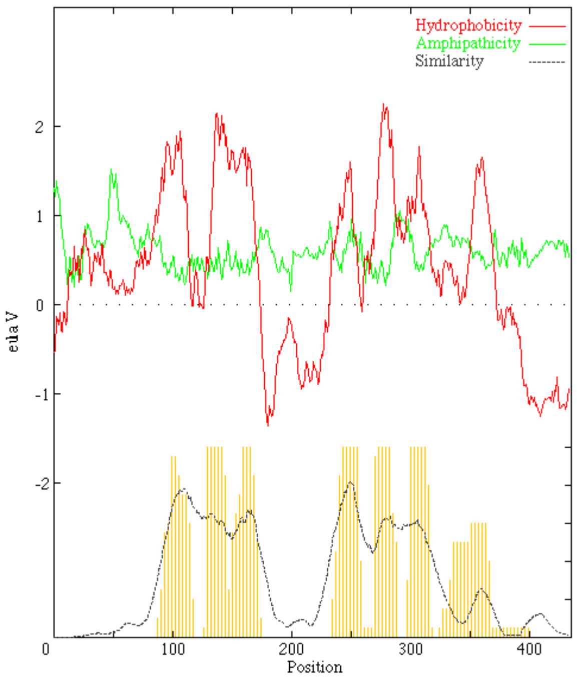

Mafft

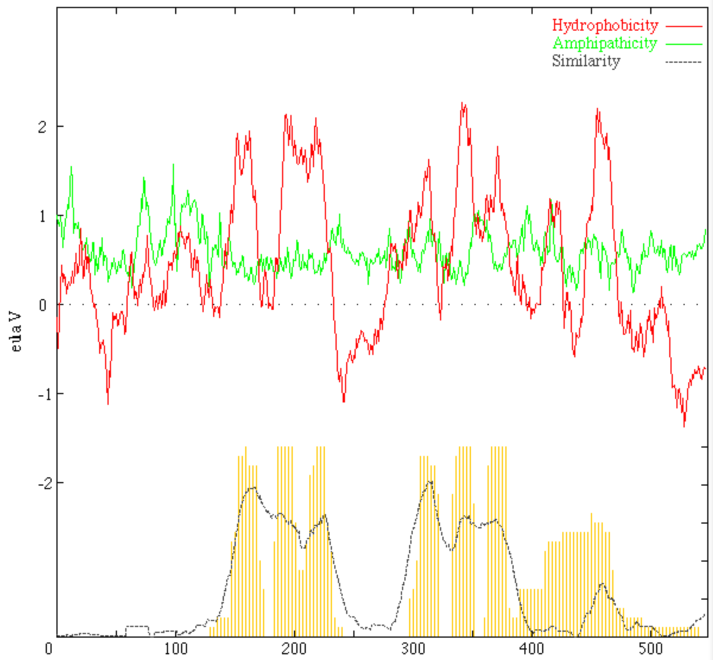

ProbCons

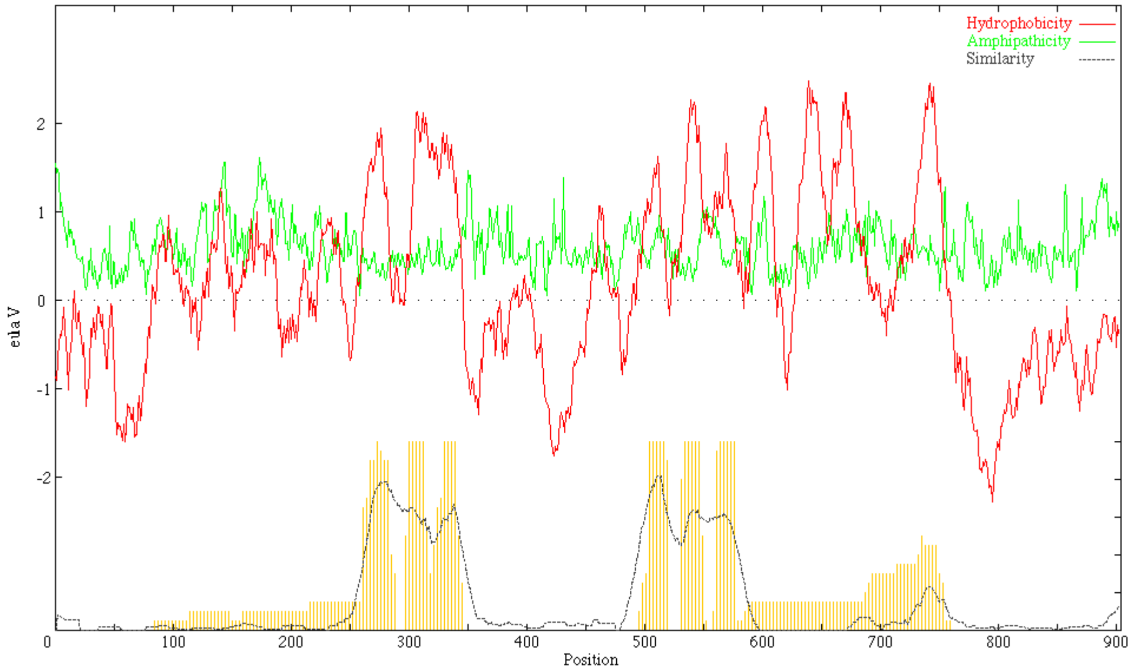

Supplement: S11 Fig — (A) LysE. (B) RhtB. (C) CadD. (D) CaCA2. (E) MntP. (F) NAAT. (G) NicO. (H) GAP. (I) DsbD. (J) ILT. (K) TerC. (PDF) [file pone.0137184.s011.pdf]
