# Supplementary material for: The LysE Superfamily of Transport Proteins Involved in Cell Physiology and Pathogenesis
Source: PLoS One. 2015 Oct 16;10(10):e0137184. doi: 10.1371/journal.pone.0137184 (PMC4608589; doi:10.1371/journal.pone.0137184)
Supplement: S12 Fig — GSAT comparisons between TMS#1–3 and TMS#4–6 for three CaCA2 homologues with assigned UniProt accession numbers. (A) Q2JWH3. (B) I7M883. (C) K4DX00. (PDF) [file pone.0137184.s012.pdf]

Fig. S12A

```
# 1: A_Sequence: Ssp2 TMS #1-3 (Q2JWH3 ; 2.A.106 homologue)
# 2: B_Sequence: Ssp2 TMS #4-6
# Matrix: EBLOSUM62
# Gap_penalty: 8.0
# Extend_penalty: 2.0
#
# Length: 118
# Identity:      30/118 (25.4%)
# Similarity:    39/118 (33.1%)
# Gaps:          29/118 (24.6%)
# Score: 111.0
#
#
#=======================================
                                                     1  ExGD(K/R)(T/S)
A_Sequence         1 ----------------------MWAGFASSLLLVTVAEFGDKTFFTPLIL     28
                                            ||    :  |  :|||||||    : |
B_Sequence         1 EEEEEALRLVEQAEAKGAGRGGAWAVVWEAFSLTALAEFGDKTQIATVSL     50
                                                     4  ExGD(K/R)(T/S)
                                  2                                 3
A_Sequence        29 AMRHPRRWVFLGTWLALAAMTLLAVVAGKVLFELLPPLGVRVLSAGVFAA     78
                     |  ||   |: |  |    |  |||| |: |   :    |  :  |:|
B_Sequence        51 AATHPGLSVWAGATLGHGLMVGLAVVGGRFLAAHISERAVHWVGGGLFLL    100
                                  5                                 6

A_Sequence        79 FGLRMLWQAYQMTPQQEK     96
                     | |   |:
B_Sequence       101 FALVTSWELLG-------    111


#---------------------------------------

============ FINISHED =============
Average Quality (AQ)      19.10 +/- 6.83
Standard score (Z):       13.0
Precise score (Z):        13.5
```

Fig. S12B

```
# 1: A_Sequence: Tth1 TMS #1-3 (I7M883 ; 2.A.106 homologue)
# 2: B_Sequence: Tth1 TMS #4-6
# Matrix: EBLOSUM62
# Gap_penalty: 8.0
# Extend_penalty: 2.0
#
# Length: 172
# Identity:      34/172 (19.8%)
# Similarity:    58/172 (33.7%)
# Gaps:          48/172 (27.9%)
# Score: 102.0
#
#
#=======================================

A_Sequence         1 MKVLYILIISFLLLSSINTKEPNNEKGNSSEKSLLNSFNDDQILQSHGSF     50
                              : |   | : |: ||:  || |          : : : |
B_Sequence         1 ---------NDLKEKSTSDKQQNNQ-ANSQENEKKKKKKQIKGIAAPGYV     40

                           1    ExGD(K/R)(T/S)                     2
A_Sequence        51 IG--SFISTSVSEIGDKTFIMTAILSSKYNRFWVFVGSVGSMLIMTLISC     98
                     |   :|:|    | |||: | |  :|: |:  :||:|:|   :   |::
B_Sequence        41 IAMQTFVSNFFGEWGDKSQISTIAISASYDFVFVFLGTVVGQIFCILLAL     90
                           4    ExGD(K/R)(T/S)                     5
                                        3
A_Sequence        99 LLGS-LTEYFIPLVYVKFISSALFLIFGLKMLYEVYTDTVDDEDDEAEEE    147
                     : |  | : |     :  :   ||:||    ||
B_Sequence        91 IGGQVLAKQFSEKT-MALLGGILFIIFSFITLYTTLNK------------    127
                                        6

A_Sequence       148 VEELEKRLSKIVTKPKTETDQN    169

B_Sequence       127 ----------------------    127


#---------------------------------------

============ FINISHED =============
Average Quality (AQ)      18.33 +/- 7.38
Standard score (Z): 11.0
Precise score (Z):  11.3
```

Fig. S12C

```
# 1: A_Sequence: Tcr1 TMS #1-3 (K4DX00 ; 2.A.106 homologue)
# 2: B_Sequence: Tcr1 TMS #4-6
# Matrix: EBLOSUM62
# Gap_penalty: 8.0
# Extend_penalty: 2.0
#
# Length: 137
# Identity:      25/137 (18.2%)
# Similarity:    47/137 (34.3%)
# Gaps:          26/137 (19.0%)
# Score: 52.0
#
#
#=======================================
                                               1  ExGD(K/R)(T/S)
A_Sequence         1 -------MAIHATRRW--TEGLLSS-FSMILVSEIGDKTFFIACLMAMRH     40
                             |  | | |     :::  |::  |:| ||::      :|
B_Sequence         1 TGSISSTGAGCARRHWFAFHPVMAEVFALTFVAEWGDRSQLATIALAAAK     50
                                               4  ExGD(K/R)(T/S)
                                 2                          3
A_Sequence        41 SKVLVFLGAIGALAGMTVLSALMGLVVPSVLSVRVTKMLAVVLFFGFGGK     90
                     :   | :| :   |  | :: | | :    :|:|   ::   ||  |
B_Sequence        51 NPFAVTIGGVLGHAVCTGVAVLCGNMTARYVSMRSVNIVGGGLFIVFALA    100
                                 5                          6
A_Sequence        91 ILYDEFAKRGQGDAESDDEMTEAAAIIRKKDPNDAVE    127
                      ||:        | |   :  :
B_Sequence       101 TLYELITNTHHID-EMQQQKEK---------------    121


#---------------------------------------

============ FINISHED =============
Average Quality (AQ)      14.58 +/- 6.61
Standard score (Z): 6.0
Precise score (Z):  5.7
```
