# Supplementary material for: The LysE Superfamily of Transport Proteins Involved in Cell Physiology and Pathogenesis
Source: PLoS One. 2015 Oct 16;10(10):e0137184. doi: 10.1371/journal.pone.0137184 (PMC4608589; doi:10.1371/journal.pone.0137184)
Supplement: S13 Fig — GSAT comparisons between TMS#1–3 and TMS#4–6 for three ILT homologues with assigned UniProt accession numbers. (A) Q8YX33. (B) K9Q6B8. (C) J2KV33. (PDF) [file pone.0137184.s013.pdf]

Fig. S13A

```
# 1: A_Sequence: Nps1 TMS #1-3 (Q8YX33 ; 2.A.108 homologue)
# 2: B_Sequence: Nps1 TMS #4-6,7
# Matrix: EBLOSUM62
# Gap_penalty: 8.0
# Extend_penalty: 2.0
#
# Length: 150
# Identity:      30/150 (20.0%)
# Similarity:    50/150 (33.3%)
# Gaps:          54/150 (36.0%)
# Score: 99.0
#
#
#=======================================
                                             1 (D/E)xxE
A_Sequence         1 -----------MNWEIVLASFASSLIELVEILGIVIVVGRL-AGWRNALV     38
                                 ||  |: :|  :|:: ||:   |: :|   : |  |:
B_Sequence         1 EQLARDENESGWNWFAVVTTFKGALLDSVEVAIAVVTLGAAQSQWLEAIS     50
                                             4 (D/E)xxE
                           2                            3
A_Sequence        39 GSGAGIALTLLLSLVLGKSLTLIPVNILRIVAGVLLLLFGQKWTRSIVRY     88
                     |:|      |:|: :    |  :||  ::  | :||: ||  |
B_Sequence        51 GAGFATFSLLVLAFLFRTPLQQVPVKPMKFTAAMLLMGFGLYWLG-----     95
                           5                            6

A_Sequence        89 YAGLPKKRKGGGEDSLE---------------------------------    105
                      |||  :  |   | |
B_Sequence        96 -AGLNVEWPG---DELAIIWLPLAWGVGMAIASTIWRWRVSLDKPEEAIG    141
                                               7

#---------------------------------------

============ FINISHED =============
Average Quality (AQ)      20.11 +/- 7.38
Standard score (Z): 11.0
Precise score (Z):  10.7
```

Fig. S13B

```
# 1: A_Sequence: Nps3 TMS #1-3 (K9Q6B8 ; 2.A.108 homologue)
# 2: B_Sequence: Nps3 TMS #4-6,7
# Matrix: EBLOSUM62
# Gap_penalty: 8.0
# Extend_penalty: 2.0
#
# Length: 151
# Identity:      30/151 (19.9%)
# Similarity:    47/151 (31.1%)
# Gaps:          58/151 (38.4%)
# Score: 96.0
#
#
#=======================================
                                               1(D/E)xxE
A_Sequence         1 -------------MNWEIFLASFVGSLIELVEILGLVLIVGKLAG-WRNA     36
                                   ||     :| |:|:: ||:   |: :|   | |  |
B_Sequence         1 LETELANTGNQLGWNWFAIATTFKGALLDSVEVAIAVVTLGATGGKWLEA     50
                                               4 (D/E)xxE
                           2                                3
A_Sequence        37 FVGA-GSGIGLTLLASLILGTSLTIIPVDILRIVAGVFLLAFGQKWTRSI     85
                       ||  :  || ::|  :  | |  :|:  ::  | : |: ||  |
B_Sequence        51 AGGASAAAFGLVVVA-FLFRTPLNQVPIKPMKFTAAMLLMGFGIYWLSE-     98
                           5                                6

A_Sequence        86 VKYYAGIPKKRKDEEDD----------------------------------    102
                          |   | |   ||
B_Sequence        99 -----GF--KIKLPGDDWAIVWLPIVWGCLMAVSALLLRWQVGLQPKEIV    141
                                               7

A_Sequence       102 -    102

B_Sequence       142 S    142


#---------------------------------------

============ FINISHED =============
Average Quality (AQ)      21.97 +/- 7.85
Standard score (Z): 9.0
Precise score (Z):  9.4
```

Fig. S13C

```
# 1: A_Sequence: Rsp3 TMS #1-3 (J2KV33 ; 2.A.108 homologue)
# 2: B_Sequence: Rsp3 TMS #4-6,7
# Matrix: EBLOSUM62
# Gap_penalty: 8.0
# Extend_penalty: 2.0
#
# Length: 140
# Identity:      37/140 (26.4%)
# Similarity:    56/140 (40.0%)
# Gaps:          30/140 (21.4%)
# Score: 84.0
#
#
#=======================================
                                        1 (D/E)xxE
A_Sequence         1 MTTITSITSTMA--ASFLGSFVEVVEAFTIILAVGVTQSWRPAFIGTGLA      48
                       :       :|  |:|    :| ||   |::| |      | : | |
B_Sequence         1 --SADRRADFLAGTAAFKAVLLEGVEVVFIVIATGARPGMLP-YAGLGAL      47
                                        4 (D/E)xxE
                           2                          3
A_Sequence        49 LSVLAVLV---LIFGPLLGLIPIDILQFTIGTLLILFGMRWLRKAI----      91
                     :: :||||   |:  | |  :| : |:| :| ||  ||: |: : |
B_Sequence        48 IACIAVLVIGLLVHKP-LSSVPENTLKFIVGLLLTAFGIFWIGEGIGTPW      96
                           5                          6

A_Sequence        92 ----LRASGFIALHDEEKAFASETDALARQ----------    117
                         |   |  ||     ||:       ||
B_Sequence        97 PGEDLSLIGIFAL---LAAFSFIAVRWLRQYHHAQTEPAR    133
                                  7

#---------------------------------------

============ FINISHED =============
Average Quality (AQ)       22.15 +/- 7.74
Standard score (Z): 8.0
Precise score (Z):  **8.0**
```
