# Supplementary material for: The LysE Superfamily of Transport Proteins Involved in Cell Physiology and Pathogenesis
Source: PLoS One. 2015 Oct 16;10(10):e0137184. doi: 10.1371/journal.pone.0137184 (PMC4608589; doi:10.1371/journal.pone.0137184)
Supplement: S14 Fig — GSAT comparisons between TMS#1–3 and TMS#4–6 for three MntP homologues with assigned UniProt accession numbers. (A) A8SU47. (B) R9SLI6. (C) C6JCY1. (PDF) [file pone.0137184.s014.pdf]

Fig. S14A

```
# 1: A_Sequence: Ceu1 TMS #1-3 (A8SU47 ; 2.A.107 homologue)
# 2: B_Sequence: Ceu1 TMS #4-6
# Matrix: EBLOSUM62
# Gap_penalty: 8.0
# Extend_penalty: 2.0
#
# Length: 98
# Identity:      22/98 (22.4%)
# Similarity:    42/98 (42.9%)
# Gaps:           8/98 ( 8.2%)
# Score: 82.0
#
#
#=======================================
                                 1 Fully conserved D
A_Sequence         1 ---MSIVELFMLAVGLSMDAFAVSICKGLSLRDIKVKHMVIAGVWFGGFQ     47
                        ||   :|:|||  |:|| || :    :   :   ::|:| :: |
B_Sequence         1 NADMSAKVMFLLAVATSIDALAVGV--SFAFLKLSTLYIVLAVIFIGCIT     48
                                 4 Fully conserved D
                           2                         3
A_Sequence        48 ALMPTLGYVLGSFFADLVSKWSHWIAFVLLLFIGGSMIKESFGGEEEV     95
                      :    |  :|| |       :     ::|: ||  :: :  |
B_Sequence        49 FIFSAAGVKIGSIFGTKYKSKAELAGGIILILIGIKVVLDGLGIL---     93
                           5                         6

#---------------------------------------

============ FINISHED =============
Average Quality (AQ)      24.98 +/- 7.01
Standard score (Z): 8.0
Precise score (Z):  8.1
```

Fig. S14B

```
# 1: A_Sequence: Rsp2 TMS #1-3 (R9SLI6 ; 2.A.107 homologue)
# 2: B_Sequence: Rsp2 TMS #4-6
# Matrix: EBLOSUM62
# Gap_penalty: 8.0
# Extend_penalty: 2.0
#
# Length: 102
# Identity:      22/102 (21.6%)
# Similarity:    40/102 (39.2%)
# Gaps:          20/102 (19.6%)
# Score: 64.0
#
#
#=======================================
                                        1 Fully conserved D
A_Sequence         1 ----------MNIFELFILAIGLSMDAFAVSVCKGLSLGRINAKHMCIAG     40
                               |:|  :||||:  |:|| ||    |::   :  : :  |
B_Sequence         1 SKEEEHVNADMDIKSMFILAVATSIDALAV----GVTFAFLKVE-IVSAV     45
                                        4 Fully conserved D
                           2                                3
A_Sequence        41 AWFGGFQALMPLVGYFGGRFFADKVTRYSHWVAFVLLVFIGAGMIKE---     87
                     :: |    :    |   |  |  |    :     ::|: ||  :: |
B_Sequence        46 SFIGVITFVCSAAGVKIGSLFGMKYKSKAELCGGIILILIGTKILLEGLG     95
                           5                                6

A_Sequence        87 --     87

B_Sequence        96 MI     97


#---------------------------------------

============ FINISHED =============
Average Quality (AQ)       19.03 +/- 6.10
Standard score (Z): 7.0
Precise score (Z):  7.4
```

Fig. S14C

```
# 1: A_Sequence: Msp1 TMS #1-3 (C6JCY1 ; 2.A.107 homologue)
# 2: B_Sequence: Msp1 TMS #4-6
# Matrix: EBLOSUM62
# Gap_penalty: 8.0
# Extend_penalty: 2.0
#
# Length: 103
# Identity:      22/103 (21.4%)
# Similarity:    43/103 (41.7%)
# Gaps:          20/103 (19.4%)
# Score: 67.0
#
#
#=======================================
                                       1 Fully conserved D
A_Sequence         1 -----------MDIVSTLLIAVALAMDAFSVSLTKGFTLKNITLKQILWF     39
                                     :|:||| ::|||:| :|    ||    |  ::
B_Sequence         1 FSDDLDDDEDTFSFAELILLAVATSIDAFAVGVTYA-VLKIDILIPVIII     49
                                       4 Fully conserved D
                           2                                 3
A_Sequence        40 GVFFGGFQSLMPILGWTLGVQLQLIVSEVAPWIAFILLVLIGANMIRES-     88
                     |:     |  :  |:|  || ::      :     :  ::|:|:|  :: |
B_Sequence        50 GLV--AF--IFTIIGIYLGKKIGDYFGDKFEILGGVILILLGCRILLEGL     95
                           5                                 6

A_Sequence        88 ---     88

B_Sequence        96 GFL     98


#---------------------------------------

============ FINISHED =============
Average Quality (AQ)       21.80 +/- 6.60
Standard score (Z): 7.0
Precise score (Z):  6.9
```
