# Supplementary material for: The LysE Superfamily of Transport Proteins Involved in Cell Physiology and Pathogenesis
Source: PLoS One. 2015 Oct 16;10(10):e0137184. doi: 10.1371/journal.pone.0137184 (PMC4608589; doi:10.1371/journal.pone.0137184)
Supplement: S15 Fig — GSAT comparisons between TMS#1–3 and TMS#4–6 for three TerC homologues with assigned UniProt accession numbers. (A) A4IKQ1. (B) G8M4S7. (C) R9LI44. (PDF) [file pone.0137184.s015.pdf]

Fig. S15A

```
# 1: A_Sequence: Gth1 TMS #1-3 (A4IKQ1 ; 2.A.109 homologue)
# 2: B_Sequence: Gth1 TMS #4-6,7
# Matrix: EBLOSUM62
# Gap_penalty: 8.0
# Extend_penalty: 2.0
#
# Length: 141
# Identity:      29/141 (20.6%)
# Similarity:    52/141 (36.9%)
# Gaps:          50/141 (35.5%)
# Score: 98.0
#
#
#=======================================
                                       1 DxxxxxDN
A_Sequence         1 MSVDLFSPEFWTALLSIVIIDLVLAGDNAIVIGLAARNLPKHQQKKAVIW     50
                               | |: :|:| | ::  || : :  ||     |     ||
B_Sequence         1 -------GSLWEAVRTIIIADALMGLDNVLAVAGAA-----HGHFLLVIL     38
                                       4 DxxxxxDN
                           2                         3
A_Sequence        51 GTVGAVVIRAM-ATIFVVWLLKIPGLLLVGGLLLVWIAYKLLVEE---KG     96
                     | : :| |    :|: : |: : | :: :|  :| | | |::|:|   ||
B_Sequence        39 GLLISVPIMVWGSTLILKWIERFPIIITIGAGILAWTASKMIVDEPFLKG     88
                           5                         6

A_Sequence        97 H---DDIEAG-------------------------------    103
                     :     |: |
B_Sequence        89 YFANPVIKYGFELLLVAAVIAIGTQKKRKAAKKPHLKVANE    129
                                 7

#---------------------------------------

============ FINISHED =============
Average Quality (AQ)       23.59 +/- 7.93
Standard score (Z): 9.0
Precise score (Z):  9.4
```

Fig. S15B

```
# 1: A_Sequence: Bsp2 TMS #1-3 (G8M4S7 ; 2.A.109 homologue)
# 2: B_Sequence: Bsp2 TMS #4-6,7
# Matrix: EBLOSUM62
# Gap_penalty: 8.0
# Extend_penalty: 2.0
#
# Length: 143
# Identity:      30/143 (21.0%)
# Similarity:    44/143 (30.8%)
# Gaps:          50/143 (35.0%)
# Score: 92.0
#
#
#=======================================
                                      1 DxxxxxDN
A_Sequence         1 MLEFFSTLHWGAVVQIIVIDILLGGDNAVVIALACRNLPDRQRTRGIVLG     50
                          |   | ||  |:: | ::  || : || |      | |   :: |
B_Sequence         1 -----SDRLWAAVKTIVIADAVMSLDNVIAIAGAAEAADPRHRLALVIFG     45
                                      4 DxxxxxDN
                           2                         3
A_Sequence        51 TLGAILLRVILIAFAVMLLD-VPFLKFVGGVLLLWIGVKLMQPDHDEHHI     99
                      : :| | |      : |||  | :  :|  || ||   |:    |:  |
B_Sequence        46 LIVSIPLIVWGSTLVLKLLDRFPVVVLLGAALLGWIAGGLI---IDDPFI     92
                           5                         6

A_Sequence       100 DA-----------------------------------------    101
                     |
B_Sequence        93 DRWPALNTDIVGYAARVAGALFVVGVGWLLRRRALADGNRATG    135
                                        7

#---------------------------------------

============ FINISHED =============
Average Quality (AQ)       21.13 +/- 7.79
Standard score (Z): 9.0
Precise score (Z):  9.1
```

Fig. S15C

```
# 1: A_Sequence: Pba1 TMS #1-3 (R9LI44 ; 2.A.109 homologue)
# 2: B_Sequence: Pba1 TMS #4-6,7
# Matrix: EBLOSUM62
# Gap_penalty: 8.0
# Extend_penalty: 2.0
#
# Length: 143
# Identity:      25/143 (17.5%)
# Similarity:    47/143 (32.9%)
# Gaps:          53/143 (37.1%)
# Score: 82.0
#
#
#=======================================
                                      1 DxxxxxDN
A_Sequence         1 MDLLSPEFWMALLSIVLIDLVLAGDNAIVIGLAARNVPQQDQKKVIVWGT     50
                           : | |: :|:: | ::  || : :  ||      |   ||: |
B_Sequence         1 -----NQMWAAIRTIIIADAMMGLDNVLAVAGAAHG----DTLLVII-GL     40
                                      4 DxxxxxDN
                           2                          3
A_Sequence        51 LGAILIRVVMTLLVVQLL-NIPGLRLAGGLALVWIAYKLLIEEK-SHEIK     98
                       :: | |  : ::::|    | :   |   | | | |:::||   |:
B_Sequence        41 AVSVPIMVWGSTMILKLTERFPIVITIGAAVLAWTASKMIVEEPLIHDWF     90
                           5                          6

A_Sequence        99 AG-----------------------------------------    100
                     |
B_Sequence        91 ASPWIKYGFELLVIAAVVLLGNLMKKRKARLHQAKAMPQTNGS    133
                           7

#---------------------------------------

============ FINISHED =============
Average Quality (AQ)      23.22 +/- 7.53
Standard score (Z): 8.0
Precise score (Z):  7.8
```
