# Supplementary material for: The LysE Superfamily of Transport Proteins Involved in Cell Physiology and Pathogenesis
Source: PLoS One. 2015 Oct 16;10(10):e0137184. doi: 10.1371/journal.pone.0137184 (PMC4608589; doi:10.1371/journal.pone.0137184)
Supplement: S16 Fig — (A) LysE. (B) RhtB. (C) CadD. (D) CaCA2. (E) MntP. (F) ILT. (G) TerC. (H) NAAT. (I) NicO. (J) GAP. (K) DsbD. (PDF) [file pone.0137184.s016.pdf]

Fig. S16A

```
# 1: A_Sequence: Ame2 (MC homologue)
# 2: B_Sequence: Spl1 (LysE homologue)
# Matrix: EBLOSUM62
# Gap_penalty: 8.0
# Extend_penalty: 2.0
#
# Length: 195
# Identity:      16/195 ( 8.2%)
# Similarity:    35/195 (17.9%)
# Gaps:         131/195 (67.2%)
# Score: 38.0
#
#
#=======================================
                          4
A_Sequence          1 VLGLYRGFNVSVQGIIIYRAAYFGFYDTTKNLLPDPKKTPLHITFLIAQT     50

B_Sequence          0 --------------------------------------------------      0

                          5
A_Sequence         51 VTTLAGIISYPFDTVRRRMMMQSGLKRAE----VMYKNTLDCWIKTAKTE     96
                                         ::  |:::       :  :  | :: :|
B_Sequence          1 -------------------VLTQGIRKQHRFVVALICSLCDAFLISAGVA     31
                                          1                 2

A_Sequence         97 GIAAFFKGSLSNI-LRGTGGALVLTLYDSIKDILEKSLRK----------    135
                      |: :  : | : : | | |||| | :|  :|  |  :|:
B_Sequence         32 GLGSLIEQSPTLLRLAGGGGALFLFIY-GLK-CLFSALQAEQELGETESN     79
                                           3

A_Sequence        135 ---------------------------------------------    135

B_Sequence         80 PTSRRQVILTILAITLCNPNVYLDTVVLLGGISATFVGQGRYLFG    124
                                   4

#---------------------------------------

============ FINISHED =============
Average Quality (AQ)      14.44 +/- 5.82
Standard score (Z):       4.0
Precise score (Z):        4.1
```

Fig. S16B

```
# 1: A_Sequence: Pmo1 (MC homologue)
# 2: B_Sequence: Hgr1 (RhtB homologue)
# Matrix: EBLOSUM62
# Gap_penalty: 8.0
# Extend_penalty: 2.0
#
# Length: 89
# Identity:      26/89 (29.2%)
# Similarity:    39/89 (43.8%)
# Gaps:           7/89 ( 7.9%)
# Score: 70.0
#
#
#=======================================
                                  3
A_Sequence         1 TQFWRYFIGNLASGGAAGDTSLCFVYTLDFARTRLAADIGKGAGQREFNG     50
                     |  | | :| :      |  || || ::  ||      :|  |    |
B_Sequence         1 TDLWTYVLGAIGIVLLPGPNSL-FVLSVATAR---GVRVGYHAACGVF--     44
                                  1                                 2
                                          4
A_Sequence        51 LGDCLVKIFKADGIMGLYRGFGVSVQGIIIYRAAFFGFY     89
                     ||| :: :| | |   | ||:      :: |  | : |:
B_Sequence        45 LGDSILLLFTALGAASLLRGYPALFM-VVKYVGAAYLFW     82
                                                   3

#---------------------------------------

============ FINISHED =============
Average Quality (AQ)     16.98 +/- 5.99
Standard score (Z):      9.0
Precise score (Z):       8.8
```

Fig. S16C

```
# 1: A_Sequence: Oga1 (MC homologue)
# 2: B_Sequence: Sro1 (CadD homologue)
# Matrix: EBLOSUM62
# Gap_penalty: 8.0
# Extend_penalty: 2.0
#
# Length: 87
# Identity:      26/87 (29.9%)
# Similarity:    39/87 (44.8%)
# Gaps:          14/87 (16.1%)
# Score: 59.0
#
#
#=======================================
                           5
A_Sequence         1 ISWMIAQSATAVAGLTSYPFDTVHHCMMMQSVRKGTGIMYTGATDCWRKI      50
                     |: ::| || |  |||  | |      ::  |  | |:        |  :
B_Sequence         1 IAILVAVSAVAALGLTVVP-D--RWAGLLGLVPFGMGV--------WGLV      39
                           2                          3

                                        6
A_Sequence        51 LRDEGGKAFFKGAXSSVLRGVGGAFVLVLYDEIKKYT     87
                      :|:||:|   |  :| :  | |  :    | |  ||
B_Sequence        40 RKDDGGEA---GPVASGVVSVAGVTLANGADNISVYT     73
                                        4

#---------------------------------------

============ FINISHED =============
Average Quality (AQ)      13.78 +/- 5.35
Standard score (Z): 8.0
Precise score (Z):  8.5
```

Fig. S16D

```
# 1: A_Sequence: Isc1 (MC homologue)
# 2: B_Sequence: Ghi1 (CaCA2 homologue)
# Matrix: EBLOSUM62
# Gap_penalty: 8.0
# Extend_penalty: 2.0
#
# Length: 97
# Identity:      30/97 (30.9%)
# Similarity:    51/97 (52.6%)
# Gaps:          10/97 (10.3%)
# Score: 75.0
#
#
#=======================================
                                        4
A_Sequence         1 LGNCLTKIFKSDGL-MGLYRGFG--VSVQGIIIYRAA-YFGF--FDTAKG     44
                     :|: |  :  :| | : : | ||  :  : : :: || :|||  :   :|
B_Sequence         1 IGSTLGMV-AADALAIAIGRAFGRHLPERTVALFAAALFFGFGIWLLTQG     49
                           5                         6
                                        5
A_Sequence        45 MLPDPKNTPLVISWLIAQTVTTVAGIMSYPFDTVRRRMMMQSGRAKA     91
                     :| |    |::|  | |  |  ||||      | ||| : :: | :|
B_Sequence        50 LL-D-ATVPVLIGTLTAVAVVMVAGI-GVIVSTHRRRQLEKAIRTRA     93
                                        7

#---------------------------------------

============ FINISHED =============
Average Quality (AQ)      13.98 +/- 5.83
Standard score (Z): 10.0
Precise score (Z):  10.5
```

Fig. S16E

```
# 1: A_Sequence: Mbr1 (MC homologue)
# 2: B_Sequence: Cst1 (MntP homologue)
# Matrix: EBLOSUM62
# Gap_penalty: 8.0
# Extend_penalty: 2.0
#
# Length: 79
# Identity:      22/79 (27.8%)
# Similarity:    39/79 (49.4%)
# Gaps:           7/79 ( 8.9%)
# Score: 62.0
#
#
#========================================
                                  2
A_Sequence         1 FWRYFA-GNLASGGAAGATSLCFVYPLD----FA-RTRLAADVGKGSAQR     44
                     |: :|  | |: |  :|:|   |: |:|    |   | :   : | | :
B_Sequence         1 FFGFFQWGMLSLGWLSGSTFRTFIEPVDHWIAFVLLTFIGVKMWKESTEE     50
                           2                   3
                                        3
A_Sequence        45 MLP-DPKNVHIFISWMIAQSVTAVAGLVS     72
                       | |  :| : ::  :| |: | |  :|
B_Sequence        51 AEPLDLTSVKLMLTLSVATSIDAFAAGIS     79
                                        4

#----------------------------------------

============ FINISHED =============
Average Quality (AQ)      13.34 +/- 5.38
Standard score (Z): 9.0
Precise score (Z):  9.1
```

Fig. S16F

```
# 1: A_Sequence: Cmi2 (MC homologue)
# 2: B_Sequence: Aho1 (ILT homologue)
# Matrix: EBLOSUM62
# Gap_penalty: 8.0
# Extend_penalty: 2.0
#
# Length: 142
# Identity:      36/142 (25.4%)
# Similarity:    58/142 (40.8%)
# Gaps:           7/142 ( 4.9%)
# Score: 65.0
#
#
#=======================================
                                  2
A_Sequence         1 QGFLSFWRGNLANVIRYFPTQALNF-AFKDKYKQIFMSGIDKK---TQFG     46
                     | :| : |  : : :       | | ||   |::|| : :  :   ||
B_Sequence         1 QRWLGYIRDKVDSALGRGTVWTLAFVAFISVYREIFETILFYQALWTQVD     50
                                          3
                                  3
A_Sequence        47 KWFLANLASGGAAGATSLCFVYPLDFARTRLAADVGKGNEERQFKGLADC     96
                         | |  |  |   :|  |  | | |  :   :|          |
B_Sequence        51 GQTQAFLFYGIGAAVLALA-VVSLLFFRVGMTLPLGVFFRVTSLVLLVLS     99
                                  4
                                          4
A_Sequence        97 LAKIGKRDGIQGLYQGFAVSVNGIIVYRASYFGCYDTIKGIL    138
                     :  :||  ||  | :   :||  : |    : | | |::|:|
B_Sequence       100 VILLGK--GIAALQEAGLISVMHLAVPTVDWLGVYPTVQGLL    139
                         5

#---------------------------------------

============ FINISHED =============
Average Quality (AQ)     13.51 +/- 5.69
Standard score (Z):      9.0
Precise score (Z):       9.1
```

Fig. S16G

```
# 1: A_Sequence: Rsy1 (MC homologue)
# 2: B_Sequence: Sya2 (TerC homologue)
# Matrix: EBLOSUM62
# Gap_penalty: 8.0
# Extend_penalty: 2.0
#
# Length: 144
# Identity:      28/144 (19.4%)
# Similarity:    48/144 (33.3%)
# Gaps:          50/144 (34.7%)
# Score: 38.0
#
#
#=======================================
                                                                    1
A_Sequence         1 ANVIRYFPTQALNFGFKDKYKKIFLDNVDKRTQFWRYFAGNLASGGAAGA     50
                                   |:|  ::::      :  :   : ||  |  | | |
B_Sequence         1 ------------WVGWK-MWRELRAHGEPEDAE---HMAGKAAPKGFAQA     34
                                  3

A_Sequence        51 TSLCFVYPLDFARTRLAAD--VGKAGAGREFNGLGDCLAKIFKSDGLKGL     98
                           : :  |   :: |  :  ||| ||  |:   :  :  |  | |:
B_Sequence        35 -----AWAVAIADVSMSLDNVLAVAGAAREHPGI--LVIGLVLSVALMGV     77
                                          4                         5
                                  2
A_Sequence        99 YQGFNVSVQGIIIYRA-AYFGI----------------------    119
                         |:  : |   ||| ||||:
B_Sequence        78 --AANLLARVIERYRAVAYFGLIVILYVAGKMIYEGAIDPATGL    119
                                          6

#---------------------------------------

============ FINISHED =============
Average Quality (AQ)     12.70 +/- 5.66
Standard score (Z):      4.0
Precise score (Z):       4.4
```

Fig. S16H

```
# 1: A_Sequence: Rsy1 (MC homologue)
# 2: B_Sequence: Orf9 (NAAT homologue)
# Matrix: EBLOSUM62
# Gap_penalty: 8.0
# Extend_penalty: 2.0
#
# Length: 75
# Identity:      19/75 (25.3%)
# Similarity:    35/75 (46.7%)
# Gaps:           3/75 ( 4.0%)
# Score: 63.0
#
#
#=======================================
                           2                                3
A_Sequence         1 SVQGIIIYRAAYFGIYDTAKGMLPDPKNTHIFVSWMIAQSVTAVAGF-GS     49
                     ::   |:| | |  ::||  |::        | ||:: :|   :  | |
B_Sequence         1 AISSTIVYGARYPSMFDT-MGIIL-TIIAFCFCSWLLFRSAPLLVRFLGQ     48
                           4                   5

A_Sequence        50 YPFDTVRRRMMMQSGRKGAEIMYSG     74
                        : : | | :  |  | | : :|
B_Sequence        49 TGINVITRIMGLILGALGIEFIANG     73
                                 6

#---------------------------------------

============ FINISHED =============
Average Quality (AQ)      12.39 +/- 5.04
Standard score (Z): 10.0
Precise score (Z):  10.0
```

Fig. S16I

```
# 1: A_Sequence: Cfe1 (MC homologue)
# 2: B_Sequence: Bsm1 (NicO homologue)
# Matrix: EBLOSUM62
# Gap_penalty: 8.0
# Extend_penalty: 2.0
#
# Length: 114
# Identity:      26/114 (22.8%)
# Similarity:    46/114 (40.4%)
# Gaps:           8/114 ( 7.0%)
# Score: 67.0
#
#
#=======================================
                                           2
A_Sequence         1 SYRGIFHAFSTIYQQEGFLAFYRGVSLTVLVYMN-LEKIWNGPRDRFSLF     49
                     :|:|| :  |      | :    | :  ||: |: :|| | |    : ||
B_Sequence         1 TYKGIPYVKSLFI---GIIHGLAGSAAMVLLTMSTVEKAWEGL--LYILF     45
                                           3

A_Sequence        50 QNFANVCLAAAVTQTLSFPFDTVKRKMQAQSPYLPHCGGVDVHFSGAVDC     99
                          |      |  :  ||    ||::  : ::   | :   |   :
B_Sequence        46 FGAGTVLGMLCFTTLIGIPFTLSARKIRIHNAFIQITGFISTVF--GIHY     93
                         4                                 5
                                  3
A_Sequence       100 FRQVVKAQGVLGLW    113
                        :   :|:  ||
B_Sequence        94 MYNLGVTEGLFKLW    107


#---------------------------------------

============ FINISHED =============
Average Quality (AQ)      14.43 +/- 5.72
Standard score (Z):       9.0
Precise score (Z):        9.3
```

Fig. S16J

```
# 1: A_Sequence: Cmi2 (NicO homologue)
# 2: B_Sequence: Msp16 (GAP homologue)
# Matrix: EBLOSUM62
# Gap_penalty: 8.0
# Extend_penalty: 2.0
#
# Length: 153
# Identity:      19/153 (12.4%)
# Similarity:    30/153 (19.6%)
# Gaps:          78/153 (51.0%)
# Score: 40.0
#
#
#=======================================

A_Sequence         1 -----------------------------------------QGFLSFWRG      9
                                                                  :|  | |
B_Sequence         1 PRVQMAAGVIVLLVAAAVAVGLGGTKAGRRGQLATRTSRLMEGH-SLWIA     49
                                  3
                         2                                          3
A_Sequence        10 NLANVIRYFPTQALNFAFKDKYKQIFMSGIDKKTQFGKWFLANLASGGAA     59
                      :| :    |    :  :      |  ||    || |   | |: : |
B_Sequence        50 GVAGLGIALP----SVDYLAALTIIIASGAAAATQVGALLLFNVVAFGLV     95
                         4                                          5
                                                                      4
A_Sequence        60 GATSLCFVYPLDFARTRLAADVGKGNEERQFKGLADCLAKIGKRDGIQGL    109
                         :|::   |  |  |:|
B_Sequence        96 EIPLICYLVAPDRTRAMLSAL-----------------------------    116


A_Sequence       110 YQG    112

B_Sequence       116 ---    116


#---------------------------------------

============ FINISHED =============
Average Quality (AQ)     10.38 +/- 5.16
Standard score (Z):      6.0
Precise score (Z):       5.8
```

Fig. S16K

```
# 1: A_Sequence: Cmy1 (MC homologue)
# 2: B_Sequence: Glo1 (DsbD homologue)
# Matrix: EBLOSUM62
# Gap_penalty: 8.0
# Extend_penalty: 2.0
#
# Length: 116
# Identity:      29/116 (25.0%)
# Similarity:    51/116 (44.0%)
# Gaps:          16/116 (13.8%)
# Score: 69.0
#
#
#========================================
                                                   5
A_Sequence         1 SYFGCYDTIKGLLP--NPKQTPFVLSFLIAQAVTTFSGI-LSYPFDTVRR     47
                     :| | :  : |||   :|   | : |::      :|| :   :|   |||
B_Sequence         1 TYIGAF--VAGLLSFLSPCVLPLIPSYITYITGLSFSDLDAEHPTHVVRR     48
                                  1

A_Sequence        48 RMMMQSGEAERQYKGTIDCFFKIYKQEGLKAFFRGAF----SNILRGTGG     93
                     : |: |          :  |  ::   |  | : |:|      ::|  ||
B_Sequence        49 KTMLHS-------LAFVSGFTVVFVLLGASATYIGSFLQQHMELVRKLGG     91
                                  2
                          6
A_Sequence        94 ALVLVLYDKIKELVNL    109
                      |::|    :   || |
B_Sequence        92 ILIIVFGIHVTGLVPL    107
                          3

#---------------------------------------

============ FINISHED =============
Average Quality (AQ)      13.12 +/- 5.64
Standard score (Z):       10.0
Precise score (Z):        9.9
```
