# Supplementary material for: The LysE Superfamily of Transport Proteins Involved in Cell Physiology and Pathogenesis
Source: PLoS One. 2015 Oct 16;10(10):e0137184. doi: 10.1371/journal.pone.0137184 (PMC4608589; doi:10.1371/journal.pone.0137184)
Supplement: S5 Supporting Information — The corresponding PDF file contains the S2–S10 Figs described previously. (PDF) [file pone.0137184.s034.pdf]

Fig. S2A

```
# 1: A_Sequence: Pst1 (2.A.75.1.1 homologue)
# 2: B_Sequence: Bth1 (2.A.76.1.5 homologue)
# Matrix: EBLOSUM62
# Gap_penalty: 8.0
# Extend_penalty: 2.0
#
# Length: 171
# Identity:      57/171 (33.3%)
# Similarity:    87/171 (50.9%)
# Gaps:          20/171 (11.7%)
# Score: 159.0
#=======================================
                         1                        2
A_Sequence         1 LILPLGPQNAFVLN----QGVKRHYHLMTATLCSLSDVVLICAGIFGGSA     46
                     ::|| || : :||:    :|||  |         : | ||:     | ::
B_Sequence         1 ILLP-GPNSMYVLSLAAQRGVKAGYRAACGVF--VGDTVLMVLSAAGVAS     47
                         1                        2

                              3
A_Sequence        47 LLQQSPLLLTVITWAGVAFLLWYGWGALRTAFRRELALA-SGLDIRQS-R     94
                     ||: :||| :|: : | |:||: | | || |:|:    | :| |:|::
B_Sequence        48 LLKANPLLFSVVKYGGAAYLLYIGSGMLRGAWRKLARPADAGADVRRAVD     97
                              3

                               4                                    5
A_Sequence        95 G-RIIATLLAVTWLNPHVYLDTFVVLGSLGSQFPD---TH-ARQWFALGT    139
                     | |     | |: |||   |  | :  |   || |    | |  :  ||
B_Sequence        98 GERPFRKALVVSLLNPKAIL--FFI--SFFIQFVDPSYAHPALSFVVLGA    143
                               4                                    5

A_Sequence       140 VS--ASVLWFFGLALLAAWLA    158
                     ::  || ::   |    | ||
B_Sequence       144 IAQFASFVYLSTLIFTGARLA    164
#---------------------------------------
============ FINISHED =============
Average Quality (AQ)     18.75 +/- 6.96
Standard score (Z):      20.0
Precise score (Z):  20.1
```

Fig. S2B

```
# 1: A_Sequence: Hal1 (2.A.76.1.5 homologue)
# 2: B_Sequence: Oki1 (2.A.77.1.1 homologue)
# Matrix: EBLOSUM62
# Gap_penalty: 8.0
# Extend_penalty: 2.0
#
# Length: 124
# Identity:      33/124 (26.6%)
# Similarity:    63/124 (50.8%)
# Gaps:          12/124 ( 9.7%)
# Score: 107.0
#=======================================
                          2                      3
A_Sequence         1 IGDAVLIFCAYIGIASLIRSSPFLFSLVKMLGALYLLYLGLKILYSTLAK     50
                     ||: :||  : : :| |::  |  : :: :|| |: : :|||  :|
B_Sequence         1 IGNGILIVMSLL-LAYLLKFIPESW-ILGLLG-LFPITVGLKTFFS----     43
                          2                      3

                                           4
A_Sequence        51 KGQEQSAAKEEPEHTFRKALTLSLTNPKA--ILFYVSFFVQFIDMDYAHT     98
                     |  | : ||    |  |  : ::||   |  :  |: ||     :|:::
B_Sequence        44 KEDETAKAKASDAHLIRDVVLMTLTTCSADNLAIYIPFFA---SVDFSYL     90
                                           4

                          5
A_Sequence        99 GVSFAILAVILEMISFCYMTLLIF    122
                      |   :  :||  :||  : :  |
B_Sequence        91 PVILIVFLLILSAVSFTALKITKF    114
                          5
#---------------------------------------
============ FINISHED =============
Average Quality (AQ)      23.76 +/- 7.02
Standard score (Z):       12.0
Precise score (Z):  11.9
```

Fig. S2C

```
# 1: A_Sequence: Asp2 (2.A.75.1.3 homologue)
# 2: B_Sequence: Cth1 (2.A.77.1.2 homologue)
# Matrix: EBLOSUM62
# Gap_penalty: 8.0
# Extend_penalty: 2.0
#
# Length: 144
# Identity:      38/144 (26.4%)
# Similarity:    65/144 (45.1%)
# Gaps:          14/144 ( 9.7%)
# Score: 92.0
#=======================================
                                 2                             3
A_Sequence         1 LRQGLRREHVMPVVLVCALSDAVLLQVGVWGMGGVLLARPEWAQFMRWAG     50
                     :    || |:: :      :  ||  :  :  ||::: | ||   :   |
B_Sequence         1 INANFRRRHIV-IGQYLGFTTIVLASLPGF-FGGLIVPR-EWIGLL---G     44
                                 2                             3

                                                            4
A_Sequence        51 ALFLLMYAAQTAARALRPGQLLVATSGPGTSLRTTLATVVALTWLNPHVY    100
                      | :::   |   | :   |:   ||   :| | :  : : |: |||| |
B_Sequence        45 LLPIIIGFKQLVNRKIETVQVQTVTSFENSSYRNSTFSFL-LSLLNPHTY     93
                                                            4

                                                  5
A_Sequence       100 LDTVVLLGTMATPYPAWGRALFAAGGSLAS-----ALWFLLIGL    139
                         | |         :   || ||  |||     |::||::|:
B_Sequence        94 KVAAVTLANGGDNISIY-IPLF-AGSQLASLSIILAVFFLMVGV    135
                                                  5

#---------------------------------------
============ FINISHED =============
Average Quality (AQ)      16.11 +/- 6.25
Standard score (Z):       12.0
```

Precise score (Z): **12.1**

Fig. S3A

```
# 1: A_Sequence: Efa1 (2.A.77.1.1 homologue)
# 2: B_Sequence: Ptr2 (2.A.106.1.1 homologue)
# Matrix: EBLOSUM62
# Gap_penalty: 8.0
# Extend_penalty: 2.0

# Length: 115
# Identity:      32/115 (27.8%)
# Similarity:    57/115 (49.6%)
# Gaps:          11/115 ( 9.6%)
# Score: 108.0
#=======================================
                            1                                     2
A_Sequence         1 LQNILSALAVYISTSI-DYLFILLIIFSQNHTKKGLRQIFFGQYLGTGIL     49
                          |::|: |:| | |  | :  : |  |::     :|||  |   ::
B_Sequence         1 WNAFTSSVAMIIATEIGDKTFFIAAVLSMKHSRSA---VFFGAILALIVM     47
                            1                                     2

                                         3
A_Sequence        50 VAISLFAAYVL-NFIPQDWIIGLLGLIPIYLGIRVAF------VGEEEEE     92
                       :|     :| ||||:::   | ||: :| | :: :       |:  ||
B_Sequence        48 TVLSTAMGMMLPNFIPKEYTHLLGGLLFLYFGCKLIYDSRQMEAGKTSEE     97
                                         3

A_Sequence        93 EGEVVEKLGSRGTNR    107
                       || |:|  :|  :
B_Sequence        98 LEEVEEELLQQGKKK    112
#---------------------------------------
============ FINISHED =============
Average Quality (AQ)     15.13 +/- 6.54
Standard score (Z):      14.0
Precise score (Z):  14.2
```

Fig. S3B

```
# 1: A_Sequence: Cac2 (2.A.75.1.1 homologue)
# 2: B_Sequence: Mpu4 (2.A.106.1.1 homologue)
# Matrix: EBLOSUM62
# Gap_penalty: 8.0
# Extend_penalty: 2.0

# Length: 139
# Identity:      38/139 (27.3%)
# Similarity:    62/139 (44.6%)
# Gaps:          16/139 (11.5%)
# Score: 91.0
#=======================================
                            1                               2
A_Sequence        1 LSLIVAVGPQNAMLLKYGIRRDHIGLIIVVCALSDVILITSGTAGVGYLV     50
                    | |:  :| :   |      | |  | : : ::| :  :| |:|  ||||
B_Sequence        1 LILLTEIGDKTFFLAMMLAAR-HGKLQVFLASISALFFMTLGSALAGYLV     49
                            1                              2
                                          3
A_Sequence       51 ----ERFPNALEALKYIGAAYLAFFTFTCFRDAFKTKGEAIDVESTSPNS     96
                        |   :::: : :: |     |      || |   |  | :
B_Sequence       50 STSAEMLHSSVKIMDWVAAVLFVLFGAQMLWDARKLHKE--DAKD-----     92
                                          3
A_Sequence       97 TEEVATFDGDGDSTGGVGTEHGSVATATATQRQEIKRSP    135
                     ||||   | |:  |   : ||  | |  | |:: ::||
B_Sequence       93 -EEVAALLG-GE--GARSSSHGERADAEETLREKDEKSP    127

#---------------------------------------
============ FINISHED =============
Average Quality (AQ)      12.36 +/- 5.83
Standard score (Z):       13.0
Precise score (Z):  13.5
```

Fig. S3C

```
# 1: A_Sequence: Hal1 (2.A.76.1.5 homologue)
# 2: B_Sequence: Cmi1 (2.A.106.1.1 homologue)
# Matrix: EBLOSUM62
# Gap_penalty: 8.0
# Extend_penalty: 2.0

# Length: 166
# Identity:      42/166 (25.3%)
# Similarity:    81/166 (48.8%)
# Gaps:          19/166 (11.4%)
# Score: 103.0
#=======================================
                          2                                3
A_Sequence         1 LAVFIGDAVLIFCAYIGIASLIRSSPFLFSL-VKMLGALYLLYLGLKILY      49
                     |:|:||  :::    :|   |  |  ||  : :: :||:   : |:|:||
B_Sequence         1 LSVWIGQLLMLLPKLVG-QYLPPSLGFLTHISIEYVGAVLFFFFGIKLLY      49
                          2                                3
                                                                    4
A_Sequence        50 S--TLAKKGQ-----EQSAAKEEPEHTFRKALTL-SLTNPKAILFYVSFF      91
                     |   :::|       |   | |: |  |::  |   :     :| :|: :
B_Sequence        50 SARNMSRKTDIEVMAEAEEAIEDGERKFKQRNTAWKIFIESGVLTFVAEW      99
                                                                    4
                                                   5
A_Sequence        92 ---VQFIDMDYAHTGVSFAILAVILEMISFCYMTLLIFSGAALAHFLSEK     138
                         ||  :  | |  |  ::|  :   : | : : :  | |:|  :||
B_Sequence       100 GDRTQFATVTLAATKDSLGVMAGGIVGHAICAL-IAVIGGRAIASHISE-     147
                                                   5
                                   6
A_Sequence       139 KRLAKLGNSMVGLLFL     154
                     : :  :|    ||||:
B_Sequence       148 RTITIIG----GLLFI     159
                                   6
#---------------------------------------
============ FINISHED =============
Average Quality (AQ)      18.42 +/- 6.49
Standard score (Z):       13.0
Precise score (Z):  13.0
```

Fig. S4A

```
# 1: A_Sequence: Kor1 (2.A.76.1.5 homologue)
# 2: B_Sequence: Cce1 (2.A.107.1.2 homologue)
# Matrix: EBLOSUM62
# Gap_penalty: 8.0
# Extend_penalty: 2.0

# Length: 105
# Identity:      26/105 (24.8%)
# Similarity:    58/105 (55.2%)
# Gaps:           9/105 ( 8.6%)
# Score: 80.0
#=======================================
                          3                                         4
A_Sequence         1 LYLAYLGINMLRGAWAARRRTAAQAPAQTLSNIHTHDNV-FRHALLLSLS     49
                     : || :||||:: :    | :: :    |:::::| ::: |::  :|:::
B_Sequence         1 ILLAIIGINMIKES----RNSSCEVAVDTVADVNTDNSLSFKNMFVLAVA     46
                          3                                         4
                                                           5
A_Sequence        50 NPKAALFFLSFFIPFVNPRYPHPALSFFILAAVMQTLSMCYLATLALAGD     99
                         ||  :     |:|     ||:||  :  |  ||||  :   :: |:
B_Sequence        47 TSIDAL-AVGITFAFLNVNI-IPAVSF--IGIVTFTLSMIGVRIGSVFGE     92
                                                           5

A_Sequence       100 KLLAK    104
                     |  ::
B_Sequence        93 KFKSR     97
#---------------------------------------
============ FINISHED =============
Average Quality (AQ)     11.53 +/- 4.99
Standard score (Z):      14.0
Precise score (Z):  13.7
```

Fig. S4B

```
# 1: A_Sequence: Hku1 (2.A.77.1.5 homologue)
# 2: B_Sequence: Dvu1 (2.A.107.1.2 homologue)
# Matrix: EBLOSUM62
# Gap_penalty: 8.0
# Extend_penalty: 2.0

# Length: 137
# Identity:      41/137 (29.9%)
# Similarity:    69/137 (50.4%)
# Gaps:          16/137 (11.7%)
# Score: 110.0
#=======================================
                                  3                                  4
A_Sequence         1 -DKWIVGLLGLIPLFIGIKFALSGEDEDETEEIREKIEQDKSKNLLWTVV     49
                      | |:    ||: |:||::  :  |  :||||  ::   | :: |  |::
B_Sequence         1 WDHWLA--FGLL-LYIGVR--MMREAFEETEENDDRC--DPTRGL--TLI     41
                                  3                                  4

                                                        5
A_Sequence        50 LLTIASGGDNLGVYIPYFSSLNWSKIIIVLIIFAIGIAILCELSRSLSKI     99
                     :| :|:  | | | :    ||:   | ||     ||:  |   :  |
B_Sequence        42 MLAVATSIDALAVGL----SLSVLGIDIVTPAIVIGVVCLLFTATGLHLG     87
                                                        5


                                           6
A_Sequence       100 PMVS--EIIEKYEKIIVPVVFIALGIYIMYENGTIQT    134
                      |:|  | : :   :   || | :|: |:||:|   |
B_Sequence        88 RMLSRAESLGRRAALAGGVVLIGIGLRILYEHGVFDT    124
                                           6
#---------------------------------------
============ FINISHED =============
Average Quality (AQ)     14.66 +/- 6.04
Standard score (Z):      16.0
Precise score (Z):  15.7
```

Fig. S4C

```
# 1: A_Sequence: Csp2 (2.A.106.1.1 homologue)
# 2: B_Sequence: Eco2 (2.A.107.1.1 homologue)
# Matrix: EBLOSUM62
# Gap_penalty: 8.0
# Extend_penalty: 2.0

# Length: 152
# Identity:      47/152 (30.9%)
# Similarity:    70/152 (46.1%)
# Gaps:          15/152 ( 9.9%)
# Score: 134.0
#=======================================
                         2                                     3
A_Sequence         1 AALASMTLLSVLMGQAISFLPKHYI----HWAEIALFLGFGLKLIYDASQ      46
                     |   |:  |: |:| ||  : :|||    ||    | |  ||::|| | |
B_Sequence         1 AVFGSVETLTPLIGWAIGSVAQHYIADWDHWIAFTLLLLLGLRMIYGALQ      50
                         2                                     3
                                                         4
A_Sequence        47 MPSQSQGTVIKEAAEAVDQIPQSGNR-----LTKLLARYPQIGIWLQAFS      91
                      | |  |   :::|||  :  ||| |     :   :|    |   :
B_Sequence        51 -PEQPAG---EQSAEAQPESGQSGRRPPSPLMLVAIAFATSIDSMIVGVG      96
                                                         4
                                                   5
A_Sequence        92 MTFLAEWGDRTQISTIALASS-YNVIGVTTGAILGHGICSVIAVIGGKLV     140
                     : || |         | ||::    ||:  |: ||  |     ::|| ::
B_Sequence        97 LAFL-EVNILLTALAIGLATTIMAAIGLRLGSFLGSAIGKRAEILGGLVL     145
                                  5                                  6

A_Sequence       141 AG     142
                      |
B_Sequence       146 IG     147
#---------------------------------------
============ FINISHED =============
Average Quality (AQ)      22.13 +/- 7.43
Standard score (Z): 15.0
Precise score (Z):  15.1
```

Fig. S5A

```
# 1: A_Sequence: Lbr1 (2.A.77.1.1 homologue)
# 2: B_Sequence: Sma2 (2.A.108.2.4 homologue)
# Matrix: EBLOSUM62
# Gap_penalty: 8.0
# Extend_penalty: 2.0

# Length: 207
# Identity:      55/207 (26.6%)
# Similarity:   101/207 (48.8%)
# Gaps:          31/207 (15.0%)
# Score: 138.0
#=======================================
                          1                        2
A_Sequence         1 IDYLIILMVIFGA--TPKRHRFLVYLGDFLGTAILVLTSYLMAVILGFV-      47
                     :: |:|:: : |     |: | | ::  :|| |: || | : |::| |:
B_Sequence         1 VEALLIVLALIGTLKASKQKRGLKWV--YLGAALGVLASVVTAIMLQFLF      48
                          2                        3
                                          3
A_Sequence        48 PA-------EWLLGFLGLIPILM--GVKLLIFGEKEDDDLIENEIQKKTN      88
                     ||       | | | :|:  : |  || : :   | :    :| ::|: |
B_Sequence        49 PALTSGNNREMLEGAVGIFAVFMMIGVGVWLH-SKANISAWQNYMEKQLN      97
                                          4
                          4                                     5
A_Sequence        89 VILK---------VAIITIATCGADNIGIYVPLFTQISPTN--IPILLVT     127
                     :::          :: : :   ||: |  || :   ||  |  : ||
B_Sequence        98 LVMSTGSFVSMFALSFLAVFREGAETILFYVGILPNISLQNLLLGILAAV     147
                          5                                     6
                                                  6
A_Sequence       128 FFIMMTLFCYLGYLLSKIPTIGNILE--KWSRYITAVVYIGLGIYILWES     175
                       :||  | ::     ||| |  : :   |: || |   :|: |: |  :
B_Sequence       148 LILMMLAFVFI-KSSEKIP-IHRVFQLLTWTIYILAFKMLGVSIHALQLT     195
                                                  7
A_Sequence       176 GTL-THL    181
                       | ||:
B_Sequence       196 NALPTHV    202
#---------------------------------------
============ FINISHED =============
Average Quality (AQ)     27.14 +/- 8.23
Standard score (Z):      13.0
Precise score (Z):  13.5
```

Fig. S5B

```
# 1: A_Sequence: Aau1 (2.A.76.1.5 homologue)
# 2: B_Sequence: Eli1 (2.A.108.2.1 homologue)
# Matrix: EBLOSUM62
# Gap_penalty: 8.0
# Extend_penalty: 2.0

# Length: 104
# Identity:      25/104 (24.0%)
# Similarity:    48/104 (46.2%)
# Gaps:           5/104 ( 4.8%)
# Score: 84.0
#=======================================
                                  4                                   5
A_Sequence         1 KKNESALSMFQRGIWVNLLNPKAIVFFLA-FMPQFIRPDQPLLQQYAVLT     49
                     |  :     |  | |   |   |: :  | ::       :  :: :  |
B_Sequence         1 KAGQDRALRFVHGGWTGALVAGALTWLAATYLLDISGAGRESIEAFGSLI     50
                                  2                                   3

                                                           6
A_Sequence        50 ATVIIIDIMVMWFFFAFAARSFQRFTHDQKGQKVLNRVFGCLFVLVGILL     99
                     | :::: : | |      | ::||:  |: | | |:|  | |: | ||:
B_Sequence        51 AALVLLSVGV-WMHGKSQADNWQRYIRDKLG-KALSR--GSLWFLFGIVF     96
                                                           4

A_Sequence       100 AVIH    103
                      |::
B_Sequence        97 LVVY    100
#---------------------------------------
============ FINISHED =============
Average Quality (AQ)     11.31 +/- 5.29
Standard score (Z):      14.0
Precise score (Z):  13.7
```

Fig. S5C

```
# 1: A_Sequence: Aan1 (2.A.106.1.1 homologue)
# 2: B_Sequence: Sso1 (2.A.108.3.3 homologue)
# Matrix: EBLOSUM62
# Gap_penalty: 8.0
# Extend_penalty: 2.0

# Length: 82
# Identity:      28/82 (34.1%)
# Similarity:    47/82 (57.3%)
# Gaps:           2/82 ( 2.4%)
# Score: 104.0
#========================================
                     1                    2                         3
A_Sequence         1 IAAILAMKHARLVIFLGAVSALAVMTVLSAAMGYALPALMPRTYTHYASA     50
                     ||||    :   : |: ||  :|:: : :  :|  |  |:|  |   |||
B_Sequence         1 IAAIYHNIYKNNLPFIYAVLGVAIVLIPTFTLG-KLIYLVPLNYVLLASA     49
                      1                   2                         3

A_Sequence        51 LLFFYFGCRMLKDASSMSGSGVSEELGEVEEE     82
                     :: |||| |::: |   |  |: :: || :||
B_Sequence        50 VILFYFGYRLIRSA-RRSFKGIKKKGGEEKEE     80
#----------------------------------------
============ FINISHED =============
Average Quality (AQ)      13.74 +/- 5.89
Standard score (Z):       15.0
Precise score (Z):  15.3
```

Fig. S6A

```
# 1: A_Sequence: Pre2 (2.A.76.1.5 homologue)
# 2: B_Sequence: Lfr1 (2.A.109.1.3 homologue)
# Matrix: EBLOSUM62
# Gap_penalty: 8.0
# Extend_penalty: 2.0

# Length: 120
# Identity:      39/120 (32.5%)
# Similarity:    55/120 (45.8%)
# Gaps:          16/120 (13.3%)
# Score: 91.0
#=======================================
                         1                                          2
A_Sequence         1 NFWTYLAGLVLIIIVPGPNSLYVLKTSTS-SGTRFGYRAAL--GVFTGDG     47
                     | |  :  ||:|  :   ::  ||   |    |:     :|  |:: |
B_Sequence         1 NDWLIIFSLVVIECLLSVDNAVVLAAQTQVLPTKKWQEESLFYGMW-GAY     49
                         1                                          2
                                                   3
A_Sequence        48 ILIFLSFIGV-ASVIKASPTLFMIVRYLGAAYLLYLGCKILYSTFM--HK     94
                     |  ||  |||   :||     | |:: |||||| ||     |:     ||
B_Sequence        50 IFRFL-IIGVGVYLIK-----FWIIKVLGAAYLFYLAFSFFYNMHQNRHK     93
                                                   3

A_Sequence        95 KSNQDGTDTISIKTENHFTR    114
                     ||:   |  :    :||  |
B_Sequence        94 KSH---THQVKPNKKNHTRR    110
#---------------------------------------
============ FINISHED =============
Average Quality (AQ)     13.27 +/- 5.74
Standard score (Z):      14.0
Precise score (Z):  13.5
```

Fig. S6B

```
# 1: A_Sequence: Osp1 (2.A.77.1.1 homologue)
# 2: B_Sequence: Bsp1 (2.A.109.1.3 homologue)
# Matrix: EBLOSUM62
# Gap_penalty: 8.0
# Extend_penalty: 2.0

# Length: 118
# Identity:      36/118 (30.5%)
# Similarity:    60/118 (50.8%)
# Gaps:          11/118 ( 9.3%)
# Score: 104.0
#=======================================
                          1                                2
A_Sequence         1 SIDYIVIL--VVLFAQNERRKRAVRDIFLGQYIGFTILIAISLLAAFGLT     48
                     |||   :|  :::  : | ||:|::    | |  |     |||:  |
B_Sequence         1 SIDNAAMLASMIMKLKKEDRKKALKYGIFGAYF-FR---GISLI--FASI     44
                          1                                2
                                  3                                  4
A_Sequence        49 LIPQHWIGLL-GLVPIFIGLKVLFEKE--DDDDQEEIIDTNRFTSFILSV     95
                     ||   |: || ||  ::||:   |:|:    : :: ||  | |   |:|:
B_Sequence        45 LIKIWWLKLLGGLYLVYIGISHFFKKKLIKKNSKKNIILRNSFWKIIISI     94
                                  3                                  4


A_Sequence        96 AVIMLAAGGDNLGVYIPY    113
                      :: |    ||:   | :
B_Sequence        95 EIMDLTFSIDNIFATIAF    112
#---------------------------------------
============ FINISHED =============
Average Quality (AQ)      16.41 +/- 6.46
Standard score (Z):       14.0
Precise score (Z):  13.6
```

Fig. S6C

```
# 1: A_Sequence: Pfr1 (2.A.75.1.1 homologue)
# 2: B_Sequence: Rpa3 (2.A.109.1.5 homologue)
# Matrix: EBLOSUM62
# Gap_penalty: 8.0
# Extend_penalty: 2.0

# Length: 103
# Identity:      31/103 (30.1%)
# Similarity:    51/103 (49.5%)
# Gaps:          11/103 (10.7%)
# Score: 112.0
#=======================================
                     3                                              4
A_Sequence         1 YLCWFAWRSFRSALRPQSD--DALTGQGPDAGALRPIVGTTL-ALTWLNP     47
                     ::||  ||  ||  :  :|  |||   |  :|| |  :|  :  :|  :
B_Sequence         1 WVCWKMWRELRSQSQHDADALDALNDDGTASGAPRKTLGQAVWQITLADI     50
                     3                                              4
                                                   5
A_Sequence        48 HVYLDTMVMLGGLANQHPGLTRWAFAGGAMLGSALWFAALGLGARALSRP     97
                      : || :: : | | :|| :         : | ||  | :|| |  :::
B_Sequence        51 SMSLDNVLAVAGAAREHPII--------LVFGLALSIALMGLAASFIAKL     92
                                                   5

A_Sequence        98 LSK    100
                     | |
B_Sequence        93 LQK     95
#---------------------------------------
============ FINISHED =============
Average Quality (AQ)     16.06 +/- 6.58
Standard score (Z):      15.0
Precise score (Z):  14.6
```

Fig. S6D

```
# 1: A_Sequence: Lmi1 (2.A.107.1.1 homologue)
# 2: B_Sequence: Ddo1 (2.A.109.5.1 homologue)
# Matrix: EBLOSUM62
# Gap_penalty: 8.0
# Extend_penalty: 2.0

# Length: 188
# Identity:      48/188 (25.5%)
# Similarity:    94/188 (50.0%)
# Gaps:          29/188 (15.4%)
# Score: 132.0
#========================================

                         2                                 3
A_Sequence         1 LSQALGIGILFGV---VEATTPLIGWLLGSAASRFVASIDHWVAFVLLAG      47
                     |:  : | :|||:   |  : |   | :   ||     |  | | :|:||
B_Sequence         1 LAMGMRIALLFGISWLVALSAPF--WHIN--ASWITGGIS-WQAVILIAG      45
                         2                                 3

A_Sequence        48 LGIHMVWKSFQPLEPDCDDQTDAPYDTGVQLGADGSALRTGRLLPAGLLS      97
                      || ::|||   :    |       :||::   :  : ::   |   ::
B_Sequence        46 -GIFLIWKSVHEIHEKVD-------ETGLE--EEEISKKSSTTLGNAIVQ      85

                         4                                 5
A_Sequence        98 MLLTSVATSIDAM--AVGVTLAFVDVPIGQVALVIGLCTTMMVTLGVML-     144
                     : : ::  | |::  |||:|    | |    ||:| :   :::::|:|:
B_Sequence        86 IAVINLVFSFDSILTAVGMTNGLSDNPTD--ALII-MVIAVVISVGIMML     132
                         4                                 5
                                          6
A_Sequence       145 -GRLLGTLVGRR--AEMLGGIVLIVIGTVILYE--HLA    177
                         :|  : :    ::||   ||:|| ::: |  ||:
B_Sequence       133 FANPVGNFIAKHPSLQILGLSFLILIGFMLIAEGAHLS    170
                                          6
#----------------------------------------
============ FINISHED =============
Average Quality (AQ)     26.22 +/- 7.83
Standard score (Z):      14.0
Precise score (Z):  13.5
```

Fig. S6E

```
# 1: A_Sequence: Npe1 (2.A.108.2.1 homologue)
# 2: B_Sequence: Cte1 (2.A.109.1.3 homologue)
# Matrix: EBLOSUM62
# Gap_penalty: 8.0
# Extend_penalty: 2.0

# Length: 202
# Identity:      57/202 (28.2%)
# Similarity:    91/202 (45.0%)
# Gaps:          19/202 ( 9.4%)
# Score: 125.0
#========================================
                                                                    2
A_Sequence         1 SSFVAAFTILVREGLEAI---LIVIAMITFLAKADRRDVLPYVHGGWIAA      47
                     || :  | ::| ||| ::    ::  |:  | :  |   | |   | : |
B_Sequence         1 SSLLVIFNLIVIEGLLSVDNAAVLATMVLDLPQKQRPAALTY---GILGA      47
                                  1                                 2
                                                   3
A_Sequence        48 -LFAGAGTWAAATWLITISGASRELTEGFGGVFAALVLLWVGIWMH-GKS      95
                      || |   : || :|:     |      |||::  | |:|   | : |
B_Sequence        48 YLFRGLFLFFAA-FLV-----SAWWLRPFGGLY-LLYLVW-NWWNNRGSK      89
                                                   3
                                                            4
A_Sequence        96 NADAWQRYIRD-KLGRALNRRSAWFLFALAFIVVYREVFETILFYAAIWS     144
                     : ||     || :| | ::||   |   : |: :    |     :||:
B_Sequence        90 DGDAMCTEKRDNRLYRFVSRRIGPFWATVLFVEMMDIAFSIDNVFAAVAF     139
                                                            4
                                  5                                 6
A_Sequence       145 QGNGGAVVAGAFAAIAVLAVIAFVMLRHSRTLPIGKFFAYSSALIAVLAV     194
                       |   |  | |  | |:  :|:  :|     |  :  ||   ::||| :
B_Sequence       140 TDNLILVCTGVFIGILVMRFVAYGFIRLMEEYPFLESCAY--IVLAVLGL     187
                                  5                                 6


A_Sequence       195 VL    196
                      |
B_Sequence       188 RL    189
#---------------------------------------
============ FINISHED ============
Average Quality (AQ)     22.32 +/- 7.82
Standard score (Z):      13.0
Precise score (Z):  13.1
```

Fig. S6F

```
# 1: A_Sequence: Ptr2 (2.A.106.1.1 homologue)
# 2: B_Sequence: Lga1 (2.A.109.1.3 homologue)
# Matrix: EBLOSUM62
# Gap_penalty: 8.0
# Extend_penalty: 2.0

# Length: 194
# Identity:      53/194 (27.3%)
# Similarity:    85/194 (43.8%)
# Gaps:          31/194 (16.0%)
# Score: 111.0
#=======================================
                                  1                                 2
A_Sequence         1 GGFWNAFTSSVAMIIATEIGDKTFFIAA---VLSMK-HSRSAVFFGAILA      46
                     |  |    : : |     : |    :||   ||  |   | :: :| :
B_Sequence         1 GQDWMMILTLILMECLLSV-DNAVVLAAQTQVLPTKDEQRKSLVYG-LWG      48
                                  1                                 2
                                                   3
A_Sequence        47 LIVMTVLSTAMGMMLPNFIPKEYTHLLGGLLFLYFGCKLIYDSRQMEAGK      96
                       :   :   :|  | ||       |||||  ||   |  || |
B_Sequence        49 AYLFRFIVIGIGTYLINFWE---IKLLGGLYLLYLVYKYFYDVRHP----      91
                                                   3
                                                                      4
A_Sequence        97 TSEELEEVEEELLQQGKKKADLEEGSRSNRPPSKKQMGWNQVV-IQSLTL     145
                           :| ::  :  ||:|  :: |:: :      : |  |: |:|: :
B_Sequence        92 -----AQVAKK--EAAKKEAHKKKNSKTRK--HHLSLFWRTVISIESMDI     132
                                                                      4
                                                   5
A_Sequence       146 TFVAEWGDRSQIATIALAASKNPIGVTIGGCVGHSLC-TGLAVV    188
                      |  :    | :|  ||| | ||: | :|| :|  ||  |:| |
B_Sequence       133 VFSID----SVLA--ALAMSNNPVVVLVGGMIG-ILCMRGVAEV    169
                                                   5
#---------------------------------------
============ FINISHED =============
Average Quality (AQ)      13.76 +/- 5.99
Standard score (Z):       16.0
Precise score (Z):  16.2
```

Fig. S7A

```
# 1: A_Sequence: Spl1 (2.A.75.1.1 homologue)
# 2: B_Sequence: Ogr1 (2.A.95.1.3 homologue)
# Matrix: EBLOSUM62
# Gap_penalty: 8.0
# Extend_penalty: 2.0

# Length: 157
# Identity:      50/157 (31.8%)
# Similarity:    74/157 (47.1%)
# Gaps:          14/157 ( 8.9%)
# Score: 124.0
#=======================================
                          1                         2
A_Sequence         1 LIVAIGAQNTFV-LTQGIRKQHRFVVALICSL-CDAFLISAGVAG--LGS     46
                     :|  ||    || ||||:  : |  :|:  :|   | |::  | |  |
B_Sequence         1 IIDPIGLTPLFVALTQGMPDRQRRAIAVRATLVAVAVLLAFAVFGEALLG     50
                          1                         2

                                  3
A_Sequence        47 LIEQSPTLLRLAGGGGALFLFIYGLKCLFSALQAEQELGETESNPTSRRQ     96
                      :  |    |:|||   : ||:  |  ||   || :|  :|  :||
B_Sequence        51 FVGISMAAFRIAGG---VLLFLTALDMLFQRRQARRE--DTADDPTEDPS     95
                                  3

                           4                                        5
A_Sequence        97 VILTILAI-TLCNPNVYLDTVVLLGGISATFVGQGRYLFGAGAISMSFIW    145
                     |    |||  |  |   : |::|| | | :  |    | |     :: ::
B_Sequence        96 VF--PLAIPLLAGPGA-IATIILLTGQSESVAGFAAVL-GVMVAVLTIVF    141
                           4                                        5

A_Sequence       146 FFILSYG    152
                      | |: |
B_Sequence       142 LFFLAAG    148
#---------------------------------------
============ FINISHED =============
Average Quality (AQ)     20.31 +/- 7.39
Standard score (Z):      14.0
Precise score (Z):  14.0
```

Fig. S7B

```
# 1: A_Sequence = Pag1 (2.A.76.1.2 homologue)
# 2: B_Sequence = Cba1 (2.A.95.1.4 homologue)
# Matrix: EBLOSUM62
# Gap_penalty: 8.0
# Extend_penalty: 2.0

# Length: 167
# Identity:      44/167 (26.3%)
# Similarity:    79/167 (47.3%)
# Gaps:          15/167 ( 9.0%)
# Score: 115.0
#=======================================
                          1                                 2
A_Sequence         1 ALVHLVALMSP-GPDFFFVS-QTAASRSRKEAMMGVLGITLGIVVWAGV-     47
                     : : |:||::| |   ||:|  |  :   |   : :  |::  ||  |:
B_Sequence         1 SFISLLALINPIGAIPFFISLTTQQTEEEKRHTIKIAAISVATVV--GIS     48
                          1                                 2

                                           3
A_Sequence        48 ALMGLHLILEKMAWLHQVIMVGGGLYLLWMGWQLMCSARQRHKQPQQDEP     97
                     ||:|   |:|        : ||||| :: |   :: :   | |   ::|
B_Sequence        49 ALLG-QQIIEFFNISVASLQVGGGLIMIMMAMNMLNAQTSRTKATPEEED     97
                                           3

                                    4
A_Sequence        98 VVELPKRGMSFLKGLLTNLSNPKAIIYFGSVFSLFVGDDVGSAERWGLFL    147
                       |  |  :: :   |  |: |      ||: :: |    |  : |   |
B_Sequence        98 EAE-AKASIAVVPLALPLLTGP------GSISTVIV--YAGKTQHWYQLL    138
                                    4

                          5
A_Sequence       148 LIIGETFAWFALVAAIF    164
                     :::|   |  |:|  :|
B_Sequence       139 ILVGIGVALGAVVYIVF    155
                          5
#---------------------------------------
============ FINISHED =============
Average Quality (AQ)     17.31 +/- 6.50
Standard score (Z):      15.0
Precise score (Z):  15.0
```

Fig. S7C

```
# 1: A_Sequence: Msp1 (2.A.77.1.4 homologue)
# 2: B_Sequence: Orf7 (2.A.95.1.4 homologue)
# Matrix: EBLOSUM62
# Gap_penalty: 8.0
# Extend_penalty: 2.0

# Length: 152
# Identity:      42/152 (27.6%)
# Similarity:    68/152 (44.7%)
# Gaps:           9/152 ( 5.9%)
# Score: 97.0
#=======================================
                     1                             2
A_Sequence         1 FLLAAFFANPEFRAKDVVLGQYLGFIVLLT--ISSLAYFVQF--IIPSNW      46
                     ||      ||: | |         |:|| |  |:    |  |   :|:
B_Sequence         1 FLAVTTGQNPQKRRKTARKASLTAFVVLTTFAIAGTFIFKMFGITLPAFE      50
                     1                             2
                         3                                          4
A_Sequence        47 ISLLGVIPIMIGIRSFLHLKK-PQTDYSGENRDFSKYKEGQMMLPVTLVT      95
                     |:  ||| ::||:   |  |: |  : |||  : :  ||   ::|: :
B_Sequence        51 IA-GGVILLLIGL-DMLEAKRSPTQESSGETAEAAS-KEDVGIVPLGIPM      97
                         3                                          4
                                                   5
A_Sequence        96 LANGGDNLGVYMPLFASMGPFDL-FLTAIIFLIMVGVWCFLGYKLVNNRV     144
                     ||  |    | : :  :  |: :  :  | | :  |  :  ||      |:
B_Sequence        98 LAGPGAITSVMVLVGQAQNPWQVGTIIAAIAITAVSCYVVLGAATRVARI     147
                                                   5

A_Sequence       145 LG    146
                     ||
B_Sequence       148 LG    149
#---------------------------------------
============ FINISHED =============
Average Quality (AQ)     13.03 +/- 5.85
Standard score (Z):      14.0
Precise score (Z):  14.4
```

Fig. S7D

```
# 1: A_Sequence: Asu1(2.A.107.2.1 homologue)
# 2: B_Sequence: Csh1(2.A.95.1.5 homologue)
# Matrix: EBLOSUM62
# Gap_penalty: 8.0
# Extend_penalty: 2.0

# Length: 124
# Identity:      33/124 (26.6%)
# Similarity:    64/124 (51.6%)
# Gaps:           6/124 ( 4.8%)
# Score: 108.0
#=======================================
                                  3                                 4
A_Sequence         1 YISEFDHWIA-FALLCVIGINMIKMSVTNENSDDDPSD--FSL-RHLTML     46
                     ::||    ||   :| :| : |:     : |  | |:|  | :   : ::
B_Sequence         1 HLSETSLGIAGGVILFLIALRMV-FPAPHGNGADHPADEPFVVPLAIPLI     49
                                  3                                 4

                                                   5
A_Sequence        47 GVATSIDALAMGVSFAFLKVNIWTAAAIIGITTTILSL-FGVKAGHWLGD     95
                        :::  : : ||    ::  | ||  : :    |:| |  |  ||||:
B_Sequence        50 AGPSALATVLLLVSREPARLWEWVAALALTMVVCALTLAFAEKISHWLGE     99
                                                   5
                                  6
A_Sequence        96 RIHKQAELLGGIILIAMGVKVLIE    119
                     |:    | | |::| |: |::|::
B_Sequence       100 RVTTAFERLMGLVLTAIAVQMLLD    123
                                  6
#---------------------------------------
============ FINISHED =============
Average Quality (AQ)     16.70 +/- 6.06
Standard score (Z):      15.0
Precise score (Z):  15.1
```

Fig. S7E

```
# 1: A_Sequence: Gka1 (2.A.109.1.5 homologue)
# 2: B_Sequence: Dgi1 (2.A.95.1.5 homologue)
# Matrix: EBLOSUM62
# Gap_penalty: 8.0
# Extend_penalty: 2.0

# Length: 106
# Identity:      29/106 (27.4%)
# Similarity:    52/106 (49.1%)
# Gaps:           6/106 ( 5.7%)
# Score: 93.0
#========================================
                         1                                 2
A_Sequence         1 LIIGIDVILGGDNAVVIALAS-RNLPEQKRNVAIIVGTALAIAVRIVLTV     49
                     | : : :|: |   | : ::  |  | :::   |      |:|: |:
B_Sequence         1 LAVPLFLIMDGLGNVPVCMSMLRRFPPRRQQRIIFRELCFALAISILFCF     50
                         1                                 2

                                          3
A_Sequence        50 AVVWLLTI----P-FLQLAGGVVLFWIALKLIGQKDEKPTMIKAEPSLWK     94
                        |||      |  |:|||||||| |:::::   : | |    :||
B_Sequence        51 FGDWLLKFLGLGPSTLRLAGGVVLFVISMRMVFPDESKETADPEDPSALA    100
                                          3

                         4
A_Sequence        95 AIQTIV    100
                     | :  :
B_Sequence       101 AEEPFI    106
                         4
#----------------------------------------
============ FINISHED =============
Average Quality (AQ)     12.39 +/- 5.29
Standard score (Z):      15.0
Precise score (Z):  15.2
```

Fig. S8A

```
# 1: A_Sequence: Aur1 (2.A.76.1.2 homologue)
# 2: B_Sequence: Bco1 (2.A.113.1.9 homologue)
# Matrix: EBLOSUM62
# Gap_penalty: 8.0
# Extend_penalty: 2.0

# Length: 209
# Identity:      52/209 (24.9%)
# Similarity:    93/209 (44.5%)
# Gaps:          18/209 ( 8.6%)
# Score: 128.0
#=======================================
                         1                                          2
A_Sequence         1 FIALITLMFIQFCALITPGPDFFLVSQTAISRSRREAVFVVLGITVGVMF      50
                     ||::: | |:         ||   :  |  |||:: :   : |:  |:
B_Sequence         1 FISVLALGFVLGIKHAIE-PDHIIAVSTIASRSKKLSQSSLAGVFWGIGH      49
                         1                                          2

                                                   3
A_Sequence        51 WAILALMGLNIIFEK----MAWLKQILLVIGGIYLCWLGFQMLRSAFSKQ      96
                      | | ::|: ::  |      |   :  :: || | :||   | ||| :
B_Sequence        50 TATLFIVGICLLIIKGEIPEKWAMSLEFLV-GIMLVYLGITTL-SAFKRV      97
                                                   3

                                                           4
A_Sequence        97 KVQNTNTPIDLPKTETKF-FLKGLLTNLSNPKAVIYFGS-VFSLFLANPA     144
                     ::   |     |  :  : ::| :     :  |    ||    |   :
B_Sequence        98 RI---NHHYHEPGHKRNYSYIKSVCIGFVHGLA----GSGAMVLLTMSTV     140
                                                           4

                                          5
A_Sequence       145 LDHVHSLLFIII-AVETLIWFLFVVFVFSLPSFKSAYQ-NVAKWIDGVSG     192
                        | | ::|:|  : |:   ||   :  :|   || :  | | :  ::|
B_Sequence       141 KSVVESAIYILIFGIGTIFGMLFFTTILGIPFIISAKKVEVNKTLTQITG     190
                                          5

                           6
A_Sequence       193 GIFTAFGIY    201
                      | | ||||
B_Sequence       191 AISTVFGIY    199
                           6
#---------------------------------------
============ FINISHED =============
Average Quality (AQ)      23.09 +/- 7.61
Standard score (Z):       14.0
Precise score (Z):  13.8
```

Fig. S8B

```
# 1: A_Sequence = Acy3 (2.A.77.1.4 homologue)
# 2: B_Sequence = Gar1 (2.A.113.1.9 homologue)
# Matrix: EBLOSUM62
# Gap_penalty: 8.0
# Extend_penalty: 2.0

# Length: 195
# Identity:      55/195 (28.2%)
# Similarity:    94/195 (48.2%)
# Gaps:          23/195 (11.8%)
# Score: 129.0
#========================================
                          1                                        2
A_Sequence        1 NIITPILTGV-FAFI-ATNIDDIVILLVFFSQVNEN--FRPWQIVMGQYL     46
                    : :  :|||: |  | | ::| || :  |  | |:|     :    |
B_Sequence        1 DFLAAVLTGIMFGIIHAFDVDHIVAMATFSEQKNKNKQILTYAFKWGTGH     50
                          1                                        2

                                                          3
A_Sequence       47 GFTILVIFSLPGFFGGLILPPAWIG----LLGLIPIGIGISSLVNKEKEQ     92
                    |  ||::  :   | |  ||  ::     :|:: | :|:  ||   ::
B_Sequence       51 G-GILLLLGMLLIFIGFQLPNWFVHYSEIMVGVLLIYLGVKLLVLLHRKG     99
                                                          3

                                                  4
A_Sequence       93 LADVPEEIISPATSINNYSLTPQIYTVAAITVANGSDNISIYIPLFSSIS    142
                       ||| :   | |:| :  || ::    | : :|    :  : |  ::
B_Sequence      100 TFSVPESLDLAARSLNKHDHTP-LF----IGMLHGVAGSAPLLALLPNML    144
                                                  4

                                 5                             6
A_Sequence      143 FNSFLLIIGLFFF--LLGVWC--YV--AYQL-THQK--KVADFFT    178
                       ||| | ||    | |::|  |:  :||:   ||  |:|  ||
B_Sequence      145 ETQFLLHISLFSIGCLFGMFCFGYIFGSYQVYIKQKKEKLAKAFT    189
                                 5                             6
#----------------------------------------
============ FINISHED =============
Average Quality (AQ)      18.67 +/- 7.32
Standard score (Z):       15.0
Precise score (Z):  15.1
```

Fig. S8C

```
# 1: A_Sequence: Bdi1 (2.A.109.1.5 homologue)
# 2: B_Sequence: Cul1 (2.A.113.1.9 homologue)
# Matrix: EBLOSUM62
# Gap_penalty: 8.0
# Extend_penalty: 2.0

# Length: 196
# Identity:      55/196 (28.1%)
# Similarity:    83/196 (42.3%)
# Gaps:          19/196 ( 9.7%)
# Score: 117.0
#=======================================
                                  1                        2
A_Sequence         1 ASALGKVLMIDLVLAGDNAVAVGLAAAALPQEQRRKAILIGLA-----AA      45
                     |  :| :| :   |  |: |||  :  || :    :   ||       |
B_Sequence         1 AMGIGILLGLRHALDADHVVAV--STMALEERNLLRGGWIGFCWGVGHAL      48
                                  1                        2
                                          3
A_Sequence        46 VVMRIGLALIT--VQLLAIVGLLLAG--GFLLLWVCWKMWRELREQATHD      91
                     |:   | |||   ::|  :||  | |  | :|: :    || :|    |
B_Sequence        49 VLFLFGGALILSGIRLPEVVGRWLEGGVGVMLILIALGSWRRMRRSKLHI      98
                                          3
                                                            4
A_Sequence        92 QAEAEAEIERAMAIEHGGGPSPEEL----GLK-RKTFGAALIQIMIADLT     136
                         : : ||     |    ||  |    | |   :|    :  :
B_Sequence        99 HVH-QHDGERYHTHFHVHDDSPRHLEKHHGWKGSHSFLIGTVHGLAGTGA     147
                                                            4
                                          5
A_Sequence       137 MSLDNVLAVAGASHEHPWIMVFGL--ILSIALMGLAATFIAKLLNR     180
                     : :  : ||:       ::  |||  |||: |  |: | | |||||
B_Sequence       148 VMVLTIAAVSDPLQRIAYLASFGLGTILSMTLFSLSLTLITKLLNR     193
                                          5
#---------------------------------------
============ FINISHED =============
Average Quality (AQ)     18.97 +/- 7.06
Standard score (Z):      14.0
Precise score (Z):  13.9
```

Fig. S8D

```
# 1: A_Sequence: Mfo1 (2.A.95.1.4 homologue)
# 2: B_Sequence: Orf5 (2.A.113.2.1 homologue)
# Matrix: EBLOSUM62
# Gap_penalty: 8.0
# Extend_penalty: 2.0

# Length: 128
# Identity:      33/128 (25.8%)
# Similarity:    66/128 (51.6%)
# Gaps:          11/128 ( 8.6%)
# Score: 109.0
#=======================================
                                                                    4
A_Sequence         1 FKIAWDMLHAEMSKTKHSPREEIDMRMGSVAVVPLAIPLLAGPGAITTTI     50
                     :: | :  |||  : : : ||   :  | : :  |   |:  |||||  :
B_Sequence         1 YQDAHERQHAEDIRRRFAGRE---VTTGQIILFGLTGGLIPCPGAITVLL     47
                                                                    4
                                                   5
A_Sequence        51 ILME-KAQSLANKTIVISSI--ILTMIVSGLILSASDIVVKKLKVSGINA     97
                     : :: |  :| :  ::  ||   |||: || :::|  :   : : ||  :
B_Sequence        48 LCLQLKRVALGSVLVLCFSIGLALTMVASG-VIAALSVKYAERRFSGFGS     96
                                                   5
                                          6
A_Sequence        98 IVR----IMGLILAAISVQIIFSGAYGL    121
                     :||      ||::  : : :  || : |
B_Sequence        97 LVRKAPYASGLVILCVGLYVALSGWHSL    124
                                          6
#---------------------------------------
============ FINISHED =============
Average Quality (AQ)     18.86 +/- 6.66
Standard score (Z):      14.0
Precise score (Z):  13.5
```

Fig. S9A

```
# 1: A_Sequence: Hgr1 (2.A.76.1.5 homologue)
# 2: B_Sequence: Ssp3 (2.A.116.1.7 homologue)
# Matrix: EBLOSUM62
# Gap_penalty: 8.0
# Extend_penalty: 2.0

# Length: 188
# Identity:      60/188 (31.9%)
# Similarity:    91/188 (48.4%)
# Gaps:          27/188 (14.4%)
# Score: 138.0
#=======================================
                          1                                 2
A_Sequence         1 IGIVLLPGPNSLFVLSVATA-RGVRVGYHAACGVF----LGDSILL-LFT      44
                     : | :: ||  :  : :||| | |||      ||     || :|:| : |
B_Sequence         1 LAITMMAGPQIMSAVILATAQRAVRVSLGFVTGVLIATSLGVAIMLGIAT      50
                          1                                 2

                                           3
A_Sequence        45 ALGAA---SLLRGYPALFMVVKYVGAAYLFWVGMNLAWSAWRKWRAAGIA      91
                     ||| |          ::  |::||  | |      ||  | | ||
B_Sequence        51 ALGGAVDFGSSGDKSSVGRVIQYVLVALLI-----LA--ALRNWR----K      89
                                           3

                                                            4
A_Sequence        92 TQLVEPTA-LAAAQSAHLLAPFQRALVISLLNPKAILFLLSFFVQFIDPA     140
                      : |||   | |  ||     |:  |:: || |  :: :|:  |  :|
B_Sequence        90 RETVEPPKWLHALMSADTRKAFETGLLVVLLMPSDLMVMLTVGVH-LDQG     138
                                                            4

                                           5
A_Sequence       141 YDT--PAIPFLILSVIVMAFSAVYLSVLIVAGARLADA     176
                     : :   |:||: |: :| | : : | ||:  | | | |
B_Sequence       139 HSSFVDALPFIALTTLVAA-TPLLLRVLL--GRRAASA     173
                                           5

#---------------------------------------
============ FINISHED =============
Average Quality (AQ)     26.84 +/- 7.65
Standard score (Z):      15.0
Precise score (Z):  14.5
```

Fig. S10A

```
# 1: A_Sequence: Btr2 (2.A.76.1.2 homologue)
# 2: B_Sequence: Cba1 (5.A.1.2.1 homologue)
# Matrix: EBLOSUM62
# Gap_penalty: 8.0
# Extend_penalty: 2.0

# Length: 196
# Identity:      52/196 (26.5%)
# Similarity:    90/196 (45.9%)
# Gaps:          24/196 (12.2%)
# Score: 134.0
#=======================================
                                                   2
A_Sequence         1 YVCRKAMADSRRNAMLGALGIALG--VGFWAVIVLFGLT--FLNHTIPNF     46
                     |:    : :  ::  | ||   ||  :||  : ::||:   |:      :
B_Sequence         1 YITGTTLEEELQDKKLFALSRTLGFVLGFTIIFMIFGILAGFVGQAFIRY     50
                                                   2
                                  3
A_Sequence        47 QFYLMLLGGSYLAYCGIKMVQVRKSVEIDENLKSQANEKSPL----W-KE     91
                     :  |  :||  :   |: || : |     | |  | | :||     |
B_Sequence        51 RNVLTKIGGIIIVLFGLNMVGLLKL----EFLNKQRNVRSPKEVKNWFSS     96
                                  3
                         4                                          5
A_Sequence        92 ILGGLAINLS-NPKVVVFFSSVL--AGYVANISAFKDILAVLAILMGSTL    138
                     || |:|      | :     ::|   |  | :|  | |: :||  :|
B_Sequence        97 ILMGMAFAAGWTPCIGPVLGTILIYVGTTATVS--KGIILLLAYSIG-LA    143
                         4                                          5
                                                           6
A_Sequence       139 IWFWTVAILFSQNKIRRFYAKNNR---YLDNAAGVVFILFGLKLIY    181
                     | |   |:|   |:  :|  |: :   |:   :||| |: |: :::
B_Sequence       144 IPFLLTALLI--NQFSKFLMKSEKVLPYIVKISGVVIIVVGVLIVF    187
                                                           6

#---------------------------------------

============ FINISHED =============
Average Quality (AQ)      23.74 +/- 7.86
Standard score (Z):       14.0
Precise score (Z):  14.0
```

Fig. S10B

```
# 1: A_Sequence = Sne3 (2.A.106.1.2 homologue)
# 2: B_Sequence = Orf5 (5.A.1.2.1 homologue)
# Matrix: EBLOSUM62
# Gap_penalty: 8.0
# Extend_penalty: 2.0

# Length: 161
# Identity:      52/161 (32.3%)
# Similarity:    82/161 (50.9%)
# Gaps:          22/161 (13.7%)
# Score: 130.0
#=======================================



                          1                                         2
A_Sequence         1 LLGIILAFLIVDGIAILAGEWITDIAPRELIKMLSGAIFI-IFGLVTLIF     49
                     |:|  : |: :   : | | :      ::|:: : ||||| ||||: | |
B_Sequence         1 LVGFSVIFIFLGYSSSLVGTFFYQY--QDLLRQI-GAIFIVIFGLMILGF     47
                          2                                         3
                                                  3
A_Sequence        50 RNKREEIK-TKYHFEN-P--FYSGFI--LIFVSEWGDKTQIATG---LFA     90
                        :  :|  |  |:| |  ::  |:  | | : |   |   ||   : |
B_Sequence        48 FTPKFLMKEKKLQFKNRPAGYFGTFLIGLAFAAGWTPCTGPITGAVFMMA     97
                                                  4
                                          4
A_Sequence        91 TQYNG------LMVLTGVIIALSLLSVIAIYSGKFISDKVTRETLTKLTG    134
                      |  |      |: : |  |   |||:  |   |:|  |  | |:||: |
B_Sequence        98 AQNPGSGMWYMLVYVLGFAIPFFLLSIF-ITRVKWI-QKYNR-TITKVGG    144
                                 5
                          5
A_Sequence       135 FLFISMGVLFF    145
                     :| |::|:| |
B_Sequence       145 YLMIALGILLF    155
                          6
#---------------------------------------
============ FINISHED =============
Average Quality (AQ)     26.20 +/- 7.85
Standard score (Z):      13.0
Precise score (Z):  13.2
```

Fig. S10C

```
# 1: A_Sequence: Cac1 (2.A.107.2.1 homologue)
# 2: B_Sequence: Dsp2 (5.A.1.2.1 homologue)
# Matrix: EBLOSUM62
# Gap_penalty: 8.0
# Extend_penalty: 2.0

# Length: 154
# Identity:      47/154 (30.5%)
# Similarity:    79/154 (51.3%)
# Gaps:          14/154 ( 9.1%)
# Score: 146.0
#=======================================
                           2                                 3
A_Sequence          1 FAISFGFFQFLCTFIGAYSGFLFNTYITYVPQIIGGMIIAFVGAFM---I     47
                      | : |    |   |  :: |  |: |   : :::||::|| :| ||   |
B_Sequence          1 FILGFSIIFFALGFSASWVGSFFSEYRDLI-RMLGGVLIAVMGLFMLGLI     49
                           2                                 3
                                                    4
A_Sequence         48 KEGFDNKEEKLLLNFKMYFVLGISVSIDAAVVGFT-MFNKISSNYVILGD     96
                      | ||  ||::| :  | :  || ||   |   |:|     |  : : |
B_Sequence         50 KPGFMMKEKRLEVGRKRWGYLGSSVIGMAFAAGWTPCVGPILVSVLALAA     99
                                                    4
                                   5                                  6
A_Sequence         97 S------VFIGIVTLILSIIAFIISRYLKRIQLVCKYADYI---GGIILV    137
                      |       :|   ||  :|  ||:: :| | : : ||:: :   || ::|
B_Sequence        100 SNPSAGLAYITAYTLGFAIPFFIMAFFLGRTRWILKYSNSLMKAGGALMV    149
                                   5                                  6


A_Sequence        138 IFGL    141
                      :||:
B_Sequence        150 VFGV    153

#---------------------------------------
============ FINISHED =============
Average Quality (AQ)      28.64 +/- 7.96
Standard score (Z):       15.0
Precise score (Z):  14.7
```

Fig. S10D

```
# 1: A_Sequence: Pfu1 (2.A.95.1.4 homologue)
# 2: B_Sequence: Dto1 (5.A.1.2.1 homologue)
# Matrix: EBLOSUM62
# Gap_penalty: 8.0
# Extend_penalty: 2.0

# Length: 213
# Identity:      57/213 (26.8%)
# Similarity:   104/213 (48.8%)
# Gaps:          25/213 (11.7%)
# Score: 140.0
#=======================================
                          1                                         2
A_Sequence         1 GLFAITNP--IGAVPVFIAVTRNLSPEKRKEIARKTSTTVMVTLLVFALV      48
                     || :  :|  :  :| : :    || :: ||  |:|   | ::  || :
B_Sequence         1 GLLSFFSPCILPLIPAYFSFITGLSLDELKENKRQTRQKVFLS-TVFYVA      49
                          1                                         2

                                                            3
A_Sequence        49 G-EWIFKFFGASTDAFS------------IAGGIILFRMSLEMLSGKLSS      85
                     |  :||  ||||                 | |||||    | :| | ::
B_Sequence        50 GFSFIFILFGASASFLGGLASQYAWVVRYIGGGIILV-FGLHLL-GIINI      97
                                                            3

                                                   4
A_Sequence        86 VKIS-EEEEHISEEAVTLEEVAIIPLAIPLLSGP--GAITTTMLYMAKSS     132
                        : |:: |: |: : |    :| :|      |  | :  ::| :| :
B_Sequence        98 KGFNFEKKIHVKEKPLHLMGTFVIGMAFGAGWSPCIGPLLGSILIVAGNQ     147
                                                   4

                                  5
A_Sequence       133 TMIEKSIVLLVVVAIGITV-WIILSA-ANRIHQ--KLGTIGIKVMTRMMG     178
                       : | : || | : |: | ::|||   | | :  |  |  |:|: :: |
B_Sequence       148 ETVLKGVFLLAVYSAGLAVPFLILSVFINSILEIMKRATKFIRVLNKISG     197
                                  5

                          6
A_Sequence       179 LILASMAVQMVIN    191
                     ::| :: : :| :
B_Sequence       198 ILLIAIGLLLVFD    210
                          6
#---------------------------------------
============ FINISHED =============
Average Quality (AQ)     22.82 +/- 7.67
Standard score (Z):      15.0
Precise score (Z):  15.3
```

Fig. S10E

```
# 1: A_Sequence: Sni1 (2.A.116.1.4 homologue)
# 2: B_Sequence: Psp5 (5.A.1.2.1 homologue)
# Matrix: EBLOSUM62
# Gap_penalty: 8.0
# Extend_penalty: 2.0

# Length: 194
# Identity:      46/194 (23.7%)
# Similarity:    97/194 (50.0%)
# Gaps:          15/194 ( 7.7%)
# Score: 117.0
#========================================
                                     2                                  3
A_Sequence        1 KPRPTSLAFLAGWVVGLVGLTVVFIEASSLAGGEQHTRPAWMSWVRIILG      50
                    |  |  |  ::  |: ::| :::|:                |  |: | |
B_Sequence        1 KNSPNKLTVISQTVLFILGFSILFVLLGISVSTVSRLLSEHMRLVQQIGG      50
                                     2                                  3
                                                                  4
A_Sequence       51 AALIVFGVYRF-VTRHR--HTEQPRWMRPFAKLTPG-RAG--LTGVAVAV      94
                    | ::|||::   : | :  ::|: |:: |    :|| :||  : |:| ||
B_Sequence       51 ALIVVFGLHMTGLLRIKLLYSEK-RYL-PSG--SPGKKAGALVLGMAFAV      96
                                                                  4
                                                                        5
A_Sequence       95 VRPEVLALVATAGLEIGAGGLSTAGAWTCGVLFIAVAASTVAIPVLAYAI     144
                         :  : :: | | || ::| |    ||| ::: |  :|:| |  |:
B_Sequence       97 GWTPCIGPILSSIL-IYAGSMATLGK---GVLLLSMYALGLAVPFLLSAV     142
                                                                        5
                                                     6
A_Sequence      145 AGERLDPTMARIKDWMDRNLGAMEAVVLVVIGLMVIEKGISSLS     188
                      : |   : ::   : : :     ||::::|::|    :   |
B_Sequence      143 LIDNLTAYLRKVTKHLPK-ISVASGVVMMLMGVLVFTNQLEVFS     185
                                                     6
#---------------------------------------
============ FINISHED =============
Average Quality (AQ)      21.78 +/- 7.27
Standard score (Z):       13.0
Precise score (Z):  13.1
```
