# Supplementary material for: The LysE Superfamily of Transport Proteins Involved in Cell Physiology and Pathogenesis
Source: PLoS One. 2015 Oct 16;10(10):e0137184. doi: 10.1371/journal.pone.0137184 (PMC4608589; doi:10.1371/journal.pone.0137184)
Supplement: S6 Supporting Information — The corresponding PDF file contains the S12–S15 Figs described previously. (PDF) [file pone.0137184.s035.pdf]

Fig. S12A

```
# 1: A_Sequence: Ssp2 TMS #1-3 (Q2JWH3 ; 2.A.106 homologue)
# 2: B_Sequence: Ssp2 TMS #4-6
# Matrix: EBLOSUM62
# Gap_penalty: 8.0
# Extend_penalty: 2.0
#
# Length: 118
# Identity:      30/118 (25.4%)
# Similarity:    39/118 (33.1%)
# Gaps:          29/118 (24.6%)
# Score: 111.0
#
#
#=======================================
                                                    1  ExGD(K/R)(T/S)
A_Sequence         1 ----------------------MWAGFASSLLLVTVAEFGDKTFFTPLIL     28
                                            ||     :  |  :|||||||    : |
B_Sequence         1 EEEEEALRLVEQAEAKGAGRGGAWAVVWEAFSLTALAEFGDKTQIATVSL     50
                                                    4  ExGD(K/R)(T/S)
                                  2                                  3
A_Sequence        29 AMRHPRRWVFLGTWLALAAMTLLAVVAGKVLFELLPPLGVRVLSAGVFAA     78
                     |  ||   |: |  |    |  |||| |: |   :    |  :  |:|
B_Sequence        51 AATHPGLSVWAGATLGHGLMVGLAVVGGRFLAAHISERAVHWVGGGLFLL    100
                                  5                                  6

A_Sequence        79 FGLRMLWQAYQMTPQQEK     96
                     | |   |:
B_Sequence       101 FALVTSWELLG-------    111


#---------------------------------------

============ FINISHED =============
Average Quality (AQ)      19.10 +/- 6.83
Standard score (Z):       13.0
Precise score (Z):        13.5
```

Fig. S12B

```
# 1: A_Sequence: Tth1 TMS #1-3 (I7M883 ; 2.A.106 homologue)
# 2: B_Sequence: Tth1 TMS #4-6
# Matrix: EBLOSUM62
# Gap_penalty: 8.0
# Extend_penalty: 2.0
#
# Length: 172
# Identity:      34/172 (19.8%)
# Similarity:    58/172 (33.7%)
# Gaps:          48/172 (27.9%)
# Score: 102.0
#
#
#=======================================

A_Sequence         1 MKVLYILIISFLLLSSINTKEPNNEKGNSSEKSLLNSFNDDQILQSHGSF     50
                              : |   | : |: ||:  || |          : : : |
B_Sequence         1 ---------NDLKEKSTSDKQQNNQ-ANSQENEKKKKKKQIKGIAAPGYV     40

                           1   ExGD(K/R)(T/S)                      2
A_Sequence        51 IG--SFISTSVSEIGDKTFIMTAILSSKYNRFWVFVGSVGSMLIMTLISC     98
                     |   :|:|    | |||: | |  :|: |:  :||:|:|   :   |::
B_Sequence        41 IAMQTFVSNFFGEWGDKSQISTIAISASYDFVFVFLGTVVGQIFCILLAL     90
                           4   ExGD(K/R)(T/S)                      5
                                        3
A_Sequence        99 LLGS-LTEYFIPLVYVKFISSALFLIFGLKMLYEVYTDTVDDEDDEAEEE    147
                     : |  | : |     :  :   ||:||    ||
B_Sequence        91 IGGQVLAKQFSEKT-MALLGGILFIIFSFITLYTTLNK------------    127
                                        6

A_Sequence       148 VEELEKRLSKIVTKPKTETDQN    169

B_Sequence       127 ----------------------    127


#---------------------------------------

============ FINISHED =============
Average Quality (AQ)      18.33 +/- 7.38
Standard score (Z): 11.0
Precise score (Z):  11.3
```

Fig. S12C

```
# 1: A_Sequence: Tcr1 TMS #1-3 (K4DX00 ; 2.A.106 homologue)
# 2: B_Sequence: Tcr1 TMS #4-6
# Matrix: EBLOSUM62
# Gap_penalty: 8.0
# Extend_penalty: 2.0
#
# Length: 137
# Identity:      25/137 (18.2%)
# Similarity:    47/137 (34.3%)
# Gaps:          26/137 (19.0%)
# Score: 52.0
#
#
#=======================================
                                               1  ExGD(K/R)(T/S)
A_Sequence         1 -------MAIHATRRW--TEGLLSS-FSMILVSEIGDKTFFIACLMAMRH     40
                             |  | | |     :::  |::  |:| ||::      :|
B_Sequence         1 TGSISSTGAGCARRHWFAFHPVMAEVFALTFVAEWGDRSQLATIALAAAK     50
                                               4  ExGD(K/R)(T/S)
                                 2                          3
A_Sequence        41 SKVLVFLGAIGALAGMTVLSALMGLVVPSVLSVRVTKMLAVVLFFGFGGK     90
                     :   | :| :   |  | :: | | :    :|:|   ::   ||  |
B_Sequence        51 NPFAVTIGGVLGHAVCTGVAVLCGNMTARYVSMRSVNIVGGGLFIVFALA    100
                                 5                          6
A_Sequence        91 ILYDEFAKRGQGDAESDDEMTEAAAIIRKKDPNDAVE    127
                      ||:        | |   :  :
B_Sequence       101 TLYELITNTHHID-EMQQQKEK---------------    121


#---------------------------------------

============ FINISHED =============
Average Quality (AQ)       14.58 +/- 6.61
Standard score (Z): 6.0
Precise score (Z):  5.7
```

Fig. S13A

```
# 1: A_Sequence: Nps1 TMS #1-3 (Q8YX33 ; 2.A.108 homologue)
# 2: B_Sequence: Nps1 TMS #4-6,7
# Matrix: EBLOSUM62
# Gap_penalty: 8.0
# Extend_penalty: 2.0
#
# Length: 150
# Identity:      30/150 (20.0%)
# Similarity:    50/150 (33.3%)
# Gaps:          54/150 (36.0%)
# Score: 99.0
#
#
#=======================================
                                             1 (D/E)xxE
A_Sequence         1 -----------MNWEIVLASFASSLIELVEILGIVIVVGRL-AGWRNALV     38
                                 ||  |: :|  :|:: ||:   |: :|   : |  |:
B_Sequence         1 EQLARDENESGWNWFAVVTTFKGALLDSVEVAIAVVTLGAAQSQWLEAIS     50
                                             4 (D/E)xxE
                           2                            3
A_Sequence        39 GSGAGIALTLLLSLVLGKSLTLIPVNILRIVAGVLLLLFGQKWTRSIVRY     88
                     |:|      |:|: :    |  :||  ::  | :||: ||  |
B_Sequence        51 GAGFATFSLLVLAFLFRTPLQQVPVKPMKFTAAMLLMGFGLYWLG-----     95
                           5                            6

A_Sequence        89 YAGLPKKRKGGGEDSLE---------------------------------    105
                      |||  :  |   | |
B_Sequence        96 -AGLNVEWPG---DELAIIWLPLAWGVGMAIASTIWRWRVSLDKPEEAIG    141
                                               7

#---------------------------------------

============ FINISHED =============
Average Quality (AQ)      20.11 +/- 7.38
Standard score (Z): 11.0
Precise score (Z):  10.7
```

Fig. S13B

```
# 1: A_Sequence: Nps3 TMS #1-3 (K9Q6B8 ; 2.A.108 homologue)
# 2: B_Sequence: Nps3 TMS #4-6,7
# Matrix: EBLOSUM62
# Gap_penalty: 8.0
# Extend_penalty: 2.0
#
# Length: 151
# Identity:      30/151 (19.9%)
# Similarity:    47/151 (31.1%)
# Gaps:          58/151 (38.4%)
# Score: 96.0
#
#
#=======================================
                                               1(D/E)xxE
A_Sequence         1 -------------MNWEIFLASFVGSLIELVEILGLVLIVGKLAG-WRNA     36
                                   ||     :| |:|:: ||:   |: :|   | |  |
B_Sequence         1 LETELANTGNQLGWNWFAIATTFKGALLDSVEVAIAVVTLGATGGKWLEA     50
                                               4 (D/E)xxE
                           2                                3
A_Sequence        37 FVGA-GSGIGLTLLASLILGTSLTIIPVDILRIVAGVFLLAFGQKWTRSI     85
                       ||  :  || ::|  :  | |  :|:  ::  | : |: ||   |
B_Sequence        51 AGGASAAAFGLVVVA-FLFRTPLNQVPIKPMKFTAAMLLMGFGIYWLSE-     98
                           5                                6

A_Sequence        86 VKYYAGIPKKRKDEEDD---------------------------------    102
                          |   | |   ||
B_Sequence        99 -----GF--KIKLPGDDWAIVWLPIVWGCLMAVSALLLRWQVGLQPKEIV    141
                                               7

A_Sequence       102 -    102

B_Sequence       142 S    142


#---------------------------------------

============ FINISHED =============
Average Quality (AQ)      21.97 +/- 7.85
Standard score (Z): 9.0
Precise score (Z):  9.4
```

Fig. S13C

```
# 1: A_Sequence: Rsp3 TMS #1-3 (J2KV33 ; 2.A.108 homologue)
# 2: B_Sequence: Rsp3 TMS #4-6,7
# Matrix: EBLOSUM62
# Gap_penalty: 8.0
# Extend_penalty: 2.0
#
# Length: 140
# Identity:      37/140 (26.4%)
# Similarity:    56/140 (40.0%)
# Gaps:          30/140 (21.4%)
# Score: 84.0
#
#
#=======================================
                                        1 (D/E)xxE
A_Sequence         1 MTTITSITSTMA--ASFLGSFVEVVEAFTIILAVGVTQSWRPAFIGTGLA     48
                       :       :|  |:|    :| ||   |::| |      | : | |
B_Sequence         1 --SADRRADFLAGTAAFKAVLLEGVEVVFIVIATGARPGMLP-YAGLGAL     47
                                        4 (D/E)xxE
                           2                          3
A_Sequence        49 LSVLAVLV---LIFGPLLGLIPIDILQFTIGTLLILFGMRWLRKAI----     91
                     :: :||||   |:  | |  :| : |:| :| ||  ||: |: : |
B_Sequence        48 IACIAVLVIGLLVHKP-LSSVPENTLKFIVGLLLTAFGIFWIGEGIGTPW     96
                           5                          6

A_Sequence        92 ----LRASGFIALHDEEKAFASETDALARQ----------    117
                         |   |  ||     ||:       ||
B_Sequence        97 PGEDLSLIGIFAL---LAAFSFIAVRWLRQYHHAQTEPAR    133
                                 7

#---------------------------------------

============ FINISHED =============
Average Quality (AQ)       22.15 +/- 7.74
Standard score (Z): 8.0
Precise score (Z):  8.0
```

Fig. S14A

```
# 1: A_Sequence: Ceu1 TMS #1-3 (A8SU47 ; 2.A.107 homologue)
# 2: B_Sequence: Ceu1 TMS #4-6
# Matrix: EBLOSUM62
# Gap_penalty: 8.0
# Extend_penalty: 2.0
#
# Length: 98
# Identity:      22/98 (22.4%)
# Similarity:    42/98 (42.9%)
# Gaps:           8/98 ( 8.2%)
# Score: 82.0
#
#
#=======================================
                                 1 Fully conserved D
A_Sequence         1 ---MSIVELFMLAVGLSMDAFAVSICKGLSLRDIKVKHMVIAGVWFGGFQ     47
                        ||   :|:|||  |:|| || :    :   :   ::|:| :: |
B_Sequence         1 NADMSAKVMFLLAVATSIDALAVGV--SFAFLKLSTLYIVLAVIFIGCIT     48
                                 4 Fully conserved D
                           2                          3
A_Sequence        48 ALMPTLGYVLGSFFADLVSKWSHWIAFVLLLFIGGSMIKESFGGEEEV     95
                      :    |  :|| |       :     ::|: ||  :: :  |
B_Sequence        49 FIFSAAGVKIGSIFGTKYKSKAELAGGIILILIGIKVVLDGLGIL---     93
                           5                          6

#---------------------------------------
```

============ FINISHED =============
Average Quality (AQ)   24.98 +/- 7.01
Standard score (Z): 8.0
Precise score (Z): **8.1**

Fig. S14B

```
# 1: A_Sequence: Rsp2 TMS #1-3 (R9SLI6 ; 2.A.107 homologue)
# 2: B_Sequence: Rsp2 TMS #4-6
# Matrix: EBLOSUM62
# Gap_penalty: 8.0
# Extend_penalty: 2.0
#
# Length: 102
# Identity:      22/102 (21.6%)
# Similarity:    40/102 (39.2%)
# Gaps:          20/102 (19.6%)
# Score: 64.0
#
#
#=======================================
                                        1 Fully conserved D
A_Sequence         1 ----------MNIFELFILAIGLSMDAFAVSVCKGLSLGRINAKHMCIAG     40
                               |:|  :||||:  |:|| ||    |::   :  : :  |
B_Sequence         1 SKEEEHVNADMDIKSMFILAVATSIDALAV----GVTFAFLKVE-IVSAV     45
                                        4 Fully conserved D
                           2                                 3
A_Sequence        41 AWFGGFQALMPLVGYFGGRFFADKVTRYSHWVAFVLLVFIGAGMIKE---     87
                     :: |    :    |   |  |  |    :     ::|: ||  :: |
B_Sequence        46 SFIGVITFVCSAAGVKIGSLFGMKYKSKAELCGGIILILIGTKILLEGLG     95
                           5                                 6

A_Sequence        87 --     87

B_Sequence        96 MI     97


#---------------------------------------

============ FINISHED =============
Average Quality (AQ)      19.03 +/- 6.10
Standard score (Z): 7.0
Precise score (Z):  7.4
```

Fig. S14C

```
# 1: A_Sequence: Msp1 TMS #1-3 (C6JCY1 ; 2.A.107 homologue)
# 2: B_Sequence: Msp1 TMS #4-6
# Matrix: EBLOSUM62
# Gap_penalty: 8.0
# Extend_penalty: 2.0
#
# Length: 103
# Identity:      22/103 (21.4%)
# Similarity:    43/103 (41.7%)
# Gaps:          20/103 (19.4%)
# Score: 67.0
#
#
#=======================================
                                        1 Fully conserved D
A_Sequence         1 -----------MDIVSTLLIAVALAMDAFSVSLTKGFTLKNITLKQILWF     39
                                      :|:||| ::|||:| :|    ||   |  ::
B_Sequence         1 FSDDLDDDEDTFSFAELILLAVATSIDAFAVGVTYA-VLKIDILIPVIII     49
                                        4 Fully conserved D
                           2                                3
A_Sequence        40 GVFFGGFQSLMPILGWTLGVQLQLIVSEVAPWIAFILLVLIGANMIRES-     88
                     |:    |  :  |:|  || ::     :    :  ::|:|:|  :: |
B_Sequence        50 GLV--AF--IFTIIGIYLGKKIGDYFGDKFEILGGVILILLGCRILLEGL     95
                           5                                6

A_Sequence        88 ---     88

B_Sequence        96 GFL     98


#---------------------------------------

============ FINISHED =============
Average Quality (AQ)      21.80 +/- 6.60
Standard score (Z): 7.0
Precise score (Z):  6.9
```

Fig. S15A

```
# 1: A_Sequence: Gth1 TMS #1-3 (A4IKQ1 ; 2.A.109 homologue)
# 2: B_Sequence: Gth1 TMS #4-6,7
# Matrix: EBLOSUM62
# Gap_penalty: 8.0
# Extend_penalty: 2.0
#
# Length: 141
# Identity:      29/141 (20.6%)
# Similarity:    52/141 (36.9%)
# Gaps:          50/141 (35.5%)
# Score: 98.0
#
#
#=======================================
                                       1 DxxxxxDN
A_Sequence         1 MSVDLFSPEFWTALLSIVIIDLVLAGDNAIVIGLAARNLPKHQQKKAVIW     50
                               | |: :|:| | ::  || : :  ||     |     ||
B_Sequence         1 -------GSLWEAVRTIIIADALMGLDNVLAVAGAA-----HGHFLLVIL     38
                                       4 DxxxxxDN
                           2                         3
A_Sequence        51 GTVGAVVIRAM-ATIFVVWLLKIPGLLLVGGLLLVWIAYKLLVEE---KG     96
                     | : :| |    :|: : |: : | :: :|  :| | | |::|:|   ||
B_Sequence        39 GLLISVPIMVWGSTLILKWIERFPIIITIGAGILAWTASKMIVDEPFLKG     88
                           5                         6

A_Sequence        97 H---DDIEAG-------------------------------    103
                     :     |: |
B_Sequence        89 YFANPVIKYGFELLLVAAVIAIGTQKKRKAAKKPHLKVANE    129
                                 7

#---------------------------------------

============ FINISHED =============
Average Quality (AQ)       23.59 +/- 7.93
Standard score (Z): 9.0
Precise score (Z):  9.4
```

Fig. S15B

```
# 1: A_Sequence: Bsp2 TMS #1-3 (G8M4S7 ; 2.A.109 homologue)
# 2: B_Sequence: Bsp2 TMS #4-6,7
# Matrix: EBLOSUM62
# Gap_penalty: 8.0
# Extend_penalty: 2.0
#
# Length: 143
# Identity:      30/143 (21.0%)
# Similarity:    44/143 (30.8%)
# Gaps:          50/143 (35.0%)
# Score: 92.0
#
#
#=======================================
                                      1 DxxxxxDN
A_Sequence         1 MLEFFSTLHWGAVVQIIVIDILLGGDNAVVIALACRNLPDRQRTRGIVLG     50
                          |   | ||  |:: | ::  || : || |      | |   :: |
B_Sequence         1 -----SDRLWAAVKTIVIADAVMSLDNVIAIAGAAEAADPRHRLALVIFG     45
                                      4 DxxxxxDN
                           2                         3
A_Sequence        51 TLGAILLRVILIAFAVMLLD-VPFLKFVGGVLLLWIGVKLMQPDHDEHHI     99
                      : :| | |      : |||  | :  :|  || ||   |:    |:  |
B_Sequence        46 LIVSIPLIVWGSTLVLKLLDRFPVVVLLGAALLGWIAGGLI---IDDPFI     92
                           5                         6

A_Sequence       100 DA-----------------------------------------    101
                     |
B_Sequence        93 DRWPALNTDIVGYAARVAGALFVVGVGWLLRRRALADGNRATG    135
                                        7

#---------------------------------------

============ FINISHED =============
Average Quality (AQ)      21.13 +/- 7.79
Standard score (Z): 9.0
Precise score (Z):  9.1
```

Fig. S15C

```
# 1: A_Sequence: Pba1 TMS #1-3 (R9LI44 ; 2.A.109 homologue)
# 2: B_Sequence: Pba1 TMS #4-6,7
# Matrix: EBLOSUM62
# Gap_penalty: 8.0
# Extend_penalty: 2.0
#
# Length: 143
# Identity:      25/143 (17.5%)
# Similarity:    47/143 (32.9%)
# Gaps:          53/143 (37.1%)
# Score: 82.0
#
#
#=======================================
                                     1 DxxxxxDN
A_Sequence         1 MDLLSPEFWMALLSIVLIDLVLAGDNAIVIGLAARNVPQQDQKKVIVWGT      50
                           : | |: :|:: | ::  || : :  ||      |   ||: |
B_Sequence         1 -----NQMWAAIRTIIIADAMMGLDNVLAVAGAAHG----DTLLVII-GL      40
                                     4 DxxxxxDN
                           2                          3
A_Sequence        51 LGAILIRVVMTLLVVQLL-NIPGLRLAGGLALVWIAYKLLIEEK-SHEIK      98
                       :: | |  : ::::|    | :   |   | | | |:::||   |:
B_Sequence        41 AVSVPIMVWGSTMILKLTERFPIVITIGAAVLAWTASKMIVEEPLIHDWF      90
                           5                          6

A_Sequence        99 AG-----------------------------------------    100
                     |
B_Sequence        91 ASPWIKYGFELLVIAAVVLLGNLMKKRKARLHQAKAMPQTNGS    133
                           7

#---------------------------------------

============ FINISHED =============
Average Quality (AQ)       23.22 +/- 7.53
Standard score (Z): 8.0
Precise score (Z):  7.8
```
