# Supplementary material for: The LysE Superfamily of Transport Proteins Involved in Cell Physiology and Pathogenesis
Source: PLoS One. 2015 Oct 16;10(10):e0137184. doi: 10.1371/journal.pone.0137184 (PMC4608589; doi:10.1371/journal.pone.0137184)

Fig. 18A

LysE - Clustal

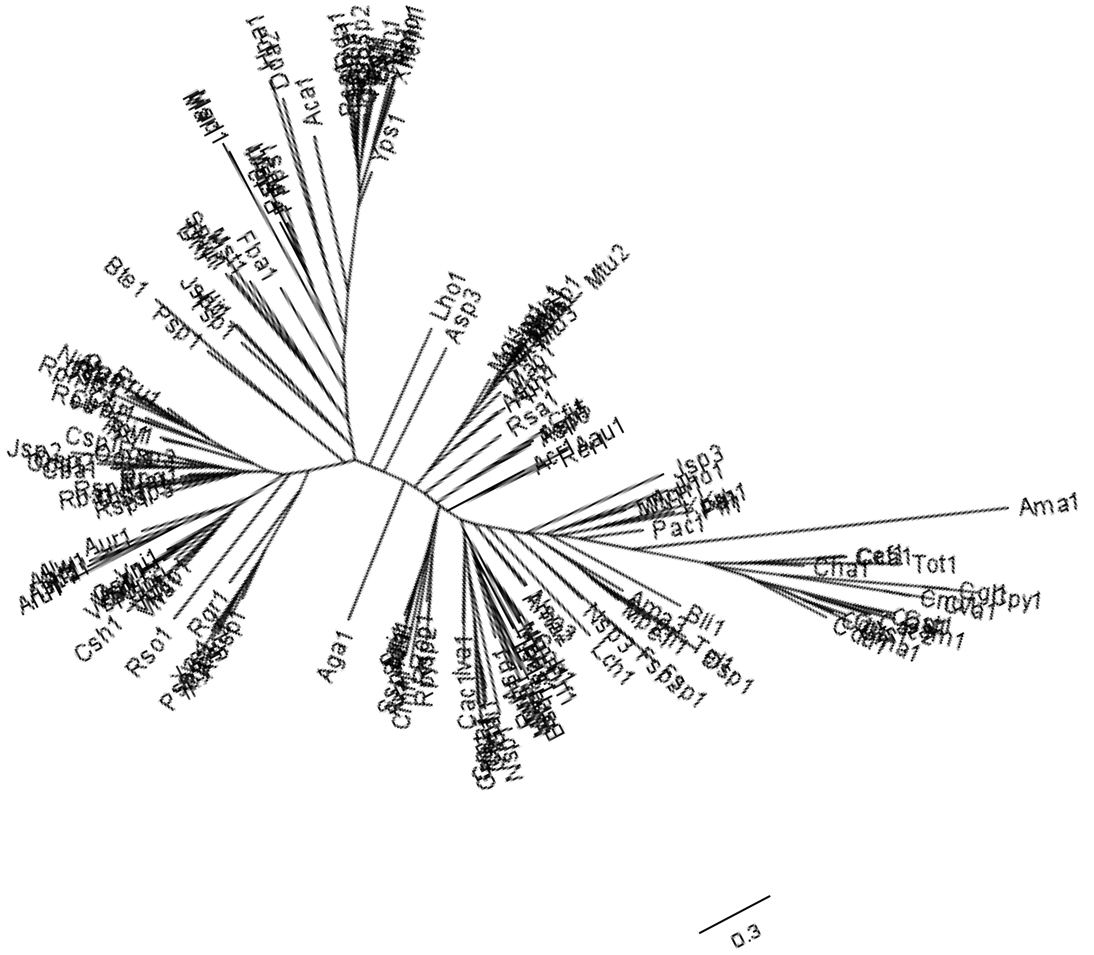

Fig. 18B

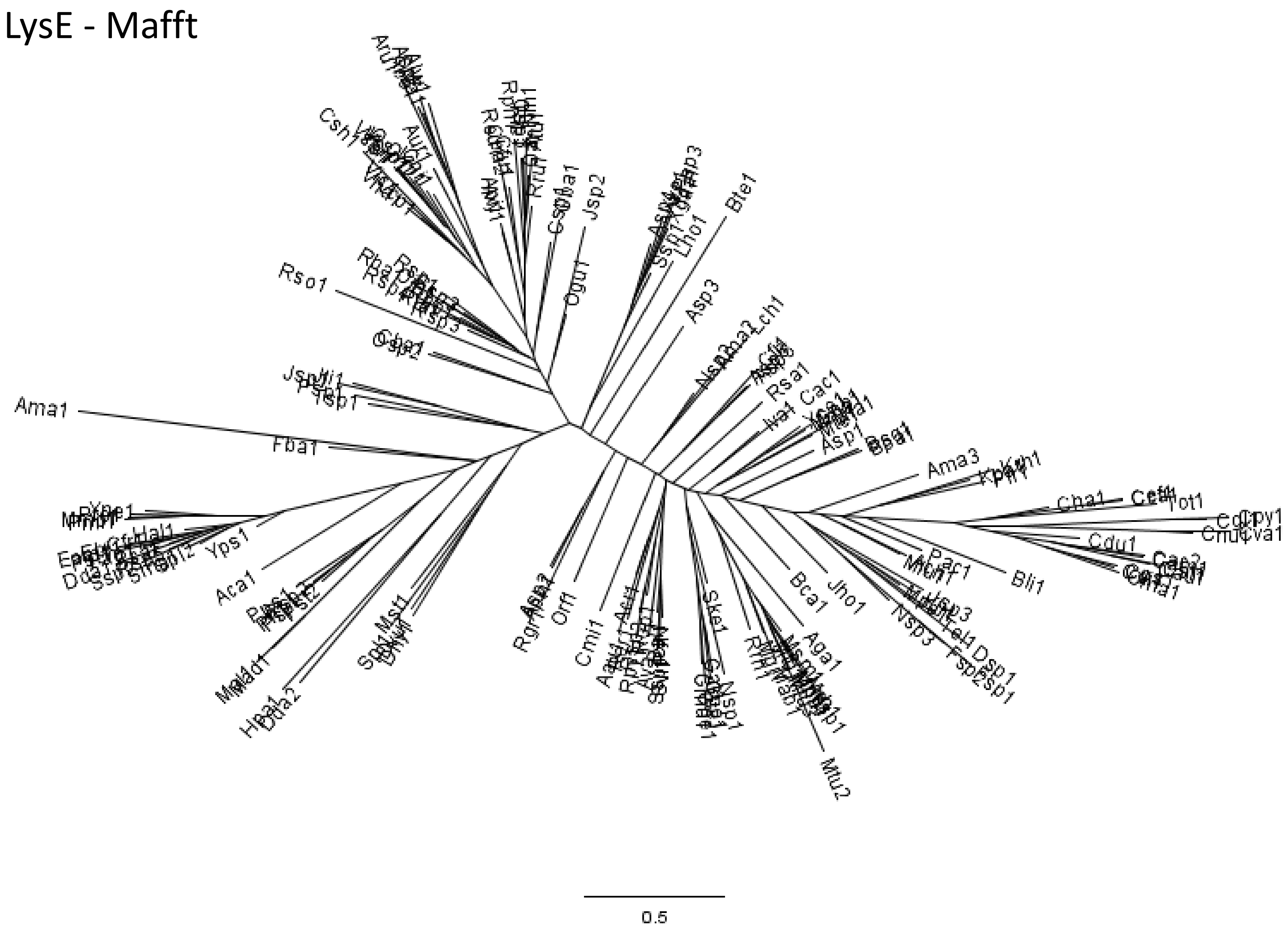

LysE - Mafft
Ama1
Fba1
Rso1
Yps1
Aca1
Jsp2
Ogu1
Bte1
Asp3
Rsa1
Cac1
Ama3
Cha1
Tot1
Cdu1
Pac1
Bli1
Jho1
Bca1
Nsp3
Aga1
Mtu2
Ske1
Cmi1
Orf1
0.5

Fig. 18C

LysE - ProbCons

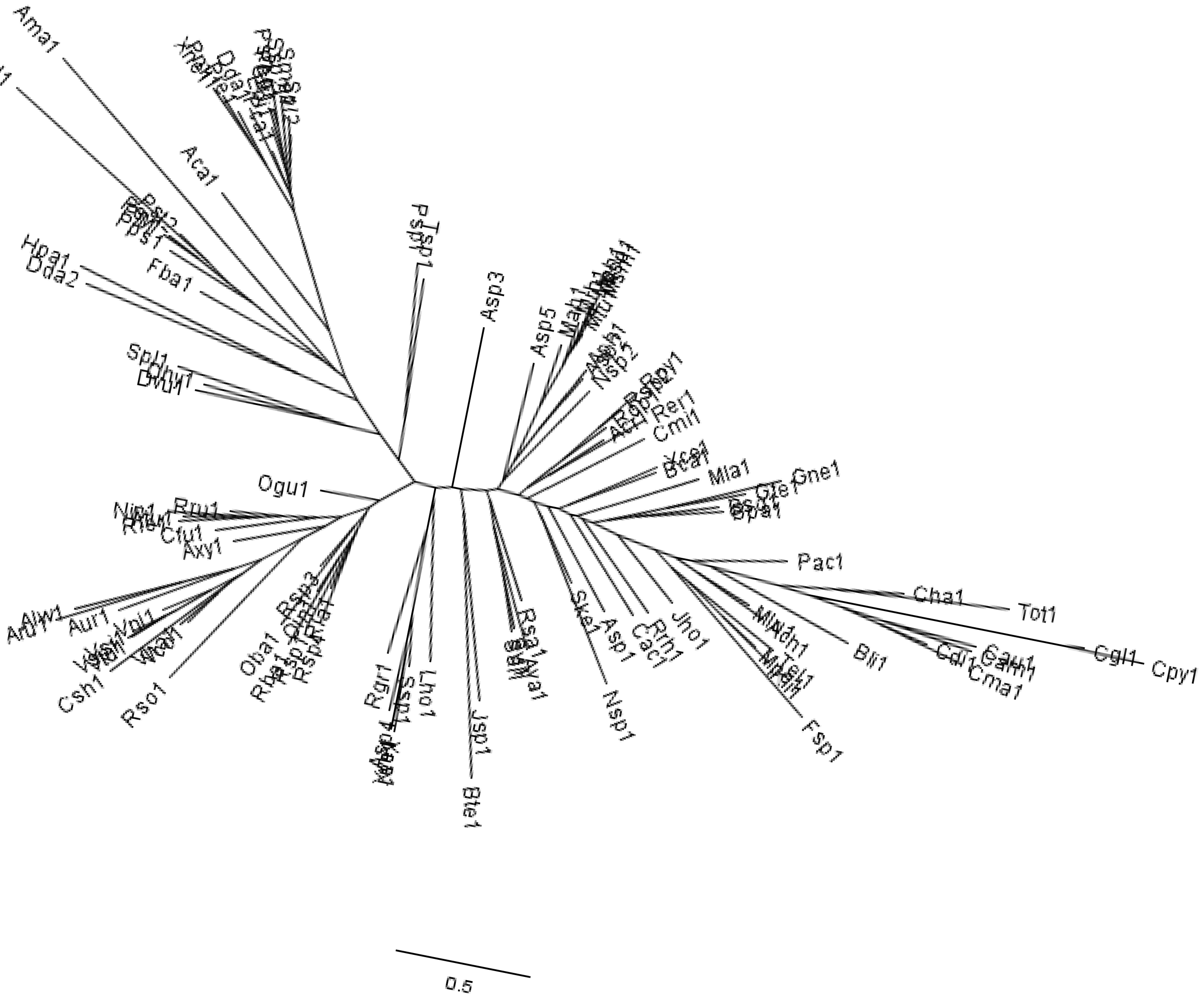

Fig. 19A

RhtB - Clustal

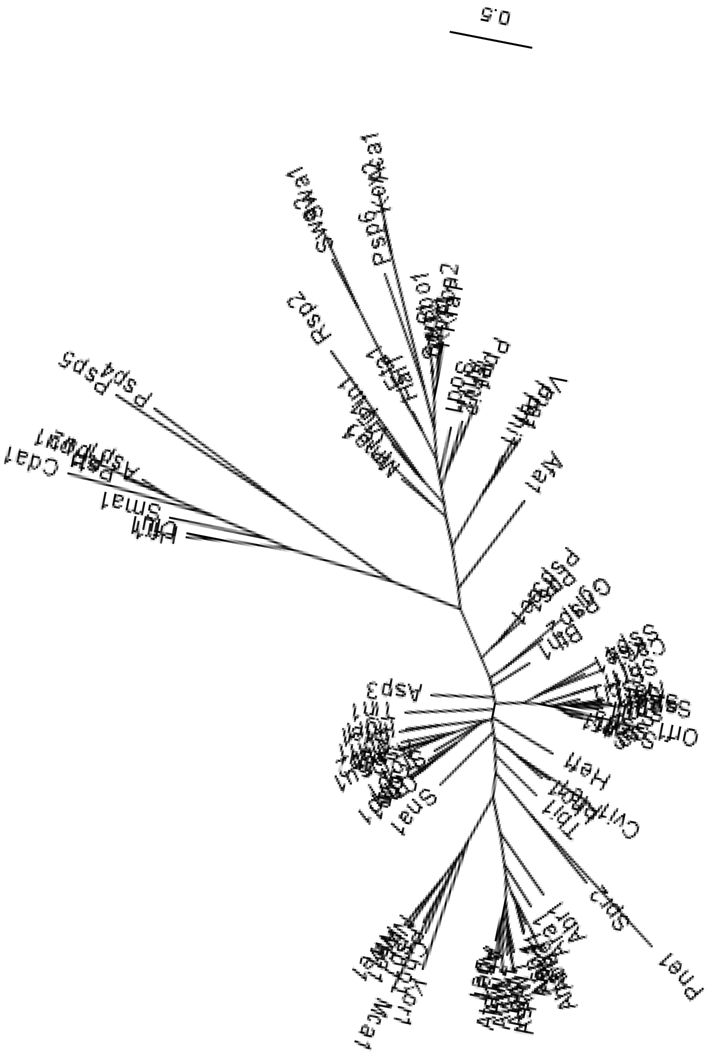

Fig. 19B

RhtB - Mafft

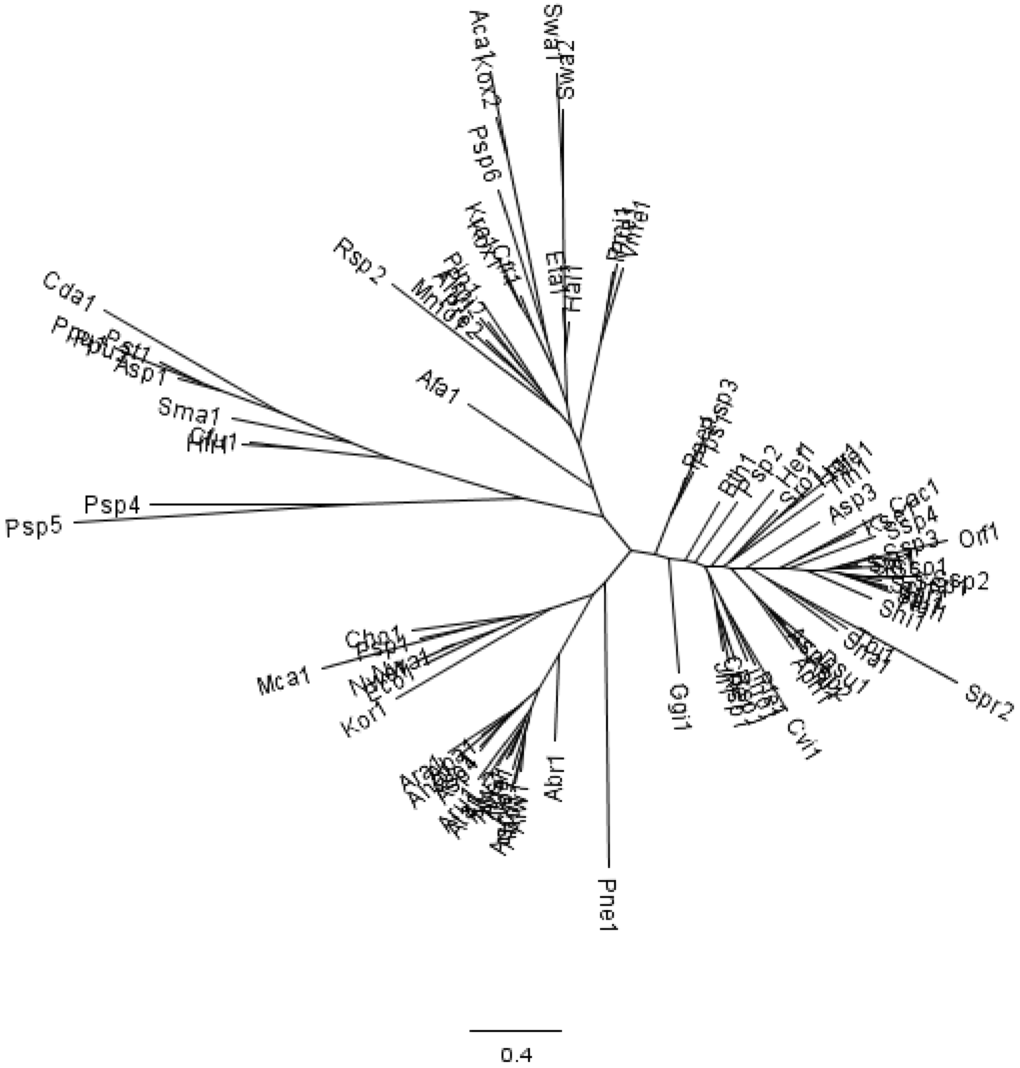

Fig. 19C

RhtB - ProbCons

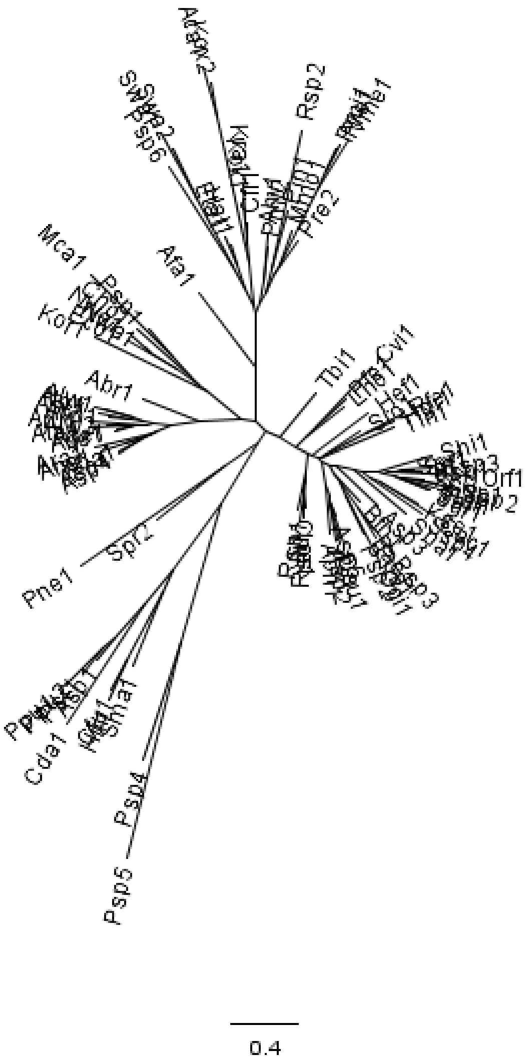

Fig. 20A

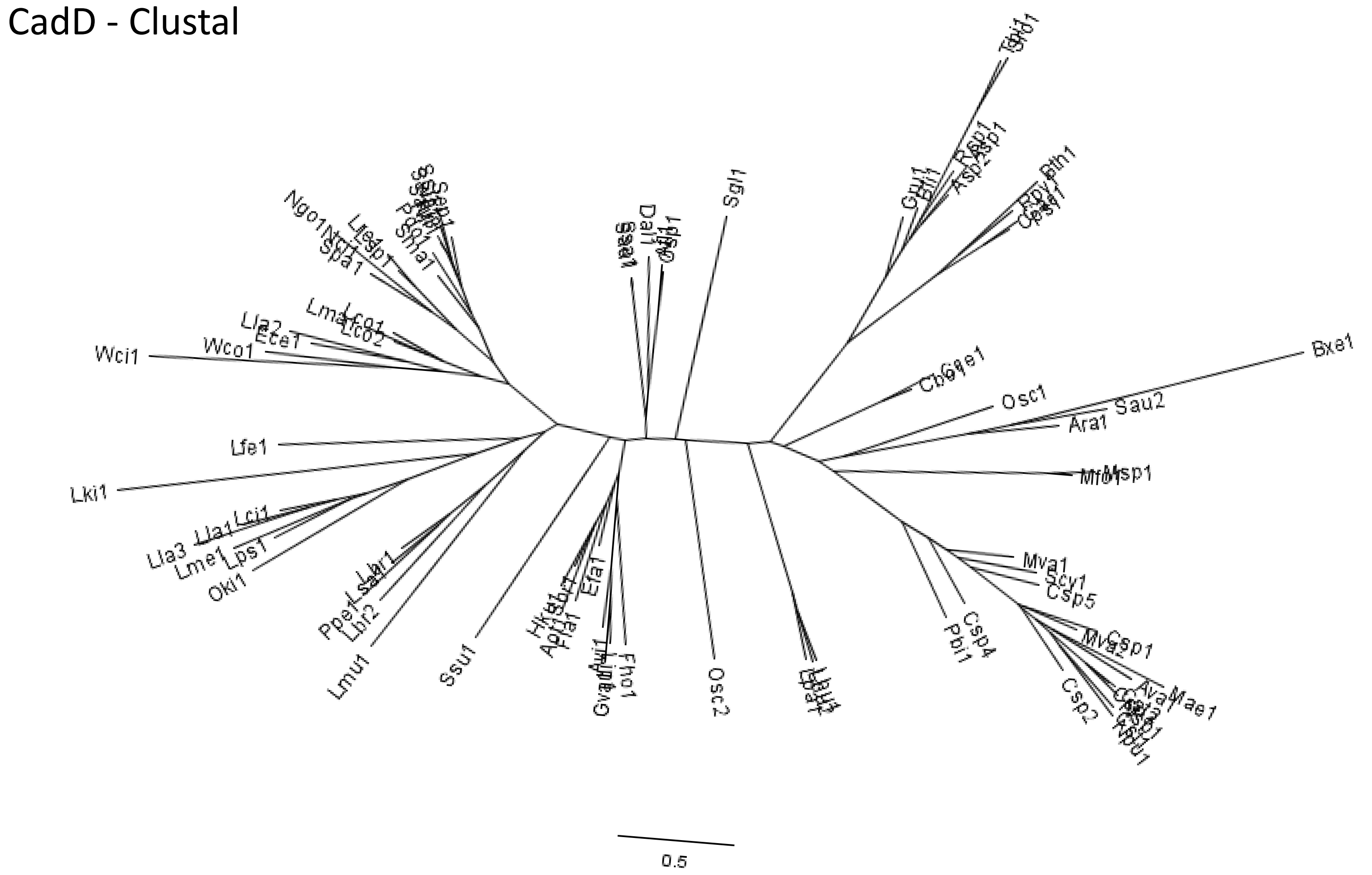

CadD - Clustal
Sgl1
Dal1
Bxe1
Osc1
Sau2
Ara1
Msp1
Mva1
Scy1
Csp5
Csp1
Mva2
Mae1
Csp4
Pbi1
Csp2
Osc2
Fho1
Ssu1
Lmu1
Lbr2
Ppe1
Lbr1
Oki1
Lps1
Lme1
Lla3
Lla1
Lci1
Lki1
Lfe1
Wci1
Wco1
Ece1
Lla2
Lco1
Lco2
Spa1
Gpu1
Rsp1
Asp1
Pth1
Efa1
Fla1
0.5

Fig. 20B

CadD - Mafft

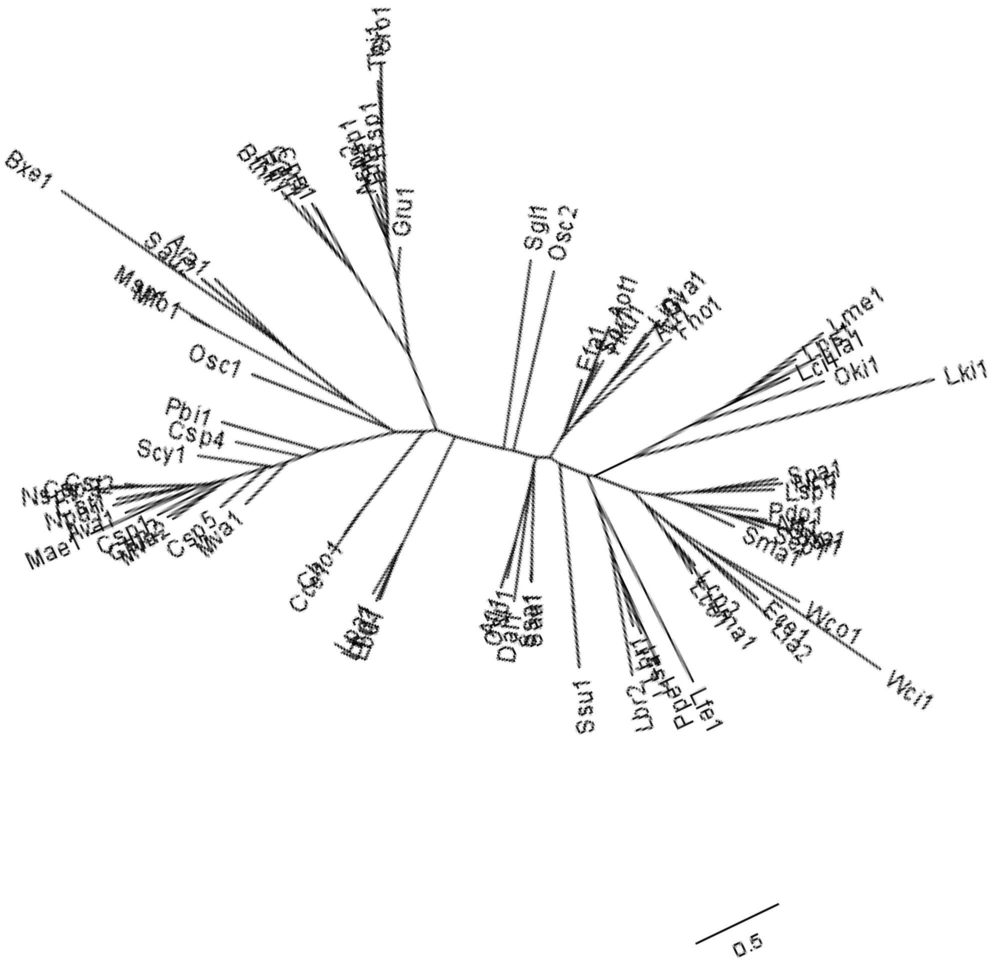

Fig. 20C

CadD - ProbCons

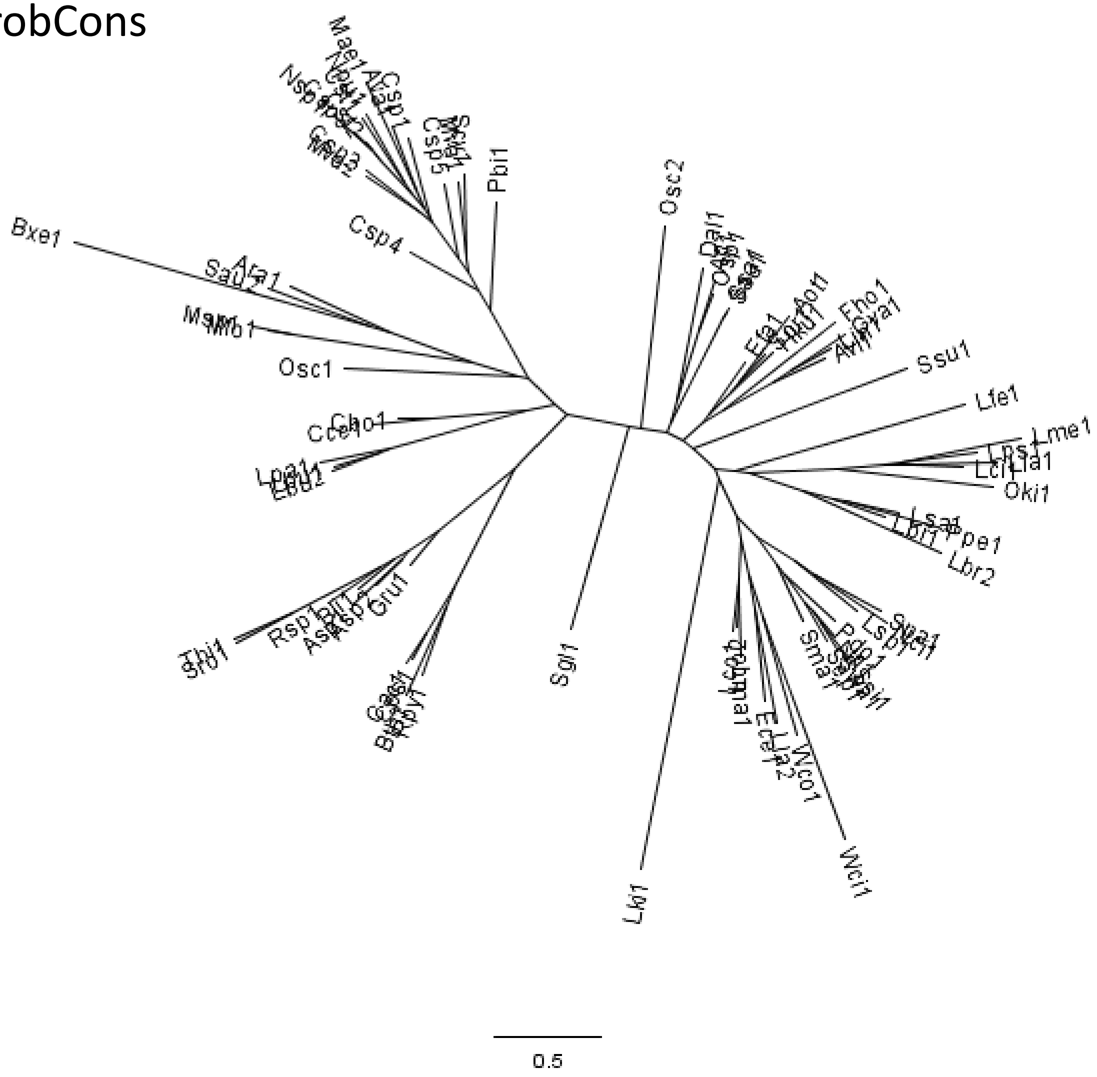

Fig. 21A

CaCA2 - Clustal

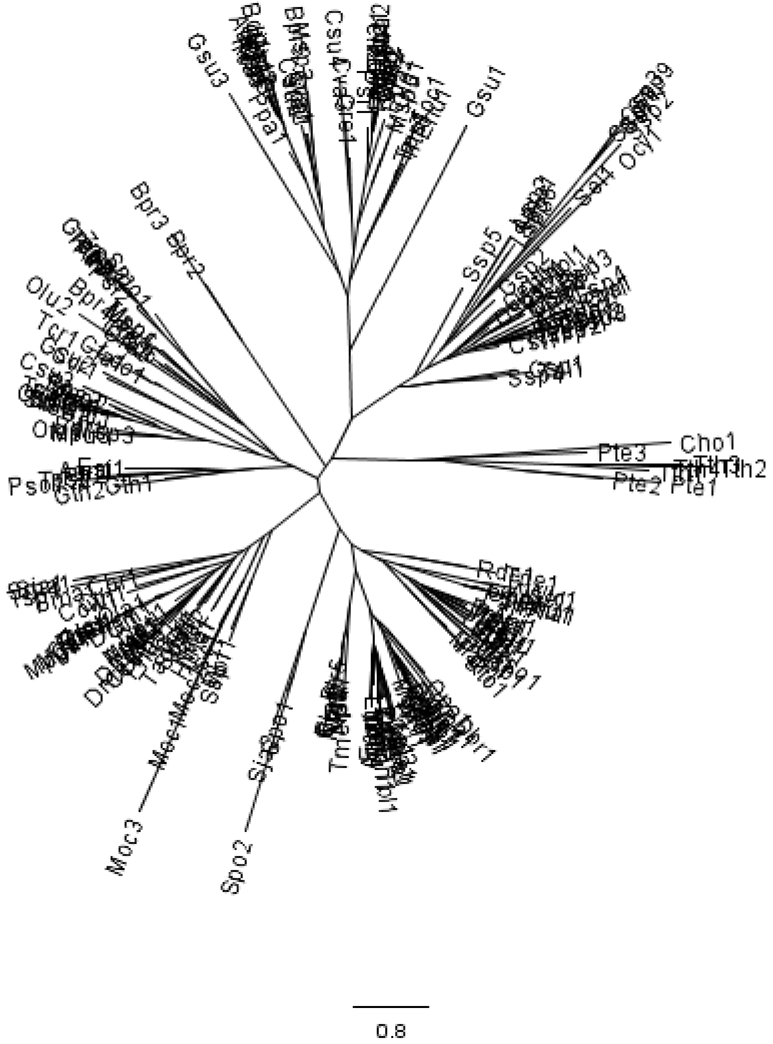

Fig. 21B

CaCA2 - Mafft

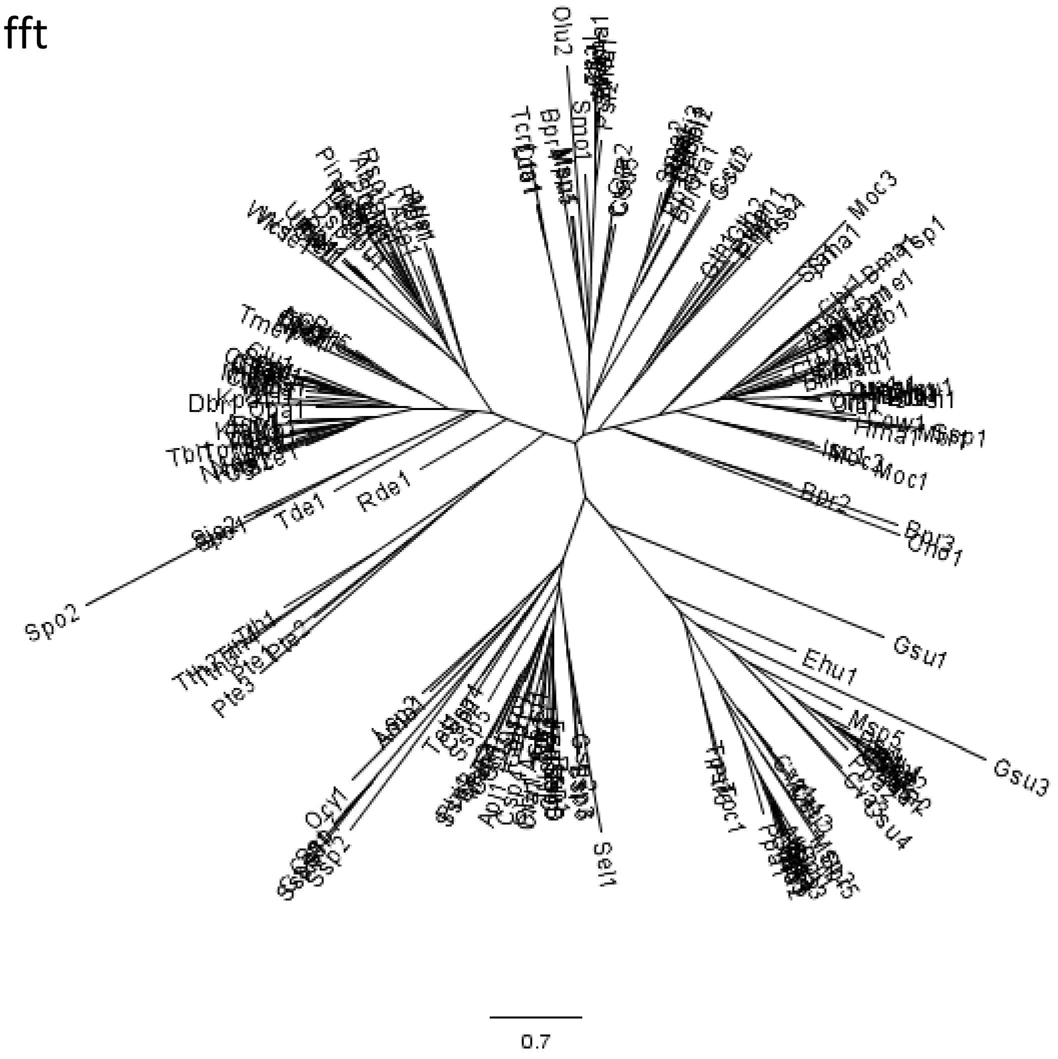

Fig. 21C

CaCA2 - ProbCons

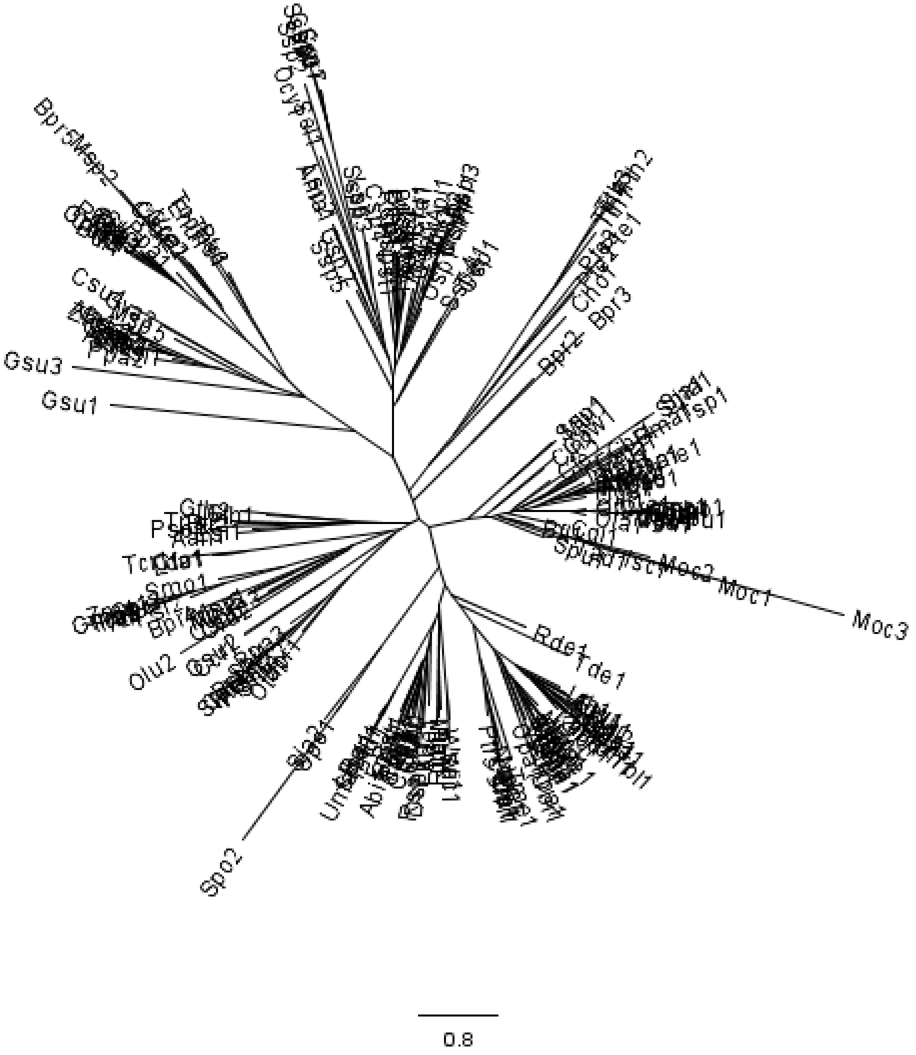

Fig. 22A

MntP - Clustal

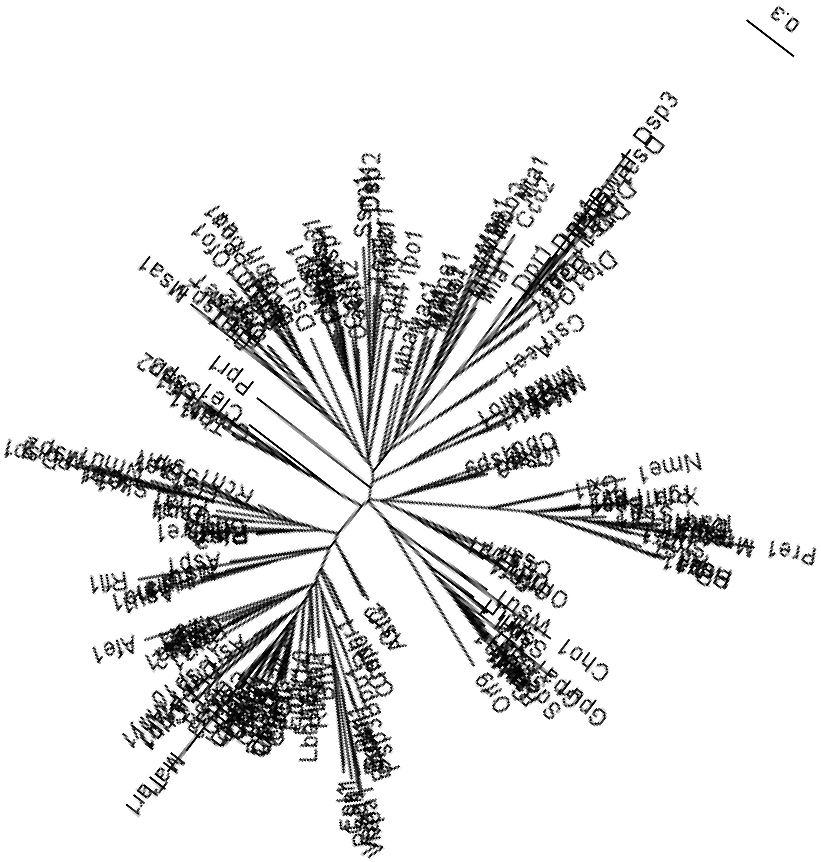

Fig. 22B

MntP - Mafft

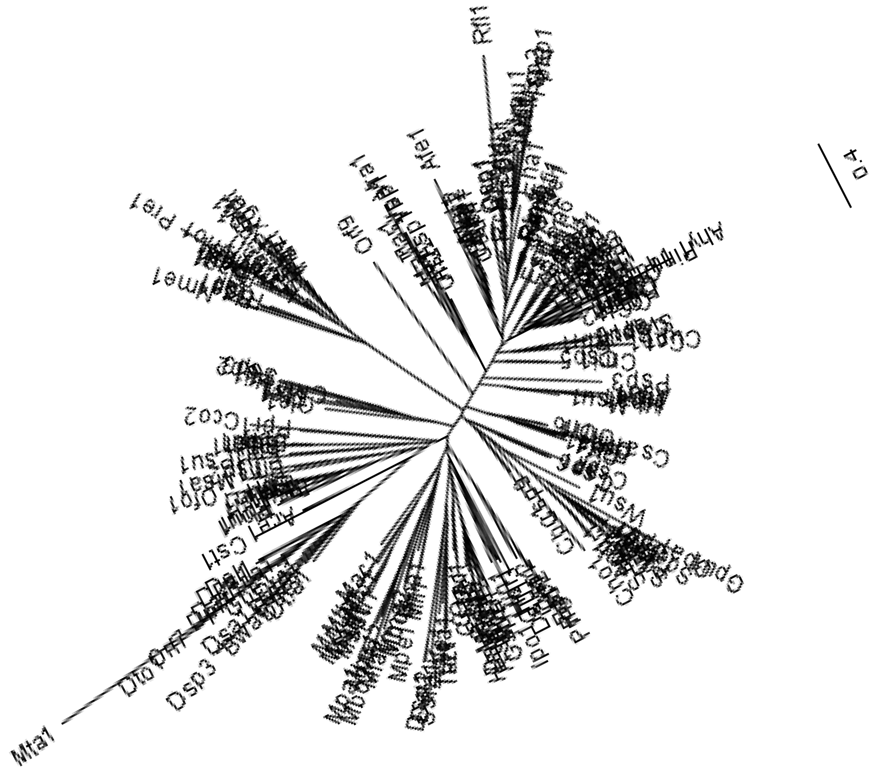

Fig. 22C

MntP - ProbCons

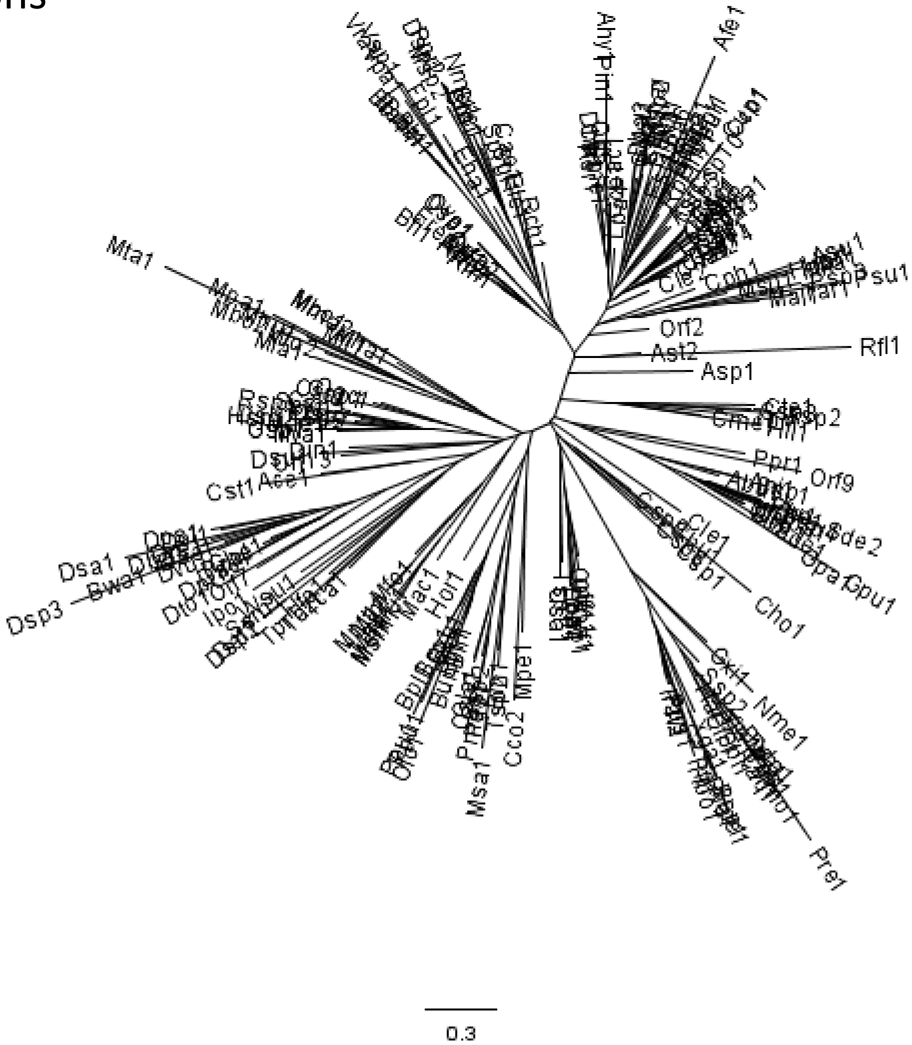

Fig. 23A

ILT - Clustal

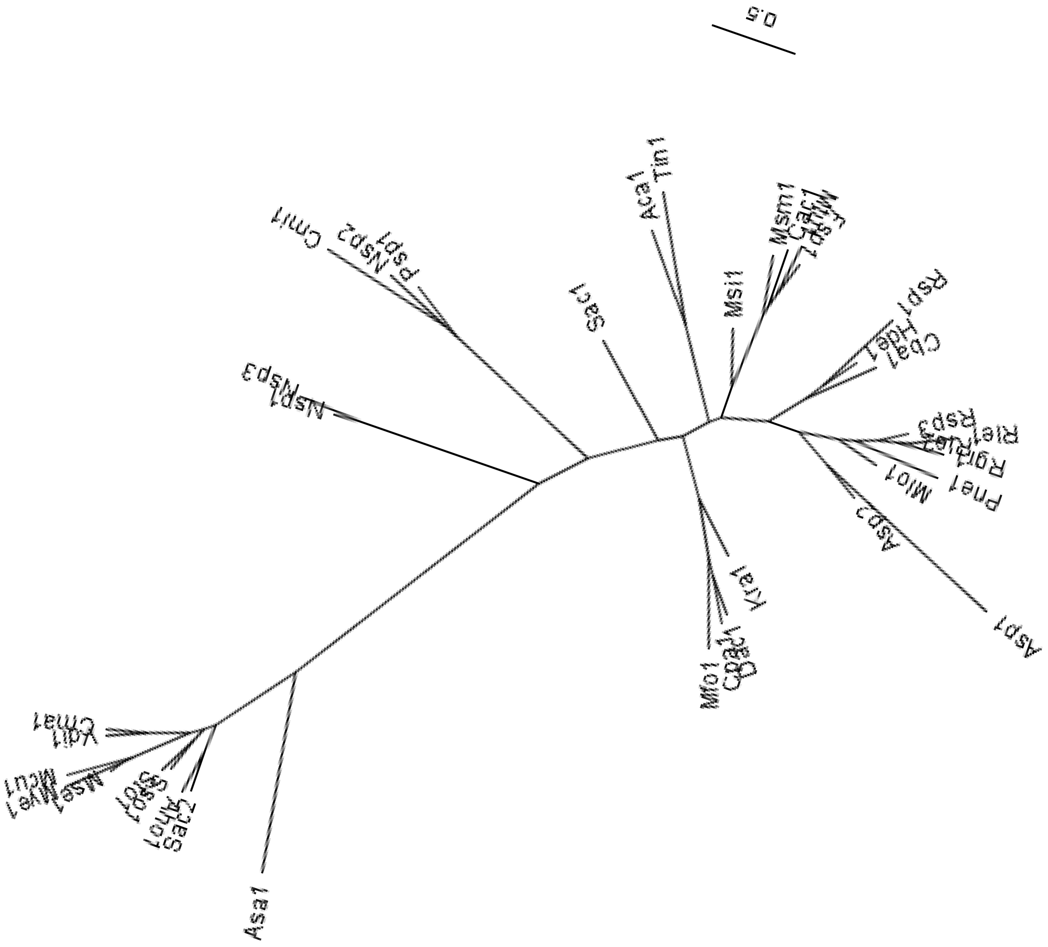

Fig. 23B

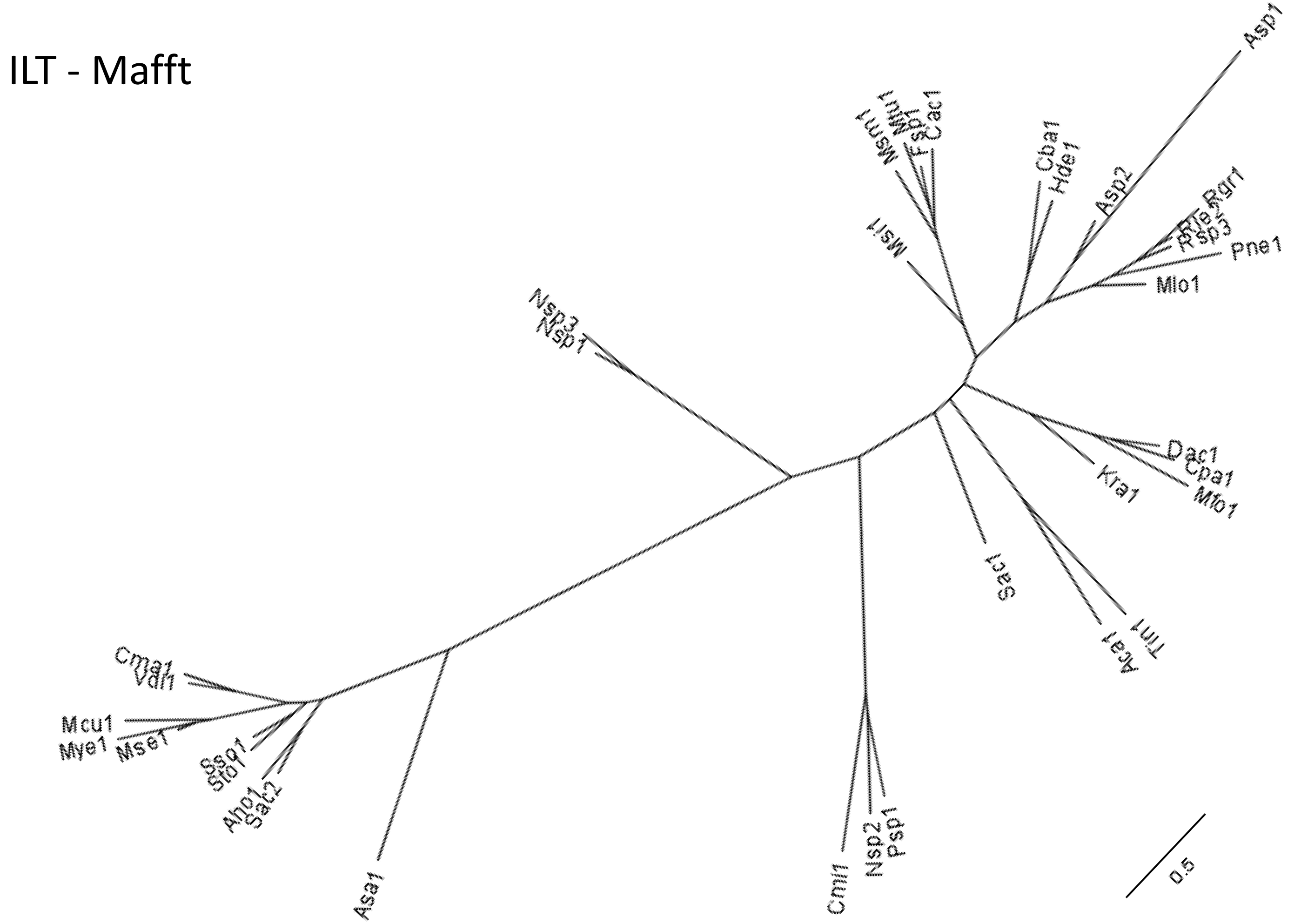
ILT - Mafft
Asp1
Asp2
Cba1
Hde1
Msm1
Mtu1
Fsp1
Cac1
Msi1
Rgr1
Rsp3
Pne1
Mlo1
Nsp3
Nsp1
Dac1
Cpa1
Mfo1
Kra1
Sac1
Aca1
Tin1
Cma1
Vdi1
Mcu1
Mye1
Mse1
Ssp1
Sto1
Aho1
Sac2
Asa1
Cmi1
Nsp2
Psp1
0.5

Fig. 23C

ILT – ProbCons

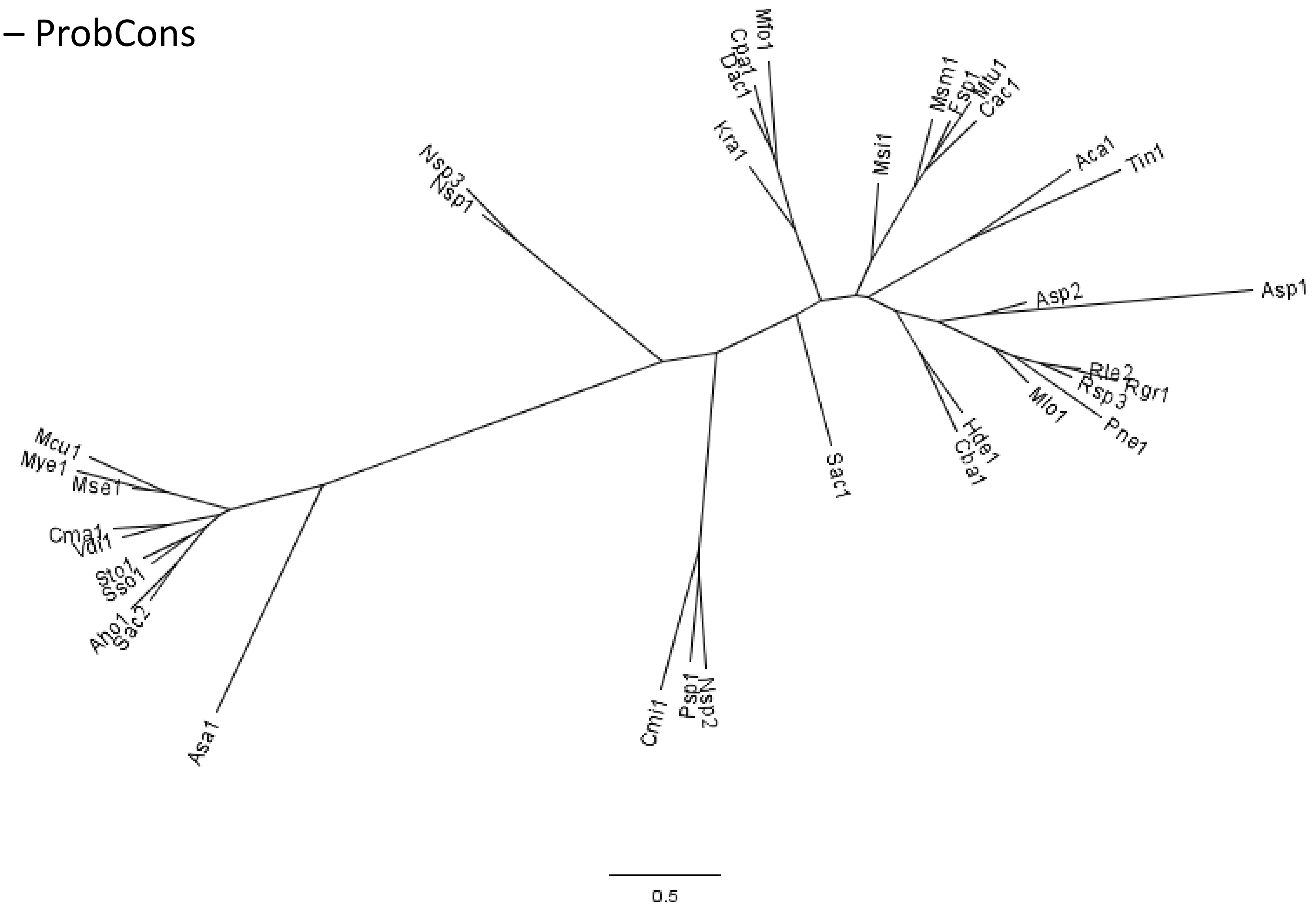

Fig. 24A

TerC – Clustal

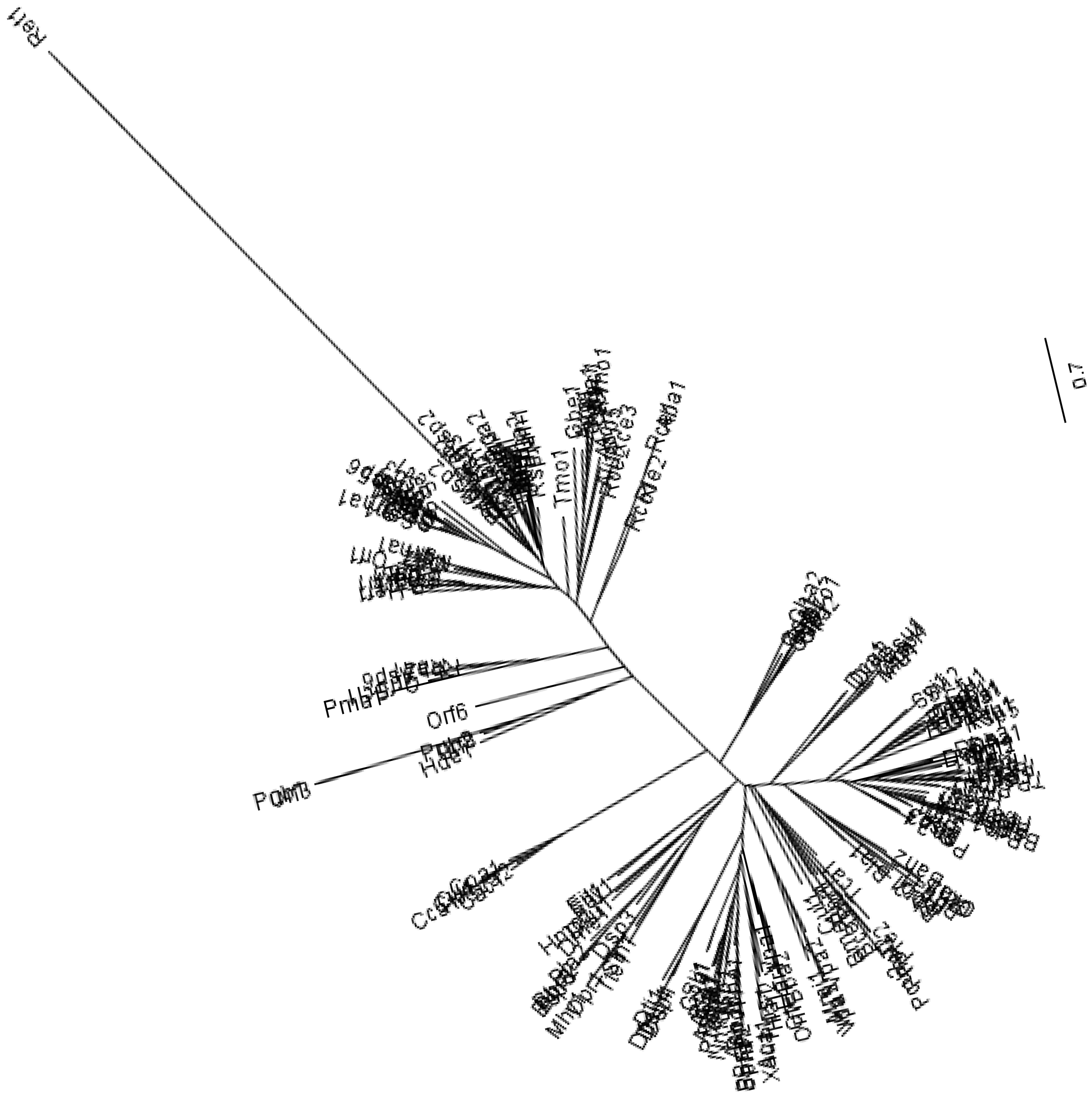

Fig. 24B

TerC - Mafft

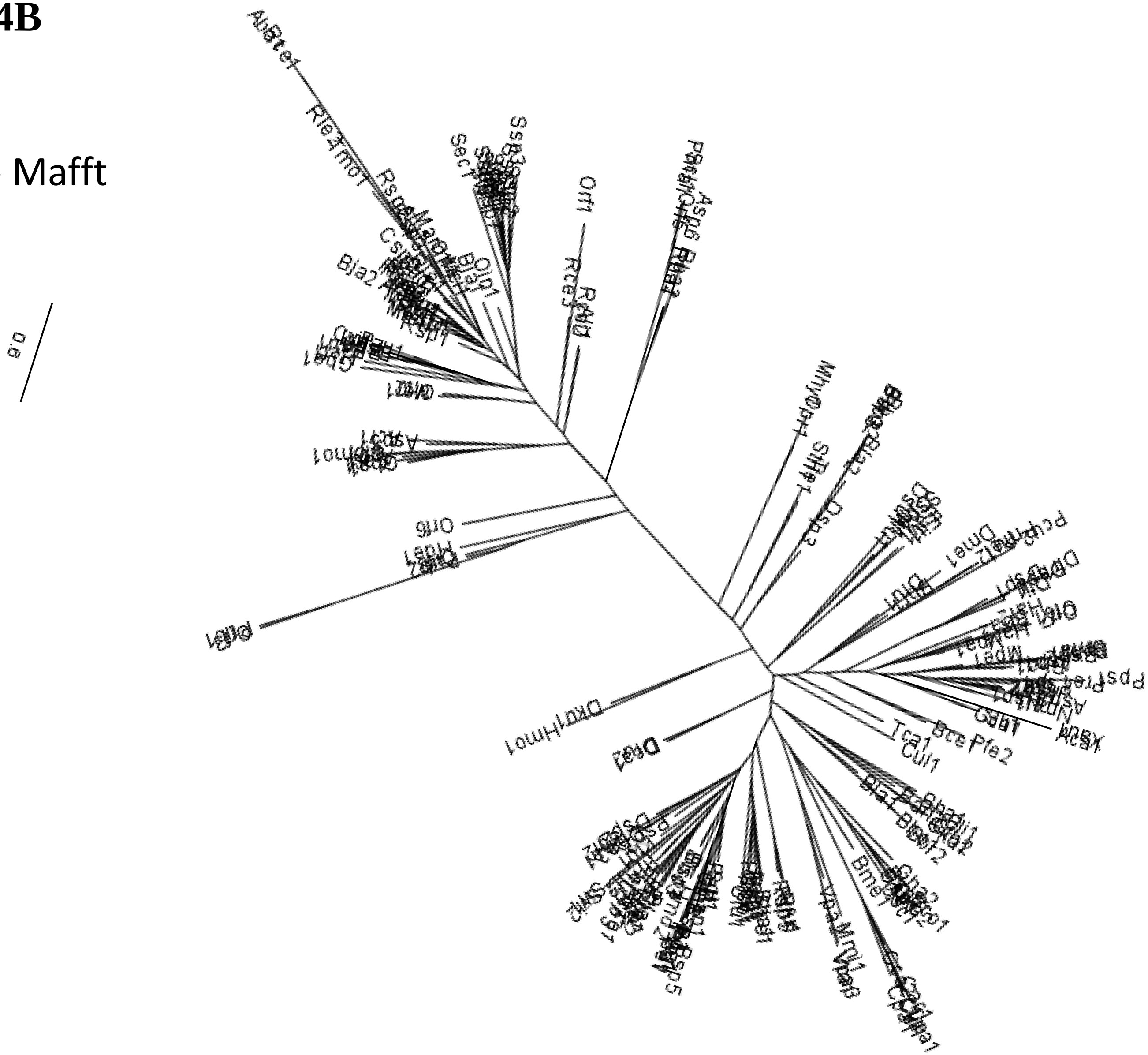

Fig. 24C

TerC - ProbCons

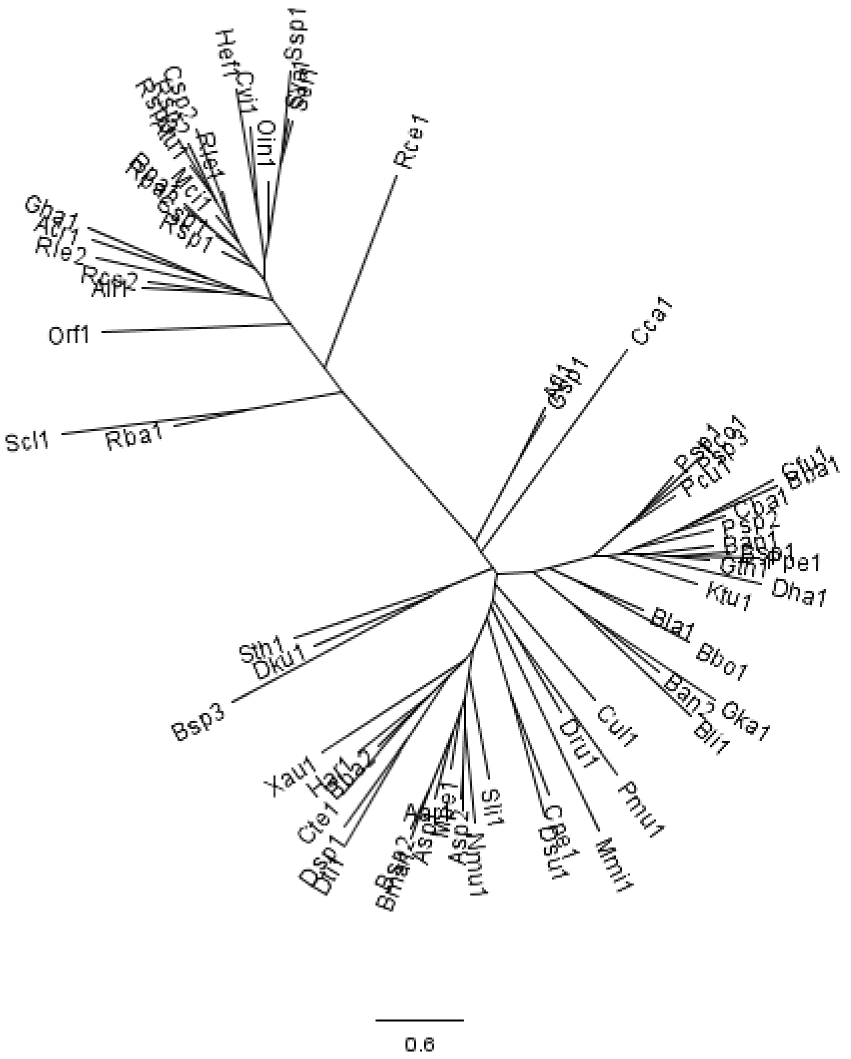

Fig. 25A

NAAT– Clustal

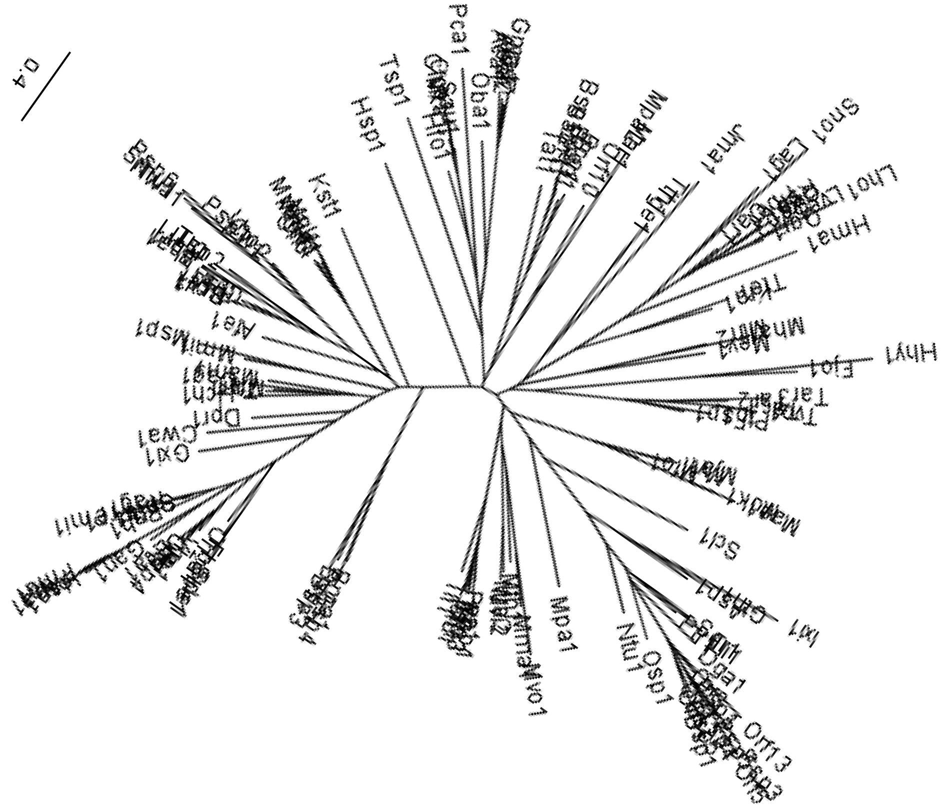

Fig. 25B

NAAT - Mafft

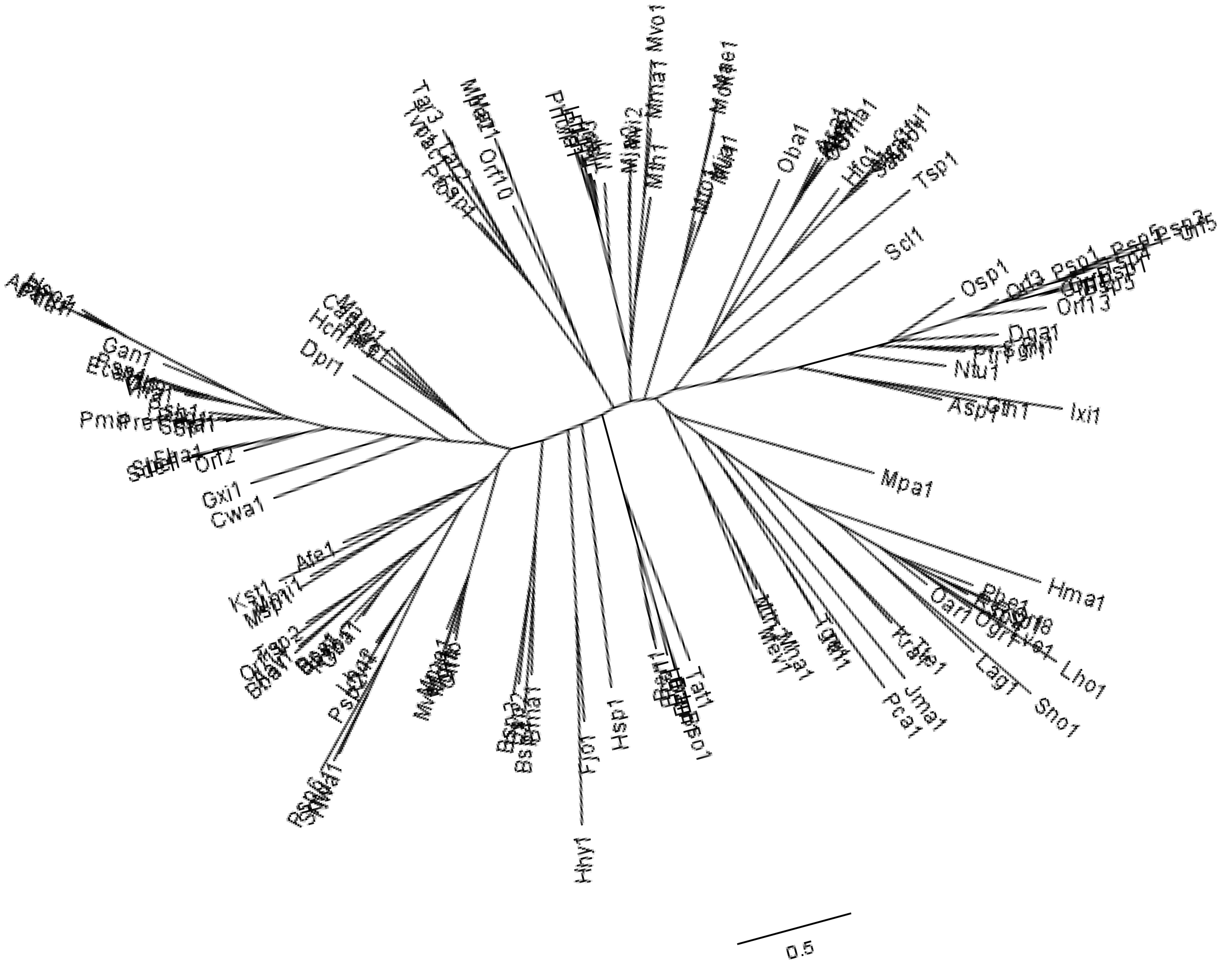

Fig. 25C

NAAT - ProbCons

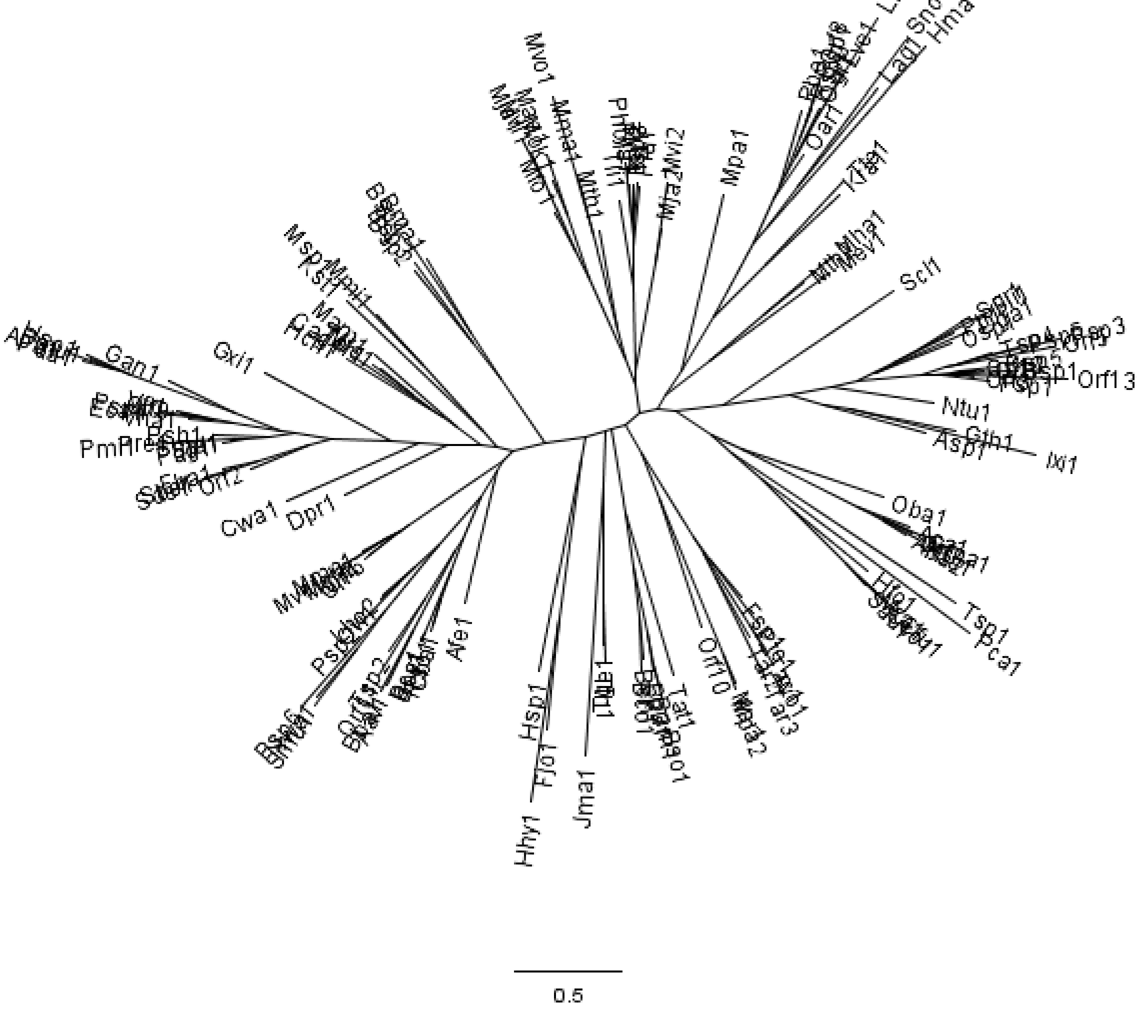

Fig. 26A

NicO – Clustal

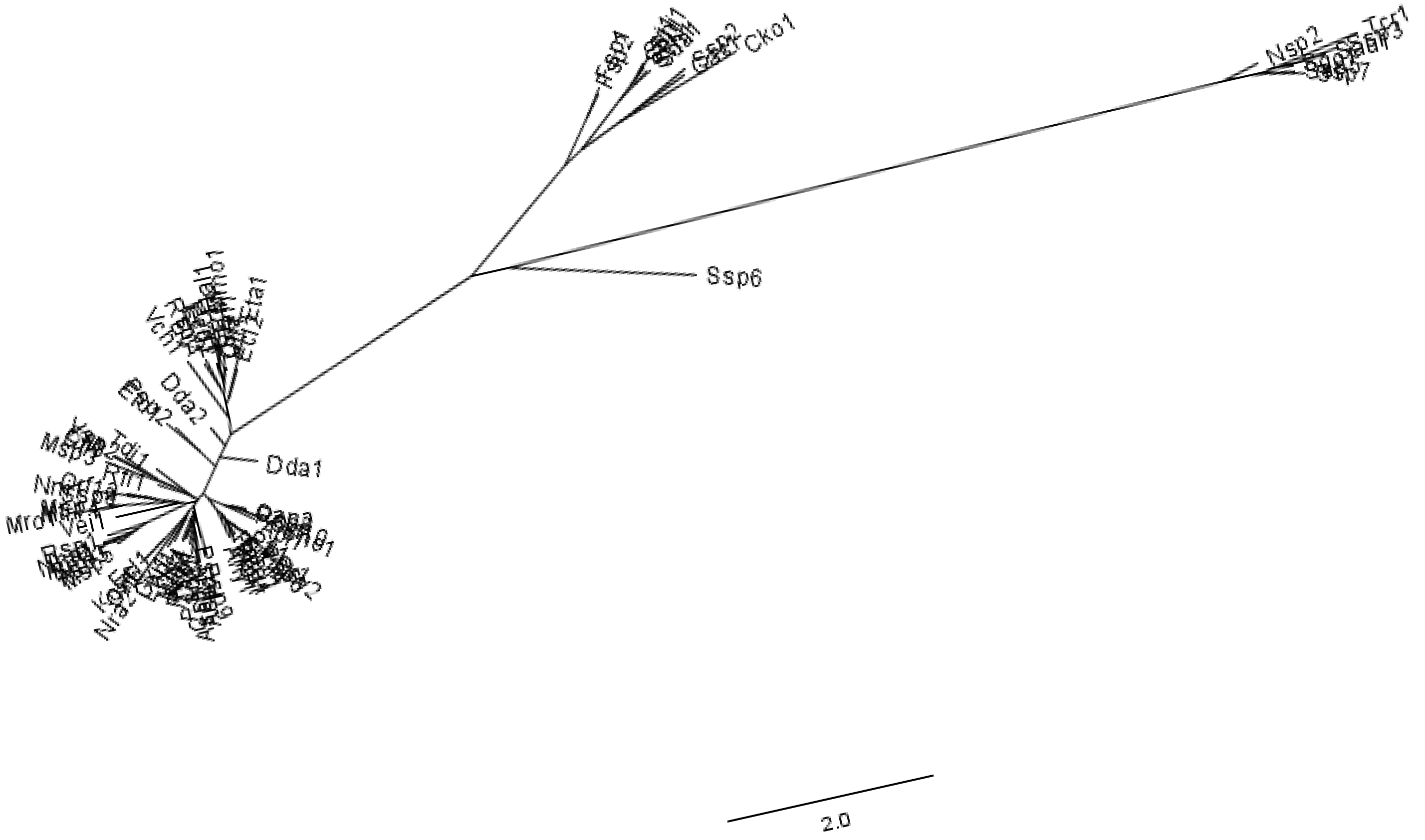

Fig. 26B

NicO - Mafft

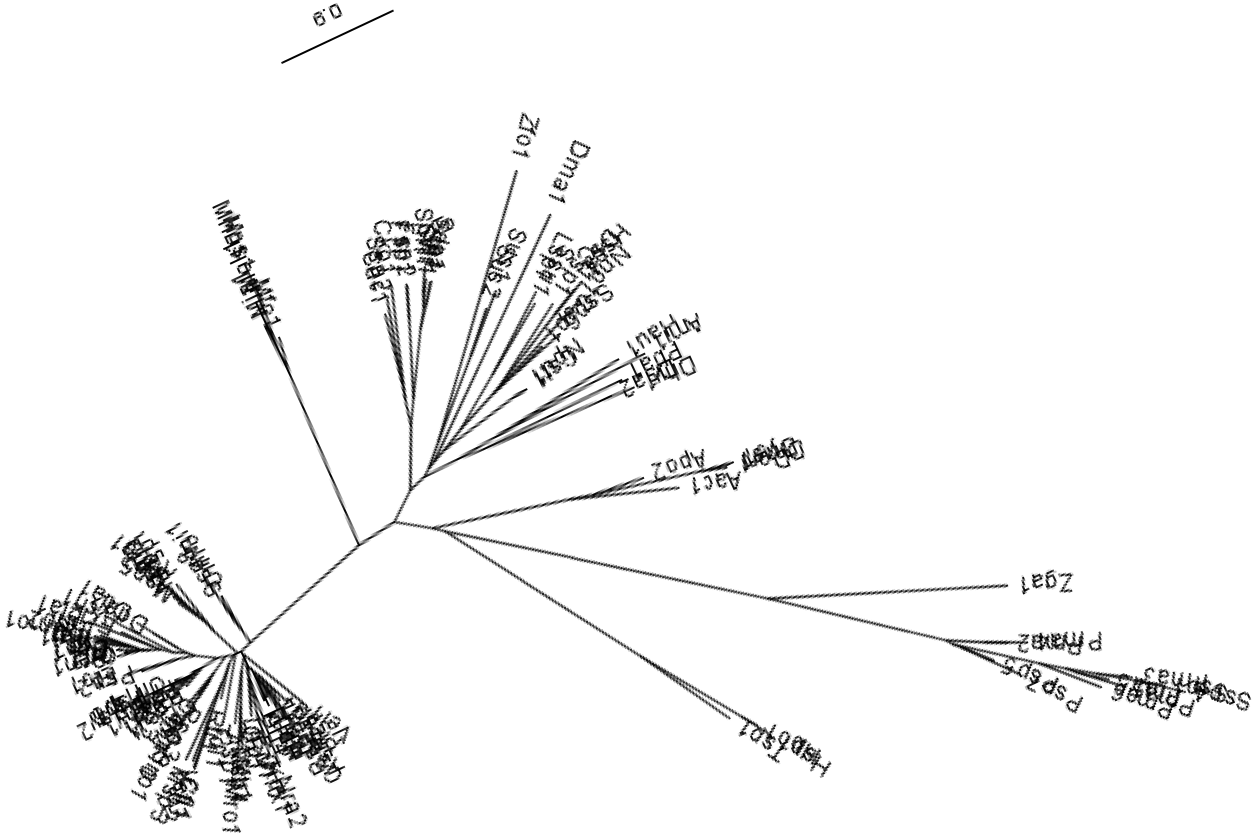

Fig. 26C

NicO - ProbCons

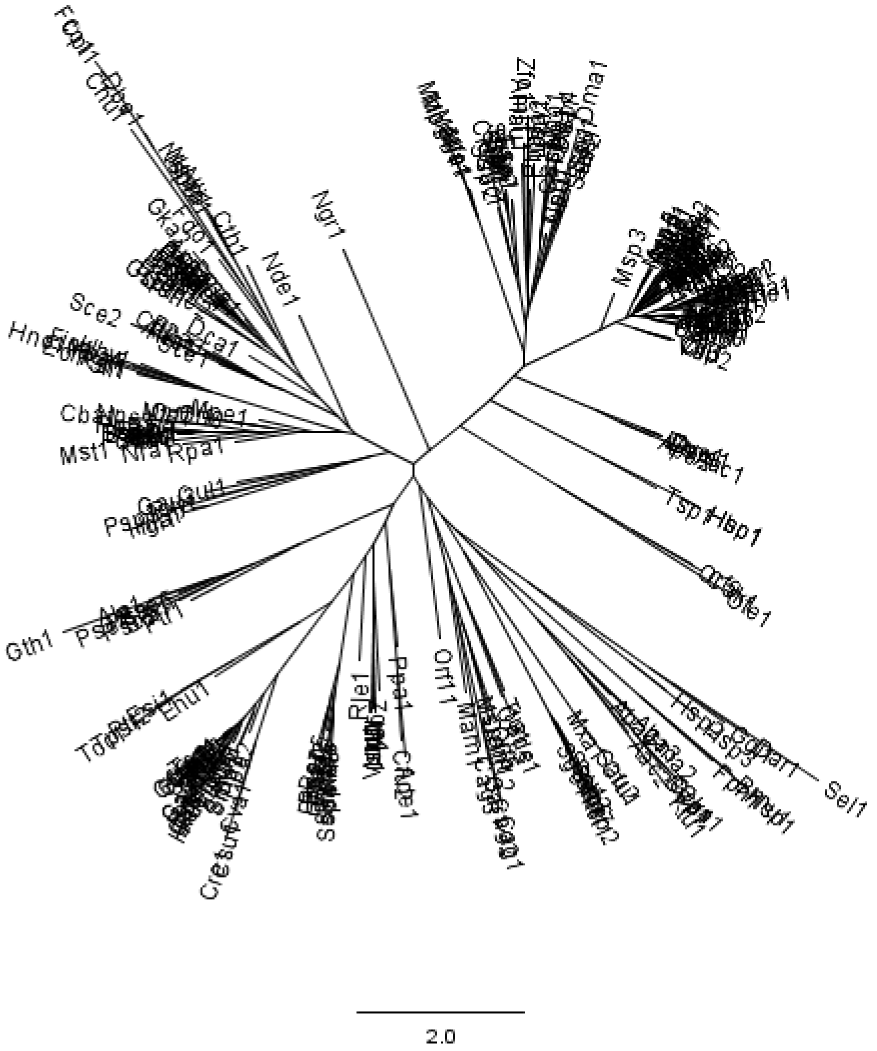

Fig. 27A

GAP – Clustal

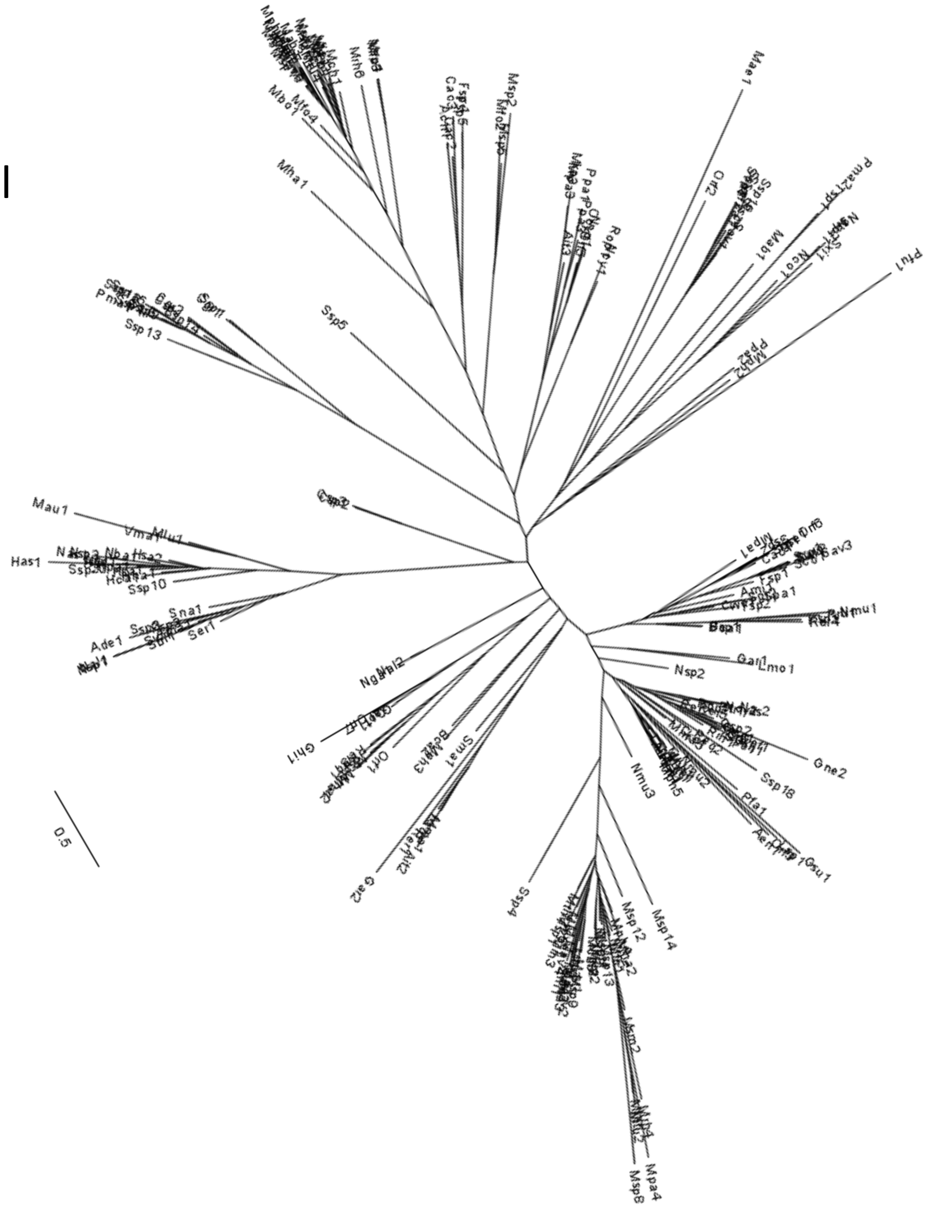

Fig. 27B

GAP - Mafft

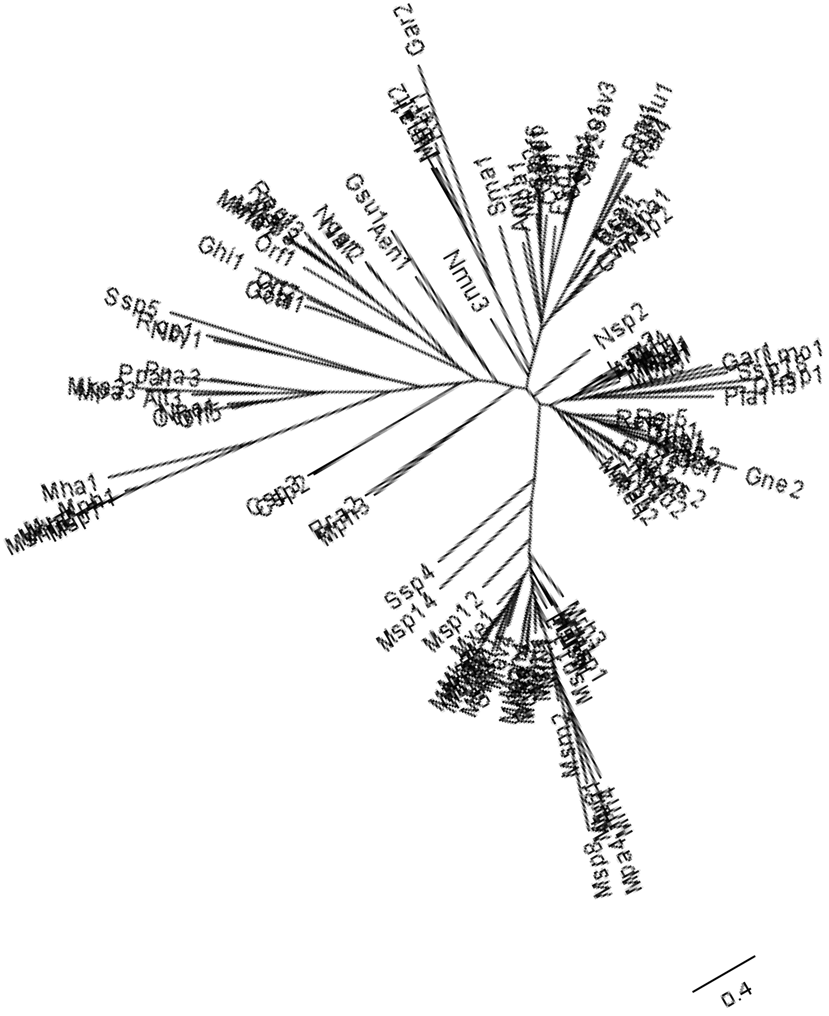

Fig. 27C

GAP - ProbCons

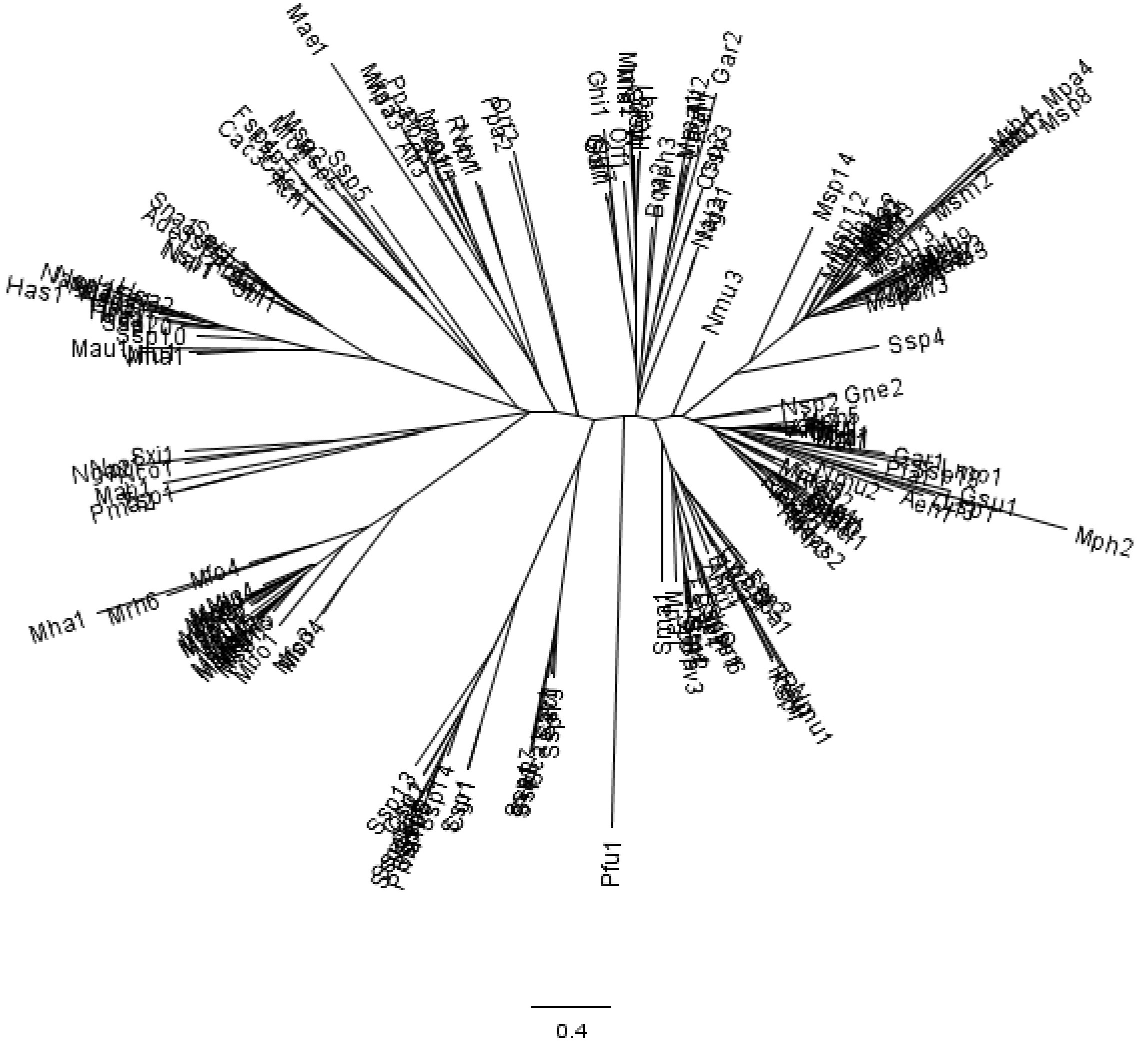

Fig. 28A

DsbD – Clustal

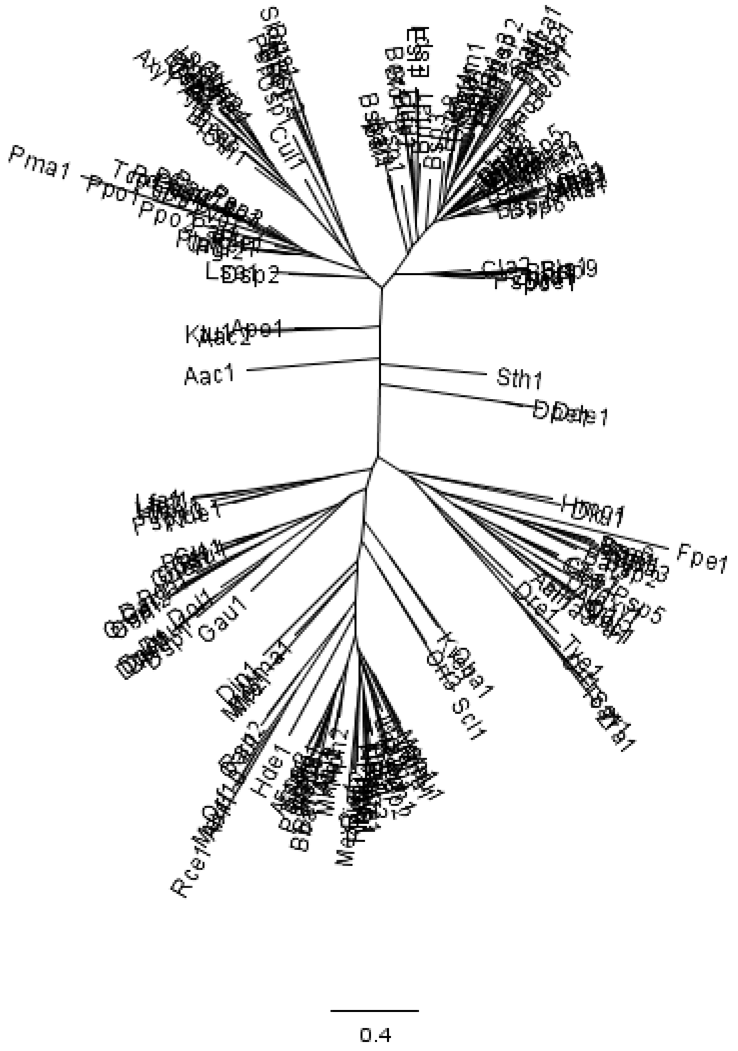

Fig. 28B

DsbD - Mafft

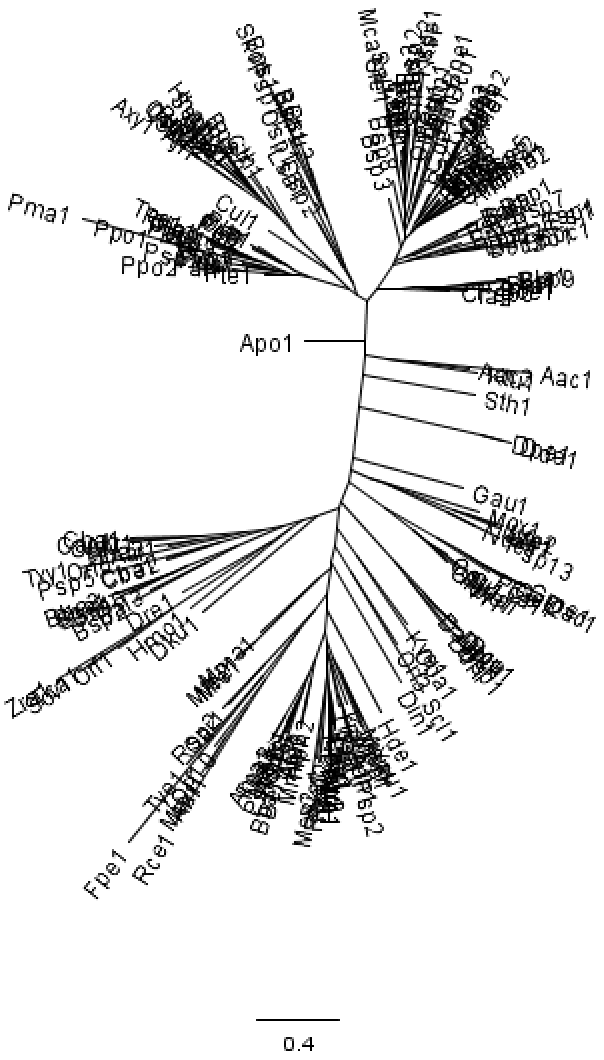

Fig. 28C

DsbD - ProbCons

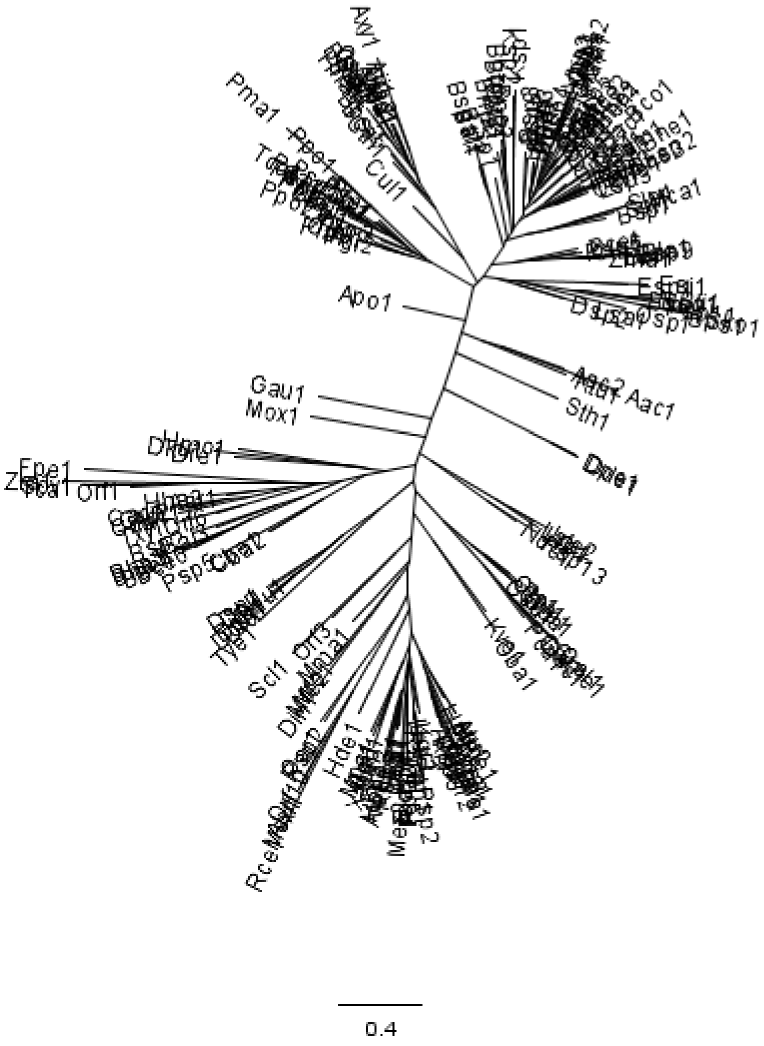

Supplement: S7 Supporting Information — The corresponding PDF file contains the S18–S28 Figs described previously. (PDF) [file pone.0137184.s036.pdf]
